# Transcriptome sequencing and histology reveal dosage compensation in the liver of triploid pre-smolt Atlantic salmon

**DOI:** 10.1038/s41598-020-73814-6

**Published:** 2020-10-08

**Authors:** Derrick K. Odei, Ørjan Hagen, Stefano Peruzzi, Inger-Britt Falk-Petersen, Jorge M. O. Fernandes

**Affiliations:** 1 Faculty of Biosciences and Aquaculture, Nord University, 8049 Bodø, Norway; 2 Faculty of Biosciences, Fisheries and Economics, University of Tromsø-The Arctic University of Norway, 9035 Tromsø, Norway

**Keywords:** Molecular biology, Transcriptomics

## Abstract

Triploid Atlantic salmon (*Salmo salar* L.) is seen as one of the best solutions to solve key issues in the salmon farming industry, such as the impact of escapees on wild stocks and pre-harvest sexual maturation. However, the effects of triploidy on salmon smoltification are poorly understood at the molecular level, even though smoltification is a very sensitive period that has a major influence on survival rate and performance of farmed salmon. In this study, we have compared the liver transcriptomes of diploid and triploid Atlantic salmon at three ontogeny stages: fry, parr and smolt. In diploid fish, a total of 2,655 genes were differentially expressed between fry and parr, whereas 506 genes had significantly different transcript levels between parr and smolts. In triploids, 1,507 and 974 genes were differentially expressed between fry and parr, and between parr and smolts, respectively. Most of these genes were down-regulated and 34 genes were differentially expressed between ploidies at the same stage. In both ploidy groups, the top differentially expressed genes with ontogeny stage belonged to common functional categories that can be related to smoltification. Nucleotide and energy metabolism were significantly down-regulated in fry when compared to parr, while immune system processes were significantly down-regulated in parr when compared to smolts. The close resemblance of enriched biological processes and pathways between ploidy groups suggests that triploidy is regulated by genome dosage compensation in Atlantic salmon. Histological analysis revealed that areas of vacuolization (steatosis) were present only in fry and parr stages, in contrast to a compact cellular histology with glycogen granules after smoltification. There was no significant difference in vacuolization between ploidy groups at the fry stage but the liver of diploid parr had a 33.5% higher vacuolization area compared to their triploid counterparts. Taken together, our data provide novel insights into the changes that occur at the molecular and histological level in the liver of both diploid and triploid Atlantic salmon prior to and during smoltification.

## Introduction

The use of functionally sterile triploid fish has the potential to improve post-pubertal growth, survival and carcass quality under culture conditions^1^. Sterility may also minimize the risk of genetic and ecological interactions between farmed and wild stocks in the event of accidental farmed fish escapees^2^. In Atlantic salmon (*Salmo salar* L.) aquaculture, escaped fish from sea cages represent one of the most persistent issues for sustainable development of the industry and have generated considerable concerns across all producing regions^3^. In Norway, farmed escapees are one of the main anthropogenic impact factors identified as an expanding threat to wild Atlantic salmon populations, besides sea lice infestations^4^. Genetic introgression from farmed salmon is reported to vary across rivers but remains widespread in Norwegian wild salmon populations, encouraging additional efforts to implement more robust production systems, escape prevention plans and other mitigation measures^5^.

Despite the potential of sterile triploid fish to contribute towards the development of a more sustainable and environmentally-friendly Atlantic salmon industry, uncertainties regarding the performance of cultured triploids have hindered their widespread adoption by most producers^2^. Regardless of the theoretical advantages that triploids might have over their diploid counterparts, comparative studies assessing their culture performance under different settings have produced “conflicting” results. For instance, a large and comprehensive study assessing the growth performance and sexual maturation in diploid and triploid Atlantic salmon of both sexes in seawater tanks under natural or continuous photoperiod regimes resulted in growth performance of triploid groups being equally or better in some cases when compared to their diploid counterparts^6^. In addition, a study assessing the performance of diploids and triploids in freshwater from hatching until smolt stages showed no significant differences in survival rate, but triploids were smaller in size at earlier stages^7^. Welfare issues such as high prevalence of skeletal deformities in addition to other potential limitations in triploid Atlantic salmon, including abnormal heart morphology and abdominal lesions has been highlighted to limit its performance and subsequent adoption by producers^8,9^. In addition, escaped triploid males have been shown to interfere with natural reproduction in the wild by competing with wild males for wild fertile females by stimulating spawning in Atlantic salmon^10^ and in Atlantic cod^11^ as well.

From a nutritional perspective, a gut morphology study comparing triploid and diploid Atlantic salmon revealed a lower pyloric caeca number and shorter relative gut length in triploid post-smolts compared to its diploid counterparts^12^. Contrary to expectations, the use of dietary hydrolysed fish proteins rich in free amino acids and low molecular peptides that may ease feed absorption had no positive influence on nutrient utilization and growth in triploids as in diploids^13^. Reports at the molecular level with emphasis on understanding the physiology and impact of triploidy on Atlantic salmon during ontogeny until post-smoltification are quite scarce. A study comparing growth-related gene expression profiles of diploid and triploid bighead catfish (*Clarias macrocephalus*) showed similar liver transcriptome responses in both ploidy groups^14^. In contrast, a molecular study showed that early nutritional programming for up to 6 weeks affected metabolic processes in the liver of diploid and triploid Atlantic salmon, where ploidy differences indicated the need for different ploidy-specific nutritional requirements for optimal performance^15^. A more recent study has shown that increase in dietary micronutrient levels led to vertebral expression of bone biomarker genes associated with reduced skeletal malformations in parr diploids and triploids but was found to be significant in the former^16^. Findings from these studies^12,13,16^ suggest that morphological, physiological and ploidy differences could play a role in determining digestive efficiency, nutritional requirements and subsequent growth and welfare of these fish.

Fish liver is involved in numerous functions including metabolism and immune system processes, to mention just a few. A study assessing the liver transcriptome response in Atlantic salmon fed diets with either fish oil or vegetable oil for up to 55 weeks revealed that there are genotype-specific metabolic responses at the molecular level^17^. Little is known about changes in the liver transcriptome of diploid and triploid Atlantic salmon prior to and during smoltification. Hence, we used RNA-seq to investigate potential differences in the liver transcriptome of diploid and triploid individuals at three ontogeny stages (fry, parr and smolts). This molecular approach was complemented by histological observations aimed at examining liver morphology and degree of steatosis in relation to ontogeny and ploidy over the same developmental stages.

## Results

### Characterization of RNA-seq libraries

**Figure 1:**
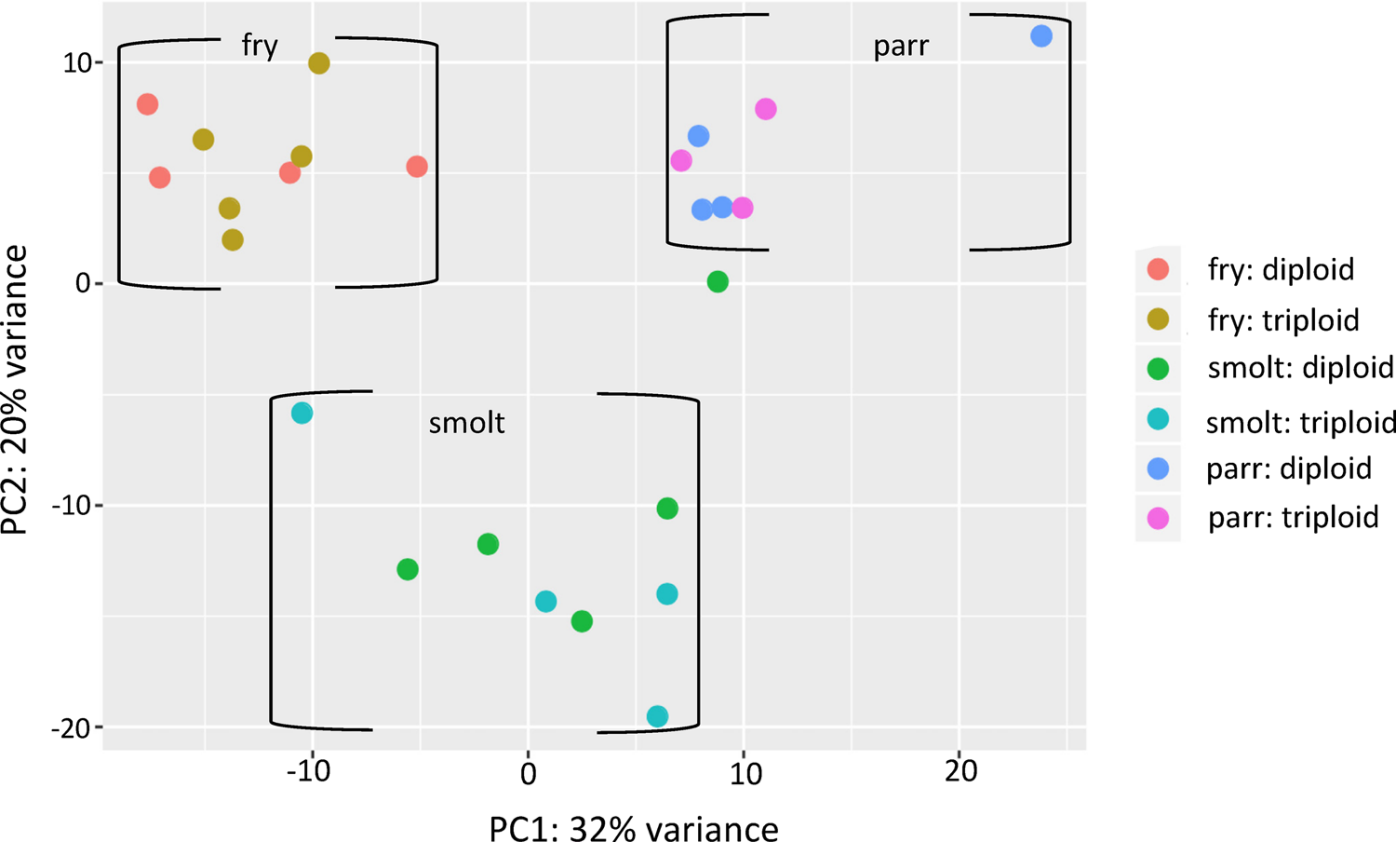
PCA plot for all sample points. All individual samples of diploids and triploids Clustering brackets for: fry, parr and smolts.

A similar number of raw reads was obtained for the three ontogeny groups, ranging from 7.9 to 40.9 million sequences (Supplementary info file, Fig. S1). After trimming, a total of 7.9 to 35.6 million reads remained and the overall mapping efficiency of these high-quality reads against the Atlantic salmon reference genome ranged from 82 to 99%. Three samples with total number of raw reads below 5 million and one extreme outlier were excluded from further analysis. As observed in the PCA plot (Fig. 1), all individuals from fry and parr groups clustered together, whereas one smolt sample was closer to the parr cluster. This could be attributed to late smoltification in this individual. Diploid and triploid fish within each group were found in the same cluster.

### Liver transcriptome differences with ontogeny and ploidy

Comparisons of liver transcriptomes between diploid and triploid fish at the same stage revealed that only 34 genes in total were differentially expressed between diploid and triploid salmon at a false discovery rate (FDR < 0.05) (Supplementary info file, Table S1). The number of differentially expressed genes (DEGs) identified when diploid is compared to triploid fish was highest at the parr stage, with 25 DEGs (17 up- and 8 down-regulated in diploid fish when compared to triploid counterparts and corresponding maximum fold changes of 3.3 for up-regulated and − 4.9 for down-regulated DEGs respectively). In contrast, there were 5,642 DEGs between successive ontogeny stages within each ploidy (Supplementary info file, Table S2).

**Figure 2:**
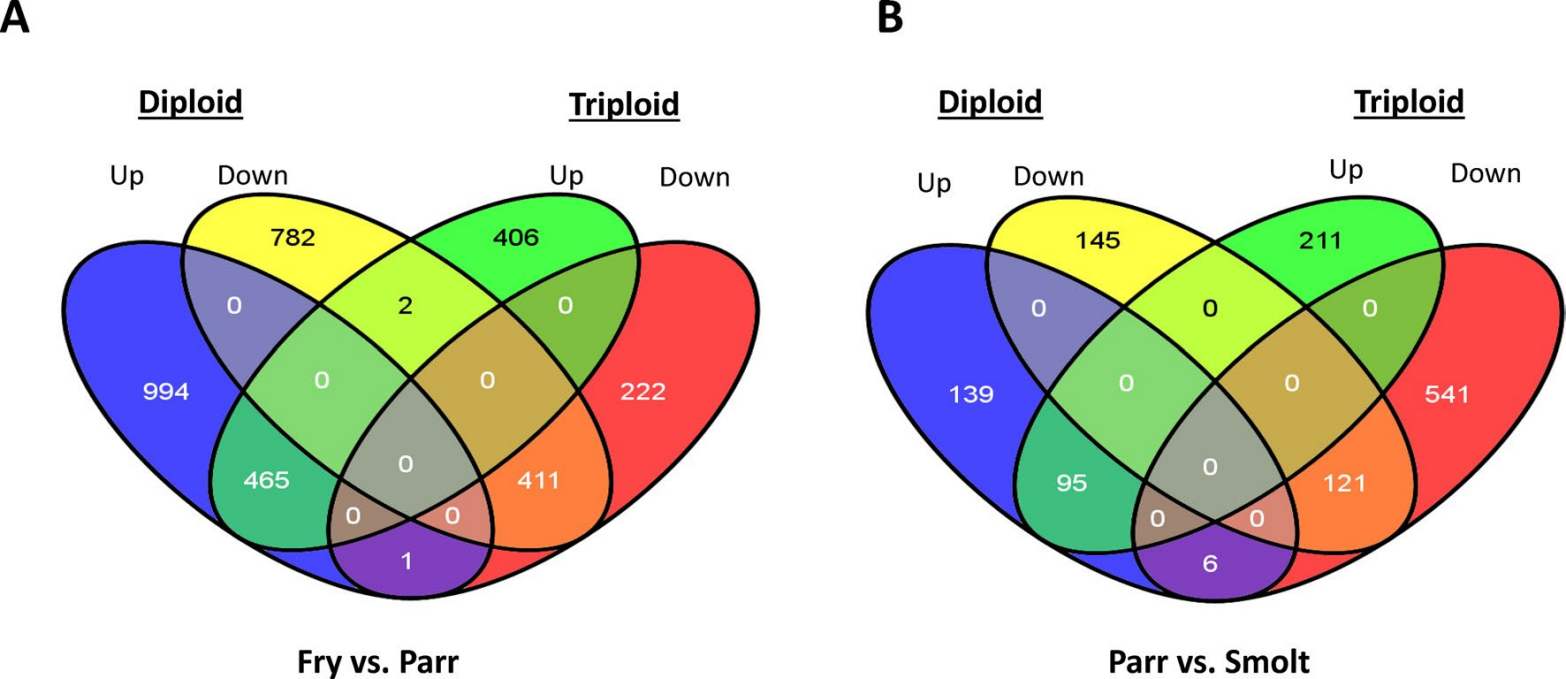
Venn diagrams for differentially expressed genes identified for comparisons between ‘fry vs. parr’ and ‘parr vs. smolt’ of diploid and triploid groups.

The largest number of DEGs was observed when fry was compared to parr. There were 2,655 DEGs for diploid fry when compared to their parr counterparts, of which 1460 were up- and 1195 were down-regulated, with corresponding maximum fold changes of 8.7 (Table 1) and − 24.7 (Table 2). Triploid fry when compared to parr showed 873 up-regulated DEGs with a maximum fold-change of 44.2 (Table 1) and 634 down-regulated with fold changes down to − 6.9 (Table 2). It is noteworthy that 465 and 411 up- and down-regulated DEGs were common between diploid and triploid fry when compared to their parr counterparts. A single DEG was up-regulated in diploids and down-regulated in triploids (Fig. 2A).

**Table 1:** Up-regulated DEGs in diploid and triploid fry compared to parr. Fold changes (FC) and q values (p adj.) are indicated.

Gene/locus	Description	FC	*q*	Function
**Diploid**				
*cpt1b*	Carnitine palmitoyltransferase 1B	8.7	0.00	Integral component of cell membrane
*gtaJ*	GATA zinc finger domain-containing protein 10-like	8.5	0.00	DNA binding transcription factor
*cpt1a*	Carnitine O-palmitoyltransferase 1, liver isoform-like	7.8	0.00	Integral component of membrane
*acot11*	Acyl-coenzyme A thioesterase 11-likenter	7.8	0.04	Lipid binding
*bhlhb2*	Class E basic helix-loop-helix protein 40-like	7.7	0.00	Regulation of DNA transcription
*loc106571795*	n/d	7.6	0.02	n/d
*loc106612462*	n/d	7.5	0.01	n/d
*c1ql2*	Complement C1q-like protein 2e 2-like	7.3	0.02	n/d
*irs2*	Insulin receptor substrate 2-likeor	7.2	0.00	n/d
*bnip3l*	BCL2/adenovirus E1B 19 kDa protein-interacting protein 3-like	7.0	0.00	Positive regulation of apoptotic processes
**Triploid**				
*adh1*	Alcohol dehydrogenase 1-like	44.2	0.00	Alcohol metabolism and oxidative stress
*urgcp*	Up-regulator of cell proliferation-like	12.4	0.00	GTP binding
*loc106584294*	n/d	11.3	0.00	n/a
*inhba*	Inhibin beta A chain-like	10.9	0.04	Growth factor activity and Disulfide bond
*fads6*	Fatty acid desaturase 6-like	10.3	0.00	Lipid metabolism
*taar13C*	Trace amine-associated receptor 13c-like	8.6	0.00	G protein receptor, integral component of membrane
*c1ql2*	Complement C1q-like protein 2	8.1	0.01	n/d
*cox7r*	Cytochrome c oxidase subunit 7A-related protein, mitochondrial	7.7	0.00	Transmembrane
*slc6a13*	Sodium- and chloride-dependent GABA transporter 2-like	7.3	0.01	Integral component of membrane
*ifi44*	Interferon-induced protein 44-like	6.6	0.00	Viral defence

n/d: not determined.

**Table 2:** Down-regulated DEGs in diploid and triploid fry compared to parr. Fold changes (FC) and q values (p adj.) are indicated.

Gene/locus	Description	FC	*q*	Function
**Diploid**				
*pck1*	Phosphoenolpyruvate carboxykinase 1	− 24.7	0.00	Involved in glucogenesis
*lipc*	Hepatic triacylglycerol lipase-like	− 19.4	0.00	Lipid metabolism
*dcor1*	Ornithine decarboxylase 1-like	− 11.1	0.00	Polyamine metabolism
*pfkfb3*	6-phosphofructo-2-kinase/fructose-2,6-biphosphatase 3	− 8.3	0.00	Fructose metabolism
*pla2g3*	Group 3 secretory phospholipase A2-like	− 7.5	0.03	Phospholipid metabolism
*loc106603905*	n/a	− 7.0	0.01	n/d
*cyp7a*	Cholesterol 7-alpha-monooxygenase-like	− 6.9	0.00	Bile acids biosynthesis, cellular response to glucose stimulus, cholesterol catabolism and homeostasis
*dd1t4*	DNA damage-inducible transcript 4 protein-like	− 6.2	0.00	Negative regulation of signal transduction
*lipe*	Lipase, hormone-sensitive	− 6.1	0.00	Lipid metabolism
*ppp1r3b*	Protein phosphatase 1 regulatory subunit 3B	− 5.5	0.00	Glycogen metabolism
**Triploid**				
*lipc*	Hepatic triacylglycerol lipase-like	− 6.9	0.00	Lipid metabolism
*dcor1*	Ornithine decarboxylase 1-like	− 5.4	0.00	Polyamine biosynthesis
*impa1*	Importin subunit alpha-1-like	− 4.9	0.02	Protein import into nucleus
*cybg2*	Cytoglobin-2-like	− 4.7	0.00	Heme, ion and oxygen binding
*tubb4b*	Tubulin beta 4B class IVb	− 4.7	0.01	Microtubule based processes
*gilt*	Gamma-interferon-inducible lysosomal thiol reductase	− 4.7	0.03	Signal peptide
*ima2*	Importin subunit alpha-1-like	− 4.6	0.00	Protein import into nucleus
*ret7*	Retinoid-binding protein 7-like	− 4.3	0.00	Lipid binding
*ppp1r3b*	Protein phosphatase 1 regulatory subunit 3B	− 4.3	0.00	Glycogen metabolism
*aurkb*	Aurora kinase B-like	− 4.3	0.00	Cell cycle

n/d: not determined.

There were 506 DEGs between diploid parr and smolt, of which 240 were up-regulated with a maximum fold change of 9.6 (Table 3) and 266 were down-regulated (Table 4). The liver transcriptome of triploid parr when compared to their smolt counterparts showed 974 DEGs (306 up- and 668 down-regulated with maximum fold changes of 6.0 (Table 3) and − 12.2 (Table 4), respectively). Furthermore, there were 95 and 121 up- and down-regulated DEGs in common to diploid and triploid fish when comparing parr to smolts, respectively. Only 6 DEGs were up-regulated in diploids and down-regulated in triploids (Fig. 2B).

**Table 3:** Up-regulated DEGs in diploid and triploid parr compared to smolt. Fold changes (FC) and q values (p adj.) are indicated.

Gene/Locus	Description	FC	*q*	Function
**Diploid**				
*pck1*	Phosphoenolpyruvate carboxykinase 1	9.6	0.03	Involved in glucogenesis
*gnai2*	Guanine nucleotide-binding protein G(i) subunit alpha-2	9.0	0.00	Adenylate cyclase-modulating G-proteins coupled receptor pathway
*loc106566987*	n/d	6.5	0.00	n/d
*lpin1*	Phosphatidate phosphatase LPIN1-like	6.4	0.00	Lipid metabolism
*odc1*	Ornithine decarboxylase 1	6.3	0.01	Polyamine biosynthesis
*cyp2r1*	Vitamin D 25-hydroxylase-like	6.1	0.00	Heme and ion binding, monooxygenase and oxidoreductase activity
*cyp7a1*	Cholesterol 7-alpha-monooxygenase-like	5.6	0.00	Bile acids biosynthesis, cellular response to glucose stimulus, cholesterol catabolism and homeostasis
*4ebp*	Eukaryotic translation initiation factor 4E binding protein 3–1	4.9	0.00	Negative regulation of translational initiation
*epo*	Erythropoietin-like	4.8	0.00	Erythrocyte maturation
*ga45b*	Growth arrest and DNA damage-inducible protein	4.7	0.00	Regulation of cell cycle and response to stress
**Triploid**				
*loc106566987*	n/d	6.0	0.00	n/d
*c2cd4c*	C2 calcium-dependent domain-containing protein 4C-like	5.6	0.00	n/d
*cish*	Cytokine inducible SH2 containing protein	5.3	0.03	Intracellular signal transduction, protein ubiquitination and others
*cdh6*	Cadherin-6-like	5.0	0.03	Homophilic cell adhesion via plasma membrane
*fkbp5*	FK506 binding protein 5	4.9	0.03	Heat shock protein binding
*cyp2k1*	Cytochrome P450 2K1-like	4.8	0.02	Heme and ion binding and oxidoreductase activity
*bhmt*	Betaine–homocysteine S-methyltransferase 1-like	4.8	0.03	Methionine biosynthesis
*4ebp*	Eukaryotic translation initiation factor 4E binding protein 3–1	4.7	0.02	Negative regulation of translational initiation
*cyp27b1*	25-hydroxyvitamin D-1 alpha hydroxylase, mitochondrial-like	4.6	0.04	Heme and ion binding, monooxygenase and oxidation
*odc1*	Ornithine decarboxylase 1-like	4.5	0.00	Polyamine biosynthesis

n/d: not determined.

**Table 4:** Down-regulated DEGs in diploid and triploid parr compared to smolt. Fold changes (FC) and q values (p adj.) are indicated.

Gene/locus	Description	FC	*q*	Function
**Diploid**				
*gtaJ*	GATA zinc finger domain-containing protein 10-like	− 5.4	0.00	DNA binding transcription factor
*ccl19*	C–C motif chemokine 19-like	− 5.3	0.00	Immune processes
*gvinp1*	Interferon-induced very large GTPase 1-like	− 5.1	0.00	GTP binding
*bhlhb2*	Class E basic helix-loop-helix protein 40-like	− 4.1	0.00	Regulation of transcription, DNA templated
*scyb7*	Platelet basic protein	− 4.1	0.00	Immune system processes
*egr1*	Early growth response protein 1-like	− 4.0	0.00	Regulation of transcription, DNA templated
*fcn1*	Ficolin-1-like	− 3.9	0.01	Disulphide bond
*loc106575521*	n/d	− 3.9	0.00	n/d
*irf-1*	Interferon regulatory factor 1	− 3.8	0.01	Apoptotic processes immune response
*arrdc3*	Arrestin domain-containing protein 3-like	− 3.5	0.00	Signal transduction
**Triploid**				
*ifi44*	Interferon-induced protein 44-like	− 12.2	0.00	Viral defence
*adh1*	Alcohol dehydrogenase 1-like	− 8.5	0.05	Alcohol metabolism and oxidative stress
*c1ql2*	Complement C1q-like protein 2	− 8.1	0.04	n/d
*fgg*	Fibrinogen gamma chain-like	− 8.0	0.03	Platelet activation, protein polymerisation
*ccl19*	C–C motif chemokine 19-like	− 7.8	0.01	Immune processes
*loc106597213*	n/d	− 7.6	0.00	n/d
*gimap4*	GTPase IMAP family member 4-like	− 7.5	0.02	GTP binding
*gvinp1*	Interferon-induced very large GTPase 1-like	− 7.4	0.01	GTP binding
*ccl19*	C–C motif chemokine 19-like	− 7.3	0.01	Immune processes
*pisd*	Phosphatidylserine decarboxylase	− 7.2	0.00	Phosphatydyleethanolamine biosynthesis and protein auto processing

n/d: not determined.

### GO enrichment analysis for biological processes

**Figure 3:**
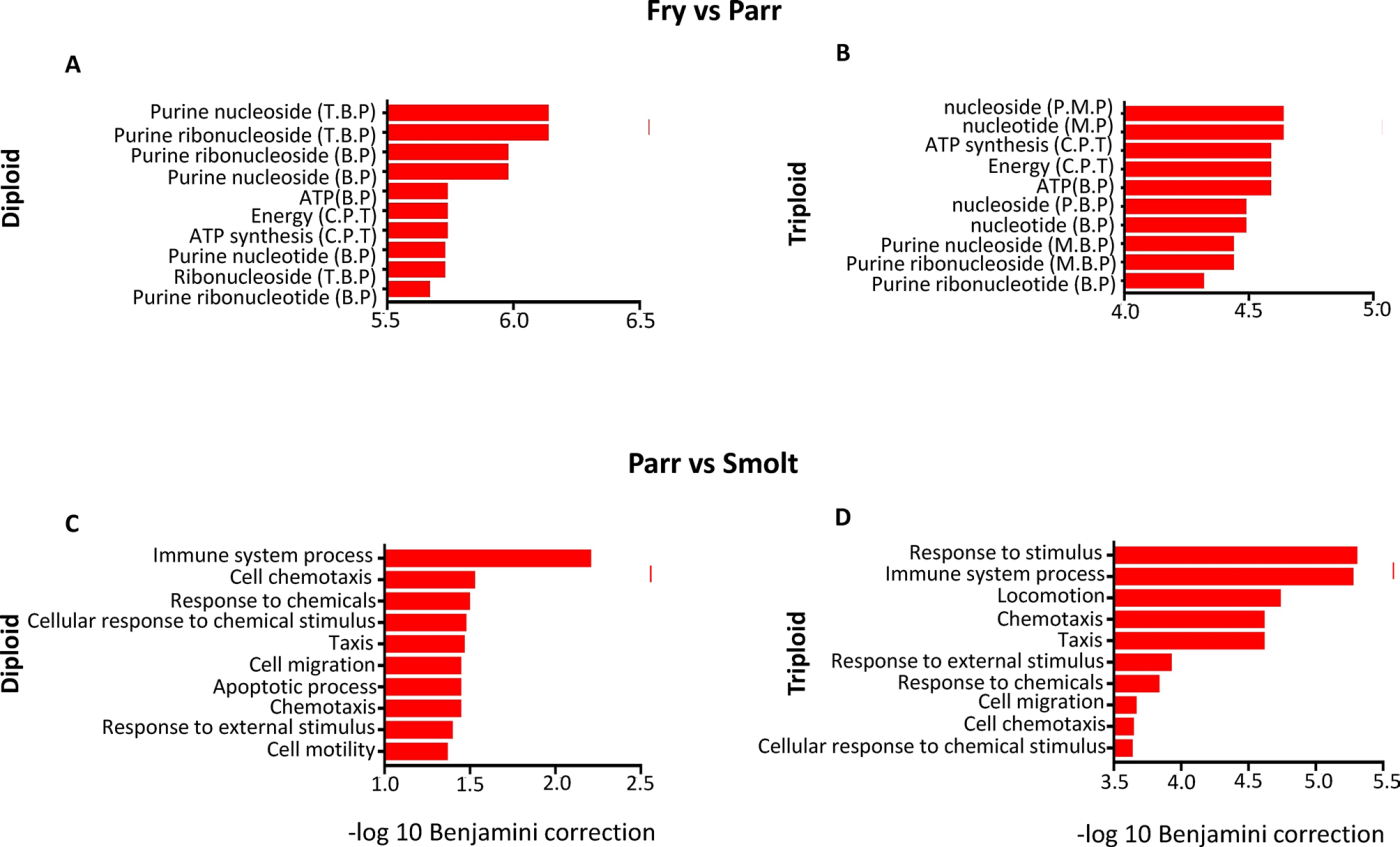
GO enrichment of down-regulated DEGs in fry when compared to parr in diploids (**A**) and respective triploids (**B**), while **C** (diploids) and **D** (triploids) represents GO enrichment of down-regulated DEGs in parr when compared to smolts. T.B.P (triphosphate biosynthetic process), B.P (biosynthetic process), C.P.T (coupled proton transport), P.M.P (phosphate metabolic process), M.P (metabolic process), M.B.P (monophosphate biosynthetic process).

Not all genes differentially expressed between diploid and triploid fish at the same stage had significantly enriched GO terms, while DEGs between successive stages within the same ploidy group showed significantly enriched GO terms (q < 0.05). There were 69 significantly enriched GO terms associated with down-regulated DEGs in diploid fry when compared to their parr counterparts (Supplementary info file, Table S3). Of these, approximately 91% were involved in metabolism and 9% were involved in cellular and other processes. Within comparisons between triploid fry and parr, there were 77 enriched GO terms (q < 0.05), only associated with down-regulated genes between stages (Supplementary info file, Table S3). Similarly to the diploid groups, 81% were involved in metabolism and 19% were involved in cellular and other processes. The top GO terms identified for fry when compared to parr in both ploidy groups were related to either nucleotide or energy metabolism (Fig. 3 A,B). A total of 18 GO terms were significantly enriched (q < 0.05) within the down-regulated genes between parr diploids and smolt diploids, and were involved in immunity (50%) or cellular processes (50%) (Supplementary info file, Table S4). There were 17 significantly enriched GO terms (q < 0.05) associated with the down-regulated genes for triploid parr when compared to their smolt counterparts. (Supplementary info file, Table S4). Approximately 57% of these were involved in immunity and 43% were related to cellular processes. In both diploid and triploid fish, the top GO terms identified for parr when compared to smolt fish were associated with general or cell-mediated immune processes (Fig. 3 C,D).

### KEGG pathway analysis for down-regulated DEGs

The KEGG pathway analysis was performed on only down-regulated genes, since there were no significantly enriched GO terms associated with up-regulated genes. Diploid fry when compared to their parr counterparts had 17 KEGG pathways linked to 6 functional categories (Supplementary info file, Table S5): metabolism, genetic information processing, environmental processing, cellular processing, organismal systems and human diseases. DEGs down-regulated between triploid fry and parr were involved in 12 KEGG pathways associated with 5 functional categories (Supplementary info file, Table S6): genetic information processing, cellular processing, organismal systems and human diseases. There were 18 distinct KEGG pathways for diploid parr versus smolts (Supplementary info file, Table S7), which were associated with 6 functional categories: metabolism, genetic information processing, environmental processing, cellular processes, organismal systems and human diseases. The 10 KEGG pathways associated with down-regulated genes for triploid parr when compared to their smolt counterparts were linked to the functional categories genetic information processing, environmental processing and cellular processing (Supplementary info file, Table S8).

### Top regulated genes in diploid and triploid fish

The top up-regulated genes in diploid fry when compared to their parr counterparts belonged to functional categories including, cellular processes and lipid metabolism (Table 1). Alternatively, when triploid fry were compared to parr, top up-regulated DEGs in parr had relatively higher maximum fold-change values compared to diploids. A gene involved in alcohol metabolism and oxidative stress (*alcohol dehydrogenase 1-like*) was the most up-regulated in fry, with a fold-change of 44.2 when compared to smolt fish. Other up-regulated DEGs in this comparison were related to immune system and cellular processes (Table1).

Within diploids, most down-regulated DEGs for fry compared to parr were associated with either carbohydrate, lipid or other metabolic processes (Table 2). Notable genes involved in lipid metabolism were *hepatic triacylglycerol lipase-like*. Down-regulated genes found for triploid fry compared to parr counterparts had functions related to cellular processes (*importin subunit alpha-1-like*, *tublin beta 4B class IVb* and *aurora kinase B-like*), lipid metabolism (*hepatic triacylglycerol lipase-like*) and carbohydrate metabolism (Table 2). A gene involved in amino acid metabolism through polyamine biosynthesis (*ornithine decarboxylase 1-like*) was down-regulated in both diploid and triploid fry when compared to their parr counterparts.

Most up-regulated DEGs in diploid parr versus smolt counterparts were linked to metabolism, genetic information processes, immune system and other cellular processes (Table 3). Notable of all DEGs identified was, *ornithine decarboxylase 1*. On the other hand, DEGs up-regulated for triploid parr when compared to smolt were linked to cellular processes*,* polyamine biosynthesis (*ornithine decarboxylase 1-like*), genetic information processes (*eukaryotic translation initiation factor 4E binding protein 3–1*),heme and ion binding and oxidoreductase activity (Table 3). Down-regulated genes in diploid parr when compared to smolt were mostly involved in genetic information processes and immunity (Table 4).. Within triploids, the top down-regulated DEGs in parr when compared to smolt include metabolism, general immune system-related genes, alcohol metabolism and oxidative stress (*alcohol dehydrogenase 1-like*) (Table 4).

### Liver histology

**Figure 4:**
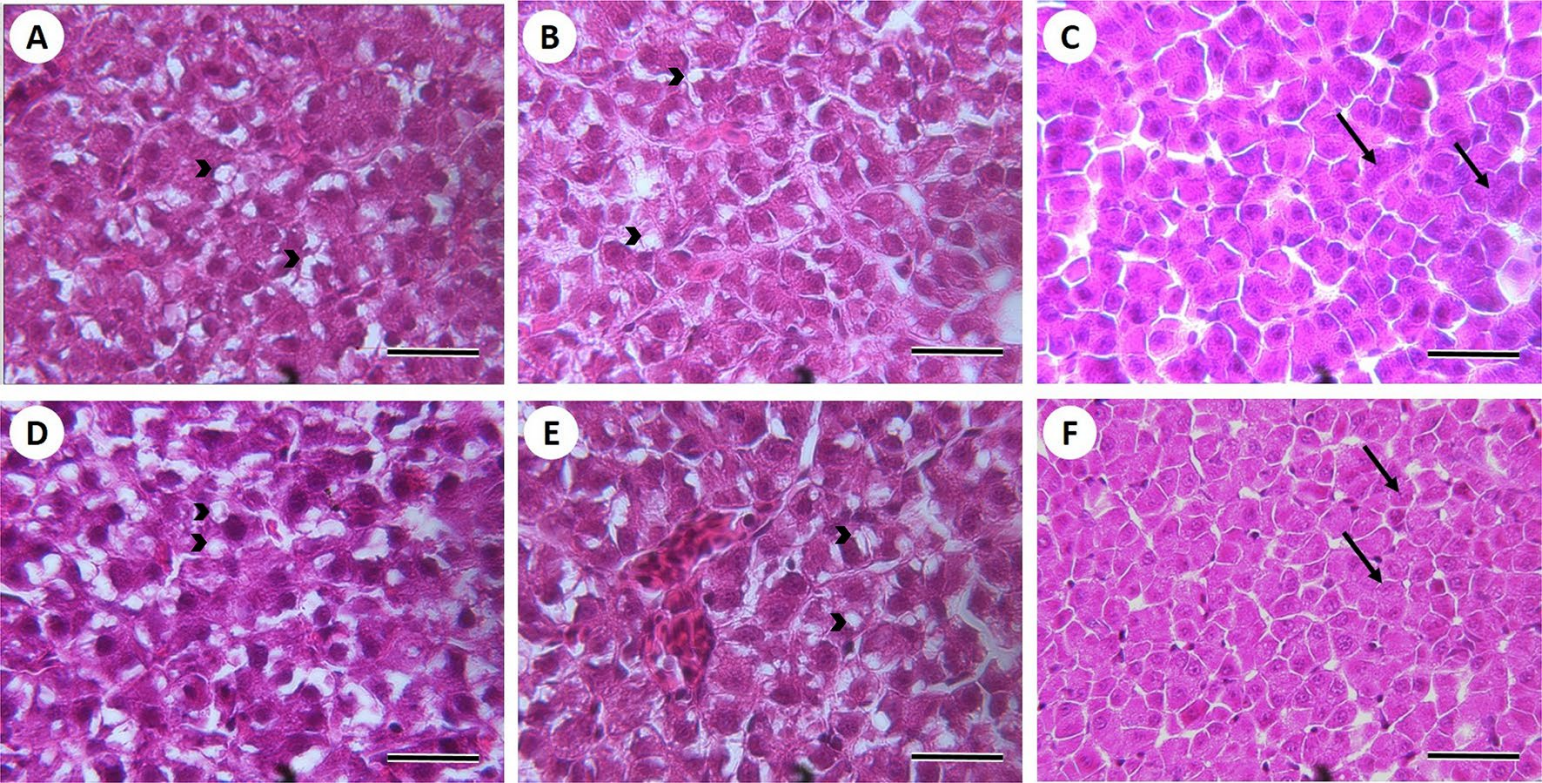
Liver sections (H&E staining) of diploid (**A**–**C**) and triploid (**D**–**F**) Atlantic salmon. The images show various degrees of vacuolization (arrowheads) in hepatocytes at fry (**A**,**D**) and parr (**B**,**E**) stage, and a compact cellular histology with glycogen granules (arrows) at smolt stage (**C**,**F**). Scale bars represent 50 µm.

**Figure 5:**
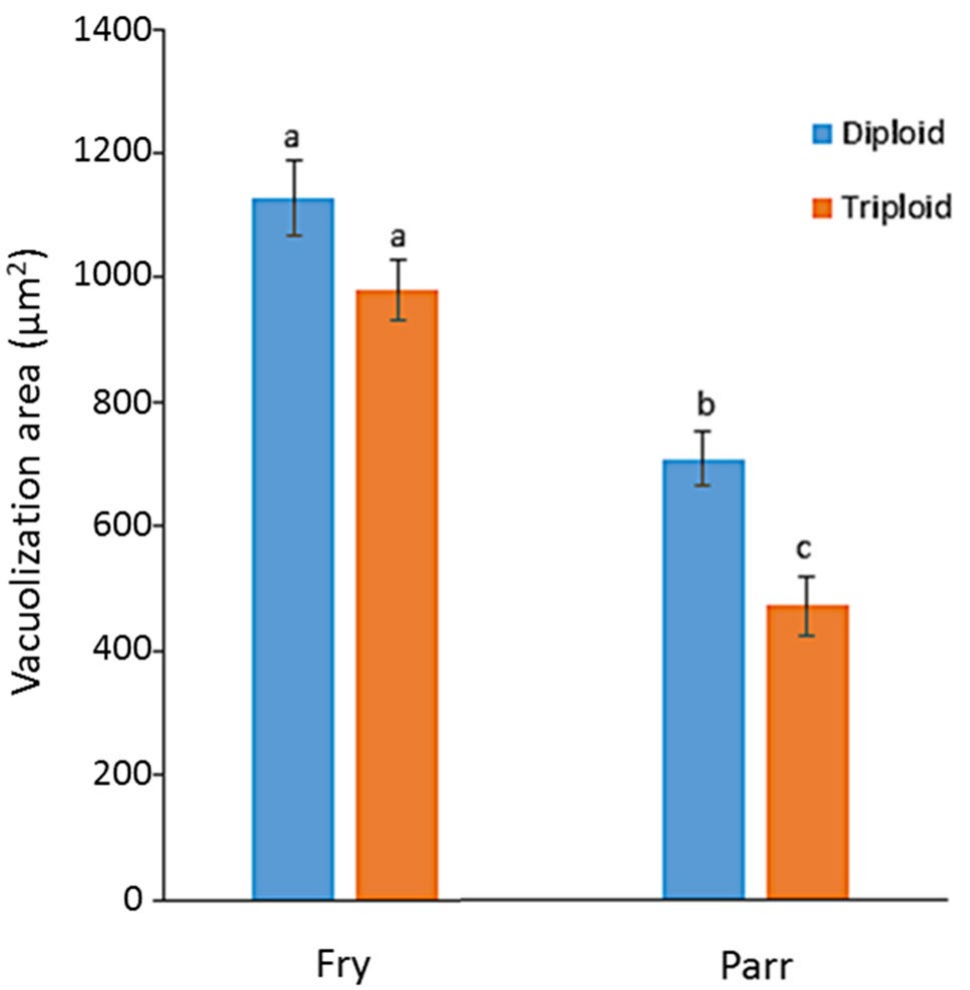
Quantification of liver steatosis (vacuolization area) in diploid and triploid Atlantic salmon at fry and parr stages. Data are presented as means ± SEM (n = 3). Different letters denote significant differences (P < 0.05).

There were no obvious differences in the general liver morphology between ploidy groups at fry, parr or smolt stages (Fig. 4). Liver steatosis was only observed and therefore quantified in the first two ontogeny stages. No sign of vacuolization in hepatocytes was found in smolts, which had only glycogen granules and a very compact cellular structure, independently of ploidy (Fig. 4). The degree of liver steatosis was similar between diploid and triploid individuals at the fry stage and significantly decreased (*P* < 0.001) in both ploidy groups at the parr stage. This was more pronounced in triploid parr, which had a degree of vacuolization significantly lower (*P* < 0.001) than their diploid counterparts (Fig. 5). The nuclear size (minor and major axis) of hepatocytes was significantly larger (*P* < 0.001) in triploid than in diploid fish at all three developmental stages (Table 5). The minor and major axis of hepatocytes in triploids measured 1.2–1.4 times and 1.2–1.3 times those of diploids, respectively, with a tendency for nuclear differences between ploidies to decrease, although not significantly, from fry to smolt stage.

**Table 5:** Nuclear size (minor and major axis) of hepatocytes in diploid and triploid Atlantic salmon from fry to smolt stage.

Stage	Minor axis (µm)				Major axis (µm)			
	Diploid	Triploid	Ratio*	*P*	Diploid	Triploid	Ratio*	*P*
Fry	0.79 ± 0.02^b^	1.09 ± 0.03^a^	1.38	< 0.001	0.97 ± 0.02^b^	1.28 ± 0.04^a^	1.32	< 0.001
Parr	0.87 ± 0.02^b^	1.08 ± 0.02^a^	1.24	< 0.001	0.99 ± 0.02^b^	1.25 ± 0.03^a^	1.26	< 0.001
Smolt	0.79 ± 0.01^b^	0.95 ± 0.02^a^	1.20	< 0.001	0.92 ± 0.01^b^	1.09 ± 0.02^a^	1.18	< 0.001

Data are presented as means ± SEM (n = 4–8 individuals). Different letters in the same row denote significant differences (Students t-test) between ploidy groups. *Ratio of means in hepatocytes of triploid over diploid fish.

## Discussion

We have found very similar liver transcriptomes between diploid and triploid Atlantic salmon at the same ontogeny stage. In contrast, gene expression levels changed substantially from fry to parr to smolt. Only the DEGs up-regulated during ontogeny (i.e., down-regulated in fry versus parr and parr versus smolt) were significantly enriched for biological processes and pathways in both diploid and triploid fish.

There were several down-regulated pathways in both diploid and triploid fry when compared to parr, namely genetic information processes, cellular processes and organismal systems. Some pathways involved in metabolism (carbohydrate, energy and amino acid) and environmental processes (signal transduction) were exclusively down-regulated between diploid fry and diploid parr. Nevertheless, the biological processes observed for the top 10 GO terms significantly enriched and down-regulated between fry and parr were identical in diploid and triploid fish. Most biological processes significantly down-regulated in fry when compared to parr were involved in either energy or nucleotide metabolism. Nucleotides are known to be involved in a variety of chemical functions, including energy metabolism^18,19^. Therefore, a down-regulation of nucleotide metabolism can potentially decrease energy metabolism. It has been reported that nucleotide deficiency negatively impacts the performance of key organs and processes, such as liver, heart, intestine and immune functions^20^. The higher levels of transcripts related to nucleotide and energy metabolism in parr when compared to fry are likely related to a surge in energy metabolism needed for subsequent smolt transformation, according to a previous report^21^.

Compared to parr, fry had lower transcript levels of genes related to lipid, carbohydrate and amino acid metabolism. In fish, a lower lipid content is known to favour increase in body water content and vice versa^22,23^. It has been reported that decline in lipid metabolism of salmonids during smoltification helps mobilise energy reserves (triglycerols) required for environment and ontogeny-associated changes^21^. Also, Wedemeyer et al.^24^ highlighted the contribution of lipid decline as salmonid develops into smolts towards silvering, increased growth rate, salinity tolerance and increased pituitary growth hormones in order to adapt to environmental-associated changes at sea. These findings demonstrate the importance of down-regulating lipid metabolism during ontogeny and environmental-associated changes encountered by salmonids during smoltification. In addition, the interactions between lipid, carbohydrate and amino acid metabolism decline in Atlantic salmon facilitate smoltification^24,25^. Lipid and carbohydrate depletion during smoltification are the results of increased lipolytic/glygenolytic enzyme activity, decrease in lipid/glycogen synthesis and increase in the rate of plasma fatty acid/glucose turn over^21^. Hence, the observed down-regulation of genes involved in lipid and carbohydrate metabolism in the liver of fry compared to parr probably favour mobilisation of energy required to reach the parr stage. Common to both ploidy groups for the same comparison was the down-regulation of *ornithine decarboxylase 1-like*, which is known to be involved in immune and inflammatory responses through directing arginine flux away from nitric oxide synthase and nitric oxide production by existing as a free radical toxic to bacteria and a signalling molecule^26^. Down-regulation of this gene in fry when compared to parr stages may be related to the susceptibility of Atlantic salmon fry to bacterial infections^27^ during transition to parr. Some genes involved in various cellular processes (e.g., *aurora kinase B-like* and *importin subunit alpha-1-like*) were down-regulated exclusively in triploid fry when compared to their parr counterparts. This could be due to the fact that triploidy leads to an overall reduced cell number in comparison to diploids, which limits their normal cellular functions and organ system processes to some extent^28^. We did not observe significant differences in hepatocyte number between diploid and triploid fish but our histology analysis was limited to the mid-portion of the liver.

Both diploid and triploid fry had relatively few up-regulated DEGs when compared to their parr counterparts and these DEGs belonged to similar functional categories, mostly related to cellular processes. A notable difference between ploidy groups was the 44-fold up-regulation exclusively in triploid fry of *adh1*, a gene involved in alcohol metabolism and oxidative stress. This may be linked to the fact that triploid salmonids tend to be more prone to oxidative stress than their diploid counterparts at juvenile^29^ and post-smolt stages^30^.

The enriched GO terms for down-regulated genes between parr and smolt fish were very similar in diploid and triploid fish, and they were related to cell-mediated immunity or general immune system processes. In both ploidy groups, the top significantly enriched GO terms down-regulated in parr when compared to smolts included taxis and chemotaxis, cellular response to chemical stimulus and cell migration, which indicate increased cell-mediated immunity and directional phagocytosis in smolts. Movement of microphages in fishes has already been demonstrated to be by either chemokinesis (a non-directional movement of the phagocyte) or chemotaxis (a directional movement of the phagocyte) in response to bacterial antigens in vivo or in vitro^31–33^. Cell-mediated immunity is particularly important in the elimination of intracellular pathogens^31^. A recent study showed a strong relationship between MHC and disease resistance of Atlantic salmon to infectious salmon anaemia^34^. The potential changes in the immune system of smolts is likely important for their survival at sea, where they will be exposed to new pathogens.

The most up-regulated DEGs in diploid and triploid parr when compared to smolts included genes involved in metabolism and cellular processes. For example, a gene involved in polyamine biosynthesis (*ornithine decarboxylase 1-like*) was up-regulated in parr with fold changes of 6.3 and 4.5 in diploids and triploids, respectively. The enzyme encoded by this gene is a key regulator in growth processes in vertebrates and has been positively correlated with specific growth rate in Atlantic salmon^35^. Its higher transcript levels in parr may be related to the rapid growth change known to occur in pre-smolts. Up-regulated metabolic rates could also be due to the fish being bigger in size and with a subsequently higher basal metabolic rate. The *eukaryotic translation initiation factor 4E binding protein 3–1* was also up-regulated in parr and it can affect growth, since it encodes a vital mRNA cap-binding protein that controls global translation rates^36^.

Similar KEGG pathways for down-regulated DEGs were observed in both diploid and triploid parr when compared to their smolt counterparts, and include genetic information processing, environmental processing and cellular processing. The down-regulated pathways specific to diploid parr were involved in lipid and amino acid metabolism and organismal system processes related to development.

**Figure 6:**
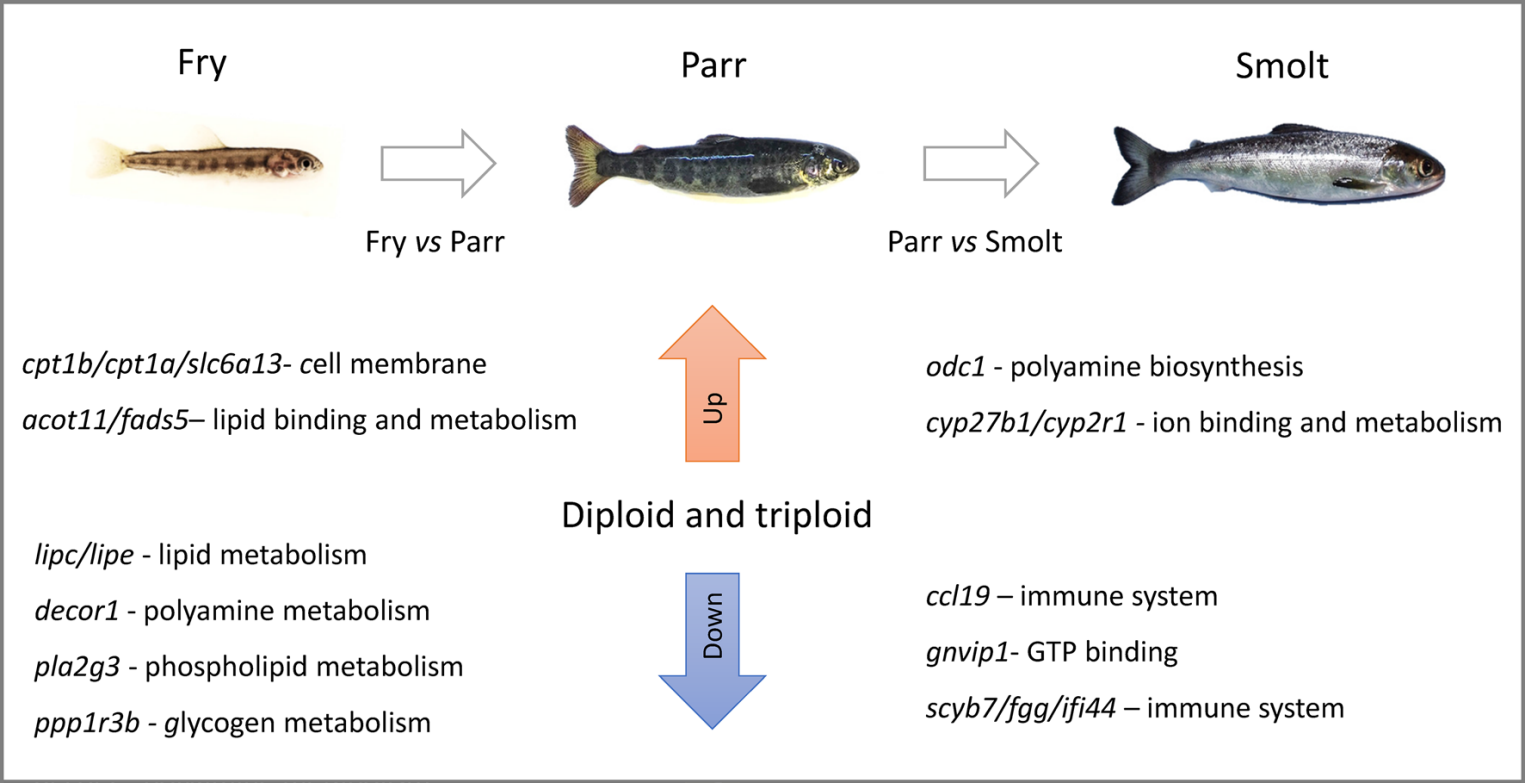
Overview of the changes in the Atlantic salmon liver transcriptome during ontogeny. There were very few differences between diploid and triploid individuals at the same stage, indicating a clear dosage compensation. The main DEGs between fry vs parr and parr vs smolt are indicated, along with their functions and significantly enriched GO terms.

The top up- and down-regulated genes in fry compared to parr and in parr versus smolt were remarkably similar in diploid and triploid fish. Moreover, the significantly enriched down-regulated GO terms showed a striking resemblance in functional categories for both ploidy groups (Fig. 6). Compared to mammals, lower vertebrates such as fish cope quite well with polyploidy^37,38^. Generally, polyploidisation is regulated through two processes: genome dosage effect or genome dosage compensation^39^. The genome dosage effect is reflected by a correlation between expressed genes and the number of chromosomes, while in the case of genome dosage compensation, gene expression for polyploids tends to mirror that of diploids^40^.

A recent study has shown that performance and growth of triploid Atlantic salmon is linked to the second maternal chromosome set^41^. These findings suggest that maternal and paternal contributions from diploid broodstock may not have equal weight when selecting for best performing triploids. To add up to these findings, a study determining dosage compensation using liver transcriptome of diploid parent grass carp (*Ctenopharyngodon idella*) and their triploid offspring showed similar expression levels in both ploidies where expression level dominance (ELD) was found to be biased towards the maternal genome^42^. In addition, homolog gene expression levels in this study showed the combined role of regulatory functions and epigenetics through a transcriptome network vital for adaptation during growth of triploids^42^ which is likely to be the case of this study. Generally, triploid salmon shows either a positive or negative dosage compensation when compared to diploid full-sibs counterparts^39,43^. Determining dosage compensation with focus on the liver can be questionable because the liver of eukaryotic organisms has been reported to form variable percentages of polyploidy cells during liver growth and under other circumstances^44^. In mammals, the onset and degree of polyploidization in hepatocytes vary across species and can be observed during normal or pathological conditions but the functional significance of the process remains uncertain^46^. To date, polyploidization events have not been reported for the liver of fish^45–47^, including salmonids^48^, irrespective of their ploidy status but information on Atlantic salmon is still lacking. In our study, we compared the nuclear size of hepatocytes in the liver of diploid and triploid salmon^13^ as indicator of their ploidy status. Cell size measurements (e.g. minor axis of erythrocytes) are frequently used as indirect measurements of DNA content in artificially induced triploids^28,49^. Overall, hepatocyte cell nuclei in triploids measured 1.2–1.4 times those of diploids and contained by definition correspondingly higher levels of DNA than diploid nuclei. Overall, these quantitative histological findings validate the use of liver as central organ regulating energy metabolism and several other functions to assess the possibility of dosage compensation in our study. This is in line with the work of Christensen et al.^50^ where liver tissue was chosen to measure gene expression levels in coho salmon under different metabolic states.

Triploid cell nuclei are expected to contain one extra haploid set of maternal chromosomes and therefore 50% more DNA than diploid cell nuclei with some degree of variation likely to occur across tissues within the same individual^49^. For example, variability in terms of nuclear differences between these two ploidies, possibly induced by specific cellular and nuclear adjustments, has been reported across tissues and organs of the marine medaka, *Oryzias dancena*^51^. In our work, cell measurements performed on histological sections showed that the nuclear size (minor and major axis) of triploid over diploid hepatocytes of juvenile Atlantic salmon was below the expected 1.5 ratio and further studies are required to explore the cellular processes occurring in the liver of these two ploidy groups.

Overall, our results indicate clear similarities in the general liver morphology and steatosis between diploid and triploid fish prior to and after smoltification. Quantification of liver steatosis based on the degree of vacuolization in the cytoplasm and the degree of distribution of the vacuolated hepatocytes has been employed in numerous fish studies addressing lipid metabolism. In Atlantic salmon, examples include nutritional and nutrigenomics studies performed to assess the effects of plant meal diets and natural plant extracts^52^, functional feeds^53,54^ and inclusion of micronutrients in low marine fish diets^16^ on fish growth, development and health. In the present work, the observed decrease in liver steatosis from fry to parr reflected the hepatic transcriptome profiles, which showed a significant up-regulation of genes involved in lipid metabolism in parr compared to fry, independently from their ploidy status. Such higher levels of relevant transcripts likely supported the mobilisation of energy required to reach this phase of development and in preparation of smoltification. These results are in agreement with Peruzzi et al.^13^, where the same experimental diploid and triploid fish fed a standard commercial fishmeal diet displayed a surge in somatic growth in the period from fry to parr. It is known that alterations in lipid metabolism during parr-smolt transformation in Atlantic salmon lead to smolt having reduced available liver, as well as muscle, energy reserves compared to parr and these are regarded as an integral part of the process^21,55,56^. In the present study, the lack of vacuolisation in the hepatocytes of both diploid and triploid smolts points indicates additional similarities between these two ploidy groups in the pattern of lipid mobilisation required during the final steps of parr-smolt transformation.

In Atlantic salmon, the liver transcriptome is different between fry, parr and smolts, as expected. The top differentially expressed genes between ontogeny stages are involved in preparation for smoltification. These include *aurkb*, *ima2*, *cpt1b*, *cpt1a*, *slc6a13*, *odc1*, *lipc*, *lipe*, *dcor1*, *pla2g3*, *ppp1r3b*, *ccl19*, *gnvip1*, *scyb7*, *ifi44*, *adh1*, which are involved in oxidative stress, metabolism, immune system and cellular processes. Most of these genes were common to both ploidy groups, except one gene that plays a role in oxidative stress (*adh1*) and two genes involved in cellular processes (*ima2* and *aurkb*), which were differentially regulated exclusively in triploids. At each ontogeny stage, the biological processes enriched for down-regulated genes showed a striking resemblance in diploid and triploid fish, which was reflected in a similar liver morphology and level of vacuolisation between ploidy groups.

## Materials and methods

### Experimental setup and fish husbandry

Details on the relationship, origin, production, growth parameters and rearing protocols of the experimental fish are provided by Peruzzi et al.^13^. Briefly, experimental fish comprising of diploid (2n) and triploid (3n) ova were produced using siblings composed of n = 17 full-sib and half families produced by a commercial hatchery (Stonfiskur HF, Iceland) where triploidy was induced by a pressure shock of 9500 psi applied for 5 min, 300° minutes post-fertilization at 5 °C^46^. Eyed-ova (~ 400 day-degrees) were then shipped to the Aquaculture Research Station in Kårvika (Tromsø, Norway). Prior to start-feeding, the ploidy status of the experimental groups was verified by flow cytometry as previously reported^13^. The experimental fish were reared in triplicate tanks and fed a commercial standard diet (Skretting AS, Stavanger, Norway) whilst being reared at low temperature from start-feeding to completion of the parr-smolt transformation. Constant light was used throughout the experiment, except for a period of reduced day length required to simulate winter conditions and induce parr-smolt transformation^13^. For sampling purposes, fish were euthanized using an anaesthetic overdose of benzocaine (120 mgL^-1^, Sigma-Aldrich Company Ltd., United Kingdom). Liver samples were collected from fish at the ontogeny stages below, snap-frozen in liquid nitrogen and transported on dry ice to Nord University (Bodø, Norway) where they were stored at − 80 ^o^C until transcriptome analysis. The average weight of fish at each stage used for this: fry (2n = 3.62 g and 3n = 4.11 g), parr (2n = 27.83 g and 3n = 29.89 g) and smolt (2n = 56.44 g and 3n = 66.56 g). Samples collected for all analyses had weights close to the mean weight.

### Preparation of RNA-seq libraries and sequencing

Total RNA was extracted from frozen liver samples following the QIAzol protocol (Qiagen, Germany) and further cleaned with the RNeasy MinElute cleanup kit (Qiagen, Germany). Quality and quantity of total RNA were determined using the 2200 TapeStation from Agilent (USA). Each RNA sample used had a RINe (RNA integrity) value above 7. RNA was extracted from 5 individual liver samples (n = 5) per ploidy group at selected sampling stages fry (1455 degree-days post-start feeding, ddPSF), parr (1888 ddPSF) and smolt (2745 ddPSF). RNA-seq libraries were prepared from total RNA using the NEBNext Ultra II Directional RNA Library Prep kit (NEB, USA). The barcoded libraries were checked for quality and quantity on a 2200 TapeStation before pooling and sequencing. Library pools were sequenced on the NextSeq500 (Illumina, USA) with the Nextseq 500/550 high output kit v2.5 sequencing kit (150 cycles single-end reads) at Nord University, Norway.

### Bioinformatics

Data were converted from BCL to FASTA format and demultiplexed using the Illumina script bcl2fastq conversion software v2.17 with default parameters. Adapters were then removed using the Cutadapt^57^ and quality of the trimmed reads was checked using the FastQC software^58^. Clean reads were mapped to the Atlantic salmon genome and transcriptome annotation databases (RefSeq accession: GCF_000233375.1 from the NCBI database) using TopHat2^59^. Uniquely mapped transcripts were quantified using HTSeq (high-throughput sequence) counts^60^ and DEGs were then identified using DESeq2.

(https://www.bioconductor.org/packages/release/bioc/html/DESeq2.html), with an adjusted *p*-value < 0.05 (Benjamin-Hochberg method)^61^. The R packages ggplot2 and heatmap were used for graphical representation of the data. DEGs were then subjected to GO enrichment analysis for biological processes using DAVID v6.8 with an EASE score ≤ 0.01^62,63^. Selected DEGs obtained from DESeq2 in Entrez gene identifier format were converted to FASTA format for downstream analysis on the Kyoto Encyclopaedia for Genes and Genomes (KEGG) Automatic Annotation Server (KAAS)^64^.

### Liver histology

Liver samples from different fish belonging to the same group used for transcriptomic analysis were fixed in 10% (v/v) neutral buffered formalin for 48 h before transferred to 70% (v/v) ethanol for storage. Samples were then transferred to standard cassettes, dehydrated, embedded in paraffin wax and sectioned at 5 μm on a RM2255 rotary microtome (Leica Microsystems, Germany). The sections were stained with haematoxylin/eosin (Thermoscientific, USA) according to standard histological procedures and examined on a DM 2000 LED light microscope (Leica Microsystems, Germany) equipped with a Leica DFC 295 digital Colour Camera. Photographs of the sections were processed using the Leica software application suite. A total of 41 individual samples were selected for this study: 10 fry (2n = 4 and 3n = 6), 15 (2n = 8 and 3n = 7) parr and 16 (3n and 2n = 8) smolts. Liver steatosis (lipid vacuolization) was assessed on images captured at magnification of 40 × and analysed with ImageJ following a modification of the method by Campos et al.^65^. Briefly, three sections per individual were scored for vesicular steatosis (clear vacuoles with a diameter greater than 5 μm) in hepatocytes using a set scale of 7 pixels/ μm. For each section, a 5,000 μm^2^ rectangular frame was placed over three hepatic regions distant from large vessels and the average area (μm^2^) occupied by lipid vacuoles within hepatocytes was calculated. Nuclear size (minor and major axis) of hepatocytes was assessed on the above-reported histological sections (ca. n = 10 nuclei/section) captured at a magnification of 40 × and analysed with ImageJ using a set scale of 1.0 μm.

### Statistical analysis

Liver steatosis data (i.e., vacuolization area) were log-transformed and checked for normality and equality of variance prior to two-way ANOVA with fish ploidy and ontogeny stage as fixed factors. When significant differences were found, the Hochberg’s post-hoc test was used to determine differences between individual fish groups. Hepatocytes’ nuclear size measurements in both ploidies and at different ontogeny stages were compared by Students’ t-test. Ratios of nuclear measurements in hepatocytes of triploid over diploid fish at different stages were analysed by one-way ANOVA with ontogeny stage as fixed factor. All data analyses were performed using SPSS (IBM SPSS statistics, USA) and the results were considered significant at P < 0.05. Data are reported as means ± standard error of the mean (SEM).

### Ethics statement

All experimental procedures involving Atlantic salmon were in accordance with the Norwegian legislation on animal experimentation and the guidelines of the European Union Council (Directive 2010/EU) and were approved by the Norwegian Committee on Ethics in Animal Experimentation (Project license permit ID: 8180) issued by the Norwegian Food Safety Authority (Mattilsynet).

## Supplementary information

Supplementary file 1

## Data Availability

Raw sequenced data has been submitted to the National Centre for Biotechnology Information (NCBI) under the BioSample accession: SAMN13315576, ID: 13315576.

## Abbreviations

DEGs: Differentially expressed genes
GO: Gene ontology
KEGG: Kyoto encyclopaedia of genes and genomes
PCA: Principal component analysis
