# Supplementary material for: Transcriptome sequencing and histology reveal dosage compensation in the liver of triploid pre-smolt Atlantic salmon
Source: Sci Rep. 2020 Oct 8;10:16836. doi: 10.1038/s41598-020-73814-6 (PMC7544907; doi:10.1038/s41598-020-73814-6)
Supplement: Supplementary file 1 [file 41598_2020_73814_MOESM1_ESM.pdf]

Supplementary Information

Transcriptome sequencing and histology reveal dosage compensation in the liver of triploid pre-smolt Atlantic salmon

Derrick K. Odei[1,2], Ørjan Hagen[1], Stefano Peruzzi[2], Inger-Britt Falk-Petersen[2], Jorge M.O. Fernandes[1*]

[1.] *Faculty of Biosciences and Aquaculture, Nord University, 8049 Bodø, Norway*

[2.] *Faculty of Biosciences, Fisheries and Economics, University of Tromsø- The Arctic University of Norway, 9035 Tromsø, Norway*

* Correspondence and requests for materials should be addressed to J.M.O.F. (email: jorge.m.fernandes@nord.no)

Index

Supplementary Figure S1	3
Supplementary Table S1	4
Supplementary Table S2	7
Supplementary Table S3	134
Supplementary Table S4	139
Supplementary Table S5	142
Supplementary Table S6	143
Supplementary Table S7	144
Supplementary Table S8	145

Supplementary Figure S1

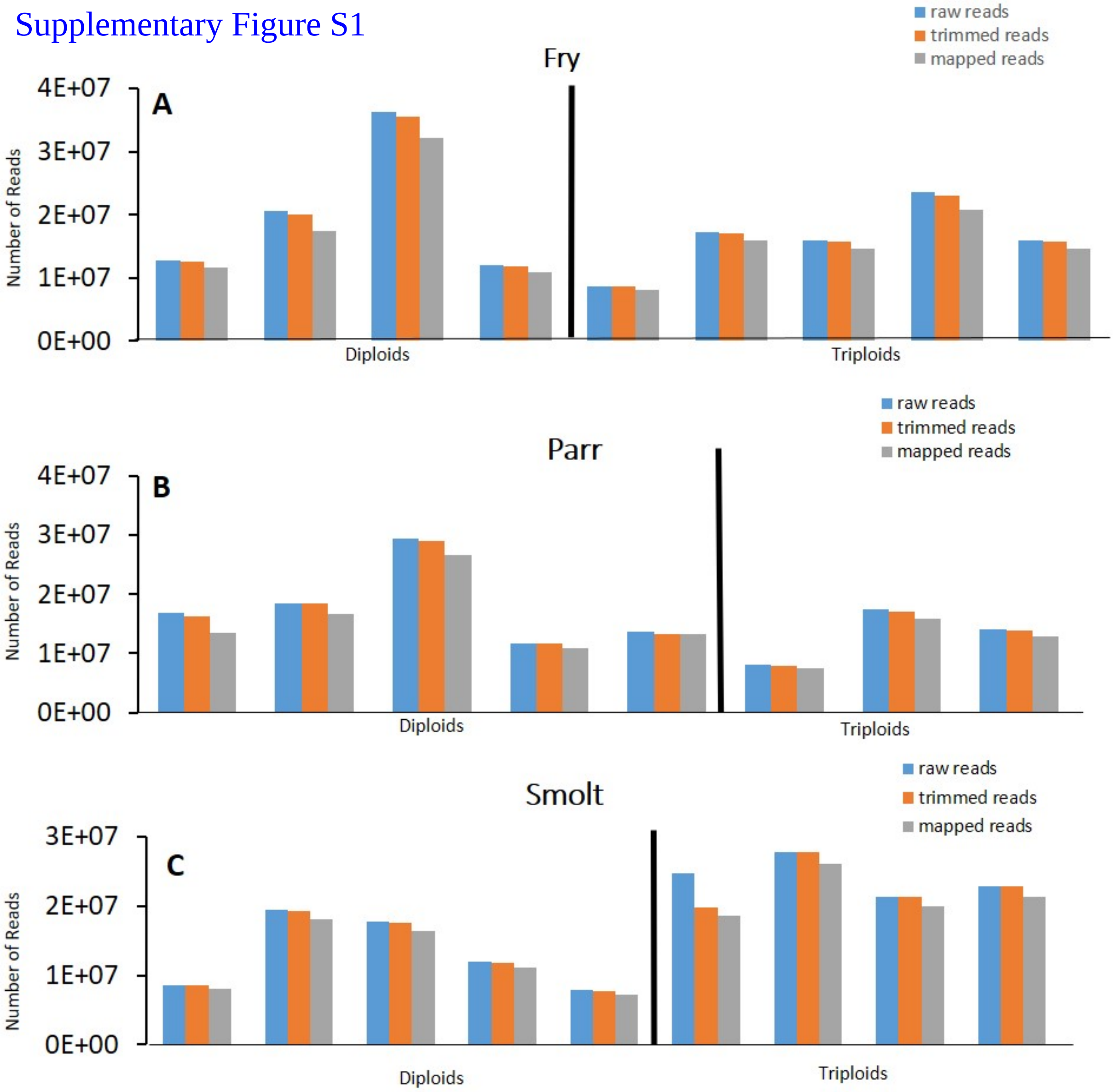

Supplementary Table S1

Ontogeny stage: fry

NCBI ID	Gene locus	Gene Name	baseMean	log2FoldChange	Fold change	pvalue	padj
**Up-regulated DEGs in diploids compared to triploids**							
**NCBI ID**	**Gene locus**	**Gene Name**	**baseMean**	**log2FoldChange**	**Fold change**	**pvalue**	**padj**
106570152	LOC106570152	Ig kappa chain C region-like	58.06	1.48	2.79	0	0
**Down-regulated in diploids compared to diploids**			**baseMean**	**log2FoldChange**	**Fold change**	**pvalue**	**padj**
106568688	LOC106568688	uncharacterized LOC106568688	28.12	-2.19	-4.58	0	0
106562616	LOC106562616	probable DNA polymerase	20.34	-1.84	-3.57	0	0
106577515	LOC106577515	uncharacterized LOC106577515	27.42	-1.75	-3.36	0	0
106605225	LOC106605225	cytochrome c oxidase subunit 7A-related protein, mitoch	82.36	-1.41	-2.66	0	0
106589046	LOC106589046	uncharacterized protein C6orf47-like	406.41	-1.13	-2.18	0	0
106567570	LOC106567570	HLA class II histocompatibility antigen, DQ beta 1 chain-li	41.93	-1.03	-2.04	0	0

Ontogeny stage: parr

Up-regulated DEGs in diploids compared to triploids							
**NCBI ID**			**baseMean**	**log2FoldChange**	**Fold change**	**pvalue**	**padj**
106603905	LOC106603905	uncharacterized LOC106603905	7.81	1.71	3.27	0	0
106585202	LOC106585202	succinyl-CoA:3-ketoacid coenzyme A transferase 1, mitoc	13.58	1.64	3.12	0	0
106613140	LOC106613140	sodium- and chloride-dependent GABA transporter 2-like	6.26	1.46	2.74	0	0
106569527	LOC106569527	uncharacterized LOC106569527	10.40	1.41	2.65	0	0
106565122	LOC106565122	G0/G1 switch protein 2-like	341.18	1.39	2.62	0	0
106590607	LOC106590607	uncharacterized LOC106590607	68.11	1.23	2.34	0	0
106588135	LOC106588135	chymotrypsin A-like	6.32	1.22	2.33	0	0
106588537	LOC106588537	C-C chemokine receptor type 7-like	40.64	1.20	2.29	0	0
106611874	LOC106611874	macrophage colony-stimulating factor 1 receptor 1-like	114.12	1.18	2.27	0	0
106612131	cystm1	cysteine rich transmembrane module containing 1	26.32	1.18	2.26	0	0
106569366	LOC106569366	complement factor B-like	15618.48	1.17	2.25	0	0
100195866	tryp	Trypsin	10.02	1.17	2.25	0	0
106610407	LOC106610407	up-regulator of cell proliferation-like	18.53	1.15	2.22	0	0
106607151	LOC106607151	interferon-induced very large GTPase 1-like	50.93	1.14	2.20	0	0
100137023	trp-ii	trypsin II	13.45	1.10	2.15	0	0
106605673	LOC106605673	B-cell antigen receptor complex-associated protein alpha	39.98	1.09	2.13	0	0
106560267	LOC106560267	ras-like protein family member 11B	40.23	1.00	2.00	0	0
**Down-regulated in diploids compared to diploids**			**baseMean**	**log2FoldChange**	**Fold change**	**pvalue**	**padj**
106565698	LOC106565698	uncharacterized LOC106565698	124.79	-2.30	-4.93	0	0
106613559	LOC106613559	PDZK1-interacting protein 1-like	49.99	-2.02	-4.06	0	0
106613443	adgrl2	adhesion G protein-coupled receptor L2	23.24	-1.58	-2.98	0	0
100194692	LOC100194692	Krueppel-like factor 11	183.66	-1.20	-2.29	0	0
106567189	LOC106567189	Krueppel-like factor 11	168.41	-1.17	-2.25	0	0
106570846	naaladl1	N-acetylated alpha-linked acidic dipeptidase like 1	21.48	-1.11	-2.16	0	0
100196094	LOC100196094	dipeptidyl peptidase 1	328.45	-1.08	-2.11	0	0
106583167	LOC106583167	class E basic helix-loop-helix protein 40-like	462.49	-1.03	-2.04	0	0

Ontogeny stage: smolt

Down-regulated DEGs in diploids compared to triploids

NCBI ID			baseMean	log2FoldChange	Fold change	pvalue	padj
106565579	LOC106565579	uncharacterized	30.59	-1.51	-2.85	0	0
106576030	LOC106576030	uncharacterized protein LOC106576030 isoform X1	82.01	-1.34	-2.54	0	0

Supplementary Table S2a

Ploidy group: Diploid

Up-regulated DEGs in fry compared to parr

NCBI ID	Gene IDs/Locus	Gene name	baseMean	log2FoldChange	Fold change	pvalue	padj
106561238	cpt1b	carnitine palmitoyltransferase 1B	8.37	3.12	8.71	0.00	0.00
106564531	LOC106564531	GATA zinc finger domain-containing protein 10-like	168.68	3.08	8.47	0.00	0.00
106573415	LOC106573415	carnitine O-palmitoyltransferase 1, liver isoform-like	295.38	2.97	7.84	0.00	0.00
106599151	LOC106599151	acyl-coenzyme A thioesterase 11-like	71.33	2.97	7.83	0.01	0.04
106583167	LOC106583167	class E basic helix-loop-helix protein 40-like	404.11	2.95	7.75	0.00	0.00
106571795	LOC106571795	uncharacterized LOC106571795	15.15	2.92	7.58	0.00	0.02
106612462	LOC106612462	uncharacterized LOC106612462	78.45	2.91	7.54	0.00	0.01
106585649	LOC106585649	complement C1q-like protein 2	697.88	2.87	7.30	0.00	0.02
106574341	LOC106574341	insulin receptor substrate 2-like	148.53	2.85	7.22	0.00	0.00
106562007	LOC106562007	BCL2/adenovirus E1B 19 kDa protein-interacting protein 3-like	615.38	2.81	7.01	0.00	0.00
106587817	LOC106587817	hyaluronan and proteoglycan link protein 3-like	14.26	2.80	6.96	0.00	0.00
106583974	LOC106583974	beta-1,3-galactosyltransferase 2-like	9.59	2.78	6.86	0.00	0.00
106565611	LOC106565611	probable G-protein coupled receptor	9.45	2.70	6.50	0.00	0.00
106565117	LOC106565117	differentially expressed in FDCP 6 homolog	35.23	2.70	6.49	0.01	0.03
100195257	ugpa	UTP--glucose-1-phosphate uridylyltransferase	387.97	2.69	6.44	0.00	0.00
106584294	LOC106584294	uncharacterized LOC106584294	145.04	2.63	6.19	0.00	0.02
106609060	LOC106609060	rho GTPase-activating protein 6-like	45.65	2.63	6.18	0.00	0.00
106570607	LOC106570607	E3 ubiquitin-protein ligase ZNRF2-like	19.54	2.61	6.09	0.00	0.00
106602798	slamf7	NA	4.77	2.55	5.84	0.00	0.00
100195724	sh21a	SH2 domain-containing protein 1A	14.40	2.53	5.76	0.00	0.00
106566619	LOC106566619	interleukin-17 receptor E-like	35.14	2.51	5.69	0.00	0.01
106568147	LOC106568147	dual specificity protein phosphatase 4-like	16.13	2.49	5.60	0.00	0.00
106586898	LOC106586898	uncharacterized LOC106586898	5.13	2.48	5.57	0.00	0.01
106603573	slc13a5	NA	290.15	2.47	5.55	0.00	0.00
106603974	LOC106603974	uncharacterized LOC106603974	522.74	2.43	5.37	0.00	0.00
106588784	snrk	NA	24.55	2.42	5.35	0.00	0.00
106608166	LOC106608166	GTPase IMAP family member 7-like	10.06	2.41	5.30	0.00	0.02
106602059	LOC106602059	inward rectifier potassium channel 2-like	4.37	2.39	5.25	0.00	0.00
106576030	LOC106576030	uncharacterized LOC106576030	62.68	2.37	5.18	0.00	0.00
106587141	LOC106587141	adhesion G-protein coupled receptor G5-like	15.54	2.36	5.13	0.00	0.00
106613433	LOC106613433	GTPase IMAP family member 4-like	24.52	2.35	5.11	0.00	0.00
106568683	LOC106568683	inhibin beta A chain-like	73.72	2.34	5.07	0.00	0.00
106612301	LOC106612301	uncharacterized LOC106612301	7.23	2.34	5.07	0.00	0.00
106567901	LOC106567901	solute carrier family 13 member 5-like	54.69	2.33	5.03	0.00	0.00
106585608	LOC106585608	vinexin-like	45.53	2.32	4.99	0.00	0.01
106584369	LOC106584369	tyrosine-protein kinase ZAP-70-like	26.72	2.32	4.98	0.00	0.00
100169854	LOC100169854	uncharacterized LOC100169854	21.35	2.31	4.95	0.00	0.00
106600509	LOC106600509	alcohol dehydrogenase 1-like	2917.07	2.29	4.89	0.00	0.00
106579828	LOC106579828	uncharacterized LOC106579828	22.78	2.28	4.86	0.00	0.01
106573520	LOC106573520	zona pellucida sperm-binding protein 4-like	2071.75	2.27	4.83	0.00	0.00
100380534	bat1	HLA-B associated transcript 1	23.21	2.25	4.77	0.00	0.00
106574728	LOC106574728	uncharacterized LOC106574728	7.89	2.25	4.75	0.00	0.00
106602253	cdkl1	cyclin dependent kinase like 1	20.58	2.25	4.74	0.00	0.00
106585857	LOC106585857	arrestin domain-containing protein 3-like	1111.31	2.24	4.72	0.00	0.02
106606102	LOC106606102	protein FAM186A-like	10.86	2.21	4.64	0.00	0.00
106566824	LOC106566824	protein-L-isoaspartate O-methyltransferase domain-containing pr	8.12	2.21	4.62	0.00	0.01
106611254	LOC106611254	B-cell lymphoma/leukemia 11B-like	14.70	2.20	4.60	0.01	0.04
100194912	angl4	Angiopoietin-related protein 4	415.97	2.16	4.48	0.01	0.03

106582130	LOC106582130	insulin receptor substrate 2-like	6.01	2.15	4.44	0.00	0.00
106565647	LOC106565647	von Willebrand factor A domain-containing protein 1-like	10.57	2.15	4.42	0.00	0.00
106577998	LOC106577998	protein NDRG2-like	1378.00	2.14	4.41	0.00	0.00
106585060	LOC106585060	B-cell linker protein-like	46.31	2.12	4.36	0.00	0.00
106611988	LOC106611988	LON peptidase N-terminal domain and RING finger protein 3-like	74.44	2.11	4.33	0.00	0.00
106578866	LOC106578866	forkhead box protein K2-like	441.47	2.11	4.32	0.00	0.00
106583358	LOC106583358	isocitrate dehydrogenase [NAD] subunit beta, mitochondrial-like	45.63	2.11	4.31	0.00	0.00
106589061	LOC106589061	transcription factor SOX-4-like	204.23	2.11	4.31	0.00	0.01
106607817	heca	hdc homolog, cell cycle regulator	13.11	2.10	4.30	0.00	0.00
100195250	typh	Thymidine phosphorylase	161.03	2.09	4.27	0.00	0.00
106583965	LOC106583965	uncharacterized LOC106583965	10.84	2.09	4.27	0.00	0.00
106606554	LOC106606554	poly [ADP-ribose] polymerase 14-like	20.15	2.09	4.27	0.00	0.00
106569984	LOC106569984	C-type lectin domain family 5 member A-like	137.38	2.08	4.22	0.00	0.00
106584938	LOC106584938	cytochrome P450 2J2-like	6.03	2.07	4.19	0.00	0.00
106562261	LOC106562261	cytochrome P450 2C8-like	19.33	2.06	4.17	0.00	0.00
100136518	igfbp-1b1	IGF binding protein 1	250.40	2.05	4.15	0.00	0.00
106570152	LOC106570152	Ig kappa chain C region-like	45.93	2.05	4.15	0.00	0.00
100136448	LOC100136448	C type lectin receptor C	3384.39	2.05	4.14	0.00	0.01
106606746	LOC106606746	interferon-induced protein 44-like	34.46	2.05	4.14	0.00	0.00
106577019	neurl1	neuralized E3 ubiquitin protein ligase 1	8.68	2.04	4.11	0.00	0.00
106610162	LOC106610162	ankyrin repeat and SOCS box protein 5-like	7.62	2.04	4.11	0.00	0.00
106573147	LOC106573147	histidine ammonia-lyase-like	9.39	2.04	4.11	0.00	0.00
106565178	LOC106565178	interferon-induced protein 44-like	92.41	2.04	4.10	0.01	0.04
106613930	LOC106613930	calcium-activated potassium channel subunit beta-2-like	211.56	2.03	4.10	0.00	0.00
106572213	LOC106572213	ERBB receptor feedback inhibitor 1-like	22.42	2.03	4.09	0.00	0.00
106568165	LOC106568165	interleukin-7 receptor subunit alpha-like	19.14	2.03	4.09	0.00	0.00
106613694	LOC106613694	lipoprotein lipase-like	64.88	2.03	4.09	0.01	0.04
106567759	LOC106567759	uncharacterized LOC106567759	78.53	2.03	4.07	0.00	0.00
106575214	LOC106575214	signal transducer and activator of transcription 1-alpha/beta-like	8.65	2.01	4.04	0.00	0.00
106584838	neil1	nei like DNA glycosylase 1	128.18	2.01	4.04	0.00	0.00
106569872	LOC106569872	semaphorin-4A-like	47.74	2.01	4.04	0.00	0.00
106608136	LOC106608136	heat shock protein HSP 90-alpha-like	10.16	2.00	4.01	0.00	0.01
101448065	igfbp-1b2	insulin-like growth factor binding protein 1 paralog B2	98.89	2.00	4.01	0.00	0.00
106560822	LOC106560822	BCL2/adenovirus E1B 19 kDa protein-interacting protein 3-like	20.03	2.00	4.00	0.00	0.00
106561596	LOC106561596	sodium-dependent neutral amino acid transporter B(0)AT2-like	13.60	1.99	3.98	0.00	0.00
106576791	LOC106576791	coiled-coil and C2 domain-containing protein 2A-like	9.43	1.99	3.97	0.00	0.02
106580167	LOC106580167	mothers against decapentaplegic homolog 4-like	3.70	1.99	3.96	0.01	0.03
106589359	LOC106589359	prostaglandin E2 receptor EP1 subtype-like	14.87	1.98	3.95	0.00	0.00
100380427	errfi	ERBB receptor feedback inhibitor 1	499.21	1.98	3.94	0.00	0.00
101448021	gbp	GSK-3-binding protein	262.78	1.98	3.94	0.00	0.00
106603875	LOC106603875	CREB3 regulatory factor-like	174.09	1.98	3.94	0.00	0.01
106567542	LOC106567542	vasodilator-stimulated phosphoprotein-like	18.04	1.98	3.94	0.00	0.00
106573414	LOC106573414	kelch repeat and BTB domain-containing protein 13-like	11.08	1.97	3.93	0.01	0.03
106613754	LOC106613754	mast cell protease 1A-like	5.67	1.97	3.92	0.00	0.00
106560619	LOC106560619	basic salivary proline-rich protein 4-like	8.40	1.97	3.91	0.00	0.00
106589283	LOC106589283	src kinase-associated phosphoprotein 1-like	5.67	1.96	3.90	0.00	0.00
106612115	LOC106612115	SH2 domain-containing protein 4A-like	35.08	1.96	3.88	0.00	0.00
106584368	LOC106584368	MAP kinase-interacting serine/threonine-protein kinase 2-like	112.00	1.95	3.88	0.00	0.00
106613733	LOC106613733	mitochondrial coenzyme A transporter SLC25A42-like	17.03	1.95	3.87	0.00	0.00
100306825	i17ra	Interleukin-17 receptor A	12.72	1.95	3.86	0.00	0.02
106582601	LOC106582601	pleckstrin homology-like domain family B member 3	26.73	1.95	3.86	0.01	0.04

106578437	LOC106578437	semaphorin-5A-like	20.73	1.95	3.86	0.00	0.00
106562131	LOC106562131	UDP-glucuronosyltransferase 2A1 pseudogene	41.05	1.94	3.83	0.00	0.00
106611919	LOC106611919	oxidoreductase-like domain-containing protein 1	7.26	1.94	3.83	0.00	0.00
106613496	LOC106613496	ubiquitin carboxyl-terminal hydrolase 13-like	19.55	1.94	3.83	0.00	0.00
106614017	LOC106614017	BCL2/adenovirus E1B 19 kDa protein-interacting protein 3-like	95.61	1.94	3.83	0.00	0.00
106577467	LOC106577467	procollagen C-endopeptidase enhancer 2-like	39.86	1.94	3.83	0.00	0.00
106576229	LOC106576229	ankyrin repeat and SOCS box protein 13-like	12.18	1.93	3.82	0.00	0.00
106565509	LOC106565509	uncharacterized LOC106565509	3.98	1.93	3.82	0.01	0.04
106611907	LOC106611907	G-protein coupled receptor family C group 5 member C-like	218.88	1.93	3.82	0.00	0.00
106605167	LOC106605167	pleckstrin homology-like domain family B member 3	62.87	1.93	3.82	0.00	0.00
106560370	LOC106560370	interferon regulatory factor 4-like	4.69	1.93	3.81	0.00	0.01
106562736	LOC106562736	b(0,+)-type amino acid transporter 1-like	15.42	1.93	3.81	0.00	0.00
106586875	LOC106586875	uncharacterized LOC106586875	13.67	1.93	3.80	0.00	0.02
106586891	LOC106586891	interferon-induced GTP-binding protein Mx2-like	10.09	1.92	3.79	0.00	0.00
106565146	LOC106565146	uncharacterized LOC106565146	22.17	1.92	3.79	0.00	0.00
106602655	LOC106602655	SLAM family member 5-like	12.47	1.92	3.78	0.00	0.00
106589372	lgi1	leucine rich glioma inactivated 1	49.54	1.92	3.78	0.00	0.00
106600841	LOC106600841	nuclear factor erythroid 2-related factor 1-like	92.24	1.91	3.76	0.00	0.00
106583020	LOC106583020	receptor-type tyrosine-protein phosphatase gamma-like	131.54	1.91	3.76	0.00	0.00
106585134	LOC106585134	serine protease HTRA1-like	166.17	1.91	3.75	0.01	0.05
106583905	LOC106583905	protein yippee-like 3	178.17	1.91	3.75	0.00	0.00
106564975	LOC106564975	integrin alpha-X-like	37.54	1.90	3.73	0.00	0.00
106612757	LOC106612757	inverted formin-2-like	4.50	1.90	3.73	0.00	0.00
106567189	LOC106567189	Krueppel-like factor 11	166.74	1.90	3.72	0.00	0.00
106570861	LOC106570861	C-C motif chemokine 20-like	28.38	1.89	3.72	0.01	0.03
106604198	LOC106604198	uncharacterized LOC106604198	8.78	1.89	3.71	0.00	0.00
106578860	LOC106578860	protein shisa-6 homolog	21.76	1.89	3.70	0.01	0.04
106611681	LOC106611681	mucin-22-like	11.99	1.89	3.69	0.00	0.01
106582291	LOC106582291	sclerostin domain-containing protein 1-like	13.19	1.88	3.69	0.00	0.00
106601791	LOC106601791	Na(+)/H(+) exchange regulatory cofactor NHE-RF2-like	2.84	1.88	3.68	0.00	0.00
106604964	LOC106604964	uncharacterized LOC106604964	3.81	1.88	3.67	0.00	0.00
106610455	LOC106610455	GTPase IMAP family member 4-like	25.14	1.87	3.67	0.00	0.02
106573445	LOC106573445	uncharacterized LOC106573445	114.54	1.87	3.66	0.00	0.00
106583939	LOC106583939	nuclear protein 1-like	2653.56	1.87	3.66	0.00	0.00
106580723	LOC106580723	tristetraprolin-like	374.26	1.87	3.66	0.00	0.01
106613130	LOC106613130	solute carrier family 25 member 36-A-like	174.29	1.87	3.65	0.00	0.00
106576895	cssa18h10orf54	chromosome ssa18 open reading frame, human C10orf54	31.90	1.87	3.65	0.00	0.01
106588908	LOC106588908	zinc finger FYVE domain-containing protein 1-like	113.03	1.87	3.65	0.00	0.02
106598853	LOC106598853	probable cation-transporting ATPase 13A3	38.34	1.86	3.64	0.01	0.04
106576235	LOC106576235	ADM2-like	79.04	1.86	3.63	0.00	0.00
106599056	LOC106599056	uncharacterized LOC106599056	3.53	1.86	3.63	0.00	0.00
106569814	LOC106569814	guanine nucleotide exchange factor VAV3-like	14.07	1.86	3.62	0.00	0.02
106565076	LOC106565076	1,25-dihydroxyvitamin D(3) 24-hydroxylase, mitochondrial-like	92.21	1.86	3.62	0.00	0.00
106610314	LOC106610314	hydroperoxide isomerase ALOXE3-like	3.74	1.85	3.61	0.01	0.03
106568567	LOC106568567	UDP-glucuronosyltransferase 2A1-like	125.67	1.85	3.60	0.00	0.00
106561703	hbp1	HMG-box transcription factor 1	167.05	1.85	3.60	0.01	0.03
100195689	mog2a	2-acylglycerol O-acyltransferase 2-A	103.89	1.85	3.60	0.00	0.01
106576058	strip2	NA	110.77	1.85	3.60	0.00	0.00
106574392	LOC106574392	ladderlectin-like	3352.50	1.84	3.58	0.00	0.00
106608992	LOC106608992	purine nucleoside phosphorylase-like	1370.16	1.84	3.58	0.00	0.00
106570908	LOC106570908	uncharacterized LOC106570908	3.10	1.84	3.58	0.00	0.00

106581032	LOC106581032	sterile alpha and TIR motif-containing protein 1-like	21.48	1.84	3.58	0.00	0.00
106575805	LOC106575805	ETS translocation variant 5-like	7.58	1.84	3.57	0.00	0.00
106612062	LOC106612062	lymphocyte cytosolic protein 2-like	12.19	1.83	3.57	0.00	0.02
106611497	LOC106611497	ETS-related transcription factor Elf-4-like	5.34	1.83	3.56	0.00	0.00
106565283	LOC106565283	beta-1,3-galactosyl-O-glycosyl-glycoprotein beta-1,6-N-acetylgluc	14.11	1.83	3.55	0.01	0.03
106606181	LOC106606181	protein Jumonji pseudogene	47.15	1.83	3.54	0.00	0.00
106610796	LOC106610796	adenylyltransferase and sulfurtransferase MOCS3-like	6.23	1.82	3.54	0.01	0.04
100194692	LOC100194692	Krueppel-like factor 11	183.96	1.82	3.54	0.01	0.03
106612469	LOC106612469	nucleolar RNA helicase 2-like	787.28	1.82	3.54	0.00	0.00
106609917	LOC106609917	uncharacterized LOC106609917	6.53	1.82	3.53	0.00	0.01
106576186	LOC106576186	hemagglutinin/amebocyte aggregation factor-like	207.91	1.82	3.52	0.00	0.00
106586789	LOC106586789	probable G-protein coupled receptor 82	4.96	1.82	3.52	0.01	0.03
106563738	LOC106563738	T-cell surface antigen CD2-like	5.61	1.81	3.52	0.00	0.00
106578216	LOC106578216	tetratricopeptide repeat protein 39B-like	3.18	1.81	3.50	0.00	0.01
106577374	LOC106577374	BCL2/adenovirus E1B 19 kDa protein-interacting protein 3-like	90.31	1.81	3.50	0.00	0.00
106563917	LOC106563917	T-cell surface protein tactile-like	27.24	1.80	3.48	0.00	0.00
106603553	LOC106603553	SAM domain-containing protein SAMSN-1-like	28.40	1.80	3.47	0.00	0.00
100196655	hsp70-3	heat shock protein 70	97.70	1.79	3.46	0.00	0.02
106584062	LOC106584062	cytochrome P450 2J2-like	782.25	1.79	3.46	0.00	0.02
106588841	LOC106588841	lysosomal alpha-glucosidase-like	13.14	1.79	3.46	0.00	0.01
106569936	LOC106569936	H-2 class I histocompatibility antigen, Q10 alpha chain-like	73.24	1.78	3.44	0.00	0.00
106601045	LOC106601045	histone-lysine N-methyltransferase EZH1-like	42.13	1.78	3.44	0.00	0.00
106567218	LOC106567218	tumor protein D54-like	27.01	1.78	3.44	0.00	0.00
106606639	LOC106606639	uncharacterized LOC106606639	8.40	1.78	3.43	0.01	0.04
106571707	LOC106571707	uncharacterized LOC106571707	12.73	1.78	3.42	0.00	0.02
106561069	LOC106561069	protein FAM60A-like	22.03	1.78	3.42	0.00	0.01
106564787	LOC106564787	heterogeneous nuclear ribonucleoprotein L-like	13.22	1.77	3.42	0.00	0.02
106564909	LOC106564909	uncharacterized LOC106564909	8.06	1.77	3.42	0.00	0.00
106613559	LOC106613559	PDZK1-interacting protein 1-like	57.07	1.77	3.42	0.00	0.00
106609985	LOC106609985	uncharacterized LOC106609985	72.01	1.77	3.41	0.00	0.00
106563276	LOC106563276	HAUS augmin-like complex subunit 4	59.74	1.77	3.41	0.01	0.03
106567124	LOC106567124	ankyrin repeat domain-containing protein SOWAHB-like	59.20	1.77	3.41	0.00	0.00
106605290	LOC106605290	myotubularin-related protein 11-like	52.94	1.77	3.40	0.01	0.05
106590089	LOC106590089	protein phosphatase 1 regulatory subunit 3G-like	403.78	1.77	3.40	0.00	0.00
106607477	LOC106607477	pyruvate dehydrogenase (acetyl-transferring) kinase isozyme 2, m	343.82	1.76	3.39	0.00	0.00
100380611	arc1b	Actin-related protein 2/3 complex subunit 1B	30.50	1.76	3.39	0.00	0.00
106610386	LOC106610386	adapter protein CIKS-like	41.58	1.76	3.39	0.00	0.01
106607748	LOC106607748	low-density lipoprotein receptor-related protein 11-like	69.91	1.76	3.38	0.00	0.00
106572227	LOC106572227	protein-L-isoaspartate O-methyltransferase domain-containing pr	6.89	1.76	3.38	0.00	0.00
106569132	ccdc178	coiled-coil domain containing 178	11.96	1.75	3.37	0.00	0.00
106566156	LOC106566156	uncharacterized LOC106566156	4.04	1.75	3.37	0.00	0.00
106568282	LOC106568282	WD repeat domain phosphoinositide-interacting protein 4-like	3.50	1.75	3.36	0.00	0.00
106585107	LOC106585107	rho GTPase-activating protein 25-like	33.78	1.75	3.36	0.01	0.04
106562802	LOC106562802	multidrug resistance-associated protein 9-like	10.94	1.75	3.35	0.00	0.00
106590522	LOC106590522	probable cationic amino acid transporter	16.66	1.74	3.35	0.00	0.01
106581655	LOC106581655	P2Y purinoceptor 8-like	8.58	1.74	3.35	0.00	0.00
106611259	LOC106611259	uncharacterized LOC106611259	2.16	1.74	3.34	0.00	0.00
106602122	LOC106602122	uncharacterized LOC106602122	5.89	1.74	3.34	0.00	0.02
106567020	LOC106567020	serine/threonine-protein kinase WNK2-like	160.59	1.74	3.34	0.00	0.02
106562180	LOC106562180	uncharacterized LOC106562180	7.74	1.74	3.33	0.00	0.00
106605619	LOC106605619	uncharacterized LOC106605619	11.89	1.74	3.33	0.00	0.00

106561469	LOC106561469	E3 ubiquitin/ISG15 ligase TRIM25-like	3.49	1.74	3.33	0.00	0.00
106577579	LOC106577579	protein NLRC3-like	84.72	1.74	3.33	0.00	0.00
106606005	LOC106606005	peroxisomal carnitine O-octanoyltransferase-like	42.14	1.74	3.33	0.00	0.01
106603574	LOC106603574	uncharacterized LOC106603574	5.11	1.73	3.32	0.00	0.00
106609354	LOC106609354	ankyrin repeat and SOCS box protein 13-like	27.37	1.73	3.32	0.00	0.00
106611569	LOC106611569	probable carboxypeptidase X1	39.76	1.73	3.32	0.00	0.00
106567018	LOC106567018	SLIT-ROBO Rho GTPase-activating protein 3-like	15.37	1.73	3.31	0.00	0.00
100136926	cyp1a	cytochrome P450 1A	3653.66	1.73	3.31	0.00	0.00
106572192	LOC106572192	intelectin	201.74	1.72	3.30	0.00	0.00
106578355	LOC106578355	zinc finger protein 281-like	6.13	1.72	3.30	0.00	0.00
106600931	LOC106600931	nuclear receptor subfamily 5 group A member 2-like	399.87	1.72	3.30	0.00	0.00
106561471	LOC106561471	tripartite motif-containing protein 16-like	3.24	1.72	3.30	0.00	0.00
106608133	LOC106608133	type III iodothyronine deiodinase-like	12.00	1.72	3.29	0.00	0.00
106561275	LOC106561275	uncharacterized LOC106561275	284.00	1.72	3.28	0.00	0.01
106571057	LOC106571057	interleukin-17F-like	2.10	1.71	3.28	0.00	0.00
106577198	LOC106577198	catechol O-methyltransferase domain-containing protein 1-like	137.37	1.71	3.27	0.00	0.00
106607198	LOC106607198	UPF0515 protein C19orf66 homolog	50.84	1.71	3.27	0.00	0.01
106582117	lrrc58	leucine rich repeat containing 58	147.31	1.71	3.26	0.00	0.01
106562986	LOC106562986	electrogenic sodium bicarbonate cotransporter 4-like	13.75	1.70	3.24	0.00	0.02
106562353	LOC106562353	uncharacterized LOC106562353	18.62	1.70	3.24	0.01	0.04
106604273	LOC106604273	rho guanine nucleotide exchange factor 9-like	6.77	1.69	3.23	0.00	0.00
106566127	LOC106566127	inhibin beta B chain-like	150.03	1.69	3.23	0.01	0.02
106600728	LOC106600728	protein yippee-like 3	26.33	1.69	3.22	0.00	0.00
106586229	LOC106586229	integrin alpha-6-like	248.57	1.69	3.22	0.00	0.00
100196604	apof	apolipoprotein F	601.97	1.68	3.21	0.00	0.00
106602597	LOC106602597	uncharacterized LOC106602597	70.29	1.68	3.21	0.00	0.00
106603300	LOC106603300	E3 ubiquitin-protein ligase RNF128-like	7.32	1.68	3.21	0.00	0.00
106568443	LOC106568443	RNA 3'-terminal phosphate cyclase-like protein	40.96	1.68	3.20	0.00	0.00
106577783	LOC106577783	putative E3 ubiquitin-protein ligase ARI6	8.52	1.67	3.19	0.00	0.00
106609586	LOC106609586	T-complex protein 11-like protein 2	108.79	1.67	3.19	0.00	0.00
106611011	LOC106611011	uncharacterized LOC106611011	511.33	1.67	3.18	0.00	0.01
106576682	LOC106576682	cGMP-dependent protein kinase 2-like	1.97	1.67	3.18	0.00	0.00
106567827	LOC106567827	zinc finger and SCAN domain-containing protein 21-like	3.34	1.67	3.18	0.00	0.01
106579132	LOC106579132	zinc finger protein 239-like	17.92	1.67	3.18	0.00	0.00
106612143	LOC106612143	syntaxin-binding protein 4-like	37.95	1.67	3.17	0.00	0.00
106585105	LOC106585105	max dimerization protein 1-like	123.39	1.67	3.17	0.00	0.01
106566870	LOC106566870	rho guanine nucleotide exchange factor 19-like	23.21	1.66	3.17	0.00	0.00
106577640	LOC106577640	uncharacterized LOC106577640	93.04	1.66	3.17	0.00	0.00
106602156	LOC106602156	uncharacterized LOC106602156	14.27	1.66	3.16	0.00	0.00
106579130	LOC106579130	myosin light chain kinase, smooth muscle-like	3.65	1.66	3.15	0.00	0.00
106571437	LOC106571437	uncharacterized LOC106571437	10.04	1.66	3.15	0.01	0.04
106609013	LOC106609013	natural killer cell receptor 2B4-like	19.31	1.66	3.15	0.00	0.01
100286735	pss	CDP-diacylglycerol--serine O-phosphatidyltransferase	5.98	1.65	3.15	0.00	0.00
106605368	LOC106605368	T-cell differentiation antigen CD6-like	4.56	1.65	3.15	0.00	0.00
106604670	LOC106604670	trichohyalin-like	27.24	1.65	3.15	0.00	0.00
106570575	LOC106570575	ataxin-1-like	10.32	1.65	3.14	0.00	0.00
106564683	LOC106564683	intraflagellar transport protein 56	14.13	1.65	3.14	0.00	0.01
106612812	LOC106612812	P2X purinoceptor 5-like	3.21	1.65	3.14	0.00	0.00
106583431	LOC106583431	interferon-induced protein 44-like	140.34	1.65	3.14	0.00	0.01
100380645	LOC100380645	nuclear receptor subfamily 2 group F member 5	120.12	1.65	3.13	0.00	0.00
106589295	LOC106589295	neuronal-specific septin-3-like	9.38	1.65	3.13	0.00	0.02

106613765	LOC106613765	cyclic AMP-responsive element-binding protein 3-like protein 3-B	903.01	1.64	3.12	0.00	0.00
106561913	epb41l4a	erythrocyte membrane protein band 4.1 like 4A	67.67	1.64	3.12	0.00	0.02
106566022	LOC106566022	synaptotagmin-2-like	4.99	1.64	3.11	0.01	0.04
106605130	LOC106605130	mitogen-activated protein kinase kinase kinase 5-like	111.93	1.64	3.11	0.00	0.00
106569509	LOC106569509	uncharacterized LOC106569509	9.11	1.64	3.11	0.00	0.01
106571912	LOC106571912	zinc finger CCCH domain-containing protein 18-like	5.14	1.63	3.10	0.00	0.01
106576376	LOC106576376	differentially expressed in FDCP 6-like	81.01	1.63	3.10	0.01	0.03
106608426	LOC106608426	tyrosine-protein kinase TXK-like	3.27	1.63	3.09	0.00	0.00
106589038	LOC106589038	unconventional myosin-Ig-like	4.20	1.63	3.09	0.00	0.00
106570173	LOC106570173	uncharacterized LOC106570173	26.64	1.63	3.09	0.00	0.00
106608841	LOC106608841	probable E3 ubiquitin-protein ligase RNF144A-A	8.77	1.63	3.09	0.00	0.00
106572338	LOC106572338	ras-related protein Rab-27B-like	4.78	1.63	3.08	0.00	0.01
106586664	LOC106586664	insulin receptor substrate 2-like	211.10	1.62	3.08	0.00	0.00
106567883	LOC106567883	uncharacterized LOC106567883	27.29	1.62	3.08	0.00	0.00
106564314	LOC106564314	transmembrane channel-like protein 8	80.18	1.62	3.08	0.00	0.02
106575482	LOC106575482	glutamate receptor ionotropic, NMDA 3B-like	5.60	1.62	3.08	0.00	0.01
106613975	LOC106613975	ABC transporter G family member 20-like	2.71	1.62	3.07	0.00	0.00
106560337	ptprf	NA	5.83	1.62	3.07	0.00	0.02
106604927	LOC106604927	Down syndrome cell adhesion molecule-like	65.16	1.62	3.07	0.00	0.00
106589069	LOC106589069	uncharacterized LOC106589069	4.19	1.62	3.07	0.00	0.00
106601629	LOC106601629	uncharacterized LOC106601629	5.45	1.62	3.06	0.01	0.04
106569947	LOC106569947	major histocompatibility complex class I-related gene protein-like	778.64	1.62	3.06	0.00	0.00
106604826	LOC106604826	dedicator of cytokinesis protein 2-like	45.33	1.61	3.06	0.00	0.00
106585022	LOC106585022	serine incorporator 5-like	20.00	1.61	3.05	0.00	0.00
106564916	LOC106564916	tripartite motif-containing protein 35-like	2.80	1.61	3.05	0.01	0.05
106613695	LOC106613695	lipoprotein lipase-like	1152.56	1.61	3.05	0.00	0.00
106599613	kiaa0040	KIAA0040 ortholog	4.62	1.61	3.04	0.00	0.01
106565145	LOC106565145	uncharacterized LOC106565145	25.78	1.61	3.04	0.00	0.01
100306869	can3	Calpain-3	3.79	1.61	3.04	0.00	0.01
106610004	LOC106610004	uncharacterized LOC106610004	5.73	1.60	3.04	0.00	0.00
106606426	LOC106606426	transcription factor Sox-9-A-like	32.20	1.60	3.04	0.00	0.00
106588934	LOC106588934	pyruvate dehydrogenase (acetyl-transferring) kinase isozyme 2, m	14.96	1.60	3.03	0.00	0.00
106605575	LOC106605575	N(4)-(Beta-N-acetylglucosaminyl)-L-asparaginase-like	13.22	1.60	3.03	0.00	0.01
106612738	LOC106612738	uncharacterized LOC106612738	3.50	1.60	3.03	0.01	0.02
106571642	LOC106571642	potassium channel subfamily K member 10-like	3.21	1.60	3.03	0.00	0.01
106573240	LOC106573240	ankyrin repeat and IBR domain-containing protein 1-like	11.76	1.60	3.03	0.00	0.00
106601276	LOC106601276	uncharacterized LOC106601276	4.58	1.60	3.02	0.00	0.00
106578205	LOC106578205	SH3 domain-containing protein 19-like	2.78	1.59	3.02	0.00	0.01
106562362	LOC106562362	tripartite motif-containing protein 66-like	21.46	1.59	3.02	0.00	0.00
106609291	LOC106609291	plexin-C1-like	5.43	1.59	3.02	0.00	0.01
106562908	LOC106562908	calcium/calmodulin-dependent protein kinase type II subunit beta	3.07	1.59	3.02	0.00	0.00
106583398	LOC106583398	uncharacterized LOC106583398	11.44	1.59	3.01	0.01	0.04
106602317	LOC106602317	probable E3 ubiquitin-protein ligase HERC6	36.69	1.59	3.01	0.00	0.00
106609550	LOC106609550	angiopoietin-related protein 5-like	25.64	1.59	3.01	0.00	0.00
106565108	LOC106565108	bombesin receptor-activated protein C6orf89 homolog	721.70	1.59	3.01	0.00	0.00
106604702	cssa05hxorf65	chromosome ssa05 open reading frame, human CXorf65	3.25	1.59	3.01	0.00	0.00
100195060	ets2	v-ets erythroblastosis virus E26 oncogene homolog 2 (avian)	21.93	1.59	3.01	0.00	0.00
106567876	LOC106567876	acetylcholine receptor subunit gamma-like	16.38	1.59	3.01	0.00	0.01
100196256	stat1	Signal transducer and activator of transcription 1	6.87	1.59	3.00	0.00	0.00
106566176	LOC106566176	sperm-specific antigen 2 homolog	11.01	1.58	3.00	0.00	0.02
106567317	LOC106567317	neural proliferation differentiation and control protein 1-like	81.79	1.58	3.00	0.00	0.00

106600392	LOC106600392	rho GTPase-activating protein 30-like	17.27	1.58	2.99	0.00	0.00
106585882	LOC106585882	C-C motif chemokine 4-like	5.91	1.58	2.99	0.00	0.01
106601448	LOC106601448	growth arrest-specific protein 7-like	52.39	1.58	2.99	0.00	0.00
100380391	tm149	Transmembrane protein 149	9.80	1.58	2.99	0.00	0.00
106601033	LOC106601033	complement C1q-like protein 2	912.01	1.58	2.99	0.00	0.00
106601770	LOC106601770	centrosomal protein of 112 kDa-like	1.95	1.58	2.99	0.01	0.04
106570897	LOC106570897	probable bifunctional methylenetetrahydrofolate dehydrogenase,	57.19	1.58	2.98	0.00	0.01
106607539	LOC106607539	calcium/calmodulin-dependent protein kinase type II subunit delt	4.36	1.58	2.98	0.00	0.00
106579491	LOC106579491	runt-related transcription factor 3-like	20.82	1.57	2.98	0.00	0.00
106580528	LOC106580528	leukocyte surface antigen CD53-like	10.42	1.57	2.98	0.00	0.01
106586665	LOC106586665	uncharacterized LOC106586665	5.53	1.57	2.98	0.01	0.05
106562810	LOC106562810	multidrug resistance-associated protein 9-like	32.34	1.57	2.97	0.00	0.01
106574320	LOC106574320	ephrin-A1-like	98.53	1.57	2.97	0.00	0.00
100194961	stoml3	stomatin like 3	103.57	1.57	2.96	0.01	0.03
106603807	sytl2	NA	24.95	1.57	2.96	0.00	0.00
106580823	rrnad1	NA	4.93	1.57	2.96	0.00	0.01
106578021	LOC106578021	purine nucleoside phosphorylase-like	182.04	1.57	2.96	0.00	0.00
106589597	fam20a	FAM20A, golgi associated secretory pathway pseudokinase	30.96	1.57	2.96	0.01	0.04
106589305	LOC106589305	glucose-6-phosphatase-like	449.47	1.56	2.96	0.00	0.00
106613678	LOC106613678	megakaryocyte-associated tyrosine-protein kinase-like	33.09	1.56	2.95	0.01	0.03
106582575	LOC106582575	MAGUK p55 subfamily member 4-like	2.28	1.56	2.95	0.00	0.00
106573537	LOC106573537	fibronectin-like	12.57	1.56	2.95	0.00	0.00
106604524	tdo2	NA	7099.89	1.56	2.94	0.00	0.00
106578476	vav3	NA	3.17	1.56	2.94	0.00	0.00
106569352	LOC106569352	arf-GAP with GTPase, ANK repeat and PH domain-containing prot	7.46	1.56	2.94	0.00	0.00
106572964	LOC106572964	uncharacterized LOC106572964	4.40	1.56	2.94	0.01	0.03
106609289	LOC106609289	plexin-C1-like	33.15	1.56	2.94	0.00	0.00
106563228	LOC106563228	retinoic acid receptor RXR-alpha-A	5.03	1.55	2.94	0.00	0.00
106575180	LOC106575180	beta-2-glycoprotein 1-like	2.08	1.55	2.93	0.00	0.00
106576927	LOC106576927	uncharacterized LOC106576927	2.08	1.55	2.93	0.01	0.02
106588628	LOC106588628	receptor-type tyrosine-protein phosphatase U-like	2.94	1.55	2.93	0.00	0.01
106570240	LOC106570240	membrane progestin receptor alpha-B-like	6.90	1.55	2.93	0.00	0.01
106606572	LOC106606572	arf-GAP with dual PH domain-containing protein 2-like	5.20	1.55	2.93	0.01	0.05
106608991	LOC106608991	calpain-1 catalytic subunit-like	156.43	1.55	2.93	0.01	0.04
106599413	astn1	astrotactin 1	2.21	1.55	2.93	0.00	0.00
106586170	LOC106586170	cytohesin-interacting protein-like	17.33	1.55	2.92	0.00	0.00
106562181	LOC106562181	uncharacterized LOC106562181	2.63	1.55	2.92	0.00	0.00
106585438	LOC106585438	LIM domain transcription factor LMO4.1-like	5.12	1.55	2.92	0.00	0.00
106607470	LOC106607470	uncharacterized LOC106607470	4.60	1.54	2.92	0.00	0.00
106606685	LOC106606685	uncharacterized LOC106606685	4.77	1.54	2.91	0.00	0.00
106580113	LOC106580113	galactosylceramide sulfotransferase-like	5.24	1.54	2.91	0.00	0.01
106610565	LOC106610565	uncharacterized LOC106610565	4.20	1.54	2.91	0.00	0.01
106610669	LOC106610669	cathepsin L1-like	15.20	1.54	2.91	0.00	0.00
106573988	klhl30	kelch like family member 30	28.37	1.54	2.91	0.00	0.00
106570812	LOC106570812	up-regulator of cell proliferation-like	5.12	1.54	2.91	0.00	0.00
106562030	LOC106562030	uncharacterized LOC106562030	26.07	1.54	2.91	0.00	0.00
106563255	LOC106563255	endothelial differentiation-related factor 1 homolog	100.83	1.54	2.91	0.00	0.00
106580158	LOC106580158	rhotekin-like	4.66	1.54	2.91	0.01	0.04
106567252	LOC106567252	collagen alpha-1(I) chain-like	18.92	1.54	2.91	0.00	0.00
106569315	cables1	Cdk5 and Abl enzyme substrate 1	4.08	1.54	2.90	0.00	0.00
106562346	LOC106562346	la-related protein 6-like	9.51	1.54	2.90	0.00	0.00

100380585	rab9a	RAB9A, member RAS oncogene family	27.94	1.54	2.90	0.00	0.00
106560919	LOC106560919	protein NDRG4-like	5.45	1.54	2.90	0.00	0.01
106604303	LOC106604303	uncharacterized LOC106604303	10.66	1.54	2.90	0.00	0.00
106570109	LOC106570109	pre-B-cell leukemia transcription factor-interacting protein 1-like	133.21	1.53	2.90	0.00	0.00
106565235	LOC106565235	twist-related protein 2-like	2.39	1.53	2.90	0.01	0.04
106602649	LOC106602649	T-cell surface glycoprotein CD5-like	12.27	1.53	2.90	0.00	0.00
106577966	LOC106577966	nuclear factor 7, brain-like	11.67	1.53	2.89	0.01	0.03
106568985	LOC106568985	probable ATP-dependent DNA helicase HFM1	26.48	1.53	2.89	0.00	0.00
106584350	LOC106584350	glypican-5-like	2.19	1.53	2.89	0.00	0.00
106575700	LOC106575700	uncharacterized LOC106575700	41.37	1.53	2.89	0.00	0.00
106589578	LOC106589578	brain-specific angiogenesis inhibitor 1-associated protein 2-like pr	2.85	1.53	2.89	0.00	0.00
106611010	LOC106611010	ataxin-3-like	42.77	1.53	2.88	0.00	0.00
106561206	LOC106561206	cystathionine beta-synthase-like	14.21	1.53	2.88	0.00	0.00
106563389	LOC106563389	period circadian protein homolog 1-like	159.05	1.53	2.88	0.00	0.00
106562510	LOC106562510	cryptochrome-2-like	4.78	1.53	2.88	0.01	0.05
106586468	LOC106586468	calcium/calmodulin-dependent 3',5'-cyclic nucleotide phosphodie	19.82	1.53	2.88	0.00	0.01
106569646	LOC106569646	kelch-like protein 24	5.81	1.52	2.88	0.00	0.00
106577180	LOC106577180	galectin-related protein B-like	10.86	1.52	2.88	0.00	0.00
106562530	LOC106562530	uncharacterized LOC106562530	40.48	1.52	2.88	0.00	0.02
106601599	sos2	NA	12.92	1.52	2.87	0.00	0.00
106565553	LOC106565553	arrestin domain-containing protein 1-like	12.92	1.52	2.87	0.00	0.00
106613845	LOC106613845	tyrosine-protein kinase ZAP-70-like	10.90	1.52	2.87	0.00	0.00
106586895	LOC106586895	E3 ubiquitin-protein ligase TRIM39-like	8.02	1.52	2.87	0.01	0.03
106566474	LOC106566474	uncharacterized LOC106566474	3.63	1.52	2.87	0.00	0.00
106572202	tprg1l	NA	17.89	1.52	2.87	0.00	0.00
106613637	LOC106613637	interleukin-6 receptor subunit beta-like	8.07	1.52	2.86	0.00	0.01
106601527	ubald2	NA	36.63	1.52	2.86	0.00	0.00
106603693	LOC106603693	protein-tyrosine kinase 2-beta-like	22.65	1.52	2.86	0.00	0.00
106609505	LOC106609505	cystine/glutamate transporter-like	298.35	1.52	2.86	0.01	0.03
106565863	LOC106565863	ankyrin repeat and SAM domain-containing protein 1A-like	38.23	1.52	2.86	0.01	0.03
106561510	LOC106561510	laminin subunit beta-4-like	113.87	1.52	2.86	0.00	0.00
106605567	LOC106605567	C-X-C chemokine receptor type 3-like	8.06	1.52	2.86	0.00	0.00
106562123	LOC106562123	CUGBP Elav-like family member 4	138.64	1.52	2.86	0.00	0.00
106568527	LOC106568527	cyclin-G2-like	48.00	1.51	2.86	0.00	0.00
106608989	LOC106608989	SLAM family member 9-like	17.44	1.51	2.86	0.00	0.00
106609328	LOC106609328	transcription factor SOX-5-like	168.48	1.51	2.86	0.00	0.00
106565782	LOC106565782	granzyme K-like	13.99	1.51	2.85	0.00	0.00
106581845	slc40a1	NA	25.08	1.51	2.85	0.00	0.01
106599149	LOC106599149	piggyBac transposable element-derived protein 4-like	16.35	1.51	2.85	0.00	0.00
106561493	LOC106561493	neuron navigator 2-like	173.96	1.51	2.85	0.00	0.00
106561491	LOC106561491	uncharacterized LOC106561491	12.06	1.51	2.85	0.00	0.00
106573819	LOC106573819	UPF0469 protein KIAA0907 homolog	81.96	1.51	2.85	0.00	0.01
106575218	LOC106575218	major facilitator superfamily domain-containing protein 6-A-like	14.22	1.51	2.84	0.00	0.00
106570873	LOC106570873	sonic hedgehog protein-like	2276.29	1.51	2.84	0.00	0.01
106600396	LOC106600396	zinc finger and SCAN domain-containing protein 2-like	6.71	1.51	2.84	0.00	0.02
106567524	LOC106567524	transcription factor 7-like 2	18.04	1.51	2.84	0.01	0.04
106603685	LOC106603685	tripartite motif-containing protein 16-like	7.51	1.50	2.84	0.01	0.03
106582019	LOC106582019	guanine nucleotide exchange factor DBS-like	66.70	1.50	2.84	0.01	0.04
106583818	slmap	NA	10.81	1.50	2.84	0.00	0.00
106607313	LOC106607313	histone-lysine N-methyltransferase EZH1-like	56.11	1.50	2.83	0.00	0.00
106579844	LOC106579844	C-C chemokine receptor type 5-like	2.27	1.50	2.83	0.00	0.02

106574115	LOC106574115	uncharacterized LOC106574115	10.86	1.50	2.83	0.00	0.00
106586621	LOC106586621	uncharacterized LOC106586621	14.25	1.50	2.83	0.00	0.02
106564255	LOC106564255	far upstream element-binding protein 3-like	4.61	1.50	2.83	0.00	0.02
106588420	LOC106588420	guanine nucleotide-binding protein G(I)/G(S)/G(T) subunit beta-3-	11.61	1.50	2.83	0.00	0.00
106613397	LOC106613397	trehalase-like	69.93	1.50	2.82	0.00	0.00
100196280	vamp5	vesicle-associated membrane protein 5	137.50	1.50	2.82	0.00	0.00
106611396	LOC106611396	uncharacterized LOC106611396	3.69	1.50	2.82	0.00	0.00
106605375	tp53inp1	NA	229.35	1.49	2.82	0.00	0.00
106562907	LOC106562907	aftiphilin-like	18.19	1.49	2.82	0.00	0.00
106573352	LOC106573352	inactive serine protease PAMR1-like	67.68	1.49	2.82	0.00	0.00
106568453	LOC106568453	seizure 6-like protein	11.69	1.49	2.81	0.00	0.00
106589540	LOC106589540	SH3 domain-binding protein 1-like	7.96	1.49	2.81	0.00	0.00
106567335	LOC106567335	zinc finger protein 395-like	213.82	1.49	2.81	0.00	0.01
106610627	LOC106610627	ena/VASP-like protein	21.32	1.49	2.80	0.00	0.01
106603093	LOC106603093	nuclear factor related to kappa-B-binding protein-like	15.36	1.49	2.80	0.00	0.00
106581002	LOC106581002	complement C4-B-like	81.28	1.48	2.80	0.00	0.00
106585446	LOC106585446	serine/threonine-protein phosphatase 6 catalytic subunit-like	6.99	1.48	2.80	0.00	0.00
106588235	LOC106588235	GTP-binding protein Rhes pseudogene	34.80	1.48	2.80	0.00	0.00
106601093	LOC106601093	MKL/myocardin-like protein 2	5.96	1.48	2.79	0.00	0.00
106582992	LOC106582992	endothelin-converting enzyme 1-like	319.26	1.48	2.79	0.00	0.00
106611471	vps39	NA	76.55	1.48	2.79	0.00	0.00
106580061	LOC106580061	guanine deaminase-like	86.66	1.48	2.79	0.00	0.00
106582432	LOC106582432	trace amine-associated receptor 13c-like	72.78	1.48	2.79	0.00	0.02
100380499	tifa	TRAF2-binding protein	32.28	1.48	2.79	0.01	0.04
106602581	LOC106602581	lectin-like	33.26	1.48	2.79	0.01	0.03
106589480	LOC106589480	forkhead box protein K2-like	48.20	1.48	2.79	0.00	0.00
106582536	LOC106582536	T-cell-specific surface glycoprotein CD28-like	5.66	1.48	2.79	0.00	0.00
106589454	LOC106589454	probable ATP-dependent RNA helicase DDX17	42.34	1.48	2.78	0.00	0.00
106575280	LOC106575280	SLIT and NTRK-like protein 5	4.44	1.48	2.78	0.00	0.00
106571128	LOC106571128	uncharacterized LOC106571128	1151.52	1.48	2.78	0.00	0.01
106593876	LOC106593876	cytochrome P450 3A27-like	38.60	1.48	2.78	0.00	0.02
106604553	LOC106604553	SAM and SH3 domain-containing protein 3-like	30.51	1.47	2.78	0.00	0.01
106568207	LOC106568207	forkhead box protein N4-like	3.12	1.47	2.78	0.00	0.00
106562062	LOC106562062	RING finger protein 166-like	15.16	1.47	2.77	0.00	0.00
106604351	LOC106604351	tyrosine-protein kinase ITK/TSK-like	4.16	1.47	2.77	0.00	0.02
106605587	LOC106605587	rho GTPase-activating protein 33-like	17.95	1.47	2.77	0.00	0.00
106605965	LOC106605965	sialic acid-binding Ig-like lectin 15	5.92	1.47	2.77	0.00	0.01
106588402	LOC106588402	major histocompatibility complex class I-related gene protein-like	349.82	1.47	2.77	0.00	0.00
106589967	LOC106589967	uncharacterized LOC106589967	5.93	1.47	2.77	0.00	0.00
106582053	LOC106582053	B- and T-lymphocyte attenuator-like	5.58	1.47	2.77	0.00	0.00
106601172	LOC106601172	protein NLRC3-like	10.93	1.47	2.77	0.00	0.00
106604853	LOC106604853	carnosine synthase 1-like	12.16	1.47	2.77	0.01	0.03
106572813	LOC106572813	tumor necrosis factor receptor superfamily member 5-like	15.99	1.47	2.77	0.00	0.02
106602716	LOC106602716	pleckstrin homology domain-containing family H member 1-like	23.55	1.47	2.76	0.00	0.00
106566126	LOC106566126	R3H domain-containing protein 2-like	61.79	1.47	2.76	0.00	0.00
106603196	LOC106603196	calphotin-like	10.86	1.47	2.76	0.00	0.00
106582258	LOC106582258	interleukin-1 receptor type 1-like	13.14	1.47	2.76	0.00	0.00
100195074	papl	acid phosphatase 7, tartrate resistant (putative)	551.42	1.47	2.76	0.00	0.00
106612055	LOC106612055	tyrosine-protein kinase ITK/TSK-like	22.27	1.46	2.76	0.00	0.00
106603833	LOC106603833	uncharacterized LOC106603833	2.41	1.46	2.75	0.00	0.00
106610571	LOC106610571	uncharacterized LOC106610571	5.13	1.46	2.75	0.00	0.00

106588373	LOC106588373	uncharacterized LOC106588373	110.24	1.46	2.75	0.01	0.02
100194878	lck	lymphocyte-specific protein tyrosine kinase	17.40	1.46	2.75	0.00	0.00
106571232	LOC106571232	TATA box-binding protein-associated factor RNA polymerase I sub	19.04	1.46	2.75	0.00	0.00
106562881	LOC106562881	aftiphilin-like	34.17	1.46	2.74	0.00	0.00
106572629	LOC106572629	probable phospholipid-transporting ATPase IIA	6.48	1.46	2.74	0.00	0.00
106606261	LOC106606261	ketosamine-3-kinase-like	4.28	1.46	2.74	0.00	0.00
106586517	tnfsf11	NA	36.76	1.46	2.74	0.00	0.00
106582451	LOC106582451	uncharacterized LOC106582451	3.09	1.45	2.74	0.00	0.00
106568691	adipoq	adiponectin, C1Q and collagen domain containing	93.36	1.45	2.74	0.00	0.00
106610794	LOC106610794	serine/threonine-protein kinase D3-like	34.22	1.45	2.73	0.00	0.00
106574920	LOC106574920	E3 ubiquitin-protein ligase TRIM39-like	5.42	1.45	2.73	0.00	0.00
106584291	LOC106584291	cytochrome P450 2J2-like	5846.51	1.45	2.73	0.00	0.00
106612144	LOC106612144	SH3 domain and tetratricopeptide repeat-containing protein 2-like	89.92	1.45	2.73	0.00	0.00
106580394	LOC106580394	L-serine dehydratase/L-threonine deaminase-like	10.54	1.45	2.73	0.00	0.00
100195198	vps37a	VPS37A, ESCRT-I subunit	135.09	1.44	2.72	0.00	0.00
106587534	LOC106587534	ras-related protein R-Ras2	223.49	1.44	2.72	0.00	0.00
106581631	LOC106581631	uncharacterized LOC106581631	17.85	1.44	2.72	0.00	0.00
106610538	LOC106610538	scavenger receptor cysteine-rich type 1 protein M130-like	6.79	1.44	2.71	0.00	0.00
106606951	LOC106606951	forkhead box protein K2-like	9.68	1.44	2.71	0.00	0.00
100380534	bat1	HLA-B associated transcript 1	3.32	1.44	2.71	0.00	0.01
106589339	LOC106589339	somatostatin receptor type 2-like	32.61	1.44	2.71	0.00	0.00
106609294	LOC106609294	suppressor of cytokine signaling 2-like	57.29	1.44	2.71	0.00	0.02
106574276	LOC106574276	pyruvate kinase PKM-like	11.01	1.44	2.71	0.00	0.00
106589311	LOC106589311	transmembrane protein 106B-like	5.33	1.44	2.71	0.00	0.00
100380800	ngap	Ras GTPase-activating protein nGAP	41.82	1.44	2.71	0.00	0.00
106588660	LOC106588660	serine/threonine-protein kinase pdik1l-like	5.88	1.44	2.71	0.00	0.00
100380775	LOC100380775	negative elongation factor A	35.99	1.44	2.70	0.00	0.00
106581859	LOC106581859	integrin beta-2-like	88.47	1.43	2.70	0.00	0.02
106599714	LOC106599714	solute carrier family 22 member 23-like	157.89	1.43	2.70	0.00	0.00
106577690	LOC106577690	UDP-glucuronosyltransferase 1-2-like	111.78	1.43	2.70	0.00	0.00
106578001	LOC106578001	uncharacterized LOC106578001	555.67	1.43	2.70	0.00	0.00
106563242	LOC106563242	uncharacterized LOC106563242	2.76	1.43	2.70	0.00	0.00
106581115	LOC106581115	tumor necrosis factor receptor superfamily member 19-like	20.46	1.43	2.70	0.01	0.03
106579673	LOC106579673	uncharacterized LOC106579673	2.82	1.43	2.70	0.00	0.02
100194685	aff3	AF4/FMR2 family member 3	15.05	1.43	2.70	0.00	0.00
106588440	LOC106588440	myeloid-associated differentiation marker-like	5.61	1.43	2.70	0.00	0.01
106564262	LOC106564262	uncharacterized LOC106564262	12.48	1.43	2.70	0.00	0.00
106589302	LOC106589302	next to BRCA1 gene 1 protein-like	208.56	1.43	2.69	0.00	0.00
106576222	LOC106576222	uncharacterized LOC106576222	57.31	1.43	2.69	0.00	0.00
100196298	ripp1	NA	7.75	1.43	2.69	0.00	0.00
106586973	LOC106586973	intraflagellar transport protein 56-like	11.23	1.43	2.69	0.00	0.01
100136516	cd3e	CD3e molecule	56.11	1.43	2.69	0.00	0.00
106566034	LOC106566034	uncharacterized LOC106566034	45.55	1.43	2.69	0.00	0.00
106603814	LOC106603814	transcription factor 7-like 2	11.73	1.43	2.69	0.00	0.00
106572200	tp73	NA	21.36	1.43	2.69	0.00	0.00
106564951	LOC106564951	protein AF-17-like	17.16	1.43	2.69	0.00	0.02
106568120	LOC106568120	dehydrogenase/reductase SDR family member 13-like	36.99	1.42	2.68	0.00	0.00
106582437	LOC106582437	major histocompatibility complex class I-related gene protein-like	142.90	1.42	2.68	0.00	0.00
106580847	LOC106580847	uncharacterized LOC106580847	2.07	1.42	2.68	0.00	0.00
106606059	LOC106606059	cleft lip and palate transmembrane protein 1-like protein	5.05	1.42	2.68	0.00	0.00
100136450	LOC100136450	CD8 alpha	4.24	1.42	2.68	0.00	0.02

106566286	LOC106566286	uncharacterized LOC106566286	5.64	1.42	2.68	0.00	0.00
106604619	LOC106604619	phosphoinositide 3-kinase adapter protein 1-like	11.73	1.42	2.68	0.00	0.00
100526824	tbx21	T-box 21	18.23	1.42	2.68	0.00	0.00
106609237	LOC106609237	striatin-interacting protein 1 homolog	25.32	1.42	2.68	0.00	0.01
106603664	LOC106603664	dystrobrevin beta-like	9.57	1.42	2.68	0.00	0.01
106604696	LOC106604696	saxitoxin and tetrodotoxin-binding protein 2-like	110.43	1.42	2.67	0.00	0.01
106602924	LOC106602924	regulator of G-protein signaling 14-like	25.33	1.42	2.67	0.00	0.00
106601178	LOC106601178	NF-kappa-B inhibitor alpha-like	133.58	1.42	2.67	0.00	0.00
106573249	LOC106573249	mitochondrial inner membrane protease subunit 2-like	1027.82	1.42	2.67	0.00	0.00
106561231	shank3	NA	7.59	1.42	2.67	0.00	0.00
106601418	LOC106601418	butyrophilin subfamily 1 member A1-like	12.05	1.42	2.67	0.01	0.03
106579933	LOC106579933	gamma-glutamyltranspeptidase 1-like	23.16	1.42	2.67	0.00	0.00
100196398	fa60a	FAM60A	7.18	1.42	2.67	0.00	0.02
106565096	LOC106565096	phosphatidylinositol 4-phosphate 3-kinase C2 domain-containing	19.94	1.42	2.67	0.00	0.00
106593759	LOC106593759	cytochrome P450 3A27-like	232.87	1.42	2.67	0.00	0.00
106609716	LOC106609716	zinc finger protein 624-like	2.04	1.42	2.67	0.00	0.01
106573372	LOC106573372	metastasis-associated in colon cancer protein 1-like	13.73	1.41	2.67	0.00	0.00
106611589	LOC106611589	leukocyte cell-derived chemotaxin-2-like	99.54	1.41	2.67	0.00	0.00
106581784	LOC106581784	retinol dehydrogenase 3-like	112.81	1.41	2.66	0.00	0.00
106567712	LOC106567712	uncharacterized LOC106567712	2.63	1.41	2.66	0.00	0.00
106568725	LOC106568725	UDP-N-acetylglucosamine/UDP-glucose/GDP-mannose transporte	170.12	1.41	2.66	0.00	0.00
106575521	LOC106575521	uncharacterized LOC106575521	24.92	1.41	2.66	0.00	0.00
106565851	LOC106565851	nicotinamide phosphoribosyltransferase-like	70.27	1.41	2.66	0.00	0.00
106561468	LOC106561468	uncharacterized LOC106561468	1.93	1.41	2.66	0.00	0.00
106575018	LOC106575018	BTB/POZ domain-containing protein KCTD12-like	16.18	1.41	2.66	0.00	0.00
106578733	LOC106578733	zinc finger protein PLAG1-like	6.90	1.41	2.65	0.00	0.00
106586445	LOC106586445	zinc finger protein Helios-like	11.05	1.41	2.65	0.01	0.03
106569935	LOC106569935	major histocompatibility complex class I-related gene protein-like	15.44	1.41	2.65	0.00	0.00
106573901	LOC106573901	interleukin-6 receptor subunit beta-like	3.76	1.41	2.65	0.01	0.04
100380309	tmm34	Transmembrane protein 34	39.88	1.41	2.65	0.00	0.00
106607026	LOC106607026	zinc finger protein Gfi-1b-like	7.48	1.41	2.65	0.00	0.00
106569780	LOC106569780	T-cell differentiation antigen CD6-like	27.84	1.41	2.65	0.00	0.00
106600855	LOC106600855	uncharacterized LOC106600855	9.24	1.41	2.65	0.00	0.01
100380614	ikzf1	IKAROS family zinc finger 1	23.03	1.40	2.65	0.00	0.02
106561096	LOC106561096	abhydrolase domain-containing protein 2-B-like	6.16	1.40	2.65	0.00	0.00
106578744	LOC106578744	lysosomal-trafficking regulator-like	3.56	1.40	2.65	0.00	0.00
106572796	LOC106572796	differentially expressed in FDCP 6 homolog	10.37	1.40	2.65	0.00	0.00
106575090	cpo	carboxypeptidase O	190.34	1.40	2.64	0.00	0.00
106563046	LOC106563046	junction-mediating and -regulatory protein-like	99.94	1.40	2.64	0.01	0.05
106577095	LOC106577095	transmembrane protease serine 9-like	307.53	1.40	2.64	0.00	0.00
106570689	LOC106570689	MARCKS-related protein-like	200.91	1.40	2.64	0.00	0.00
106564646	LOC106564646	rab11 family-interacting protein 4A-like	21.45	1.40	2.64	0.01	0.03
106564565	LOC106564565	probable ATP-dependent RNA helicase DDX17	132.83	1.40	2.64	0.00	0.00
106612923	LOC106612923	uncharacterized LOC106612923	121.93	1.40	2.64	0.00	0.00
106568682	LOC106568682	uncharacterized LOC106568682	2.33	1.40	2.64	0.01	0.03
106568196	LOC106568196	proline dehydrogenase 1, mitochondrial-like	3.73	1.40	2.64	0.01	0.05
106587528	LOC106587528	transcription factor SOX-6-like	6.57	1.40	2.64	0.00	0.00
106575016	LOC106575016	LIM domain only protein 7-like	17.37	1.40	2.64	0.00	0.02
100195897	lrat	Lecithin retinol acyltransferase	28.61	1.40	2.64	0.00	0.00
106578142	LOC106578142	prospero homeobox protein 1-like	151.82	1.40	2.63	0.00	0.00
106582820	LOC106582820	alpha-1,3-galactosyltransferase 2-like	3.74	1.40	2.63	0.00	0.01

106568833	LOC106568833	frizzled-8-like	10.37	1.40	2.63	0.00	0.00
106589968	LOC106589968	butyrophilin subfamily 1 member A1-like	29.79	1.40	2.63	0.00	0.01
106572146	LOC106572146	perlwapin-like	93.67	1.39	2.63	0.00	0.00
106578761	LOC106578761	microfibril-associated glycoprotein 4-like	50.72	1.39	2.63	0.00	0.00
106580668	LOC106580668	E3 ubiquitin-protein ligase rififylin-like	7.02	1.39	2.63	0.01	0.04
106608571	LOC106608571	zinc finger CCHC domain-containing protein 4-like	28.92	1.39	2.62	0.00	0.00
106566146	LOC106566146	sterol O-acyltransferase 2-like	672.26	1.39	2.62	0.00	0.00
106612894	LOC106612894	neurobeachin-like	4.33	1.39	2.62	0.00	0.00
106572127	LOC106572127	tyrosine-protein kinase HCK-like	7.61	1.39	2.62	0.00	0.01
106590023	LOC106590023	homeobox protein Mohawk-like	25.20	1.39	2.61	0.00	0.00
106607718	bach2	BTB domain and CNC homolog 2	4.38	1.39	2.61	0.00	0.01
106565679	LOC106565679	integrin beta-7-like	41.58	1.39	2.61	0.01	0.03
106587865	LOC106587865	SWI/SNF-related matrix-associated actin-dependent regulator of c	29.48	1.39	2.61	0.00	0.00
106577791	LOC106577791	matrix metalloproteinase-14-like	355.99	1.38	2.61	0.00	0.00
106561150	LOC106561150	nuclear receptor ROR-alpha	6.87	1.38	2.61	0.00	0.00
106560633	LOC106560633	tyrosine-protein kinase RYK-like	2.97	1.38	2.61	0.00	0.00
106588896	LOC106588896	uncharacterized LOC106588896	5.88	1.38	2.61	0.00	0.01
106582148	LOC106582148	cystathionine beta-synthase-like	401.64	1.38	2.61	0.00	0.00
106567405	LOC106567405	carbohydrate-responsive element-binding protein-like	34.60	1.38	2.61	0.00	0.00
106563396	LOC106563396	probable G-protein coupled receptor 34	6.77	1.38	2.60	0.00	0.00
106568685	LOC106568685	prostaglandin E2 receptor EP4 subtype-like	9.83	1.38	2.60	0.00	0.00
100194767	pim1	pim-1 oncogene	213.65	1.38	2.60	0.00	0.00
106613085	LOC106613085	protein EVI2B-like	15.49	1.38	2.60	0.01	0.03
100286550	thap4	THAP domain containing 4	3.47	1.38	2.60	0.00	0.00
106608736	LOC106608736	uncharacterized LOC106608736	3.82	1.38	2.60	0.00	0.00
106565675	LOC106565675	uncharacterized LOC106565675	120.16	1.38	2.60	0.00	0.00
106569300	LOC106569300	uncharacterized LOC106569300	132.48	1.38	2.60	0.00	0.00
106600699	LOC106600699	transient receptor potential cation channel subfamily M member	3.33	1.38	2.60	0.00	0.00
106608633	LOC106608633	serum amyloid P-component-like	3177.48	1.38	2.59	0.00	0.00
106608649	LOC106608649	stabilizer of axonemal microtubules 2-like	3.66	1.38	2.59	0.00	0.00
106603155	LOC106603155	GC-rich sequence DNA-binding factor 2-like	27.52	1.37	2.59	0.00	0.01
106564061	LOC106564061	ADP-ribosylation factor-binding protein GGA2-like	49.71	1.37	2.59	0.00	0.00
106578556	LOC106578556	supervillin-like	16.17	1.37	2.59	0.00	0.00
106613787	LOC106613787	transmembrane protein 59-like	13.15	1.37	2.59	0.00	0.00
106568351	LOC106568351	protein prune homolog 2-like	97.41	1.37	2.59	0.00	0.00
106567382	LOC106567382	large neutral amino acids transporter small subunit 1-like	22.43	1.37	2.59	0.00	0.00
106562051	LOC106562051	matrix metalloproteinase-15-like	40.60	1.37	2.59	0.00	0.00
106575161	LOC106575161	bromodomain adjacent to zinc finger domain protein 2B-like	49.10	1.37	2.59	0.00	0.00
106590061	LOC106590061	succinate--hydroxymethylglutarate CoA-transferase-like	10.92	1.37	2.59	0.00	0.00
106609588	LOC106609588	UBA-like domain-containing protein 1	35.10	1.37	2.59	0.00	0.00
106610532	LOC106610532	protogenin B-like	95.08	1.37	2.58	0.00	0.01
106608028	LOC106608028	heme-binding protein 2-like	30.28	1.37	2.58	0.00	0.00
106574101	cep63	centrosomal protein 63	6.25	1.37	2.58	0.00	0.00
106601034	LOC106601034	complement C1q-like protein 2	39.85	1.37	2.58	0.01	0.03
106583810	LOC106583810	nischarin-like	8.59	1.37	2.58	0.01	0.03
106565673	LOC106565673	TRAF3-interacting JNK-activating modulator-like	17.67	1.37	2.58	0.00	0.00
106575853	LOC106575853	chloride channel protein 1-like	3.78	1.37	2.58	0.00	0.00
106588183	LOC106588183	E3 ubiquitin-protein ligase RNF115-like	15.17	1.37	2.58	0.00	0.00
106576185	LOC106576185	hemagglutinin/amebocyte aggregation factor-like	3385.34	1.36	2.57	0.00	0.00
106600395	LOC106600395	zinc finger protein 263-like	26.57	1.36	2.57	0.00	0.00
106576926	tmem72	NA	30.11	1.36	2.57	0.00	0.00

100195200	LOC100195200	dual specificity testis-specific protein kinase 2	3.21	1.36	2.57	0.00	0.00
106589645	LOC106589645	rho GTPase-activating protein 12-like	28.07	1.36	2.57	0.00	0.00
106571258	LOC106571258	CCAAT/enhancer-binding protein zeta-like	59.64	1.36	2.57	0.00	0.00
106581520	LOC106581520	fibroblast growth factor receptor homolog 1-like	4.28	1.36	2.57	0.00	0.00
100194829	creg2	cellular repressor of E1A stimulated genes 2	116.20	1.36	2.56	0.01	0.03
106588354	LOC106588354	tumor necrosis factor alpha-induced protein 8-like protein 2	18.65	1.36	2.56	0.01	0.04
106602945	LOC106602945	putative bifunctional UDP-N-acetylglucosamine transferase and d	2.84	1.35	2.56	0.00	0.00
106580765	LOC106580765	calcineurin B homologous protein 2-like	21.28	1.35	2.56	0.00	0.00
106579879	LOC106579879	BCL2/adenovirus E1B 19 kDa protein-interacting protein 3-like	94.55	1.35	2.56	0.00	0.00
106560564	LOC106560564	mesoderm induction early response protein 3-like	4.19	1.35	2.56	0.01	0.04
100380613	stea4	Metalloreductase STEAP4	981.20	1.35	2.56	0.01	0.04
106610559	LOC106610559	uncharacterized LOC106610559	36.62	1.35	2.56	0.00	0.00
100196483	atp7a	ATPase, Cu++ transporting, alpha polypeptide	8.82	1.35	2.55	0.00	0.00
106613835	LOC106613835	leupaxin-like	31.22	1.35	2.55	0.00	0.00
100286497	nbl1	neuroblastoma, suppression of tumorigenicity 1	266.73	1.35	2.55	0.00	0.00
106607465	LOC106607465	ABI gene family member 3-like	27.42	1.35	2.55	0.00	0.01
106602658	LOC106602658	tyrosine-protein phosphatase non-receptor type 9-like	3.39	1.35	2.55	0.00	0.01
106613446	LOC106613446	GTPase IMAP family member 4-like	91.62	1.35	2.55	0.00	0.00
106610540	LOC106610540	heparan sulfate glucosamine 3-O-sulfotransferase 3B1-like	20.85	1.35	2.55	0.00	0.00
106563597	LOC106563597	uncharacterized LOC106563597	286.54	1.35	2.55	0.00	0.00
106592756	LOC106592756	nucleoredoxin-like protein 1	2.22	1.35	2.54	0.00	0.00
106589306	LOC106589306	glucose-6-phosphatase-like	459.18	1.35	2.54	0.00	0.00
106586917	LOC106586917	tubulin alpha chain	6.02	1.35	2.54	0.00	0.00
106586142	LOC106586142	signal transducer and activator of transcription 1-alpha/beta-like	61.06	1.35	2.54	0.00	0.00
106568697	LOC106568697	abl interactor 1-like	8.16	1.35	2.54	0.00	0.00
106561748	LOC106561748	pollen-specific leucine-rich repeat extensin-like protein 4	2.37	1.34	2.54	0.00	0.00
106583333	LOC106583333	uncharacterized LOC106583333	6.55	1.34	2.54	0.00	0.00
106599418	brinp2	BMP/retinoic acid inducible neural specific 2	7.02	1.34	2.54	0.00	0.00
106561474	LOC106561474	tripartite motif-containing protein 47-like	8.89	1.34	2.54	0.01	0.04
106571131	LOC106571131	zinc finger and BTB domain-containing protein 25-like	23.80	1.34	2.54	0.00	0.00
106573411	LOC106573411	receptor-interacting serine/threonine-protein kinase 3-like	9.43	1.34	2.54	0.00	0.00
106571127	LOC106571127	kinesin-like protein KIF26A	4.30	1.34	2.54	0.00	0.00
106567731	arhgap32	Rho GTPase activating protein 32	69.69	1.34	2.54	0.00	0.00
106568793	LOC106568793	uncharacterized LOC106568793	9.93	1.34	2.53	0.00	0.00
106584208	prkaa2	NA	19.81	1.34	2.53	0.00	0.00
106566719	LOC106566719	probable ATP-dependent RNA helicase DDX23	23.47	1.34	2.53	0.00	0.00
106575843	LOC106575843	uncharacterized LOC106575843	2.22	1.34	2.53	0.00	0.00
106599909	LOC106599909	interferon regulatory factor 4-like	5.56	1.34	2.53	0.00	0.00
106585516	LOC106585516	E3 ubiquitin-protein ligase DTX1-like	10.71	1.34	2.53	0.00	0.00
106561501	LOC106561501	tripartite motif-containing protein 16-like	8.88	1.34	2.53	0.00	0.00
100846958	p3ip1	Phosphoinositide-3-kinase-interacting protein 1 precursor	27.24	1.34	2.53	0.00	0.00
106598874	LOC106598874	CMRF35-like molecule 9	3.84	1.34	2.52	0.00	0.00
106586887	LOC106586887	interferon-induced GTP-binding protein Mx-like	4.36	1.34	2.52	0.00	0.01
106608622	LOC106608622	alkaline ceramidase 2-like	26.70	1.34	2.52	0.00	0.00
106588553	LOC106588553	uncharacterized LOC106588553	81.00	1.33	2.52	0.00	0.00
106607255	LOC106607255	alpha-2,8-sialyltransferase 8E-like	405.90	1.33	2.52	0.00	0.00
106580972	LOC106580972	integrator complex subunit 4-like	42.74	1.33	2.52	0.00	0.02
106603578	LOC106603578	protein FAM122A-like	3.94	1.33	2.52	0.01	0.05
106611581	LOC106611581	teneurin-3-like	18.29	1.33	2.52	0.00	0.00
100380737	LOC100380737	liprin-beta-2	149.55	1.33	2.52	0.00	0.02
106611124	hs1bp3	HCLS1 binding protein 3	97.27	1.33	2.52	0.00	0.00

106607199	LOC106607199	synaptosomal-associated protein 25-B-like	2.25	1.33	2.52	0.00	0.00
106584116	fip1l1	factor interacting with PAPOLA and CPSF1	45.79	1.33	2.52	0.00	0.02
106567083	LOC106567083	carboxy-terminal domain RNA polymerase II polypeptide A small p	137.82	1.33	2.52	0.00	0.00
106584061	LOC106584061	terminal uridylyltransferase 4-like	18.59	1.33	2.52	0.00	0.01
106573028	LOC106573028	tyrosine-protein kinase CSK-like	25.64	1.33	2.52	0.00	0.00
106569529	LOC106569529	methyl-CpG-binding domain protein 3-like	28.30	1.33	2.52	0.01	0.03
106560999	LOC106560999	uncharacterized LOC106560999	6.39	1.33	2.51	0.00	0.00
106588684	LOC106588684	uncharacterized LOC106588684	15.93	1.33	2.51	0.00	0.00
106572888	LOC106572888	epidermal growth factor receptor kinase substrate 8-like protein 3	60.18	1.33	2.51	0.01	0.03
106572943	LOC106572943	uncharacterized LOC106572943	2.27	1.33	2.51	0.00	0.01
106585852	LOC106585852	SH2B adapter protein 3-like	23.04	1.33	2.51	0.00	0.00
106573062	LOC106573062	uncharacterized LOC106573062	78.12	1.33	2.51	0.00	0.00
106604522	tenm3	NA	157.39	1.33	2.51	0.01	0.04
106584667	LOC106584667	E3 ubiquitin/ISG15 ligase TRIM25-like	17.51	1.33	2.51	0.00	0.00
106587146	LOC106587146	adhesion G-protein coupled receptor G5-like	4.86	1.32	2.50	0.00	0.01
106572046	pik3cd	NA	9.15	1.32	2.50	0.00	0.00
106578354	LOC106578354	uncharacterized LOC106578354	4.17	1.32	2.50	0.00	0.01
106602929	LOC106602929	amyloid beta A4 precursor protein-binding family B member 3-like	6.56	1.32	2.50	0.00	0.01
106611208	LOC106611208	uncharacterized LOC106611208	4.04	1.32	2.50	0.00	0.00
106566613	LOC106566613	uncharacterized LOC106566613	22.41	1.32	2.50	0.00	0.00
106610616	LOC106610616	integral membrane protein GPR137B-like	78.50	1.32	2.50	0.00	0.00
106601479	LOC106601479	zinc finger protein 239-like	17.36	1.32	2.50	0.00	0.01
106587677	LOC106587677	spermatogenesis-associated protein 5-like protein 1	7.29	1.32	2.50	0.01	0.05
100286545	tmm80	Transmembrane protein 80	5.33	1.32	2.50	0.00	0.00
106575489	LOC106575489	tuftelin-like	4.79	1.32	2.50	0.01	0.04
106570091	LOC106570091	secretory carrier-associated membrane protein 3-like	22.75	1.32	2.50	0.00	0.00
106561963	LOC106561963	F-box only protein 3-like	4.70	1.32	2.50	0.01	0.03
106611571	LOC106611571	RING finger protein 145-like	16.31	1.32	2.50	0.00	0.01
106566408	LOC106566408	uncharacterized LOC106566408	5.28	1.32	2.49	0.00	0.01
106585087	LOC106585087	ankyrin repeat and LEM domain-containing protein 2-like	86.27	1.32	2.49	0.00	0.00
106564359	LOC106564359	myopalladin-like	7.17	1.32	2.49	0.01	0.04
106576902	LOC106576902	transmembrane protein 254-like	53.97	1.32	2.49	0.01	0.03
106607746	LOC106607746	probable ribonuclease ZC3H12D	6.03	1.32	2.49	0.00	0.00
106583670	LOC106583670	nuclear factor of activated T-cells, cytoplasmic 2-like	5.88	1.32	2.49	0.00	0.00
106589471	LOC106589471	protein unc-13 homolog D-like	22.06	1.32	2.49	0.00	0.01
106609557	LOC106609557	differentially expressed in FDCP 6-like	128.48	1.32	2.49	0.01	0.05
106562605	LOC106562605	glutamine and serine-rich protein 1-like	15.32	1.32	2.49	0.00	0.00
100136526	LOC100136526	peroxisome proliferator-activated receptor delta	37.57	1.32	2.49	0.01	0.04
106563178	LOC106563178	poly [ADP-ribose] polymerase 14-like	25.84	1.32	2.49	0.00	0.00
106604350	LOC106604350	LON peptidase N-terminal domain and RING finger protein 1-like	16.80	1.32	2.49	0.00	0.00
106579999	LOC106579999	folylpolyglutamate synthase, mitochondrial-like	368.32	1.31	2.49	0.00	0.00
106578760	LOC106578760	microfibril-associated glycoprotein 4-like	109.78	1.31	2.48	0.00	0.02
106606278	LOC106606278	protein NLRC3-like	43.66	1.31	2.48	0.00	0.00
106570487	LOC106570487	regulation of nuclear pre-mRNA domain-containing protein 1A-lik	16.23	1.31	2.48	0.00	0.02
106608840	LOC106608840	ankyrin repeat and IBR domain-containing protein 1-like	2.98	1.31	2.48	0.00	0.02
106580818	LOC106580818	SID1 transmembrane family member 2-like	59.91	1.31	2.48	0.00	0.00
100194852	grp2	RAS guanyl-releasing protein 2	43.87	1.31	2.48	0.00	0.00
106573759	LOC106573759	histone-lysine N-methyltransferase SUV420H1-like	13.41	1.31	2.48	0.00	0.00
106579503	LOC106579503	receptor-type tyrosine-protein kinase FLT3-like	32.79	1.31	2.48	0.00	0.00
106600234	LOC106600234	leucine-rich repeat-containing protein 16B-like	18.13	1.31	2.48	0.00	0.00
100196721	peci	Peroxisomal 3,2-trans-enoyl-CoA isomerase	474.52	1.31	2.48	0.00	0.00

106602697	LOC106602697	SLAM family member 5-like	11.05	1.31	2.48	0.01	0.04
106564592	kdm2b	lysine demethylase 2B	18.39	1.31	2.48	0.01	0.03
106612323	LOC106612323	calnexin-like	3.38	1.31	2.48	0.00	0.01
106586745	LOC106586745	DDB1- and CUL4-associated factor 6-like	30.80	1.31	2.48	0.01	0.04
106582135	LOC106582135	ras-related protein Rab-20-like	305.37	1.31	2.48	0.00	0.01
106560657	LOC106560657	uncharacterized LOC106560657	14.32	1.31	2.47	0.00	0.00
106574108	LOC106574108	endonuclease domain-containing 1 protein-like	5.87	1.31	2.47	0.00	0.00
106610678	LOC106610678	zinc finger protein Rlf-like	18.74	1.31	2.47	0.00	0.01
106600907	LOC106600907	uncharacterized LOC106600907	47.88	1.31	2.47	0.00	0.00
106588197	LOC106588197	eomesodermin-like	5.07	1.30	2.47	0.00	0.00
106579920	LOC106579920	filamin-interacting protein FAM101A-like	148.97	1.30	2.47	0.00	0.00
106610498	LOC106610498	zinc finger protein 628-like	3.71	1.30	2.47	0.00	0.01
106598245	LOC106598245	fibroblast growth factor receptor-like 1	58.35	1.30	2.47	0.01	0.03
106599958	LOC106599958	CDK5 and ABL1 enzyme substrate 1-like	45.05	1.30	2.47	0.00	0.02
100306863	mmp25	Matrix metalloproteinase-25	9.27	1.30	2.47	0.00	0.00
106600740	LOC106600740	linker for activation of T-cells family member 1-like	56.92	1.30	2.47	0.00	0.02
106580424	LOC106580424	B-cell linker protein-like	575.39	1.30	2.46	0.01	0.04
106582501	LOC106582501	interferon-induced guanylate-binding protein 1-like	33.47	1.30	2.46	0.00	0.00
106589772	LOC106589772	stromal cell-derived factor 1-like	103.83	1.30	2.46	0.00	0.00
106601031	LOC106601031	coenzyme Q-binding protein COQ10 homolog, mitochondrial-like	249.75	1.30	2.46	0.00	0.00
106571219	nfkbie	NFKB inhibitor epsilon	23.30	1.30	2.46	0.00	0.00
106582838	bpifb2	BPI fold containing family B member 2	76.79	1.30	2.46	0.00	0.00
106560216	LOC106560216	homeobox protein Meis1-like	2.96	1.30	2.46	0.01	0.03
106579655	LOC106579655	rho GTPase-activating protein 18-like	4.00	1.30	2.46	0.00	0.00
106584705	LOC106584705	transcription factor PU.1-like	48.39	1.30	2.46	0.00	0.00
106584009	LOC106584009	myomegalin-like	4.62	1.30	2.46	0.00	0.00
106582383	rab3gap1	NA	23.09	1.30	2.46	0.01	0.04
106613976	LOC106613976	dynamin-2-like	16.74	1.30	2.46	0.00	0.00
106564527	LOC106564527	hematopoietic lineage cell-specific protein-like	26.08	1.30	2.45	0.00	0.02
106609842	LOC106609842	class A basic helix-loop-helix protein 15-like	2.87	1.30	2.45	0.01	0.05
106606911	LOC106606911	solute carrier family 25 member 39-like	16.16	1.30	2.45	0.01	0.04
106561707	LOC106561707	5'-nucleotidase domain-containing protein 3-like	4.84	1.29	2.45	0.00	0.00
106573632	LOC106573632	microtubule-associated proteins 1A/1B light chain 3B	302.62	1.29	2.45	0.00	0.00
106586786	LOC106586786	peripheral plasma membrane protein CASK-like	5.36	1.29	2.45	0.00	0.00
106567101	LOC106567101	WNT1-inducible-signaling pathway protein 2-like	44.29	1.29	2.45	0.00	0.00
106599812	pcmtd1	protein-L-isoaspartate (D-aspartate) O-methyltransferase domain	38.16	1.29	2.45	0.00	0.00
106570004	LOC106570004	hepatic leukemia factor-like	10.98	1.29	2.45	0.00	0.00
106564204	LOC106564204	saposin-C-like	12.85	1.29	2.45	0.00	0.00
100380563	hhatl	hedgehog acyltransferase-like	2.39	1.29	2.45	0.00	0.00
100195997	mgp	matrix Gla protein	49.07	1.29	2.45	0.00	0.00
106582073	LOC106582073	phospholipase A1 member A-like	1414.24	1.29	2.45	0.00	0.00
106562061	LOC106562061	cytoplasmic tRNA 2-thiolation protein 2-like	29.87	1.29	2.44	0.01	0.03
106608876	LOC106608876	uncharacterized LOC106608876	32.66	1.29	2.44	0.01	0.03
106592100	LOC106592100	calcium-binding mitochondrial carrier protein SCaMC-3-like	11.90	1.29	2.44	0.01	0.03
106578585	lck	LCK proto-oncogene, Src family tyrosine kinase	9.05	1.29	2.44	0.00	0.00
100526765	LOC100526765	vascular cell adhesion molecule-like protein	14.73	1.29	2.44	0.01	0.03
100194800	tieg3	Transforming growth factor-beta-inducible early growth response	267.37	1.28	2.44	0.00	0.00
106561509	LOC106561509	E3 ubiquitin/ISG15 ligase TRIM25-like	87.99	1.28	2.43	0.00	0.00
106585878	LOC106585878	C-C motif chemokine 19-like	55.63	1.28	2.43	0.01	0.04
106575350	LOC106575350	zinc finger protein 239-like	3.69	1.28	2.43	0.00	0.00
106585958	pdzd2	PDZ domain containing 2	7.98	1.28	2.43	0.00	0.00

106579340	LOC106579340	formin-like protein 1	6.20	1.28	2.43	0.00	0.00
106613068	LOC106613068	uncharacterized LOC106613068	8.68	1.28	2.43	0.00	0.02
100195489	ccr9	C-C chemokine receptor type 9	8.50	1.28	2.43	0.00	0.00
106562199	LOC106562199	myocardial zonula adherens protein-like	13.58	1.28	2.43	0.00	0.00
106608246	LOC106608246	B-cell lymphoma/leukemia 11B-like	8.03	1.28	2.43	0.00	0.00
106586293	klf7	Kruppel like factor 7	12.11	1.28	2.43	0.00	0.00
100380684	LOC100380684	unconventional myosin-If	75.83	1.28	2.43	0.01	0.03
106573914	LOC106573914	arrestin domain-containing protein 2-like	93.32	1.28	2.43	0.00	0.02
106578984	LOC106578984	uncharacterized LOC106578984	17.64	1.28	2.43	0.00	0.02
106575701	LOC106575701	uncharacterized LOC106575701	29.32	1.28	2.43	0.00	0.00
106561158	LOC106561158	proline-serine-threonine phosphatase-interacting protein 1-like	3.09	1.28	2.42	0.00	0.00
106609502	LOC106609502	tubby-related protein 3-like	35.37	1.28	2.42	0.00	0.00
106589789	LOC106589789	structure-specific endonuclease subunit SLX4-like	21.08	1.28	2.42	0.01	0.03
106563748	LOC106563748	mitogen-activated protein kinase kinase kinase kinase 2-like	18.25	1.28	2.42	0.00	0.01
106612860	LOC106612860	RILP-like protein 1	49.13	1.28	2.42	0.00	0.00
106563413	LOC106563413	SH3 domain-binding protein 2-like	12.77	1.28	2.42	0.00	0.00
100380508	zn135	Zinc finger protein 135	30.31	1.28	2.42	0.00	0.00
106584141	LOC106584141	carboxyl-terminal PDZ ligand of neuronal nitric oxide synthase prc	8.97	1.28	2.42	0.00	0.00
106589068	LOC106589068	low density lipoprotein receptor adapter protein 1-A-like	545.24	1.27	2.42	0.00	0.00
106607854	LOC106607854	uncharacterized LOC106607854	108.72	1.27	2.42	0.01	0.03
106606968	LOC106606968	butyrophilin subfamily 1 member A1-like	8.04	1.27	2.42	0.00	0.00
106581235	LOC106581235	dehydrogenase/reductase SDR family member 13-like	101.78	1.27	2.42	0.00	0.00
106560874	LOC106560874	malonyl-CoA decarboxylase, mitochondrial-like	3.83	1.27	2.42	0.00	0.01
106568875	LOC106568875	dual specificity protein phosphatase CDC14A-like	8.93	1.27	2.42	0.00	0.00
106580893	map4k1	mitogen-activated protein kinase kinase kinase kinase 1	26.31	1.27	2.42	0.00	0.00
106572970	LOC106572970	rho guanine nucleotide exchange factor 3-like	15.21	1.27	2.42	0.01	0.04
106612524	LOC106612524	ribonuclease T2-like	487.23	1.27	2.42	0.00	0.00
106568740	LOC106568740	TSC22 domain family protein 2-like	47.56	1.27	2.42	0.00	0.00
106610846	LOC106610846	cysteine-rich motor neuron 1 protein-like	32.29	1.27	2.41	0.00	0.00
100380774	LOC100380774	CD97 antigen	15.43	1.27	2.41	0.00	0.00
106600712	LOC106600712	uncharacterized LOC106600712	6.49	1.27	2.41	0.00	0.00
106599578	LOC106599578	thymocyte selection-associated high mobility group box protein T	7.34	1.27	2.41	0.00	0.01
106607261	LOC106607261	uncharacterized LOC106607261	35.24	1.27	2.41	0.00	0.00
100306796	tmc7	Transmembrane channel-like protein 7	7.52	1.27	2.41	0.00	0.00
106605836	LOC106605836	CD209 antigen-like protein E	7.73	1.27	2.41	0.00	0.00
106600825	LOC106600825	nuclear receptor subfamily 1 group D member 1-like	75.95	1.27	2.41	0.00	0.02
106578827	LOC106578827	transcription factor 20-like	10.41	1.27	2.41	0.00	0.00
106576316	LOC106576316	PRKC apoptosis WT1 regulator protein-like	267.52	1.27	2.41	0.00	0.00
106590081	LOC106590081	succinate--hydroxymethylglutarate CoA-transferase-like	40.14	1.27	2.41	0.00	0.00
106605384	LOC106605384	uncharacterized LOC106605384	152.75	1.27	2.41	0.00	0.00
106565268	LOC106565268	P2Y purinoceptor 1-like	8.99	1.27	2.41	0.00	0.00
106611745	LOC106611745	cysteine-rich hydrophobic domain-containing protein 1-like	7.34	1.27	2.41	0.00	0.00
106565995	LOC106565995	GDH/6PGL endoplasmic bifunctional protein-like	11.72	1.27	2.41	0.00	0.00
106563730	LOC106563730	uncharacterized LOC106563730	763.10	1.27	2.41	0.00	0.00
106612915	LOC106612915	thioredoxin domain-containing protein 17-like	18.46	1.27	2.41	0.00	0.00
106574315	cpb2	carboxypeptidase B2	2245.53	1.27	2.41	0.00	0.00
100196700	tagap	T-cell activation GTPase activating protein	19.21	1.27	2.41	0.00	0.00
106577148	LOC106577148	monocarboxylate transporter 9-like	5.22	1.27	2.40	0.00	0.00
106588581	LOC106588581	protein Jumonji-like	113.16	1.27	2.40	0.00	0.00
106581216	LOC106581216	sialate O-acetylesterase-like	22.35	1.27	2.40	0.00	0.00
106612986	LOC106612986	tumor necrosis factor receptor superfamily member 19-like	39.92	1.27	2.40	0.00	0.00

106603747	LOC106603747	GATS-like protein 2	8.06	1.26	2.40	0.00	0.01
106589731	LOC106589731	integrin alpha-9-like	6.31	1.26	2.40	0.01	0.04
106576221	LOC106576221	carbohydrate sulfotransferase 11	6.20	1.26	2.40	0.00	0.01
106606231	LOC106606231	glucagon receptor-like	9.29	1.26	2.40	0.00	0.00
106561078	LOC106561078	protein phosphatase 1 regulatory subunit 15A-like	30.66	1.26	2.40	0.00	0.00
106583904	LOC106583904	glycerophosphodiester phosphodiesterase domain-containing prc	5.23	1.26	2.40	0.00	0.02
106610706	LOC106610706	antimicrobial peptide NK-lysin-like	9.01	1.26	2.40	0.00	0.00
106610091	LOC106610091	oocyte zinc finger protein XlCOF7.1-like	5.13	1.26	2.40	0.00	0.00
106606352	LOC106606352	uromodulin-like	15.43	1.26	2.40	0.00	0.00
106566754	LOC106566754	peroxisome proliferator-activated receptor gamma-like	8.95	1.26	2.40	0.00	0.00
106566967	LOC106566967	N-acylglucosamine 2-epimerase-like	5.41	1.26	2.40	0.00	0.00
106573961	LOC106573961	angiopoietin-related protein 4-like	29.39	1.26	2.40	0.00	0.00
100136577	LOC100136577	H-2 class II histocompatibility antigen, I-E beta chain	666.11	1.26	2.40	0.00	0.00
106602320	LOC106602320	probable E3 ubiquitin-protein ligase HERC6	121.34	1.26	2.40	0.00	0.00
106585165	LOC106585165	glycerol-3-phosphate acyltransferase 3-like	17.93	1.26	2.39	0.00	0.00
106610175	LOC106610175	transmembrane protein 154-like	7.58	1.26	2.39	0.00	0.00
106576002	adam22	ADAM metallopeptidase domain 22	4.86	1.26	2.39	0.00	0.00
106588086	LOC106588086	integrin alpha-L-like	30.48	1.26	2.39	0.00	0.00
106565858	LOC106565858	calcium/calmodulin-dependent protein kinase type 1-like	27.09	1.26	2.39	0.01	0.03
106589171	LOC106589171	RAS guanyl-releasing protein 1-like	28.26	1.25	2.39	0.00	0.00
106577894	LOC106577894	uncharacterized LOC106577894	28.72	1.25	2.39	0.00	0.01
106602781	LOC106602781	leucine-rich repeats and immunoglobulin-like domains protein 1	36.70	1.25	2.39	0.00	0.01
106589373	slc35g1	NA	3.78	1.25	2.38	0.00	0.00
106583450	LOC106583450	uncharacterized LOC106583450	163.66	1.25	2.38	0.00	0.00
106582276	LOC106582276	alkylglycerol monooxygenase-like	621.32	1.25	2.38	0.00	0.00
100380432	ptn12	Tyrosine-protein phosphatase non-receptor type 12	71.80	1.25	2.38	0.00	0.00
106562340	LOC106562340	transmembrane protein 150C-like	70.10	1.25	2.38	0.00	0.01
106600587	yeats2	NA	9.04	1.25	2.38	0.00	0.00
106564191	LOC106564191	eggshell protein 1-like	3.85	1.25	2.38	0.00	0.00
106611311	LOC106611311	uncharacterized LOC106611311	17.20	1.25	2.38	0.00	0.00
106608238	LOC106608238	cholesterol 24-hydroxylase-like	22.04	1.25	2.38	0.00	0.02
106574739	LOC106574739	probable G-protein coupled receptor 34	10.31	1.25	2.37	0.00	0.00
106588268	LOC106588268	piggyBac transposable element-derived protein 4-like	7.60	1.25	2.37	0.01	0.03
106612683	LOC106612683	delta-like protein C	99.61	1.25	2.37	0.00	0.01
106594578	LOC106594578	perforin-1-like	16.18	1.25	2.37	0.00	0.00
106587313	LOC106587313	E3 ubiquitin-protein ligase TRIM39-like	9.17	1.25	2.37	0.00	0.00
106583433	LOC106583433	interferon-induced protein 44-like	28.67	1.25	2.37	0.00	0.00
106590177	LOC106590177	nuclear receptor-binding protein 2-like	10.48	1.25	2.37	0.00	0.00
106563972	LOC106563972	mitochondrial dynamics protein MID51-like	21.27	1.25	2.37	0.00	0.00
106586020	LOC106586020	fas apoptotic inhibitory molecule 1-like	6.27	1.24	2.37	0.01	0.04
106604806	LOC106604806	uncharacterized LOC106604806	21.00	1.24	2.37	0.00	0.01
106611871	LOC106611871	HLA class II histocompatibility antigen gamma chain-like	827.01	1.24	2.37	0.00	0.02
106610167	LOC106610167	15-hydroxyprostaglandin dehydrogenase [NAD(+)]-like	476.07	1.24	2.37	0.01	0.05
100306856	lgmn	legumain	626.91	1.24	2.37	0.00	0.00
106577352	LOC106577352	A disintegrin and metalloproteinase with thrombospondin motifs	60.45	1.24	2.36	0.01	0.04
106570596	LOC106570596	SLIT-ROBO Rho GTPase-activating protein 3-like	11.19	1.24	2.36	0.01	0.04
106605190	LOC106605190	sialic acid-binding Ig-like lectin 5	4.65	1.24	2.36	0.00	0.00
106566480	LOC106566480	src-like-adapter 2	12.13	1.24	2.36	0.01	0.02
100194992	ripl1	RILP-like protein 1	50.50	1.24	2.36	0.00	0.00
106573758	LOC106573758	choline kinase alpha-like	425.35	1.24	2.36	0.00	0.00
106613396	LOC106613396	trehalase-like	37.65	1.24	2.36	0.00	0.00

106572080	LOC106572080	src-like-adapter 2	33.97	1.24	2.36	0.00	0.00
106603538	LOC106603538	diacylglycerol O-acyltransferase 2-like	1654.82	1.24	2.36	0.00	0.02
106567073	LOC106567073	grainyhead-like protein 1 homolog	14.54	1.24	2.36	0.00	0.01
106583288	LOC106583288	SPRY domain-containing protein 3-like	56.90	1.24	2.36	0.00	0.01
106610290	LOC106610290	protein FAM13A-like	198.69	1.24	2.36	0.00	0.00
106599665	LOC106599665	T-cell receptor beta-1 chain C region-like	31.35	1.24	2.36	0.00	0.00
106609582	LOC106609582	hepatocyte growth factor-like	21.81	1.24	2.36	0.00	0.00
106573022	LOC106573022	reelin-like	4.80	1.24	2.35	0.00	0.00
106604401	LOC106604401	SH2 domain-containing protein 4A-like	15.42	1.23	2.35	0.00	0.00
106604242	LOC106604242	myelin proteolipid protein-like	14.70	1.23	2.35	0.00	0.00
106584897	LOC106584897	calcium/calmodulin-dependent protein kinase type II subunit beta	6.28	1.23	2.35	0.00	0.00
106575562	LOC106575562	T-cell surface antigen CD2-like	10.66	1.23	2.35	0.00	0.00
106582482	LOC106582482	trace amine-associated receptor 13c-like	169.11	1.23	2.35	0.00	0.00
106610718	LOC106610718	zinc finger protein 395-like	17.01	1.23	2.35	0.00	0.00
106577279	LOC106577279	interferon-induced very large GTPase 1-like	43.40	1.23	2.34	0.00	0.00
106582667	LOC106582667	rho GTPase-activating protein 15-like	19.92	1.23	2.34	0.00	0.00
106581806	tbr1	T-box, brain 1	14.92	1.23	2.34	0.00	0.00
106563391	LOC106563391	leucine-rich repeat extensin-like protein 3	10.28	1.23	2.34	0.00	0.00
106612315	LOC106612315	catenin alpha-1-like	10.16	1.23	2.34	0.00	0.00
106609343	LOC106609343	E3 ubiquitin-protein ligase TRIM39-like	10.46	1.23	2.34	0.00	0.00
106607842	LOC106607842	collagen alpha-1(XVI) chain-like	18.91	1.23	2.34	0.01	0.04
106610140	LOC106610140	zinc finger protein 396-like	6.53	1.23	2.34	0.00	0.00
106586498	kiaa0226l	NA	8.27	1.23	2.34	0.00	0.00
106572058	masp2	mannan binding lectin serine peptidase 2	1634.16	1.23	2.34	0.01	0.05
106587052	LOC106587052	uncharacterized LOC106587052	52.10	1.23	2.34	0.00	0.00
100195230	rnd3	Rho-related GTP-binding protein RhoE	39.07	1.23	2.34	0.00	0.00
106587359	LOC106587359	DNA replication complex GINS protein PSF2-like	5.11	1.23	2.34	0.00	0.00
106589316	LOC106589316	uncharacterized LOC106589316	11.32	1.23	2.34	0.00	0.01
106571710	LOC106571710	transcription factor IIIB 90 kDa subunit-like	122.46	1.23	2.34	0.00	0.00
100194722	LOC100194722	uncharacterized LOC100194722	15.88	1.22	2.34	0.00	0.00
106563157	LOC106563157	serine/threonine-protein kinase ULK1-like	37.74	1.22	2.34	0.00	0.00
106604386	dnajc4	DnaJ heat shock protein family (Hsp40) member C4	48.64	1.22	2.33	0.00	0.00
106562247	LOC106562247	creatine kinase U-type, mitochondrial-like	11.05	1.22	2.33	0.00	0.00
106564678	LOC106564678	phosphoinositide 3-kinase regulatory subunit 5-like	8.53	1.22	2.33	0.00	0.01
106589090	LOC106589090	protein L-Myc-1b pseudogene	5.96	1.22	2.33	0.00	0.00
106576064	LOC106576064	cyclin-dependent kinase inhibitor 1-like	82.42	1.22	2.33	0.01	0.03
106566616	LOC106566616	serine/threonine-protein kinase WNK2-like	128.56	1.22	2.33	0.00	0.01
106563563	LOC106563563	transforming growth factor beta-1	7.70	1.22	2.33	0.00	0.02
106565381	LOC106565381	transforming acidic coiled-coil-containing protein 1-like	23.81	1.22	2.33	0.00	0.01
106608480	LOC106608480	circularly permutated Ras protein 1-like	20.68	1.22	2.33	0.00	0.01
106576840	LOC106576840	putative IQ motif and ankyrin repeat domain-containing protein	7.84	1.22	2.33	0.00	0.00
106569989	LOC106569989	uncharacterized LOC106569989	11.77	1.22	2.33	0.00	0.00
106602682	LOC106602682	probable G-protein coupled receptor 83	8.87	1.22	2.33	0.01	0.03
106568752	LOC106568752	eukaryotic initiation factor 4A-II	187.71	1.22	2.33	0.00	0.00
106580076	LOC106580076	calphotin-like	15.28	1.22	2.33	0.00	0.00
106576739	LOC106576739	transient receptor potential cation channel subfamily A member 1	27.68	1.22	2.33	0.00	0.00
106573916	LOC106573916	interferon-induced protein 44-like	7.84	1.22	2.33	0.00	0.00
106611637	LOC106611637	polycomb group RING finger protein 3	64.78	1.22	2.33	0.00	0.01
106561680	LOC106561680	ETS domain-containing protein Elk-3-like	79.92	1.22	2.32	0.00	0.00
106568483	nedd4l	neural precursor cell expressed, developmentally down-regulated	29.08	1.22	2.32	0.01	0.03
100136478	LOC100136478	alpha amylase	2516.87	1.22	2.32	0.00	0.00

106577053	ccser2	coiled-coil serine rich protein 2	21.20	1.22	2.32	0.00	0.00
106563215	LOC106563215	protein unc-13 homolog B-like	40.98	1.22	2.32	0.00	0.00
106564477	LOC106564477	programmed cell death protein 4-like	33.29	1.22	2.32	0.00	0.02
106594136	LOC106594136	gastrula zinc finger protein XlCGF17.1-like	4.85	1.22	2.32	0.00	0.00
100196093	chp2	calcineurin-like EF hand protein 2	92.45	1.22	2.32	0.00	0.00
106562780	LOC106562780	Bardet-Biedl syndrome 4 protein-like	18.83	1.22	2.32	0.00	0.00
106585474	LOC106585474	homeobox protein cut-like 2	9.81	1.22	2.32	0.00	0.00
106563272	LOC106563272	regulator of G-protein signaling 3-like	141.72	1.22	2.32	0.00	0.01
106604844	LOC106604844	H-2 class II histocompatibility antigen gamma chain-like	2611.57	1.21	2.32	0.01	0.04
106564301	LOC106564301	tuberin-like	12.93	1.21	2.32	0.00	0.01
106584645	LOC106584645	inositol hexakisphosphate and diphosphoinositol-pentakisphosph	31.82	1.21	2.32	0.00	0.00
106603827	LOC106603827	NACHT, LRR and PYD domains-containing protein 3-like	35.12	1.21	2.32	0.00	0.00
106581203	LOC106581203	diablo homolog, mitochondrial-like	913.88	1.21	2.32	0.00	0.00
106605346	LOC106605346	probable C-mannosyltransferase DPY19L1	1206.72	1.21	2.32	0.00	0.00
106573908	LOC106573908	pyroglutamyl-peptidase 1-like	11.62	1.21	2.32	0.00	0.00
106602542	LOC106602542	multiple epidermal growth factor-like domains protein 8	19.04	1.21	2.32	0.00	0.01
106571979	LOC106571979	uncharacterized LOC106571979	14.85	1.21	2.31	0.00	0.00
106598137	LOC106598137	uncharacterized LOC106598137	51.01	1.21	2.31	0.00	0.00
106563566	LOC106563566	G-protein coupled receptor 64-like	5.24	1.21	2.31	0.00	0.00
106604205	LOC106604205	coiled-coil domain-containing protein 69-like	43.61	1.21	2.31	0.00	0.00
106587318	LOC106587318	uncharacterized LOC106587318	93.16	1.21	2.31	0.00	0.00
106564996	LOC106564996	C-C chemokine receptor type 7-like	8.85	1.21	2.31	0.00	0.00
106578201	LOC106578201	OTU domain-containing protein 4-like	49.91	1.21	2.31	0.00	0.00
106567173	LOC106567173	uncharacterized LOC106567173	370.44	1.21	2.31	0.00	0.00
100196325	r1441	Probable E3 ubiquitin-protein ligase RNF144A-A	16.38	1.21	2.31	0.00	0.00
106605446	LOC106605446	mothers against decapentaplegic homolog 4-like	7.61	1.21	2.31	0.00	0.00
100194752	kcnk6	potassium two pore domain channel subfamily K member 6	209.93	1.21	2.31	0.00	0.00
106572546	LOC106572546	ras association domain-containing protein 6-like	321.96	1.21	2.31	0.00	0.02
106573426	LOC106573426	NACHT, LRR and PYD domains-containing protein 12-like	27.07	1.21	2.31	0.00	0.00
106577206	exd2	exonuclease 3'-5' domain containing 2	9.50	1.21	2.31	0.00	0.00
106570707	LOC106570707	uncharacterized LOC106570707	95.15	1.21	2.31	0.00	0.00
106600599	LOC106600599	kinesin-1 heavy chain	20.99	1.20	2.31	0.00	0.00
100380843	hdc	histidine decarboxylase	4.44	1.20	2.30	0.00	0.00
106603452	LOC106603452	uncharacterized LOC106603452	10.61	1.20	2.30	0.01	0.03
106582707	LOC106582707	metabotropic glutamate receptor 4-like	13.33	1.20	2.30	0.00	0.00
106579911	LOC106579911	nuclear factor 7, brain-like	13.87	1.20	2.30	0.00	0.00
106610403	LOC106610403	myotubularin-related protein 7-like	110.38	1.20	2.30	0.00	0.00
106576224	LOC106576224	histidine ammonia-lyase-like	130.87	1.20	2.30	0.00	0.00
106560893	LOC106560893	tyrosine aminotransferase-like	12075.73	1.20	2.30	0.00	0.00
106580996	LOC106580996	uncharacterized LOC106580996	19.75	1.20	2.30	0.00	0.02
106590395	LOC106590395	uncharacterized LOC106590395	16.64	1.20	2.30	0.00	0.00
106577637	LOC106577637	olfactory receptor 52B2-like	17.57	1.20	2.30	0.00	0.00
106608943	LOC106608943	chromobox protein homolog 7-like	4.50	1.20	2.29	0.01	0.04
100195626	zfn2b	AN1-type zinc finger protein 2B	79.96	1.20	2.29	0.00	0.01
106567447	LOC106567447	CREB3 regulatory factor-like	46.96	1.20	2.29	0.00	0.02
100286536	cl012	CL012 protein	386.69	1.20	2.29	0.00	0.00
106566993	LOC106566993	differentially expressed in FDCP 6 homolog	12.02	1.20	2.29	0.00	0.00
100194621	adssl2	adenylosuccinate synthase like 2	39.94	1.20	2.29	0.01	0.03
106584164	LOC106584164	zinc finger protein Gfi-1-like	13.31	1.20	2.29	0.00	0.00
100380696	LOC100380696	fibronectin	2332.32	1.20	2.29	0.00	0.00
106591866	LOC106591866	guanine nucleotide-binding protein G(o) subunit alpha-like	15.62	1.20	2.29	0.00	0.00

106566489	LOC106566489	scavenger receptor cysteine-rich type 1 protein M130-like	12.27	1.19	2.29	0.00	0.00
106581502	LOC106581502	uncharacterized LOC106581502	14.56	1.19	2.29	0.00	0.00
106613445	LOC106613445	uncharacterized LOC106613445	54.15	1.19	2.29	0.00	0.00
106571499	LOC106571499	keratinocyte-associated protein 3-like	11.29	1.19	2.29	0.00	0.00
106600934	LOC106600934	uncharacterized LOC106600934	23.08	1.19	2.29	0.00	0.00
106564360	LOC106564360	H-2 class II histocompatibility antigen, A-Q alpha chain-like	12.81	1.19	2.29	0.00	0.01
106579312	skap1	NA	22.47	1.19	2.28	0.00	0.00
106588713	LOC106588713	ADP-ribosylation factor-like protein 4A	5.33	1.19	2.28	0.00	0.00
106613581	LOC106613581	syntaxin-6-like	31.48	1.19	2.28	0.00	0.02
106562284	LOC106562284	interferon regulatory factor 8-like	38.35	1.19	2.28	0.00	0.00
106603318	LOC106603318	GRB2-associated-binding protein 3-like	13.41	1.19	2.28	0.00	0.00
106565537	LOC106565537	receptor tyrosine-protein kinase erbB-3-like	117.01	1.19	2.28	0.00	0.00
106573833	LOC106573833	sphingosine 1-phosphate receptor 4-like	9.99	1.19	2.28	0.00	0.01
106582240	LOC106582240	interferon-induced guanylate-binding protein 1-like	117.62	1.19	2.28	0.00	0.00
106587895	LOC106587895	protein AF-17-like	13.17	1.19	2.28	0.00	0.00
106580770	LOC106580770	uncharacterized LOC106580770	22.16	1.19	2.28	0.00	0.00
106605107	LOC106605107	protein THEMIS2-like	15.66	1.19	2.28	0.00	0.00
106608893	LOC106608893	alpha-actinin-3-like	13.04	1.19	2.28	0.00	0.01
106564256	LOC106564256	calcium-binding mitochondrial carrier protein SCaMC-3 pseudoge	23.37	1.19	2.28	0.00	0.00
106609046	LOC106609046	zinc finger protein 583-like	79.64	1.19	2.28	0.00	0.00
106576448	LOC106576448	T-complex protein 11-like protein 2	85.39	1.19	2.28	0.00	0.00
106590549	LOC106590549	hepatocyte nuclear factor 4-gamma-like	229.10	1.19	2.28	0.00	0.00
106609893	LOC106609893	membrane-spanning 4-domains subfamily A member 12-like	955.96	1.19	2.28	0.00	0.01
106604665	LOC106604665	large neutral amino acids transporter small subunit 3-like	16.61	1.19	2.27	0.00	0.00
106610116	LOC106610116	zinc finger protein 629-like	5.51	1.19	2.27	0.00	0.00
106561621	LOC106561621	spermatid perinuclear RNA-binding protein-like	5.08	1.18	2.27	0.00	0.00
106561517	LOC106561517	uncharacterized LOC106561517	16.22	1.18	2.27	0.00	0.00
106610690	LOC106610690	sorting nexin-14-like	125.65	1.18	2.27	0.00	0.00
106581519	LOC106581519	small G protein signaling modulator 2-like	28.51	1.18	2.27	0.00	0.00
106603753	LOC106603753	protein NLRC3-like	17.31	1.18	2.27	0.00	0.02
106569199	LOC106569199	glutamine synthetase-like	5516.61	1.18	2.27	0.01	0.04
106613647	LOC106613647	AT-rich interactive domain-containing protein 3A-like	98.05	1.18	2.27	0.00	0.00
106575362	LOC106575362	pituitary tumor-transforming gene 1 protein-interacting protein-li	5.56	1.18	2.27	0.00	0.00
106604982	LOC106604982	receptor-type tyrosine-protein phosphatase U-like	22.31	1.18	2.27	0.00	0.00
100136380	cd9	CD9 molecule	63.09	1.18	2.27	0.00	0.00
106580746	LOC106580746	interferon regulatory factor 2-binding protein 1-like	9.67	1.18	2.27	0.00	0.00
106581528	tnfaip1	NA	30.95	1.18	2.27	0.00	0.00
101448051	LOC101448051	uncharacterized LOC101448051	2065.75	1.18	2.27	0.00	0.00
106568006	LOC106568006	G protein-coupled receptor kinase 6-like	7.95	1.18	2.27	0.00	0.00
106592092	LOC106592092	uncharacterized LOC106592092	11.69	1.18	2.27	0.00	0.01
106564667	LOC106564667	uncharacterized LOC106564667	75.07	1.18	2.27	0.00	0.00
106565397	LOC106565397	repressor of RNA polymerase III transcription MAF1 homolog	53.37	1.18	2.27	0.00	0.00
106607821	hivep2	human immunodeficiency virus type I enhancer binding protein 2	7.58	1.18	2.27	0.00	0.00
106582733	LOC106582733	protein phosphatase 1H-like	8.86	1.18	2.26	0.00	0.00
100286452	as3mt	arsenic (+3 oxidation state) methyltransferase	46.78	1.18	2.26	0.00	0.00
106563882	LOC106563882	solute carrier family 46 member 3-like	41.33	1.18	2.26	0.00	0.02
106573724	LOC106573724	UDP-GlcNAc:betaGal beta-1,3-N-acetylglucosaminyltransferase 9-	5.77	1.18	2.26	0.00	0.00
106603480	LOC106603480	fibroblast growth factor receptor 4-like	21.43	1.18	2.26	0.00	0.02
106603289	LOC106603289	oligodendrocyte-myelin glycoprotein-like	21.35	1.18	2.26	0.00	0.01
106601595	LOC106601595	dynamin-2-like	12.74	1.17	2.26	0.00	0.02
106575706	LOC106575706	uncharacterized LOC106575706	43.46	1.17	2.26	0.01	0.03

106560847	LOC106560847	UDP-GlcNAc:betaGal beta-1,3-N-acetylglucosaminyltransferase 9-	8.73	1.17	2.26	0.00	0.00
106574232	LOC106574232	lymphocyte antigen 75-like	29.70	1.17	2.26	0.00	0.01
106584020	LOC106584020	leucine-rich repeat-containing protein 19-like	93.50	1.17	2.25	0.00	0.00
100194908	ub2r2	Ubiquitin-conjugating enzyme E2 R2	45.07	1.17	2.25	0.00	0.00
106578237	LOC106578237	sialic acid-binding Ig-like lectin 16	161.94	1.17	2.25	0.00	0.00
106610250	LOC106610250	TBC1 domain family member 9-like	11.33	1.17	2.25	0.00	0.00
106578417	LOC106578417	cytoskeletal protein Sojo-like	22.32	1.17	2.25	0.00	0.02
106563938	LOC106563938	serine/arginine-rich splicing factor 1B-like	87.82	1.17	2.25	0.00	0.00
106605121	LOC106605121	protein OSCP1-like	10.99	1.17	2.25	0.00	0.00
106572799	LOC106572799	differentially expressed in FDCP 6 homolog	33.56	1.17	2.25	0.00	0.01
100195945	ppip2	Proline-serine-threonine phosphatase-interacting protein 2	4.81	1.17	2.25	0.00	0.00
106587145	LOC106587145	receptor-interacting serine/threonine-protein kinase 2-like	24.34	1.17	2.24	0.00	0.00
106603497	LOC106603497	protein FAM222B-like	6.75	1.17	2.24	0.00	0.00
106574500	LOC106574500	SH2 domain-containing adapter protein F-like	12.26	1.17	2.24	0.00	0.00
106590004	LOC106590004	AT-rich interactive domain-containing protein 4B-like	8.24	1.17	2.24	0.00	0.00
106566966	renbp	NA	34.81	1.17	2.24	0.00	0.01
106577551	LOC106577551	zinc finger and BTB domain-containing protein 38-like	87.62	1.17	2.24	0.01	0.03
106562813	LOC106562813	multidrug resistance-associated protein 9-like	90.33	1.17	2.24	0.01	0.03
106575749	LOC106575749	mannan-binding lectin serine protease 1-like	1435.90	1.16	2.24	0.00	0.00
106588550	LOC106588550	rho guanine nucleotide exchange factor 2-like	7.44	1.16	2.24	0.00	0.00
106583247	LOC106583247	microspherule protein 1-like	45.27	1.16	2.24	0.01	0.05
106582076	LOC106582076	popeye domain-containing protein 3-like	10.61	1.16	2.24	0.00	0.00
106604502	LOC106604502	protocadherin-1-like	177.44	1.16	2.24	0.00	0.00
106584421	crlf1	cytokine receptor like factor 1	5.08	1.16	2.24	0.01	0.03
106561059	LOC106561059	NACHT, LRR and PYD domains-containing protein 12-like	34.40	1.16	2.24	0.00	0.01
100306822	tm56b	Transmembrane protein 56-B	19.42	1.16	2.24	0.00	0.00
106584582	LOC106584582	E3 ubiquitin/ISG15 ligase TRIM25-like	8.68	1.16	2.24	0.00	0.00
106613145	LOC106613145	von Willebrand factor A domain-containing protein 5A-like	7.89	1.16	2.24	0.00	0.00
106572891	LOC106572891	clumping factor A-like	33.00	1.16	2.24	0.01	0.04
106607362	LOC106607362	MKL/myocardin-like protein 2	23.43	1.16	2.23	0.00	0.00
100195038	purb	purine-rich element binding protein B	170.11	1.16	2.23	0.00	0.01
106575296	LOC106575296	dipeptidyl peptidase 4-like	288.18	1.16	2.23	0.00	0.00
106598906	LOC106598906	protein kinase C eta type-like	19.10	1.16	2.23	0.00	0.00
106566057	LOC106566057	guanine nucleotide-binding protein G(s) subunit alpha-like	11.32	1.16	2.23	0.00	0.00
106604056	LOC106604056	large neutral amino acids transporter small subunit 4-like	40.76	1.16	2.23	0.00	0.00
106580500	LOC106580500	phosphatidate cytidylyltransferase 2-like	15.88	1.16	2.23	0.00	0.01
106572356	dlgap4	DLG associated protein 4	5.86	1.16	2.23	0.00	0.01
106581464	LOC106581464	rho guanine nucleotide exchange factor 26-like	10.34	1.16	2.23	0.00	0.00
106607212	LOC106607212	sphingosine 1-phosphate receptor 2-like	31.66	1.16	2.23	0.00	0.00
100195301	ino1a	Inositol-3-phosphate synthase A	155.72	1.16	2.23	0.00	0.00
106560317	LOC106560317	zinc finger protein Gfi-1-like	9.26	1.16	2.23	0.00	0.02
106561328	LOC106561328	ubiquitin-conjugating enzyme E2 H-like	119.45	1.16	2.23	0.00	0.00
106579997	LOC106579997	endoplasmic reticulum mannosyl-oligosaccharide 1,2-alpha-mann	20.08	1.16	2.23	0.00	0.00
106588398	LOC106588398	proteasome subunit beta type-7-like	43.33	1.15	2.23	0.00	0.00
106565723	LOC106565723	bactericidal permeability-increasing protein-like	41.76	1.15	2.22	0.00	0.00
106611531	sash3	SAM and SH3 domain containing 3	59.70	1.15	2.22	0.01	0.03
106574355	LOC106574355	protein NLRC3-like	27.49	1.15	2.22	0.00	0.00
106594163	LOC106594163	cytochrome P450 3A27-like	424.61	1.15	2.22	0.00	0.00
106593332	LOC106593332	cytochrome P450 3A27-like	172.63	1.15	2.22	0.00	0.00
106586999	LOC106586999	G-protein coupled receptor 56-like	21.15	1.15	2.22	0.01	0.04
106568533	onecut2	one cut homeobox 2	11.48	1.15	2.22	0.00	0.00

100196105	rcl1	RNA terminal phosphate cyclase like 1	687.83	1.15	2.22	0.00	0.00
106587573	LOC106587573	arylamine N-acetyltransferase, pineal gland isozyme NAT-10-like	200.52	1.15	2.22	0.00	0.00
106580110	acacb	acetyl-CoA carboxylase beta	20.99	1.15	2.22	0.01	0.03
100196464	cebpd	CCAAT/enhancer binding protein (C/EBP), delta	67.03	1.15	2.22	0.00	0.01
106585824	stpg2	NA	150.82	1.15	2.22	0.01	0.04
106587813	LOC106587813	keratin, type I cytoskeletal 13-like	253.04	1.15	2.22	0.00	0.00
106606839	LOC106606839	cAMP-dependent protein kinase catalytic subunit alpha	95.29	1.15	2.22	0.00	0.00
106582662	LOC106582662	uncharacterized LOC106582662	20.51	1.15	2.22	0.00	0.00
106578930	LOC106578930	NACHT, LRR and PYD domains-containing protein 3-like	10.63	1.15	2.22	0.00	0.02
106608837	LOC106608837	uncharacterized protein DDB_G0290685-like	150.82	1.15	2.22	0.00	0.00
106562046	LOC106562046	adhesion G-protein coupled receptor G5-like	11.69	1.15	2.22	0.00	0.00
106574333	LOC106574333	junctional adhesion molecule B-like	31.55	1.15	2.22	0.00	0.00
106569063	LOC106569063	uncharacterized LOC106569063	18.75	1.15	2.22	0.00	0.00
106567248	LOC106567248	wiskott-Aldrich syndrome protein-like	27.69	1.15	2.21	0.00	0.00
106606357	LOC106606357	b(0,+)-type amino acid transporter 1-like	18.20	1.15	2.21	0.00	0.00
106565075	LOC106565075	nuclear factor of activated T-cells, cytoplasmic 2-like	53.75	1.15	2.21	0.00	0.00
106560813	LOC106560813	choline kinase alpha-like	69.38	1.15	2.21	0.00	0.00
106582200	LOC106582200	GDP-Man:Man(3)GlcNAc(2)-PP-Dol alpha-1,2-mannosyltransferas	24.58	1.15	2.21	0.00	0.02
106594609	LOC106594609	proto-oncogene vav-like	27.67	1.15	2.21	0.00	0.00
106574566	LOC106574566	phospholipase D1-like	89.33	1.14	2.21	0.00	0.00
106587207	LOC106587207	semaphorin-7A-like	7.58	1.14	2.21	0.00	0.00
106586623	LOC106586623	immunoglobulin superfamily member 3-like	33.83	1.14	2.21	0.00	0.00
106575331	LOC106575331	uncharacterized LOC106575331	17.65	1.14	2.21	0.00	0.00
106564585	orai1	ORAI calcium release-activated calcium modulator 1	44.34	1.14	2.21	0.00	0.02
106575735	LOC106575735	uncharacterized LOC106575735	127.46	1.14	2.21	0.00	0.00
106585399	LOC106585399	lipocalin-like	2319.21	1.14	2.21	0.00	0.00
100380863	LOC100380863	SLIT-ROBO Rho GTPase-activating protein 3	24.39	1.14	2.21	0.01	0.03
106587849	LOC106587849	major histocompatibility complex class I-related gene protein-like	6.52	1.14	2.21	0.00	0.00
106610232	LOC106610232	B-cell lymphoma/leukemia 10-like	12.86	1.14	2.21	0.00	0.00
106568171	LOC106568171	ran-binding protein 3-like	42.61	1.14	2.21	0.00	0.00
106603096	chrdl2	chordin like 2	346.27	1.14	2.21	0.00	0.00
106600745	LOC106600745	nuclear protein 1-like	2541.24	1.14	2.21	0.00	0.00
106572294	LOC106572294	phosphatidylinositol 3,4,5-trisphosphate-dependent Rac exchang	22.55	1.14	2.21	0.00	0.02
100195813	pno1	partner of NOB1 homolog	506.25	1.14	2.21	0.00	0.00
106567010	LOC106567010	rho-related GTP-binding protein Rho6-like	8.53	1.14	2.20	0.00	0.00
100195403	ip6k2	inositol hexaphosphate kinase 2	279.91	1.14	2.20	0.00	0.00
106613687	LOC106613687	mucosa-associated lymphoid tissue lymphoma translocation prot	20.69	1.14	2.20	0.00	0.00
106567255	dvl1	dishevelled segment polarity protein 1	15.50	1.14	2.20	0.00	0.00
106561241	LOC106561241	CD9 antigen-like	69.29	1.14	2.20	0.01	0.04
106611722	LOC106611722	rho guanine nucleotide exchange factor 6-like	60.95	1.14	2.20	0.00	0.00
106586577	LOC106586577	CMP-N-acetylneuraminate-beta-galactosamide-alpha-2,3-sialyltra	25.34	1.14	2.20	0.00	0.00
106607272	LOC106607272	F-box/LRR-repeat protein 20	10.04	1.14	2.20	0.00	0.01
106588888	LOC106588888	histone acetyltransferase KAT2B-like	34.92	1.14	2.20	0.01	0.03
106572904	LOC106572904	arginine-glutamic acid dipeptide repeats protein-like	12.24	1.14	2.20	0.00	0.01
106606534	LOC106606534	interleukin-6 receptor subunit beta-like	9.97	1.14	2.20	0.00	0.00
100195516	g137a	Integral membrane protein GPR137	96.75	1.14	2.20	0.00	0.00
106570836	LOC106570836	cytosolic phospholipase A2-like	15.93	1.13	2.20	0.00	0.00
106605206	LOC106605206	ADP-ribosylation factor-like protein 4A	65.13	1.13	2.20	0.01	0.03
106567798	LOC106567798	GRB2-associated-binding protein 3-like	7.98	1.13	2.19	0.00	0.00
106563878	LOC106563878	tripartite motif-containing protein 3-like	44.52	1.13	2.19	0.01	0.03
106601316	LOC106601316	netrin-1-like	5.69	1.13	2.19	0.00	0.01

106611162	LOC106611162	uncharacterized LOC106611162	9.57	1.13	2.19	0.00	0.00
100195232	tco2	Transcobalamin-2	114.04	1.13	2.19	0.00	0.01
106567251	LOC106567251	rap1 GTPase-activating protein 1-like	132.18	1.13	2.19	0.00	0.00
106610484	LOC106610484	dynein heavy chain 6, axonemal-like	15.46	1.13	2.19	0.00	0.01
106589983	LOC106589983	protein NLRC3-like	51.70	1.13	2.19	0.00	0.00
106587948	LOC106587948	tumor protein p53-inducible protein 11-like	9.99	1.13	2.19	0.00	0.00
106562590	LOC106562590	5-formyltetrahydrofolate cyclo-ligase-like	13.52	1.13	2.19	0.00	0.00
106571246	LOC106571246	interactor protein for cytohesin exchange factors 1-like	11.33	1.13	2.19	0.00	0.00
106564129	LOC106564129	uncharacterized LOC106564129	150.22	1.13	2.19	0.01	0.03
106561486	jak2	Janus kinase 2	6.38	1.13	2.19	0.00	0.00
106581262	LOC106581262	uncharacterized LOC106581262	27.56	1.13	2.19	0.00	0.01
106580043	LOC106580043	cyclin-G2-like	57.63	1.13	2.19	0.00	0.01
106563700	bbs1	Bardet-Biedl syndrome 1	46.16	1.13	2.19	0.01	0.04
106611180	LOC106611180	F-box only protein 33-like	120.37	1.13	2.19	0.01	0.03
106577773	LOC106577773	uncharacterized LOC106577773	79.75	1.13	2.18	0.00	0.00
106575925	LOC106575925	BTB/POZ domain-containing protein KCTD12-like	30.87	1.13	2.18	0.00	0.00
106574279	LOC106574279	double-stranded RNA-specific editase 1-like	138.86	1.13	2.18	0.00	0.00
106561809	sergef	NA	6.59	1.13	2.18	0.00	0.00
100136351	raf1	Raf-1 proto-oncogene, serine/threonine kinase	118.70	1.13	2.18	0.00	0.00
106599240	LOC106599240	nucleolar GTP-binding protein 1-like	25.65	1.13	2.18	0.00	0.00
106587398	LOC106587398	cytochrome P450 2D15-like	5371.58	1.13	2.18	0.00	0.02
106574695	LOC106574695	serine/threonine-protein kinase 11-interacting protein-like	9.86	1.13	2.18	0.01	0.03
106562415	LOC106562415	epithelial splicing regulatory protein 2-like	7.79	1.13	2.18	0.00	0.00
106580932	LOC106580932	zinc finger and BTB domain-containing protein 44-like	49.87	1.12	2.18	0.00	0.00
106578066	LOC106578066	protein BTG3-like	38.10	1.12	2.18	0.00	0.00
106572005	LOC106572005	E3 ubiquitin-protein ligase MIB2-like	24.64	1.12	2.18	0.00	0.00
106570183	LOC106570183	protein Jumonji-like	336.02	1.12	2.18	0.00	0.00
106572414	LOC106572414	FERM domain-containing protein 4B-like	40.32	1.12	2.18	0.00	0.00
100380738	LOC100380738	syntaxin-binding protein 2	9.43	1.12	2.18	0.00	0.00
106609639	st8sia1	NA	7.98	1.12	2.18	0.00	0.00
106566298	LOC106566298	E3 ubiquitin-protein ligase UBR4-like	8.08	1.12	2.18	0.00	0.00
106581592	LOC106581592	protein NLRC3-like	15.68	1.12	2.18	0.00	0.00
106563456	LOC106563456	fermitin family homolog 3-like	43.05	1.12	2.18	0.00	0.01
106579805	fbxo21	F-box protein 21	43.55	1.12	2.18	0.00	0.00
106560721	LOC106560721	ankyrin repeat domain-containing protein 26-like	18.36	1.12	2.18	0.00	0.00
106571745	LOC106571745	beta-soluble NSF attachment protein	34.11	1.12	2.18	0.00	0.00
106579213	nlrc3	NLR family CARD domain containing 3	19.14	1.12	2.18	0.00	0.00
106575328	LOC106575328	autophagy-related protein 9A-like	27.36	1.12	2.18	0.00	0.01
106589842	LOC106589842	uncharacterized LOC106589842	47.32	1.12	2.18	0.00	0.00
106576463	LOC106576463	gamma-secretase-activating protein-like	10.86	1.12	2.17	0.01	0.04
106589236	LOC106589236	uncharacterized LOC106589236	26.98	1.12	2.17	0.00	0.00
106604521	aga	aspartylglucosaminidase	112.14	1.12	2.17	0.00	0.00
106602019	LOC106602019	glucagon receptor-like	46.52	1.12	2.17	0.00	0.00
106572797	LOC106572797	uncharacterized LOC106572797	19.05	1.12	2.17	0.00	0.00
106566136	LOC106566136	rho GTPase-activating protein 12-like	24.69	1.12	2.17	0.00	0.02
106611733	LOC106611733	coagulation factor IX-like	3796.40	1.12	2.17	0.00	0.00
106582101	LOC106582101	immunoglobulin superfamily member 3-like	40.82	1.12	2.17	0.01	0.03
106610754	LOC106610754	transcription regulator protein BACH2-like	64.35	1.12	2.17	0.01	0.03
106565027	LOC106565027	CMP-N-acetylneuraminate-beta-galactosamide-alpha-2,3-sialyltra	468.33	1.12	2.17	0.00	0.00
106588224	LOC106588224	programmed cell death 1 ligand 1-like	9.03	1.12	2.17	0.00	0.00
106609962	LOC106609962	plasma protease C1 inhibitor-like	18912.44	1.12	2.17	0.00	0.01

106567764	LOC106567764	dystrophin-related protein 2-like	82.61	1.12	2.17	0.00	0.00
106605778	LOC106605778	uncharacterized LOC106605778	7.77	1.12	2.17	0.00	0.00
106590112	LOC106590112	homeobox protein TGIF1-like	45.39	1.11	2.17	0.00	0.00
106609904	LOC106609904	FACT complex subunit SSRP1-like	27.23	1.11	2.17	0.00	0.00
106605824	LOC106605824	forkhead box protein J2-like	6.76	1.11	2.17	0.00	0.00
106607536	LOC106607536	pleckstrin homology domain-containing family A member 1-like	32.40	1.11	2.16	0.00	0.00
100380359	sox4	SRY-box 4	39.39	1.11	2.16	0.01	0.04
106583950	LOC106583950	insulin receptor-like	13.36	1.11	2.16	0.00	0.01
106577038	LOC106577038	C-terminal-binding protein 2	53.68	1.11	2.16	0.00	0.00
106601108	LOC106601108	uncharacterized LOC106601108	51.62	1.11	2.16	0.00	0.00
100137055	csf3r	granulocyte colony-stimulating factor receptor	39.90	1.11	2.16	0.00	0.00
106581141	LOC106581141	CD166 antigen homolog	292.58	1.11	2.16	0.00	0.00
106569959	LOC106569959	proteasome subunit beta type-6-B like protein	37.71	1.11	2.16	0.00	0.00
106611050	gpatch2l	G-patch domain containing 2 like	31.50	1.11	2.16	0.00	0.00
106567242	LOC106567242	WD repeat-containing protein 13-like	29.20	1.11	2.16	0.00	0.00
100136551	igfbp-2b1	IGF binding protein 3	109.77	1.11	2.16	0.00	0.00
106574033	LOC106574033	cytochrome P450 2J5-like	87.11	1.11	2.16	0.00	0.01
106590536	armc3	armadillo repeat containing 3	340.77	1.11	2.16	0.00	0.00
106612058	LOC106612058	LON peptidase N-terminal domain and RING finger protein 1-like	15.57	1.11	2.16	0.01	0.04
106573037	LOC106573037	HMG box-containing protein 1-like	20.26	1.11	2.16	0.00	0.00
106607884	LOC106607884	protein dispatched homolog 1-like	235.05	1.11	2.16	0.00	0.00
106604650	LOC106604650	myeloid zinc finger 1-like	60.90	1.11	2.16	0.00	0.02
106578188	LOC106578188	type II inositol 3,4-bisphosphate 4-phosphatase-like	20.14	1.11	2.15	0.00	0.01
106578884	LOC106578884	uncharacterized LOC106578884	139.76	1.11	2.15	0.00	0.00
106561633	LOC106561633	uncharacterized LOC106561633	8.16	1.11	2.15	0.00	0.00
106573482	LOC106573482	uncharacterized LOC106573482	814.55	1.11	2.15	0.00	0.00
106588446	LOC106588446	thyrotroph embryonic factor-like	93.21	1.11	2.15	0.00	0.02
106571557	plcb4	NA	17.03	1.11	2.15	0.00	0.00
100196890	calcoco2	calcium binding and coiled-coil domain 2	2573.22	1.11	2.15	0.00	0.00
106603144	LOC106603144	syntaxin-5-like	8.31	1.11	2.15	0.00	0.00
106609056	LOC106609056	ras-related protein Rab-39B	250.43	1.11	2.15	0.00	0.00
106561737	LOC106561737	reelin-like	55.59	1.10	2.15	0.00	0.00
106606073	LOC106606073	metastasis-associated in colon cancer protein 1-like	17.77	1.10	2.15	0.00	0.00
106607516	LOC106607516	zinc finger protein Pegasus-like	68.71	1.10	2.15	0.00	0.01
106604970	LOC106604970	protein-tyrosine kinase 2-beta-like	38.27	1.10	2.15	0.00	0.00
106608048	ubxn2a	NA	27.20	1.10	2.15	0.00	0.00
100196683	LOC100196683	uncharacterized LOC100196683	26.70	1.10	2.15	0.01	0.04
106579334	LOC106579334	uncharacterized LOC106579334	9.29	1.10	2.15	0.00	0.02
106587004	LOC106587004	far upstream element-binding protein 3-like	179.77	1.10	2.15	0.00	0.02
106613898	LOC106613898	homeobox protein Meis1	11.67	1.10	2.15	0.00	0.00
106610675	LOC106610675	heterogeneous nuclear ribonucleoprotein R-like	47.33	1.10	2.14	0.00	0.01
106613854	LOC106613854	oxysterol-binding protein-related protein 1-like	37.33	1.10	2.14	0.00	0.00
106572594	LOC106572594	carboxy-terminal domain RNA polymerase II polypeptide A small p	114.33	1.10	2.14	0.01	0.03
106585598	LOC106585598	anthrax toxin receptor 1-like	176.76	1.10	2.14	0.00	0.00
106612893	stard13	NA	219.07	1.10	2.14	0.00	0.00
106600269	LOC106600269	homeobox and leucine zipper protein Homez-like	17.22	1.10	2.14	0.00	0.00
106610585	LOC106610585	cytoplasmic dynein 1 heavy chain 1-like	240.48	1.10	2.14	0.00	0.00
106602254	LOC106602254	zinc finger protein 2 homolog	42.02	1.10	2.14	0.00	0.00
100195035	abi1	Abl interactor 1	35.99	1.10	2.14	0.00	0.00
106602856	LOC106602856	rho guanine nucleotide exchange factor 15-like	10.58	1.10	2.14	0.01	0.03
106601006	LOC106601006	F-box/LRR-repeat protein 20	109.47	1.09	2.13	0.00	0.01

100195406	grap2	GRB2-related adaptor protein 2	22.17	1.09	2.13	0.01	0.03
106571383	LOC106571383	centrosomal protein of 85 kDa-like	17.10	1.09	2.13	0.00	0.00
106573496	LOC106573496	proline-serine-threonine phosphatase-interacting protein 1-like	32.83	1.09	2.13	0.00	0.00
106579290	LOC106579290	monocarboxylate transporter 6-like	537.35	1.09	2.13	0.00	0.00
106602577	LOC106602577	FACT complex subunit SSRP1-like	127.42	1.09	2.13	0.01	0.05
106578113	LOC106578113	circularly permutated Ras protein 1-like	30.33	1.09	2.13	0.00	0.00
106569259	LOC106569259	dual specificity testis-specific protein kinase 2-like	55.48	1.09	2.13	0.00	0.00
106582359	LOC106582359	uncharacterized LOC106582359	11.57	1.09	2.13	0.00	0.00
106585519	LOC106585519	filamin-interacting protein FAM101A-like	137.58	1.09	2.13	0.00	0.00
106587688	LOC106587688	cell surface glycoprotein 1-like	8.92	1.09	2.13	0.00	0.00
106605438	LOC106605438	semaphorin-4A-like	16.27	1.09	2.13	0.00	0.00
106575387	LOC106575387	zinc finger protein 501-like	9.70	1.09	2.13	0.00	0.01
100136365	LOC100136365	lysozyme C II	154.40	1.09	2.13	0.00	0.00
106578893	LOC106578893	uncharacterized protein KIAA0195-like	15.11	1.09	2.13	0.00	0.01
106573132	LOC106573132	endoplasmin-like	306.80	1.09	2.12	0.00	0.02
106567355	LOC106567355	PTB domain-containing engulfment adapter protein 1-like	93.44	1.09	2.12	0.00	0.01
106562488	LOC106562488	protein FAM214A-like	50.70	1.09	2.12	0.00	0.00
106613141	LOC106613141	amyloid beta A4 precursor protein-binding family B member 1-like	16.43	1.09	2.12	0.00	0.00
106563869	LOC106563869	uncharacterized LOC106563869	68.19	1.09	2.12	0.00	0.02
106563799	LOC106563799	telomerase-binding protein EST1A-like	26.38	1.09	2.12	0.00	0.02
106581257	LOC106581257	mucin-5AC-like	13.78	1.09	2.12	0.00	0.00
106579671	LOC106579671	GON-4-like protein	39.38	1.08	2.12	0.00	0.00
106582251	LOC106582251	phosducin-like protein 3	395.91	1.08	2.12	0.00	0.00
100196887	ef1a	elongation factor 1-alpha	94.57	1.08	2.12	0.01	0.03
106571502	l3hypdh	trans-L-3-hydroxyproline dehydratase	66.43	1.08	2.12	0.00	0.00
100195718	tp8l2	Tumor necrosis factor, alpha-induced protein 8-like protein 2	26.08	1.08	2.12	0.00	0.00
106581031	LOC106581031	vitronectin-like	3361.81	1.08	2.12	0.00	0.00
106603460	LOC106603460	uncharacterized LOC106603460	56.62	1.08	2.12	0.00	0.00
106588300	LOC106588300	zinc finger protein 516-like	153.62	1.08	2.12	0.00	0.00
100306798	blnk	B-cell linker	14.65	1.08	2.12	0.00	0.00
106566166	LOC106566166	nck-associated protein 1-like	36.77	1.08	2.12	0.00	0.00
106608881	LOC106608881	microfibrillar-associated protein 3-like	502.09	1.08	2.12	0.00	0.00
106606997	LOC106606997	SEC14-like protein 1	128.69	1.08	2.11	0.00	0.01
106611700	LOC106611700	protein phosphatase Slingshot homolog 3-like	71.09	1.08	2.11	0.00	0.00
100195203	lcp2	lymphocyte cytosolic protein 2	23.28	1.08	2.11	0.00	0.00
106566780	LOC106566780	cadherin EGF LAG seven-pass G-type receptor 3-like	7.69	1.08	2.11	0.00	0.00
106587077	LOC106587077	phosphoinositide 3-kinase regulatory subunit 5-like	14.45	1.08	2.11	0.00	0.00
106589738	foxn2	forkhead box N2	70.21	1.08	2.11	0.00	0.00
106582243	LOC106582243	uncharacterized LOC106582243	25.82	1.08	2.11	0.00	0.00
106613221	clpb	ClpB homolog, mitochondrial AAA ATPase chaperonin	23.79	1.08	2.11	0.00	0.00
106571242	LOC106571242	serine/threonine-protein kinase D3-like	15.85	1.08	2.11	0.00	0.00
106573888	LOC106573888	FYN-binding protein-like	46.90	1.08	2.11	0.00	0.01
106586260	LOC106586260	NADH-ubiquinone oxidoreductase 75 kDa subunit, mitochondrial-	506.07	1.08	2.11	0.00	0.00
106612744	LOC106612744	uncharacterized LOC106612744	9.70	1.08	2.11	0.01	0.04
106582062	LOC106582062	CD97 antigen-like	32.59	1.08	2.11	0.00	0.00
106607674	LOC106607674	serine/threonine-protein kinase D3-like	14.24	1.08	2.11	0.00	0.01
106612180	LOC106612180	ras-related protein Rab-40B-like	15.17	1.08	2.11	0.00	0.00
106582864	LOC106582864	class E basic helix-loop-helix protein 40-like	245.67	1.07	2.11	0.00	0.00
106576265	LOC106576265	chromodomain-helicase-DNA-binding protein 4-like	245.00	1.07	2.11	0.00	0.00
106565699	LOC106565699	H-2 class II histocompatibility antigen, A-U alpha chain-like	624.06	1.07	2.11	0.00	0.00
100194472	hoxa5aa	homeobox protein HoxA5aa	7.89	1.07	2.10	0.00	0.00

106590453	LOC106590453	receptor-type tyrosine-protein kinase FLT3-like	9.60	1.07	2.10	0.00	0.00
106601170	LOC106601170	F-box only protein 33-like	47.69	1.07	2.10	0.00	0.02
100136407	ahr2b	aryl hydrocarbon receptor 2 beta	53.80	1.07	2.10	0.00	0.00
106602323	LOC106602323	probable E3 ubiquitin-protein ligase HERC3	12.58	1.07	2.10	0.00	0.00
106567802	LOC106567802	skin secretory protein xP2-like	16.01	1.07	2.10	0.01	0.04
106610534	LOC106610534	solute carrier family 43 member 3-like	73.85	1.07	2.10	0.00	0.00
106582115	LOC106582115	nectin-4-like	73.56	1.07	2.10	0.00	0.00
106593877	LOC106593877	G patch domain and ankyrin repeat-containing protein 1-like	17.87	1.07	2.10	0.00	0.01
106578153	LOC106578153	serine/threonine-protein kinase 26-like	365.22	1.07	2.10	0.00	0.00
100194953	irf4	Interferon regulatory factor 4	14.49	1.07	2.10	0.00	0.00
106577625	LOC106577625	multiple PDZ domain protein-like	23.32	1.07	2.10	0.00	0.00
106607174	LOC106607174	MKL/myocardin-like protein 1	27.87	1.07	2.10	0.00	0.01
106591240	LOC106591240	uncharacterized LOC106591240	69.41	1.07	2.10	0.00	0.00
106601467	LOC106601467	ras-related C3 botulinum toxin substrate 2	45.00	1.07	2.10	0.00	0.00
106603106	LOC106603106	monoacylglycerol lipase ABHD12-like	15.82	1.07	2.10	0.00	0.00
106612453	LOC106612453	dedicator of cytokinesis protein 10-like	36.51	1.07	2.10	0.00	0.00
100141317	fxyd5a	FXYD domain containing ion transport regulator 5a	27.57	1.07	2.10	0.00	0.01
106572492	LOC106572492	LETM1 domain-containing protein 1-like	65.07	1.07	2.10	0.00	0.00
100380572	sc6a7	Sodium-dependent proline transporter	14.53	1.07	2.10	0.00	0.01
106608695	LOC106608695	uncharacterized LOC106608695	18.93	1.07	2.10	0.00	0.00
106587026	zmiz2	NA	39.81	1.07	2.09	0.00	0.00
106612259	gm2a	GM2 ganglioside activator	150.49	1.07	2.09	0.00	0.00
100306783	e41l4	Band 4.1-like protein 4	17.53	1.06	2.09	0.01	0.04
106606299	LOC106606299	uncharacterized LOC106606299	53.48	1.06	2.09	0.00	0.00
106586182	LOC106586182	CD302 antigen-like	753.72	1.06	2.09	0.00	0.01
106572228	LOC106572228	putative Polycomb group protein ASXL1	35.49	1.06	2.09	0.00	0.00
100136496	LOC100136496	FIP2-like	20.51	1.06	2.09	0.00	0.01
106601564	hsd3b7	hydroxy-delta-5-steroid dehydrogenase, 3 beta- and steroid delta	57.88	1.06	2.09	0.01	0.04
106604935	LOC106604935	C-X-C motif chemokine 14-like	96.93	1.06	2.09	0.00	0.00
106571305	LOC106571305	ribonuclease ZC3H12A-like	26.88	1.06	2.09	0.00	0.00
106580619	LOC106580619	selenoprotein Pa-like	360.13	1.06	2.09	0.00	0.00
100195321	arsk	arylsulfatase family member K	59.78	1.06	2.09	0.00	0.00
106604997	LOC106604997	uncharacterized LOC106604997	118.89	1.06	2.08	0.01	0.04
106573761	LOC106573761	uncharacterized LOC106573761	15.33	1.06	2.08	0.01	0.03
106590416	LOC106590416	ras-responsive element-binding protein 1-like	17.61	1.06	2.08	0.00	0.00
106605712	LOC106605712	NXPE family member 3-like	201.86	1.06	2.08	0.00	0.00
100195470	hmgb3	High mobility group protein B3	185.62	1.06	2.08	0.00	0.01
106569076	LOC106569076	uncharacterized LOC106569076	33.90	1.06	2.08	0.00	0.00
106602718	ccdc149	coiled-coil domain containing 149	51.08	1.06	2.08	0.00	0.00
106604267	LOC106604267	androgen receptor-like	148.40	1.06	2.08	0.00	0.00
106598932	LOC106598932	gelsolin-like	220.14	1.06	2.08	0.00	0.00
106577197	LOC106577197	catechol O-methyltransferase domain-containing protein 1-like	290.52	1.06	2.08	0.01	0.05
106579830	LOC106579830	triple functional domain protein-like	30.87	1.06	2.08	0.00	0.00
106601029	LOC106601029	serine/threonine-protein kinase 17A-like	76.68	1.05	2.08	0.00	0.02
100380388	syk	spleen associated tyrosine kinase	93.08	1.05	2.08	0.00	0.00
106577024	bnip3	BCL2 interacting protein 3	103.70	1.05	2.07	0.00	0.01
106589616	csnk1d	casein kinase 1 delta	111.44	1.05	2.07	0.01	0.03
106574362	LOC106574362	secreted phosphoprotein 24-like	93.59	1.05	2.07	0.00	0.00
106581934	LOC106581934	protein FAM171B-like	46.64	1.05	2.07	0.00	0.00
106582458	LOC106582458	INO80 complex subunit D-like	111.97	1.05	2.07	0.00	0.00
106605077	LOC106605077	zinc finger protein 407-like	83.65	1.05	2.07	0.00	0.00

106603421	LOC106603421	BRCA1-A complex subunit RAP80-like	35.54	1.05	2.07	0.00	0.00
106605328	LOC106605328	cathepsin S-like	141.91	1.05	2.07	0.00	0.00
106561329	LOC106561329	uncharacterized LOC106561329	12.89	1.05	2.07	0.00	0.00
106601678	LOC106601678	Ig mu chain C region membrane-bound form-like	29.45	1.05	2.07	0.00	0.00
106561808	LOC106561808	anoctamin-5-like	41.83	1.05	2.07	0.00	0.00
106568602	LOC106568602	disabled homolog 2-interacting protein-like	26.58	1.05	2.07	0.00	0.00
106563750	LOC106563750	reticulon-3-like	28.26	1.05	2.07	0.00	0.01
106590041	LOC106590041	rho GTPase-activating protein 12-like	89.73	1.05	2.07	0.00	0.00
106579791	cldn1	claudin 1	222.67	1.05	2.07	0.00	0.00
106574481	LOC106574481	guanine nucleotide-binding protein G(I)/G(S)/G(O) subunit gamm	16.03	1.05	2.07	0.00	0.00
106610990	LOC106610990	major surface trophozoite antigen 11-like	35.53	1.05	2.07	0.00	0.00
106612954	LOC106612954	serine/threonine-protein kinase ULK2-like	92.08	1.05	2.07	0.00	0.00
106605858	LOC106605858	DNA-binding death effector domain-containing protein 2-like	125.88	1.05	2.07	0.00	0.01
106601088	LOC106601088	cell death-inducing p53-target protein 1-like	70.96	1.05	2.07	0.00	0.00
106607916	LOC106607916	neuromedin-B receptor-like	17.78	1.05	2.07	0.01	0.03
106611349	LOC106611349	sodium-dependent multivitamin transporter-like	384.36	1.05	2.07	0.00	0.00
106589725	LOC106589725	tyrosine-protein kinase Mer-like	242.33	1.05	2.06	0.00	0.01
100195217	ppm1h	protein phosphatase, Mg2+/Mn2+ dependent, 1H	10.99	1.04	2.06	0.00	0.00
106586515	akap11	A-kinase anchoring protein 11	88.00	1.04	2.06	0.00	0.01
100195547	cd53	Leukocyte surface antigen CD53	91.25	1.04	2.06	0.00	0.00
106605606	LOC106605606	maternal B9.15 protein-like	42.01	1.04	2.06	0.00	0.00
106570424	hsf1	heat shock transcription factor 1	636.39	1.04	2.06	0.01	0.04
106564108	LOC106564108	bifunctional apoptosis regulator-like	117.28	1.04	2.06	0.00	0.00
106612862	bcas3	BCAS3, microtubule associated cell migration factor	100.80	1.04	2.06	0.00	0.00
106569337	LOC106569337	synaptosomal-associated protein 47-like	11.48	1.04	2.06	0.00	0.00
106611635	LOC106611635	major facilitator superfamily domain-containing protein 7-like	36.73	1.04	2.06	0.00	0.00
106589417	LOC106589417	neuronal-specific septin-3-like	44.03	1.04	2.06	0.00	0.00
106601947	cog1	component of oligomeric golgi complex 1	69.03	1.04	2.05	0.00	0.00
100195400	ds22a	Dual specificity protein phosphatase 22-A	12.28	1.04	2.05	0.00	0.00
106592646	LOC106592646	GTPase IMAP family member 4-like	26.15	1.04	2.05	0.00	0.00
106587890	LOC106587890	U2 small nuclear ribonucleoprotein A'-like	19.85	1.04	2.05	0.00	0.00
106602980	LOC106602980	complement factor B-like	2910.25	1.04	2.05	0.00	0.00
106589602	wipi1	NA	102.33	1.04	2.05	0.00	0.00
106613844	LOC106613844	A disintegrin and metalloproteinase with thrombospondin motifs	29.36	1.04	2.05	0.00	0.01
100194635	4ebp	eukaryotic translation initiation factor 4E binding protein 3-2	162.85	1.04	2.05	0.01	0.03
106603899	LOC106603899	lymphocyte cytosolic protein 2-like	53.39	1.03	2.05	0.00	0.01
106583546	LOC106583546	uncharacterized LOC106583546	32.88	1.03	2.05	0.00	0.00
106604833	LOC106604833	A disintegrin and metalloproteinase with thrombospondin motifs	24.02	1.03	2.05	0.00	0.01
106608356	slc2a12	NA	82.37	1.03	2.05	0.00	0.00
106607308	LOC106607308	C1q-related factor-like	94.49	1.03	2.05	0.00	0.00
106583779	LOC106583779	phosphatidate cytidylyltransferase, mitochondrial-like	9.84	1.03	2.05	0.00	0.01
106586460	LOC106586460	SEC14 domain and spectrin repeat-containing protein 1-like	37.64	1.03	2.05	0.00	0.01
106607758	lama2	laminin subunit alpha 2	50.78	1.03	2.05	0.01	0.04
106604870	LOC106604870	cytoplasmic polyadenylation element-binding protein 3-like	82.55	1.03	2.05	0.01	0.05
100195218	pnrc2	proline-rich nuclear receptor coactivator 2	349.49	1.03	2.05	0.00	0.00
106560328	LOC106560328	uncharacterized LOC106560328	205.56	1.03	2.05	0.00	0.02
106608834	LOC106608834	sal-like protein 4	31.90	1.03	2.05	0.00	0.00
106563758	LOC106563758	interleukin-7 receptor subunit alpha-like	10.87	1.03	2.04	0.00	0.00
106583327	LOC106583327	repressor of RNA polymerase III transcription MAF1 homolog	96.71	1.03	2.04	0.00	0.00
106593341	LOC106593341	cytochrome P450 3A27-like	284.63	1.03	2.04	0.00	0.01
106587693	LOC106587693	selenocysteine insertion sequence-binding protein 2-like	10.54	1.03	2.04	0.01	0.03

106607353	LOC106607353	3-phosphoinositide-dependent protein kinase 1-like	20.17	1.03	2.04	0.00	0.01
106577944	LOC106577944	uncharacterized LOC106577944	25.35	1.03	2.04	0.00	0.00
106585891	LOC106585891	iporin-like	21.79	1.03	2.04	0.00	0.02
106610380	LOC106610380	zinc finger and SCAN domain-containing protein 21-like	70.21	1.03	2.04	0.00	0.00
106581305	LOC106581305	solute carrier family 25 member 36-A-like	78.52	1.03	2.04	0.00	0.00
100136378	LOC100136378	nuclear receptor subfamily 1 group D member 2	59.02	1.03	2.04	0.00	0.00
106576014	LOC106576014	histone-lysine N-methyltransferase 2E-like	22.91	1.03	2.04	0.00	0.00
106612599	LOC106612599	mitogen-activated protein kinase kinase kinase kinase 5-like	78.61	1.03	2.04	0.00	0.00
106574834	LOC106574834	transmembrane and coiled-coil domain-containing protein 3-like	29.84	1.03	2.04	0.00	0.00
100195225	tnfaip2	TNF alpha induced protein 2	32.31	1.03	2.04	0.00	0.01
106560797	LOC106560797	cathepsin L1-like	28.93	1.03	2.04	0.01	0.04
106609272	prkcq	NA	10.84	1.03	2.04	0.00	0.01
100195050	plbl1	phospholipase B-like 1	109.07	1.03	2.04	0.00	0.00
106604312	LOC106604312	ankyrin repeat and KH domain-containing protein 1-like	57.04	1.03	2.04	0.00	0.00
106565098	LOC106565098	uncharacterized LOC106565098	34.80	1.03	2.04	0.00	0.00
106561185	LOC106561185	uncharacterized LOC106561185	31.51	1.03	2.04	0.00	0.01
106572567	LOC106572567	zinc finger and BTB domain-containing protein 48-like	20.97	1.02	2.03	0.00	0.00
106585272	LOC106585272	membrane-associated phosphatidylinositol transfer protein 2-like	14.61	1.02	2.03	0.00	0.02
106589545	LOC106589545	transcription factor MafF-like	141.69	1.02	2.03	0.01	0.05
106601728	LOC106601728	C-Jun-amino-terminal kinase-interacting protein 4-like	16.63	1.02	2.03	0.01	0.04
106600494	LOC106600494	methionine aminopeptidase 1	583.33	1.02	2.03	0.00	0.01
106610679	LOC106610679	nuclear receptor coactivator 7-like	13.94	1.02	2.03	0.00	0.00
106599325	LOC106599325	retinol dehydrogenase 10-B-like	19.02	1.02	2.03	0.00	0.00
100195943	rnt2	Ribonuclease T2	30.11	1.02	2.03	0.00	0.00
106561918	LOC106561918	neuron navigator 2-like	129.03	1.02	2.03	0.01	0.04
106589949	LOC106589949	calcium uniporter protein, mitochondrial-like	12.85	1.02	2.03	0.00	0.00
106606828	LOC106606828	lipid phosphate phosphohydrolase 3-like	272.42	1.02	2.03	0.00	0.00
106586501	LOC106586501	WD repeat and FYVE domain-containing protein 2-like	88.23	1.02	2.03	0.00	0.00
106606040	LOC106606040	histone-lysine N-methyltransferase SETD2-like	18.22	1.02	2.03	0.00	0.00
106569420	LOC106569420	rho GTPase-activating protein 30-like	27.04	1.02	2.03	0.00	0.00
106586145	LOC106586145	signal transducer and activator of transcription 4-like	13.24	1.02	2.03	0.00	0.01
100136932	p38a	mitogen activated protein kinase p38a	112.08	1.02	2.03	0.00	0.01
100195402	txnip	thioredoxin interacting protein	339.75	1.02	2.03	0.00	0.02
106580462	LOC106580462	tyrosyl-DNA phosphodiesterase 2-like	29.84	1.02	2.03	0.00	0.00
106603363	LOC106603363	myelin protein zero-like protein 2	43.91	1.02	2.03	0.00	0.00
106613095	LOC106613095	galectin-9B-like	591.53	1.02	2.03	0.00	0.00
100195235	acd10	Acyl-CoA dehydrogenase family member 10	54.25	1.02	2.03	0.00	0.00
106613902	LOC106613902	calreticulin-like	508.46	1.02	2.03	0.00	0.00
106611173	LOC106611173	ral GTPase-activating protein subunit alpha-1-like	324.36	1.02	2.03	0.00	0.00
106567154	LOC106567154	round spermatid basic protein 1-like	31.69	1.02	2.02	0.00	0.00
106601095	LOC106601095	insulin-like growth factor-binding protein complex acid labile subu	1988.47	1.02	2.02	0.00	0.00
106608585	LOC106608585	echinoderm microtubule-associated protein-like 3	21.39	1.02	2.02	0.00	0.02
106590198	LOC106590198	E3 ubiquitin/ISG15 ligase TRIM25-like	15.65	1.02	2.02	0.00	0.00
106576798	LOC106576798	kynurenine 3-monooxygenase-like	2285.18	1.02	2.02	0.00	0.00
106571205	tagap	NA	19.99	1.02	2.02	0.00	0.00
100195198	vps37a	VPS37A, ESCRT-I subunit	277.05	1.02	2.02	0.00	0.00
106592151	LOC106592151	uncharacterized LOC106592151	83.67	1.01	2.02	0.00	0.00
106576260	LOC106576260	transcription factor ETV6-like	36.17	1.01	2.02	0.00	0.00
106612870	LOC106612870	complement C4-like	7012.70	1.01	2.02	0.00	0.00
106562835	LOC106562835	probable palmitoyltransferase ZDHHC12	18.33	1.01	2.02	0.00	0.00
106613552	LOC106613552	SUN domain-containing protein 2-like	16.47	1.01	2.02	0.00	0.01

106576519	LOC106576519	zinc finger protein 569-like	10.67	1.01	2.02	0.00	0.01
100286559	LOC100286559	proline-rich protein 5	32.65	1.01	2.02	0.00	0.00
106576807	LOC106576807	DNA-binding protein Ikaros-like	9.19	1.01	2.01	0.00	0.00
106600889	LOC106600889	26S protease regulatory subunit 10B-like	28.99	1.01	2.01	0.01	0.04
100195389	aprin	Androgen-induced proliferation inhibitor	11.59	1.01	2.01	0.00	0.00
106563097	LOC106563097	dimethylglycine dehydrogenase, mitochondrial-like	122.19	1.01	2.01	0.00	0.00
100136432	LOC100136432	prion protein	159.75	1.01	2.01	0.00	0.02
106574474	LOC106574474	tetraspanin-3-like	14.75	1.01	2.01	0.00	0.00
106589680	tnfaip3	NA	33.72	1.01	2.01	0.00	0.00
106609508	LOC106609508	extensin-like	14.95	1.01	2.01	0.00	0.00
106580806	LOC106580806	limbic system-associated membrane protein-like	22.21	1.01	2.01	0.00	0.00
106573447	LOC106573447	hepatocyte growth factor-like	31.76	1.01	2.01	0.01	0.04
106560984	LOC106560984	SRSF protein kinase 2-like	18.32	1.01	2.01	0.00	0.01
100380789	pcat2	Lysophosphatidylcholine acyltransferase 2	68.87	1.01	2.01	0.00	0.00
106601364	LOC106601364	ras-related protein Rab-3D-like	20.34	1.01	2.01	0.00	0.00
106588746	LOC106588746	uncharacterized LOC106588746	25.28	1.00	2.01	0.01	0.03
106603569	LOC106603569	solute carrier organic anion transporter family member 2B1-like	61.18	1.00	2.01	0.01	0.03
106605765	LOC106605765	uncharacterized LOC106605765	21.93	1.00	2.01	0.00	0.00
106610330	arhgef38	Rho guanine nucleotide exchange factor 38	47.32	1.00	2.01	0.00	0.00
106612385	LOC106612385	suppressor of cytokine signaling 3-like	48.18	1.00	2.01	0.00	0.01
106602838	asl	argininosuccinate lyase	95.39	1.00	2.01	0.00	0.00
106580587	LOC106580587	FYN-binding protein-like	33.21	1.00	2.01	0.01	0.03
106602264	LOC106602264	beclin 1-associated autophagy-related key regulator-like	29.99	1.00	2.01	0.01	0.03
106573699	LOC106573699	PQ-loop repeat-containing protein 1-like	177.02	1.00	2.00	0.00	0.00
106587138	LOC106587138	two pore calcium channel protein 2-like	62.60	1.00	2.00	0.00	0.01
106590297	LOC106590297	ras-related C3 botulinum toxin substrate 2-like	35.32	1.00	2.00	0.00	0.00
106570309	LOC106570309	glucocorticoid-induced transcript 1 protein-like	155.21	1.00	2.00	0.00	0.00
106580099	ankrd13a	ankyrin repeat domain 13A	10.90	1.00	2.00	0.00	0.00
106561892	LOC106561892	glycoprotein endo-alpha-1,2-mannosidase-like	107.79	1.00	2.00	0.00	0.00
106572561	LOC106572561	uncharacterized LOC106572561	13.71	1.00	2.00	0.00	0.02
100196725	cytip	cytohesin 1 interacting protein	16.07	1.00	2.00	0.00	0.00
106577845	tesk1	NA	159.74	1.00	2.00	0.00	0.01
106570858	LOC106570858	iporin-like	16.08	1.00	2.00	0.01	0.03
106586760	LOC106586760	lysosome-associated membrane glycoprotein 1-like	533.46	1.00	2.00	0.00	0.00
106606027	LOC106606027	magnesium transporter MRS2 homolog, mitochondrial-like	29.99	1.00	2.00	0.00	0.00

Down-regulated DEGs in fry compared to parr

NCBI ID	Gene locus	Gene name	baseMean	log2FoldChange	Fold change	pvalue	padj
100195420	pck1	phosphoenolpyruvate carboxykinase 1	58.67	-4.62	-24.65	0.00	0.00
106587343	LOC106587343	hepatic triacylglycerol lipase-like	165.94	-4.28	-19.41	0.00	0.00
106611136	LOC106611136	ornithine decarboxylase 1-like	441.79	-3.47	-11.07	0.00	0.00
100380757	pfkfb3	6-phosphofructo-2-kinase/fructose-2,6-biphosphatase 3	41.70	-3.05	-8.27	0.00	0.00
106568300	LOC106568300	group 3 secretory phospholipase A2-like	12.30	-2.91	-7.49	0.01	0.03
106603905	LOC106603905	uncharacterized LOC106603905	17.54	-2.81	-7.03	0.00	0.01
106599162	LOC106599162	cholesterol 7-alpha-monooxygenase-like	1505.37	-2.78	-6.88	0.00	0.00
106577211	LOC106577211	DNA damage-inducible transcript 4 protein-like	99.26	-2.64	-6.22	0.00	0.00
100195506	lipe	lipase, hormone-sensitive	351.59	-2.62	-6.13	0.00	0.00
106568163	ppp1r3b	NA	10.84	-2.47	-5.53	0.00	0.00
106603085	LOC106603085	type II iodothyronine deiodinase-like	347.56	-2.42	-5.36	0.00	0.00
106573544	LOC106573544	lipoprotein lipase-like	9.05	-2.42	-5.35	0.00	0.01
106580452	LOC106580452	purpurin-like	65.22	-2.37	-5.16	0.01	0.04
106577852	LOC106577852	vang-like protein 2	8.60	-2.37	-5.15	0.00	0.00

106608500	LOC106608500	acyl-CoA desaturase-like	1354.24	-2.34	-5.08	0.01	0.04
106584567	LOC106584567	acyl-coenzyme A thioesterase 4-like	74.20	-2.34	-5.07	0.00	0.00
106564655	LOC106564655	transcription elongation factor, mitochondrial-like	23.33	-2.32	-4.99	0.00	0.00
106609479	LOC106609479	small nuclear ribonucleoprotein F	63.52	-2.31	-4.97	0.00	0.00
106585202	LOC106585202	succinyl-CoA:3-ketoacid coenzyme A transferase 1, mitochondrial	15.81	-2.31	-4.95	0.00	0.01
106582467	LOC106582467	cytochrome c	757.81	-2.30	-4.92	0.00	0.01
106579188	LOC106579188	SPRY domain-containing SOCS box protein 3-like	8.80	-2.28	-4.87	0.01	0.03
106571601	LOC106571601	type III iodothyronine deiodinase-like	288.97	-2.28	-4.84	0.00	0.00
106603912	LOC106603912	gastrotropin-like	210.18	-2.22	-4.67	0.00	0.01
106576293	LOC106576293	small nuclear ribonucleoprotein F-like	84.12	-2.22	-4.66	0.00	0.00
106590449	LOC106590449	transcription factor IIIA-like	5.37	-2.21	-4.61	0.00	0.00
106605929	LOC106605929	uncharacterized LOC106605929	16.43	-2.19	-4.57	0.00	0.01
106602288	LOC106602288	integrator complex subunit 12-like	35.26	-2.18	-4.52	0.01	0.03
106584915	LOC106584915	adenosine monophosphate-protein transferase FICD pseudogene	15.86	-2.17	-4.50	0.00	0.00
106586588	tmem37	NA	27.12	-2.15	-4.45	0.00	0.02
106600595	odc1	ornithine decarboxylase 1	674.47	-2.15	-4.44	0.00	0.01
106583118	LOC106583118	uncharacterized LOC106583118	4.39	-2.14	-4.41	0.00	0.01
106562806	LOC106562806	CCAAT/enhancer-binding protein alpha-like	552.53	-2.13	-4.38	0.00	0.00
106577779	LOC106577779	uncharacterized LOC106577779	4.57	-2.13	-4.37	0.00	0.00
106612704	LOC106612704	SERTA domain-containing protein 3-like	29.37	-2.11	-4.33	0.00	0.02
106564257	LOC106564257	proline dehydrogenase 1, mitochondrial-like	38.89	-2.11	-4.31	0.01	0.03
106604690	mgarp	mitochondria localized glutamic acid rich protein	18.93	-2.10	-4.30	0.00	0.00
106585173	LOC106585173	BAG family molecular chaperone regulator 4-like	51.18	-2.10	-4.29	0.00	0.00
106589612	LOC106589612	fatty acid synthase-like	19105.04	-2.09	-4.27	0.00	0.00
106612829	LOC106612829	phosphoserine phosphatase-like	362.98	-2.09	-4.27	0.00	0.00
106571818	slc27a6	NA	371.58	-2.09	-4.27	0.00	0.00
106584298	LOC106584298	polymeric immunoglobulin receptor-like	26.68	-2.07	-4.21	0.00	0.00
106569234	LOC106569234	protein canopy-1-like	13.75	-2.06	-4.17	0.00	0.00
106610271	LOC106610271	fatty acid synthase-like	3898.02	-2.06	-4.16	0.00	0.00
106586467	LOC106586467	protein phosphatase 1 regulatory subunit 1C-like	3.84	-2.05	-4.14	0.00	0.00
100380717	LOC100380717	heat shock 70 kDa protein 4	88.56	-2.04	-4.12	0.00	0.00
106567090	LOC106567090	tubulin alpha chain	37.20	-2.04	-4.10	0.00	0.00
106588409	LOC106588409	tubulin beta chain	1611.21	-2.01	-4.03	0.00	0.00
106578532	idi1	isopentenyl-diphosphate delta isomerase 1	2729.08	-2.01	-4.03	0.01	0.05
106571107	LOC106571107	coiled-coil domain-containing protein 85C-B-like	5.94	-1.99	-3.98	0.00	0.00
106562381	LOC106562381	arylamine N-acetyltransferase, pineal gland isozyme NAT-10-like	11.57	-1.99	-3.97	0.00	0.01
106611710	LOC106611710	mid1-interacting protein 1-like	267.37	-1.99	-3.97	0.00	0.02
100195204	s2533	Solute carrier family 25 member 33	122.18	-1.98	-3.93	0.00	0.01
106563212	LOC106563212	iporin-like	3.38	-1.98	-3.93	0.00	0.00
106580450	LOC106580450	uncharacterized LOC106580450	111.75	-1.97	-3.93	0.00	0.00
106585451	LOC106585451	calcium-binding mitochondrial carrier protein SCaMC-2-A-like	18.05	-1.95	-3.85	0.00	0.00
106594932	LOC106594932	piggyBac transposable element-derived protein 4-like	19.21	-1.94	-3.84	0.00	0.00
106590033	LOC106590033	ATP synthase F(0) complex subunit C3, mitochondrial-like	997.60	-1.94	-3.82	0.01	0.05
106561090	LOC106561090	keratin-associated protein 16-1-like	55.93	-1.93	-3.81	0.01	0.05
106598941	pif1	NA	12.13	-1.93	-3.81	0.00	0.00
106600865	LOC106600865	interferon a3	2.75	-1.93	-3.81	0.01	0.04
106591002	LOC106591002	probable ergosterol biosynthetic protein 28	379.81	-1.92	-3.78	0.00	0.01
106612580	LOC106612580	frizzled-9-like	2.71	-1.92	-3.78	0.00	0.00
106588577	LOC106588577	type-4 ice-structuring protein-like	57.76	-1.92	-3.78	0.00	0.00
106568047	LOC106568047	uromodulin-like 1	7.74	-1.91	-3.77	0.00	0.00
106613839	LOC106613839	long-chain-fatty-acid--CoA ligase ACSBG2-like	96.38	-1.91	-3.76	0.00	0.01

106603080	LOC106603080	uncharacterized LOC106603080	6.23	-1.91	-3.75	0.00	0.01
106565226	LOC106565226	keratin, type II cytoskeletal 8-like	3.11	-1.91	-3.75	0.00	0.02
106576547	LOC106576547	dnaJ homolog subfamily C member 2-like	23.90	-1.90	-3.74	0.00	0.00
106599548	LOC106599548	transcription cofactor HES-6-like	2.58	-1.90	-3.73	0.00	0.02
106585189	LOC106585189	uncharacterized LOC106585189	2.95	-1.90	-3.72	0.00	0.00
106564540	LOC106564540	uncharacterized LOC106564540	6.03	-1.89	-3.72	0.00	0.00
106590084	LOC106590084	cell death activator CIDE-A-like	41.95	-1.89	-3.71	0.00	0.00
106584447	LOC106584447	protein QIL1-like	235.03	-1.88	-3.69	0.01	0.04
106585583	LOC106585583	complement C1q-like protein 4	200.58	-1.87	-3.67	0.00	0.00
106565809	LOC106565809	mitochondrial intermembrane space import and assembly protei	37.13	-1.87	-3.65	0.00	0.00
106567641	LOC106567641	heat shock cognate 70 kDa protein-like	71.56	-1.86	-3.63	0.00	0.00
106560267	LOC106560267	ras-like protein family member 11B	64.88	-1.85	-3.61	0.00	0.00
106589124	LOC106589124	tribbles homolog 1-like	16.14	-1.85	-3.61	0.00	0.00
106572521	LOC106572521	bridging integrator 2-like	4.63	-1.84	-3.58	0.00	0.00
106606009	LOC106606009	protein S100-A1-like	59.20	-1.84	-3.58	0.00	0.00
106563557	LOC106563557	uncharacterized LOC106563557	1296.51	-1.83	-3.56	0.00	0.00
106608853	LOC106608853	homeobox and leucine zipper protein Homez-like	3.79	-1.83	-3.55	0.00	0.00
106583508	bora	bora, aurora kinase A activator	26.77	-1.82	-3.54	0.00	0.00
106574078	LOC106574078	SCL-interrupting locus protein homolog	10.12	-1.82	-3.53	0.00	0.00
106604754	LOC106604754	centromere protein I-like	30.01	-1.81	-3.52	0.00	0.00
106569884	LOC106569884	interleukin enhancer-binding factor 2 homolog	298.74	-1.81	-3.51	0.00	0.00
106567948	LOC106567948	diacylglycerol O-acyltransferase 2-like	2184.09	-1.81	-3.49	0.01	0.04
106579552	oplah	5-oxoprolinase (ATP-hydrolysing)	135.67	-1.80	-3.47	0.00	0.00
106579954	rad9b	NA	4.39	-1.79	-3.45	0.00	0.00
106568166	LOC106568166	calcyphosin-like protein	6.73	-1.79	-3.45	0.00	0.01
106578452	LOC106578452	pyruvate dehydrogenase (acetyl-transferring) kinase isozyme 3, m	604.76	-1.79	-3.45	0.00	0.00
106568948	LOC106568948	mpv17-like protein 2	20.38	-1.79	-3.45	0.01	0.03
100196145	pgp	phosphoglycolate phosphatase	242.00	-1.78	-3.43	0.00	0.00
106601015	LOC106601015	ATP-citrate synthase-like	992.02	-1.77	-3.41	0.00	0.02
106565243	LOC106565243	uncharacterized LOC106565243	3.11	-1.77	-3.40	0.01	0.05
106605152	LOC106605152	dehydrodolichyl diphosphate syntase complex subunit DHDDS-like	58.38	-1.76	-3.39	0.00	0.00
106610939	LOC106610939	leucine-rich repeat and immunoglobulin-like domain-containing n	14.93	-1.76	-3.39	0.00	0.00
106562012	LOC106562012	G1/S-specific cyclin-E1-like	66.21	-1.76	-3.39	0.00	0.00
106613715	LOC106613715	mitochondrial import inner membrane translocase subunit TIM44	61.65	-1.76	-3.39	0.00	0.01
106586653	LOC106586653	Na(+)/H(+) exchange regulatory cofactor NHE-RF3-like	3.55	-1.76	-3.38	0.00	0.00
106587253	LOC106587253	putative solute carrier family 22 member 31	34.10	-1.75	-3.38	0.00	0.00
106611889	LOC106611889	uncharacterized LOC106611889	5.61	-1.75	-3.37	0.00	0.02
106604756	LOC106604756	ADP-ribosylation factor-like protein 13B	30.08	-1.75	-3.36	0.00	0.01
106570509	LOC106570509	uncharacterized LOC106570509	2.78	-1.75	-3.36	0.00	0.00
106561298	LOC106561298	zinc finger protein 699-like	13.72	-1.75	-3.35	0.00	0.00
106602501	LOC106602501	zinc finger protein 25-like	2.69	-1.74	-3.35	0.01	0.03
106606883	LOC106606883	glycine-rich cell wall structural protein-like	36.52	-1.74	-3.35	0.00	0.00
106573377	lrrc17	leucine rich repeat containing 17	93.66	-1.74	-3.34	0.01	0.05
106565357	LOC106565357	mitochondrial import receptor subunit TOM6 homolog	435.32	-1.74	-3.34	0.01	0.03
106565839	slc25a26	solute carrier family 25 member 26	176.14	-1.73	-3.32	0.00	0.00
106578480	prmt6	NA	59.26	-1.73	-3.32	0.00	0.02
100195467	erg19	Diphosphomevalonate decarboxylase	349.01	-1.73	-3.32	0.00	0.01
106589569	LOC106589569	zinc finger BED domain-containing protein 1-like	9.71	-1.73	-3.31	0.00	0.00
100195967	ndub6	NADH dehydrogenase 1 beta subcomplex subunit 6	35.57	-1.72	-3.30	0.00	0.00
106569147	dhx30	DExH-box helicase 30	37.03	-1.72	-3.30	0.00	0.02
106610336	papss1	3'-phosphoadenosine 5'-phosphosulfate synthase 1	40.69	-1.72	-3.29	0.00	0.01

106573849	LOC106573849	tubulin alpha chain, testis-specific-like	4.00	-1.72	-3.29	0.00	0.01
106572760	LOC106572760	keratin, type I cytoskeletal 18-like	24.27	-1.71	-3.28	0.00	0.00
106589046	LOC106589046	uncharacterized protein C6orf47-like	392.13	-1.71	-3.27	0.01	0.03
106587299	LOC106587299	SHC SH2 domain-binding protein 1-like	31.42	-1.71	-3.27	0.01	0.04
106580744	LOC106580744	uncharacterized LOC106580744	8.00	-1.71	-3.27	0.00	0.00
106580039	LOC106580039	long-chain-fatty-acid--CoA ligase ACSBG2-like	890.31	-1.71	-3.26	0.00	0.00
106613551	LOC106613551	cytochrome b-c1 complex subunit 6, mitochondrial-like	217.19	-1.70	-3.26	0.00	0.00
106583862	mkrn2os	MKRN2 opposite strand	28.63	-1.70	-3.25	0.00	0.00
106609774	dnajc2	DnaJ heat shock protein family (Hsp40) member C2	98.26	-1.70	-3.25	0.00	0.00
106604765	LOC106604765	tubulin beta-4B chain-like	1129.44	-1.70	-3.25	0.00	0.00
106589937	LOC106589937	uncharacterized LOC106589937	256.66	-1.70	-3.24	0.01	0.04
106608896	LOC106608896	39S ribosomal protein L52, mitochondrial-like	19.23	-1.70	-3.24	0.00	0.00
106612131	cystm1	cysteine rich transmembrane module containing 1	25.73	-1.69	-3.23	0.00	0.00
106582847	LOC106582847	dehydrodolichyl diphosphate syntase complex subunit DHDDS-like	63.52	-1.69	-3.22	0.00	0.01
106589464	cssa28h17orf62	chromosome ssa28 open reading frame, human C17orf62	172.55	-1.69	-3.22	0.00	0.00
106603135	tm7sf2	NA	1607.27	-1.69	-3.22	0.00	0.00
100195726	gsta3	glutathione S-transferase alpha 3	745.19	-1.69	-3.22	0.00	0.00
106611586	elovl6	ELOVL fatty acid elongase 6	9704.81	-1.68	-3.22	0.00	0.00
106570829	hmgcr	3-hydroxy-3-methylglutaryl-CoA reductase	2073.86	-1.68	-3.21	0.00	0.00
100380512	sox11	SRY-box 11	6.01	-1.68	-3.21	0.00	0.02
106582077	LOC106582077	uncharacterized LOC106582077	9.64	-1.68	-3.21	0.01	0.04
100286461	acot13	acyl-CoA thioesterase 13	46.56	-1.68	-3.21	0.01	0.03
106584709	LOC106584709	cytochrome c oxidase subunit 5A, mitochondrial-like	631.00	-1.68	-3.20	0.00	0.01
106612989	LOC106612989	tail-anchored protein insertion receptor WRB-like	48.31	-1.67	-3.19	0.00	0.01
106588754	LOC106588754	cartilage-associated protein-like	462.91	-1.67	-3.19	0.00	0.00
106574319	LOC106574319	tubulin beta-4B chain-like	7.02	-1.67	-3.19	0.00	0.00
100194828	phop2	Pyridoxal phosphate phosphatase PHOSPHO2	9.76	-1.66	-3.17	0.00	0.00
100286659	pmf1	Polyamine-modulated factor 1	58.73	-1.66	-3.16	0.00	0.00
106565089	LOC106565089	zinc finger and SCAN domain-containing protein 2-like	35.41	-1.66	-3.16	0.00	0.00
106580001	LOC106580001	tubulin beta-1 chain	1306.87	-1.66	-3.15	0.00	0.00
106586797	LOC106586797	mediator of RNA polymerase II transcription subunit 14-like	58.89	-1.66	-3.15	0.00	0.00
106609896	LOC106609896	pre-mRNA-processing factor 19	20.02	-1.65	-3.14	0.00	0.00
106571726	LOC106571726	alpha-(1,3)-fucosyltransferase 9-like	54.43	-1.65	-3.14	0.00	0.00
106608511	LOC106608511	protein Wnt-8b	1.89	-1.65	-3.14	0.00	0.00
106589095	LOC106589095	sodium-dependent lysophosphatidylcholine symporter 1-B-like	107.68	-1.65	-3.14	0.00	0.01
106567984	p2rx5	purinergic receptor P2X 5	13.19	-1.65	-3.13	0.00	0.00
106573007	LOC106573007	3-beta-hydroxysteroid-Delta(8),Delta(7)-isomerase-like	240.16	-1.65	-3.13	0.00	0.00
106589557	LOC106589557	hemoglobin subunit alpha-2-like	3.35	-1.64	-3.12	0.00	0.00
106612554	LOC106612554	1-acyl-sn-glycerol-3-phosphate acyltransferase epsilon-like	82.20	-1.64	-3.12	0.00	0.00
100194617	tpi1b	triosephosphate isomerase 1b	855.52	-1.64	-3.12	0.00	0.00
100194920	acat2	acetyl-CoA acetyltransferase 2	1608.67	-1.64	-3.11	0.00	0.00
106605338	LOC106605338	sterol regulatory element-binding protein cleavage-activating pro	669.01	-1.64	-3.11	0.00	0.00
106611892	LOC106611892	zinc finger protein 232-like	14.74	-1.64	-3.11	0.00	0.00
106571947	LOC106571947	glucose-6-phosphate 1-dehydrogenase-like	589.73	-1.63	-3.10	0.00	0.00
106563120	LOC106563120	small nuclear ribonucleoprotein Sm D3	21.28	-1.63	-3.10	0.00	0.00
106564809	LOC106564809	phosphofurin acidic cluster sorting protein 2-like	27.64	-1.63	-3.09	0.00	0.00
100136444	fabp3	fatty acid binding protein 3	31.91	-1.63	-3.09	0.00	0.00
106580945	LOC106580945	multiple epidermal growth factor-like domains protein 6	11.81	-1.63	-3.09	0.00	0.00
106610322	LOC106610322	NADH dehydrogenase [ubiquinone] 1 beta subcomplex subunit 6-	48.51	-1.62	-3.08	0.00	0.00
106603944	LOC106603944	complement component 1 Q subcomponent-binding protein, mit	577.19	-1.62	-3.08	0.00	0.00
106571961	atp5e	ATP synthase, H+ transporting, mitochondrial F1 complex, epsilon	1119.84	-1.62	-3.07	0.00	0.01

106577153	LOC106577153	putative ATP-dependent RNA helicase DHX57	36.14	-1.62	-3.07	0.00	0.02
106564748	LOC106564748	39S ribosomal protein L45, mitochondrial-like	11.34	-1.62	-3.06	0.00	0.00
106585868	LOC106585868	malate dehydrogenase, cytoplasmic-like	1446.05	-1.61	-3.06	0.00	0.00
106581851	lss	lanosterol synthase (2,3-oxidosqualene-lanosterol cyclase)	1862.82	-1.61	-3.06	0.00	0.00
106570299	LOC106570299	endonuclease/exonuclease/phosphatase family domain-containir	827.45	-1.61	-3.06	0.00	0.00
106585061	slc25a1	NA	298.68	-1.61	-3.05	0.00	0.00
106572076	myh7b	myosin heavy chain 7B	9.09	-1.61	-3.05	0.01	0.03
106570209	LOC106570209	lanosterol 14-alpha demethylase-like	798.79	-1.61	-3.05	0.00	0.00
100286631	ube2t	ubiquitin conjugating enzyme E2 T	86.73	-1.61	-3.05	0.00	0.00
100196879	dhfr	dihydrofolate reductase	165.33	-1.61	-3.05	0.00	0.00
106607826	LOC106607826	cyclin-dependent kinase 1-like	136.08	-1.61	-3.05	0.00	0.00
106582655	LOC106582655	uncharacterized LOC106582655	10.08	-1.61	-3.05	0.00	0.00
106612600	LOC106612600	WD repeat-containing protein 20-like	6.58	-1.60	-3.04	0.00	0.00
106601037	LOC106601037	coiled-coil domain-containing protein 43-like	124.69	-1.60	-3.03	0.00	0.00
106610331	LOC106610331	integrator complex subunit 12-like	38.87	-1.60	-3.03	0.00	0.00
106606254	LOC106606254	phosphatidylinositol N-acetylglucosaminyltransferase subunit Q-li	1.93	-1.60	-3.03	0.00	0.00
106567949	LOC106567949	2-acylglycerol O-acyltransferase 2-A-like	4.01	-1.60	-3.03	0.00	0.00
100136483	LOC100136483	tubulin beta-1 chain	1277.35	-1.60	-3.03	0.01	0.03
106611197	ankrd9	ankyrin repeat domain 9	107.69	-1.60	-3.02	0.00	0.01
106570339	tubb4b	NA	80.77	-1.60	-3.02	0.00	0.00
106603131	LOC106603131	sororin-like	23.75	-1.60	-3.02	0.00	0.00
106584212	LOC106584212	cbp/p300-interacting transactivator 3-like	19.16	-1.60	-3.02	0.00	0.00
106565415	LOC106565415	protein canopy homolog 2-like	193.14	-1.59	-3.02	0.00	0.00
100196583	pebp1	phosphatidylethanolamine binding protein 1	884.01	-1.59	-3.02	0.00	0.00
106612463	drd2	dopamine receptor D2	1.89	-1.59	-3.01	0.01	0.03
106573207	e2f3	E2F transcription factor 3	36.17	-1.59	-3.01	0.00	0.00
106611042	acyp1	acylphosphatase 1	20.81	-1.59	-3.00	0.00	0.00
106566779	LOC106566779	monoglyceride lipase-like	172.53	-1.59	-3.00	0.00	0.00
100136409	LOC100136409	acyl-CoA-binding protein	16952.80	-1.58	-3.00	0.00	0.00
106608759	LOC106608759	diamine acetyltransferase 2-like	6.28	-1.58	-2.99	0.00	0.01
106562710	LOC106562710	very-long-chain 3-oxoacyl-CoA reductase-B-like	578.20	-1.58	-2.99	0.00	0.00
106588586	LOC106588586	nuclear transport factor 2-like	59.39	-1.58	-2.99	0.00	0.00
106573334	LOC106573334	PCNA-associated factor-like	110.35	-1.58	-2.99	0.00	0.00
100194586	hsp10	heat shock protein 10	662.44	-1.58	-2.99	0.00	0.00
106568761	LOC106568761	UMP-CMP kinase	424.65	-1.58	-2.99	0.00	0.00
106587180	LOC106587180	cytochrome b5-like	636.97	-1.58	-2.99	0.01	0.04
106586549	LOC106586549	centromere protein K-like	23.11	-1.58	-2.99	0.00	0.00
100195812	tbb2c	Tubulin beta-2C chain	449.68	-1.58	-2.98	0.00	0.01
106586541	LOC106586541	apolipoprotein A-I-like	3.95	-1.58	-2.98	0.00	0.02
106611697	LOC106611697	mitochondrial import receptor subunit TOM5 homolog	80.07	-1.58	-2.98	0.00	0.02
100196399	dusp23	dual specificity phosphatase 23	30.32	-1.57	-2.98	0.00	0.00
106598937	LOC106598937	cytochrome b-c1 complex subunit 6, mitochondrial-like	163.39	-1.57	-2.97	0.00	0.00
100194902	cebpa	CCAAT/enhancer binding protein (C/EBP), alpha	677.47	-1.57	-2.97	0.00	0.00
106608632	LOC106608632	histone H2A.Z	191.36	-1.57	-2.96	0.00	0.01
106572274	LOC106572274	interferon-related developmental regulator 2-like	267.62	-1.57	-2.96	0.00	0.00
106585487	LOC106585487	cytochrome c oxidase subunit 6A1, mitochondrial-like	208.67	-1.56	-2.96	0.00	0.02
106568532	LOC106568532	asparagine--tRNA ligase, cytoplasmic-like	196.70	-1.56	-2.95	0.00	0.00
106589802	fam195a	MAPK regulated corepressor interacting protein 2	319.84	-1.56	-2.95	0.00	0.00
106582509	LOC106582509	tubulin alpha chain-like	26.70	-1.56	-2.95	0.00	0.00
106595616	LOC106595616	zinc finger protein 2 homolog	2.78	-1.56	-2.95	0.00	0.00
106612247	LOC106612247	leucine-rich repeat-containing protein 59-like	213.36	-1.56	-2.95	0.00	0.01

106564700	LOC106564700	D-tyrosyl-tRNA(Tyr) deacylase 1-like	20.06	-1.56	-2.94	0.00	0.00
106581107	LOC106581107	small nuclear ribonucleoprotein Sm D2	197.46	-1.56	-2.94	0.00	0.00
106575501	LOC106575501	integral membrane protein GPR180-like	36.26	-1.56	-2.94	0.00	0.00
100195978	hdd1a	Haloacid dehalogenase-like hydrolase domain-containing protein	195.10	-1.56	-2.94	0.00	0.01
106588749	LOC106588749	peptidyl-prolyl cis-trans isomerase FKBP9-like	176.12	-1.55	-2.94	0.01	0.03
106613791	LOC106613791	cyclin-dependent kinases regulatory subunit 1-like	47.62	-1.55	-2.93	0.00	0.00
106577215	ppa1	NA	1097.37	-1.55	-2.93	0.00	0.00
106577388	LOC106577388	malate dehydrogenase, cytoplasmic-like	375.02	-1.55	-2.93	0.00	0.00
106585395	LOC106585395	monocarboxylate transporter 13-like	5.11	-1.55	-2.93	0.01	0.04
106568002	LOC106568002	arginine--tRNA ligase, cytoplasmic-like	158.99	-1.54	-2.92	0.00	0.00
106569961	LOC106569961	protein Mis18-alpha-like	3.05	-1.54	-2.92	0.00	0.01
106580382	LOC106580382	ubiquitin-conjugating enzyme E2 D4	42.29	-1.54	-2.91	0.00	0.00
106571421	atf3	activating transcription factor 3	38.02	-1.54	-2.91	0.00	0.00
106586316	LOC106586316	rho-related GTP-binding protein RhoE	66.57	-1.54	-2.91	0.00	0.00
106602584	LOC106602584	V-type proton ATPase 116 kDa subunit a isoform 4-like	14.37	-1.54	-2.90	0.00	0.00
106568292	LOC106568292	density-regulated protein-like	167.14	-1.54	-2.90	0.00	0.00
106563496	LOC106563496	peptidyl-prolyl cis-trans isomerase FKBP2-like	275.20	-1.53	-2.90	0.00	0.01
106573068	LOC106573068	vesicle transport protein GOT1B	168.95	-1.53	-2.89	0.00	0.00
106584335	LOC106584335	glutamate--cysteine ligase regulatory subunit-like	306.82	-1.53	-2.89	0.00	0.02
106565560	LOC106565560	mitochondrial intermembrane space import and assembly proteir	6.42	-1.53	-2.89	0.01	0.03
106563786	LOC106563786	uncharacterized LOC106563786	57.34	-1.53	-2.89	0.00	0.00
106563301	LOC106563301	zinc finger MYND domain-containing protein 19-like	63.76	-1.53	-2.89	0.00	0.00
106584708	LOC106584708	AT-rich interactive domain-containing protein 3B-like	4.60	-1.53	-2.89	0.00	0.00
106601796	g2e3	G2/M-phase specific E3 ubiquitin protein ligase	22.38	-1.52	-2.88	0.00	0.01
106605133	LOC106605133	four and a half LIM domains protein 3-like	2.59	-1.52	-2.88	0.00	0.00
100306826	tp4a1	tyrosine phosphatase type IVA 1	86.10	-1.52	-2.87	0.00	0.02
106601296	LOC106601296	glucose-6-phosphatase-like	2.67	-1.52	-2.87	0.00	0.00
106584422	fam46b	family with sequence similarity 46 member B	84.78	-1.52	-2.87	0.00	0.00
100196714	stml2	Stomatin-like protein 2	135.51	-1.52	-2.87	0.01	0.04
100196215	ndka	Nucleoside diphosphate kinase A	3186.88	-1.52	-2.87	0.01	0.04
100196609	stk6	Serine/threonine-protein kinase 6	64.63	-1.52	-2.87	0.00	0.00
106588057	mrpl46	mitochondrial ribosomal protein L46	119.26	-1.52	-2.86	0.00	0.00
106586251	LOC106586251	cytochrome c	467.65	-1.52	-2.86	0.01	0.03
106609721	LOC106609721	phosphoglycolate phosphatase-like	26.19	-1.51	-2.85	0.00	0.00
100196670	atp5g3	ATP synthase, H+ transporting, mitochondrial Fo complex subunit	497.73	-1.51	-2.85	0.01	0.03
106564820	LOC106564820	uncharacterized LOC106564820	71.06	-1.51	-2.85	0.00	0.02
106569990	LOC106569990	intermediate filament family orphan 1-like	4.73	-1.51	-2.85	0.00	0.01
106608861	LOC106608861	prostaglandin reductase 1-like	33.24	-1.51	-2.85	0.00	0.00
100500786	LOC100500786	arginase-1	21.58	-1.51	-2.85	0.00	0.00
106605376	ccne2	cyclin E2	36.27	-1.51	-2.85	0.01	0.04
106565122	LOC106565122	G0/G1 switch protein 2-like	410.57	-1.51	-2.85	0.00	0.00
100380465	cor1c	Coronin-1C	61.46	-1.51	-2.85	0.00	0.00
106612557	LOC106612557	mitochondrial import inner membrane translocase subunit Tim8 /	96.35	-1.51	-2.84	0.00	0.00
106567204	LOC106567204	tubulin alpha-8 chain-like	110.61	-1.51	-2.84	0.00	0.02
106608935	LOC106608935	GTPase IMAP family member 4-like	21.11	-1.50	-2.84	0.00	0.00
106598856	LOC106598856	retinol dehydrogenase 12-like	1132.42	-1.50	-2.84	0.00	0.01
106607864	LOC106607864	kinesin-like protein KIF11	70.66	-1.50	-2.83	0.00	0.01
100196188	btg3	B-cell translocation gene 3	37.77	-1.50	-2.83	0.00	0.00
106602347	LOC106602347	SWI/SNF-related matrix-associated actin-dependent regulator of (	41.00	-1.50	-2.83	0.00	0.00
100196832	usmg5	Up-regulated during skeletal muscle growth protein 5	387.95	-1.50	-2.83	0.01	0.04
106583130	LOC106583130	uncharacterized LOC106583130	3.94	-1.50	-2.82	0.00	0.00

106585939	LOC106585939	endothelial lipase-like	269.05	-1.49	-2.81	0.01	0.03
106586120	LOC106586120	histone deacetylase 4-like	4.81	-1.49	-2.81	0.00	0.00
106588720	LOC106588720	centromere protein K-like	9.85	-1.49	-2.81	0.00	0.00
106605426	LOC106605426	uncharacterized LOC106605426	3.08	-1.49	-2.81	0.00	0.00
106604299	LOC106604299	long-chain-fatty-acid--CoA ligase 4-like	391.17	-1.49	-2.81	0.00	0.00
106613710	LOC106613710	mitochondrial import inner membrane translocase subunit Tim13	142.82	-1.49	-2.80	0.00	0.00
106581789	spc25	NA	36.70	-1.49	-2.80	0.01	0.03
106601877	LOC106601877	elongation factor Tu, mitochondrial-like	112.05	-1.49	-2.80	0.00	0.02
106578378	LOC106578378	lipocalin-like	14040.68	-1.49	-2.80	0.00	0.01
106605182	LOC106605182	fatty acid-binding protein 10-A, liver basic-like	29591.18	-1.48	-2.80	0.00	0.00
106591659	LOC106591659	NADH dehydrogenase [ubiquinone] 1 beta subcomplex subunit 7-	186.12	-1.48	-2.80	0.01	0.04
106609020	LOC106609020	transmembrane 4 L6 family member 4-like	44.25	-1.48	-2.80	0.00	0.00
106562991	LOC106562991	tubulin alpha chain, testis-specific	1.95	-1.48	-2.79	0.00	0.00
100286655	ybey	ybeY metallopeptidase (putative)	73.46	-1.48	-2.79	0.00	0.00
106579221	LOC106579221	uncharacterized LOC106579221	15.63	-1.48	-2.79	0.00	0.01
106586630	LOC106586630	ATP synthase subunit O, mitochondrial-like	659.54	-1.48	-2.78	0.00	0.00
106582230	LOC106582230	arylsulfatase D-like	54.55	-1.48	-2.78	0.00	0.01
106565535	LOC106565535	peptidyl-prolyl cis-trans isomerase FKBP11-like	1339.16	-1.48	-2.78	0.01	0.04
100196339	nme3	NME/NM23 nucleoside diphosphate kinase 3	104.43	-1.48	-2.78	0.01	0.03
106600794	LOC106600794	supervillin-like	5.31	-1.47	-2.78	0.00	0.00
106565159	pigo	NA	66.98	-1.47	-2.77	0.00	0.01
106584028	LOC106584028	angiopoietin-related protein 3-like	3653.59	-1.47	-2.77	0.00	0.00
106588298	ten1	NA	63.45	-1.47	-2.77	0.00	0.00
106592277	LOC106592277	pyrroline-5-carboxylate reductase 1, mitochondrial-like	570.72	-1.47	-2.77	0.00	0.00
106606812	LOC106606812	uncharacterized LOC106606812	26.52	-1.47	-2.77	0.00	0.02
106606518	LOC106606518	centromere protein X-like	13.25	-1.47	-2.76	0.00	0.00
106577478	LOC106577478	mitochondrial import receptor subunit TOM40 homolog	263.85	-1.47	-2.76	0.00	0.00
100196484	topk	Lymphokine-activated killer T-cell-originated protein kinase homo	151.48	-1.47	-2.76	0.00	0.01
106568941	LOC106568941	mitochondrial import inner membrane translocase subunit TIM44	17.01	-1.46	-2.75	0.00	0.00
106610986	LOC106610986	transforming acidic coiled-coil-containing protein 3-like	77.49	-1.46	-2.75	0.00	0.00
106573903	LOC106573903	gamma-interferon-inducible lysosomal thiol reductase-like	23.67	-1.46	-2.74	0.00	0.00
100194764	tbb5	Tubulin beta chain	930.46	-1.46	-2.74	0.00	0.00
106562286	LOC106562286	cytochrome c oxidase subunit 4 isoform 1, mitochondrial-like	454.78	-1.46	-2.74	0.00	0.00
106577763	LOC106577763	erythropoietin-like	3.65	-1.45	-2.74	0.00	0.00
106561592	LOC106561592	ALX homeobox protein 1-like	2.98	-1.45	-2.73	0.01	0.04
106584736	LOC106584736	transcription factor 12-like	15.04	-1.45	-2.73	0.00	0.00
106613121	LOC106613121	long-chain-fatty-acid--CoA ligase 3-like	11.01	-1.44	-2.72	0.00	0.00
106588702	LOC106588702	NADH dehydrogenase [ubiquinone] iron-sulfur protein 6, mitocho	273.42	-1.44	-2.72	0.00	0.00
100286720	lsm5	U6 snRNA-associated Sm-like protein LSm5	84.19	-1.44	-2.72	0.00	0.00
106573930	LOC106573930	growth arrest and DNA damage-inducible protein GADD45 beta-li	45.93	-1.44	-2.72	0.00	0.00
106583621	LOC106583621	serine/threonine-protein kinase 35-like	6.53	-1.44	-2.71	0.00	0.00
106568945	LOC106568945	transcription factor jun-D-like	73.82	-1.44	-2.71	0.01	0.03
106584900	LOC106584900	retinol dehydrogenase 14-like	113.84	-1.44	-2.71	0.00	0.00
101448008	hdac2	Histone deacetylase 2	11.76	-1.44	-2.71	0.00	0.00
100196159	paf	PCNA-associated factor	38.27	-1.44	-2.71	0.00	0.00
106565020	LOC106565020	guanine nucleotide-binding protein G(z) subunit alpha-like	56.44	-1.44	-2.71	0.00	0.00
106591181	LOC106591181	aspartate aminotransferase, cytoplasmic-like	57.83	-1.43	-2.70	0.00	0.01
106602072	LOC106602072	uncharacterized LOC106602072	193.46	-1.43	-2.70	0.00	0.00
100380461	ci150	CI150 protein	115.59	-1.43	-2.70	0.00	0.02
100194625	atp5g2	ATP synthase, H+ transporting, mitochondrial F0 complex, subunit	1607.86	-1.43	-2.69	0.00	0.00
106584374	LOC106584374	long-chain-fatty-acid--CoA ligase ACSBG2-like	76.16	-1.43	-2.69	0.00	0.00

106604162	LOC106604162	DNA-directed RNA polymerase II subunit RPB7-like	24.23	-1.43	-2.69	0.00	0.00
100195781	pfdn5	prefoldin subunit 5	300.38	-1.43	-2.69	0.00	0.00
106607365	LOC106607365	uncharacterized LOC106607365	7.18	-1.43	-2.69	0.00	0.01
106599979	LOC106599979	E3 ubiquitin-protein ligase RNF126-like	32.16	-1.43	-2.69	0.00	0.00
106566708	LOC106566708	G2/mitotic-specific cyclin-B1-like	138.54	-1.43	-2.69	0.00	0.02
106602585	LOC106602585	pre-mRNA-processing factor 19-like	26.65	-1.43	-2.69	0.01	0.04
106610005	birc5	baculoviral IAP repeat containing 5	99.68	-1.43	-2.69	0.00	0.00
106560829	LOC106560829	uncharacterized oxidoreductase YKL071W-like	685.13	-1.43	-2.69	0.00	0.00
100380681	LOC100380681	tubulin alpha-1C chain	579.77	-1.43	-2.69	0.00	0.00
106603598	LOC106603598	very-long-chain 3-oxoacyl-CoA reductase-B-like	2.39	-1.42	-2.68	0.00	0.00
106564532	LOC106564532	nuclear factor interleukin-3-regulated protein-like	131.95	-1.42	-2.68	0.00	0.00
106587101	ndufs3	NADH:ubiquinone oxidoreductase core subunit S3	440.00	-1.42	-2.68	0.00	0.00
100192341	elovl2	ELOVL fatty acid elongase 2	3941.94	-1.42	-2.68	0.00	0.01
106590672	LOC106590672	transcription elongation factor B polypeptide 1	172.87	-1.42	-2.68	0.00	0.00
100195405	ccne2	cyclin E2	31.31	-1.42	-2.67	0.00	0.00
106590594	LOC106590594	organic solute transporter subunit alpha-like	2.15	-1.42	-2.67	0.00	0.00
100196631	thyn1	thymocyte nuclear protein 1	63.29	-1.42	-2.67	0.01	0.04
106560613	LOC106560613	28S ribosomal protein S36, mitochondrial-like	11.58	-1.42	-2.67	0.00	0.00
106574529	LOC106574529	small ubiquitin-related modifier 3	175.74	-1.41	-2.67	0.00	0.00
106576731	dnph1	2'-deoxynucleoside 5'-phosphate N-hydrolase 1	42.03	-1.41	-2.66	0.01	0.02
106606392	LOC106606392	protein amnionless-like	24.67	-1.41	-2.66	0.00	0.00
100380740	LOC100380740	perilipin-2	503.47	-1.41	-2.66	0.00	0.00
106571857	lyrm7	LYR motif containing 7	55.63	-1.41	-2.66	0.00	0.00
106612309	LOC106612309	HIG1 domain family member 2A-like	194.47	-1.41	-2.66	0.00	0.00
106585276	LOC106585276	cyclin-dependent kinase 2-associated protein 1-like	120.84	-1.41	-2.66	0.01	0.03
106588465	LOC106588465	adenylate kinase 2, mitochondrial	622.72	-1.41	-2.66	0.00	0.00
106586370	LOC106586370	MOB-like protein phocein	705.86	-1.41	-2.65	0.00	0.00
106600984	LOC106600984	ADP-ribosylation factor-like protein 5B	25.39	-1.41	-2.65	0.00	0.00
106560323	LOC106560323	4-hydroxyphenylpyruvate dioxygenase-like protein	9.13	-1.41	-2.65	0.00	0.00
106607702	LOC106607702	mitotic checkpoint serine/threonine-protein kinase BUB1-like	41.27	-1.41	-2.65	0.00	0.00
100195631	kcne4	potassium voltage-gated channel, Isk-related family, member 4	4.03	-1.41	-2.65	0.00	0.00
100380627	LOC100380627	neuronal pentraxin-1	74.99	-1.41	-2.65	0.00	0.00
106582290	LOC106582290	uncharacterized LOC106582290	3.58	-1.40	-2.65	0.00	0.00
106589088	LOC106589088	sodium-dependent lysophosphatidylcholine symporter 1-B-like	4.64	-1.40	-2.65	0.00	0.00
106585066	LOC106585066	uncharacterized LOC106585066	26.10	-1.40	-2.64	0.00	0.00
100195237	gfpt1	glutamine-fructose-6-phosphate transaminase 1	382.17	-1.40	-2.64	0.00	0.00
106563745	LOC106563745	NADH dehydrogenase [ubiquinone] 1 alpha subcomplex subunit 2	295.62	-1.40	-2.64	0.00	0.00
106602876	LOC106602876	ATP synthase subunit g, mitochondrial-like	590.69	-1.40	-2.64	0.01	0.03
106568366	LOC106568366	uncharacterized LOC106568366	1521.38	-1.40	-2.64	0.00	0.00
100380723	LOC100380723	exportin-1	57.69	-1.40	-2.64	0.00	0.00
106570560	LOC106570560	protein FAM65B-like	29.55	-1.40	-2.64	0.00	0.00
100136477	LOC100136477	peroxisome proliferator-activated receptor gamma coactivator 1-	144.73	-1.40	-2.64	0.00	0.00
106581298	LOC106581298	long-chain-fatty-acid--CoA ligase 3-like	5.21	-1.40	-2.64	0.00	0.00
106589562	LOC106589562	BRICHOS domain-containing protein 5-like	9.48	-1.40	-2.64	0.00	0.00
106589369	cep55	centrosomal protein 55	22.34	-1.40	-2.64	0.00	0.00
106589734	golga4	golgin A4	457.28	-1.40	-2.64	0.00	0.00
106571543	LOC106571543	hydroxymethylglutaryl-CoA synthase, cytoplasmic-like	3152.20	-1.40	-2.63	0.00	0.00
100195954	ccdc58	coiled-coil domain containing 58	180.14	-1.40	-2.63	0.00	0.00
106601402	LOC106601402	cytoglobin-2-like	5.35	-1.40	-2.63	0.00	0.00
100196320	rtkn2	rhotekin 2	41.66	-1.40	-2.63	0.00	0.01
106603271	LOC106603271	acetyl-CoA carboxylase	11006.06	-1.40	-2.63	0.00	0.00

106610772	LOC106610772	cytochrome c oxidase subunit 7A2, mitochondrial-like	515.96	-1.39	-2.62	0.00	0.02
106574634	LOC106574634	uncharacterized protein KIAA1211-like	110.45	-1.39	-2.62	0.00	0.02
106585804	LOC106585804	exportin-1	44.48	-1.39	-2.62	0.00	0.00
100194626	atp5g3	ATP synthase, H+ transporting, mitochondrial F0 complex, subunit	1821.59	-1.39	-2.62	0.00	0.00
106607307	LOC106607307	coiled-coil domain-containing protein 43-like	88.20	-1.39	-2.62	0.00	0.00
106605546	LOC106605546	squalene monooxygenase-like	326.54	-1.39	-2.62	0.00	0.01
100195802	g45ip	Growth arrest and DNA-damage-inducible proteins-interacting pr	95.52	-1.39	-2.62	0.00	0.00
106564887	LOC106564887	axin-2-like	21.85	-1.39	-2.62	0.00	0.00
106563787	LOC106563787	uncharacterized LOC106563787	15.36	-1.39	-2.61	0.01	0.05
106560390	LOC106560390	uncharacterized LOC106560390	3.78	-1.39	-2.61	0.00	0.00
106560341	LOC106560341	4-hydroxyphenylpyruvate dioxygenase-like protein	24.64	-1.39	-2.61	0.00	0.00
106607943	LOC106607943	protein FAM46A-like	2.29	-1.38	-2.61	0.01	0.03
106567765	LOC106567765	nuclear fragile X mental retardation-interacting protein 2-like	160.09	-1.38	-2.61	0.00	0.00
100194862	pgm3	phosphoglucomutase 3	310.39	-1.38	-2.61	0.01	0.03
106602580	LOC106602580	NADH dehydrogenase [ubiquinone] iron-sulfur protein 8, mitocho	512.55	-1.38	-2.61	0.00	0.02
106579956	pptc7	NA	221.21	-1.38	-2.61	0.01	0.03
106612226	LOC106612226	cytochrome b-c1 complex subunit 8-like	112.24	-1.38	-2.61	0.00	0.00
106586842	LOC106586842	interferon alpha/beta receptor 1a-like	7.13	-1.38	-2.61	0.00	0.00
106565803	LOC106565803	tubulin monoglycylase TTLL3-like	14.90	-1.38	-2.61	0.00	0.00
106577654	LOC106577654	uncharacterized LOC106577654	13.86	-1.38	-2.60	0.00	0.00
100306857	inceb	Inner centromere protein B	273.94	-1.38	-2.60	0.00	0.00
100196141	ap1s3	AP-1 complex subunit sigma-3	22.81	-1.38	-2.60	0.00	0.00
106612619	LOC106612619	kelch-like protein 12	31.19	-1.38	-2.59	0.00	0.00
106579160	LOC106579160	probable phosphatase phospho1	4.61	-1.37	-2.59	0.00	0.00
100380449	stk35	serine/threonine kinase 35	34.93	-1.37	-2.59	0.00	0.01
100195349	fpps	Farnesyl pyrophosphate synthetase	961.67	-1.37	-2.59	0.01	0.04
106601215	LOC106601215	coiled-coil domain-containing protein 134-like	33.41	-1.37	-2.59	0.00	0.01
100196448	apoo	apolipoprotein O	82.15	-1.37	-2.59	0.00	0.00
106562651	fah	fumarylacetoacetate hydrolase	2770.18	-1.37	-2.59	0.00	0.00
100136430	LOC100136430	60 kDa heat shock protein, mitochondrial	1561.26	-1.37	-2.59	0.00	0.00
106576752	LOC106576752	E3 ubiquitin-protein ligase TRIM39-like	13.46	-1.37	-2.58	0.00	0.00
106586099	LOC106586099	GTPase IMAP family member 8-like	28.83	-1.37	-2.58	0.00	0.00
100380815	sephs1	selenophosphate synthetase 1	290.40	-1.37	-2.58	0.00	0.00
106580785	LOC106580785	sodium-dependent lysophosphatidylcholine symporter 1-B-like	4.31	-1.37	-2.58	0.00	0.00
106568019	LOC106568019	cytochrome c oxidase subunit 7B, mitochondrial-like	954.76	-1.37	-2.58	0.01	0.03
100196122	cdc2	Cell division control protein 2 homolog	224.64	-1.37	-2.58	0.00	0.00
100196160	dck	deoxycytidine kinase	100.30	-1.37	-2.58	0.00	0.00
106586586	LOC106586586	acyl-CoA-binding protein-like	97.60	-1.37	-2.58	0.00	0.00
106572230	LOC106572230	targeting protein for Xklp2-A-like	102.54	-1.37	-2.58	0.00	0.00
106601404	LOC106601404	uncharacterized LOC106601404	7.48	-1.37	-2.58	0.00	0.00
100196702	g6pd	glucose-6-phosphate dehydrogenase	910.25	-1.36	-2.57	0.00	0.01
106588823	gmnn	geminin, DNA replication inhibitor	49.71	-1.36	-2.57	0.00	0.00
106577103	LOC106577103	F-box only protein 5-like	22.54	-1.36	-2.57	0.00	0.00
100195559	pgd	phosphogluconate dehydrogenase	2010.90	-1.36	-2.57	0.00	0.00
106562876	LOC106562876	uncharacterized LOC106562876	4.70	-1.36	-2.57	0.00	0.01
100380346	myc2	Transcriptional regulator Myc-2	128.30	-1.36	-2.57	0.00	0.00
106574774	LOC106574774	uncharacterized LOC106574774	7.73	-1.36	-2.57	0.00	0.00
106564832	LOC106564832	nidogen-1-like	30.32	-1.36	-2.57	0.00	0.01
106612565	LOC106612565	39S ribosomal protein L44, mitochondrial-like	119.08	-1.36	-2.57	0.00	0.00
100195794	prdx1	peroxiredoxin 1	418.63	-1.36	-2.56	0.00	0.00
100286718	ccne1	cyclin E1	31.87	-1.36	-2.56	0.00	0.01

106602291	LOC106602291	bifunctional 3'-phosphoadenosine 5'-phosphosulfate synthase 1-li	26.19	-1.36	-2.56	0.00	0.00
100194849	fgf13	fibroblast growth factor 13	34.96	-1.36	-2.56	0.00	0.02
106607046	LOC106607046	kinetochore protein Spc24-like	41.67	-1.36	-2.56	0.00	0.00
106580656	serp1	stress associated endoplasmic reticulum protein 1	1276.99	-1.36	-2.56	0.00	0.00
100195587	borea	Borealin	99.10	-1.35	-2.56	0.01	0.04
106578447	LOC106578447	NADH dehydrogenase [ubiquinone] 1 beta subcomplex subunit 4-	289.85	-1.35	-2.56	0.00	0.00
100194593	nt5c3	5'-nucleotidase, cytosolic III	3812.63	-1.35	-2.56	0.00	0.01
106567062	LOC106567062	RNA-binding motif, single-stranded-interacting protein 2-like	11.48	-1.35	-2.56	0.01	0.03
106578961	LOC106578961	actin, aortic smooth muscle	19.95	-1.35	-2.56	0.00	0.00
106600819	LOC106600819	uncharacterized LOC106600819	42.59	-1.35	-2.55	0.00	0.00
106563510	LOC106563510	chromosome-associated kinesin KIF4-like	95.79	-1.35	-2.55	0.00	0.00
106570496	LOC106570496	D-dopachrome decarboxylase-A-like	3804.87	-1.35	-2.55	0.00	0.00
106584275	LOC106584275	dimethylaniline monooxygenase [N-oxide-forming] 5-like	44.50	-1.35	-2.55	0.01	0.03
106572219	LOC106572219	coiled-coil domain-containing protein 22-like	115.82	-1.35	-2.55	0.00	0.01
106586529	tsc22d1	NA	64.81	-1.35	-2.55	0.00	0.02
106578510	LOC106578510	palmdelphin-like	97.47	-1.35	-2.55	0.00	0.00
100195132	ima2	Importin subunit alpha-2	243.20	-1.35	-2.55	0.00	0.00
106572462	LOC106572462	pancreatic progenitor cell differentiation and proliferation factor-	14.73	-1.35	-2.55	0.00	0.00
100196409	cks1	Cyclin-dependent kinases regulatory subunit 1	28.75	-1.35	-2.55	0.00	0.01
106604252	LOC106604252	histone-lysine N-methyltransferase 2B-like	6.50	-1.35	-2.55	0.00	0.00
106574437	LOC106574437	glycerol-3-phosphate dehydrogenase 1-like protein	903.85	-1.35	-2.55	0.00	0.00
106577204	LOC106577204	ribosomal protein 63, mitochondrial-like	104.17	-1.35	-2.54	0.00	0.02
106561084	LOC106561084	G/T mismatch-specific thymine DNA glycosylase-like	76.37	-1.35	-2.54	0.01	0.03
106579249	LOC106579249	cytochrome c oxidase subunit 6B1-like	1576.73	-1.34	-2.54	0.00	0.00
100196854	prim1	primase (DNA) subunit 1	219.52	-1.34	-2.54	0.00	0.00
106588321	LOC106588321	interleukin enhancer-binding factor 2 homolog	166.23	-1.34	-2.54	0.00	0.00
100196617	dh12b	Estradiol 17-beta-dehydrogenase 12-B	2208.09	-1.34	-2.53	0.00	0.00
106563520	LOC106563520	kinesin-like protein KIF18A	11.36	-1.34	-2.53	0.00	0.02
106568681	LOC106568681	allograft inflammatory factor 1-like	2.59	-1.34	-2.53	0.00	0.00
106566600	LOC106566600	complement component C7-like	24.39	-1.34	-2.53	0.00	0.01
106564571	LOC106564571	glutaryl-CoA dehydrogenase, mitochondrial-like	232.91	-1.34	-2.53	0.00	0.02
106587515	LOC106587515	mitochondrial glutamate carrier 1-like	481.67	-1.34	-2.53	0.00	0.00
106587404	LOC106587404	aspartate aminotransferase, mitochondrial-like	1076.74	-1.34	-2.53	0.00	0.00
106607281	LOC106607281	ATP-citrate synthase	515.23	-1.34	-2.53	0.00	0.01
100195917	ppcs	phosphopantothenoylcysteine synthetase	84.90	-1.34	-2.53	0.00	0.00
106606199	tigd1	NA	2.99	-1.34	-2.53	0.00	0.01
106580731	LOC106580731	protein YIF1B-like	15.36	-1.34	-2.53	0.00	0.01
106584012	LOC106584012	phosphatidylinositol 3-kinase regulatory subunit gamma	204.72	-1.34	-2.52	0.00	0.00
106565643	mrpl20	mitochondrial ribosomal protein L20	170.01	-1.34	-2.52	0.01	0.03
106607768	hddc2	HD domain containing 2	65.19	-1.33	-2.52	0.01	0.04
106604515	LOC106604515	elongation of very long chain fatty acids protein 6	3995.69	-1.33	-2.52	0.00	0.00
100380429	rcc1	regulator of chromosome condensation 1	338.73	-1.33	-2.51	0.00	0.00
106603038	LOC106603038	kinesin-like protein KIF20A	71.01	-1.33	-2.51	0.01	0.03
106571077	LOC106571077	dolichyl-diphosphooligosaccharide--protein glycosyltransferase su	252.15	-1.33	-2.51	0.00	0.00
106588497	LOC106588497	mediator of RNA polymerase II transcription subunit 30-like	19.00	-1.33	-2.51	0.00	0.00
106599713	LOC106599713	tubulin beta-2A chain-like	67.04	-1.33	-2.51	0.00	0.00
106564857	LOC106564857	malate dehydrogenase, cytoplasmic-like	5880.70	-1.33	-2.51	0.00	0.01
100196079	ruxg	Small nuclear ribonucleoprotein G	208.29	-1.33	-2.51	0.00	0.00
106575559	LOC106575559	integral membrane protein GPR180-like	16.79	-1.33	-2.51	0.00	0.00
106612221	eme1	essential meiotic structure-specific endonuclease 1	14.32	-1.33	-2.51	0.00	0.01
106566031	LOC106566031	uncharacterized LOC106566031	19.06	-1.33	-2.51	0.00	0.00

106561071	LOC106561071	transmembrane emp24 domain-containing protein 6-like	2.67	-1.33	-2.51	0.00	0.00
106563592	LOC106563592	lipocalin-like	23.54	-1.33	-2.51	0.00	0.00
106606342	LOC106606342	elongation factor Tu, mitochondrial-like	27.24	-1.32	-2.50	0.00	0.00
100286418	mis12	MIS12, kinetochore complex component	27.81	-1.32	-2.50	0.00	0.02
106581108	LOC106581108	clathrin heavy chain 1-like	102.53	-1.32	-2.50	0.00	0.00
106610334	aimp1	aminoacyl tRNA synthetase complex interacting multifunctional p	87.41	-1.32	-2.50	0.00	0.00
106612340	LOC106612340	uncharacterized LOC106612340	14.91	-1.32	-2.50	0.00	0.00
106562705	LOC106562705	stAR-related lipid transfer protein 5-like	35.79	-1.32	-2.50	0.00	0.00
106605708	LOC106605708	F-box only protein 5-like	4.36	-1.32	-2.50	0.00	0.00
106583326	trib3	NA	95.10	-1.32	-2.50	0.00	0.00
106579980	LOC106579980	tumor necrosis factor ligand superfamily member 6-like	73.74	-1.32	-2.50	0.00	0.00
100196541	crj1a	Crystallin J1A	246.71	-1.32	-2.50	0.00	0.00
106582879	LOC106582879	uncharacterized LOC106582879	83.13	-1.32	-2.50	0.00	0.01
106605924	LOC106605924	farnesyl pyrophosphate synthase-like	753.05	-1.32	-2.49	0.00	0.00
106581758	LOC106581758	cell division cycle-associated protein 7-like	97.45	-1.32	-2.49	0.00	0.00
106606404	LOC106606404	RNA-binding protein with serine-rich domain 1-like	116.84	-1.32	-2.49	0.00	0.00
106588762	LOC106588762	U5 small nuclear ribonucleoprotein 40 kDa protein-like	62.44	-1.32	-2.49	0.00	0.00
106612199	LOC106612199	sterol-4-alpha-carboxylate 3-dehydrogenase, decarboxylating-like	174.84	-1.32	-2.49	0.00	0.02
106584125	cep135	centrosomal protein 135	15.98	-1.32	-2.49	0.00	0.00
106568014	LOC106568014	zinc finger protein 346-like	108.25	-1.31	-2.49	0.00	0.00
106606172	LOC106606172	inactive ubiquitin carboxyl-terminal hydrolase 54-like	23.95	-1.31	-2.49	0.00	0.01
106611409	LOC106611409	solute carrier family 22 member 13-like	23.17	-1.31	-2.49	0.00	0.00
106608168	LOC106608168	26S protease regulatory subunit 10B-like	275.12	-1.31	-2.49	0.00	0.00
100380551	ifrd2	interferon related developmental regulator 2	241.14	-1.31	-2.49	0.01	0.04
106603375	ercc6l	ERCC excision repair 6 like, spindle assembly checkpoint helicase	67.99	-1.31	-2.49	0.01	0.03
106605770	LOC106605770	ubiquitin-conjugating enzyme E2 S	34.78	-1.31	-2.48	0.00	0.00
106569895	LOC106569895	T-complex protein 1 subunit gamma-like	119.17	-1.31	-2.48	0.00	0.00
106602033	LOC106602033	cofilin-2-like	62.59	-1.31	-2.48	0.01	0.04
106563509	LOC106563509	28S ribosomal protein S12, mitochondrial-like	69.25	-1.31	-2.48	0.00	0.00
106569347	LOC106569347	ATP-binding cassette sub-family B member 8, mitochondrial-like	113.95	-1.31	-2.48	0.00	0.01
106564539	LOC106564539	serine hydrolase-like protein	59.14	-1.31	-2.48	0.00	0.00
100196818	phf5a	PHD finger protein 5A	155.81	-1.31	-2.48	0.01	0.03
106607871	LOC106607871	uncharacterized LOC106607871	18.74	-1.31	-2.48	0.00	0.00
106561942	LOC106561942	NADH dehydrogenase [ubiquinone] iron-sulfur protein 3, mitocho	210.72	-1.31	-2.48	0.00	0.00
106582767	LOC106582767	retinoid-binding protein 7-like	1022.49	-1.31	-2.48	0.00	0.00
106605532	LOC106605532	protein LYRIC-like	2819.45	-1.31	-2.48	0.00	0.01
106588708	mrps18b	mitochondrial ribosomal protein S18B	213.56	-1.31	-2.48	0.00	0.00
106584797	LOC106584797	fatty acid desaturase 2-like	38.21	-1.31	-2.47	0.01	0.05
106588646	LOC106588646	stathmin-like	161.75	-1.31	-2.47	0.00	0.00
106570296	LOC106570296	actin-binding protein anillin-like	41.44	-1.31	-2.47	0.00	0.00
106568991	LOC106568991	15 kDa selenoprotein-like	217.33	-1.30	-2.47	0.00	0.01
106562291	LOC106562291	ER membrane protein complex subunit 8 pseudogene	53.04	-1.30	-2.47	0.00	0.00
106586378	LOC106586378	uncharacterized LOC106586378	18.33	-1.30	-2.47	0.00	0.00
106561666	LOC106561666	neuroepithelial cell-transforming gene 1 protein-like	47.79	-1.30	-2.47	0.00	0.00
100195529	prc1	regulator of cytokinesis 1	53.82	-1.30	-2.47	0.00	0.00
106565678	espl1	extra spindle pole bodies like 1, separase	41.02	-1.30	-2.46	0.01	0.04
106589067	LOC106589067	serum response factor-binding protein 1-like	70.18	-1.30	-2.46	0.00	0.01
100286508	cetn3	centrin 3	41.14	-1.30	-2.46	0.00	0.00
106612607	LOC106612607	heterogeneous nuclear ribonucleoprotein L-like	272.92	-1.30	-2.46	0.00	0.00
106567558	LOC106567558	uncharacterized LOC106567558	1010.64	-1.30	-2.46	0.00	0.02
106602990	LOC106602990	cytochrome c oxidase subunit 8B, mitochondrial-like	523.77	-1.30	-2.46	0.00	0.00

106560461	sgip1	NA	5.99	-1.30	-2.46	0.00	0.00
106605633	LOC106605633	transmembrane protein 147	277.61	-1.30	-2.46	0.01	0.03
106561979	LOC106561979	uncharacterized protein KIAA0754-like	112.33	-1.30	-2.46	0.00	0.00
106585828	elac1	elaC ribonuclease Z 1	68.51	-1.30	-2.46	0.00	0.00
106599157	LOC106599157	choline transporter-like protein 5-A	104.10	-1.30	-2.46	0.00	0.00
106608242	LOC106608242	coiled-coil domain-containing protein 85C-A-like	44.30	-1.30	-2.46	0.00	0.00
106571483	LOC106571483	28S ribosomal protein S18a, mitochondrial-like	74.41	-1.30	-2.45	0.00	0.00
106587100	ptpmt1	NA	310.61	-1.29	-2.45	0.00	0.00
106602346	LOC106602346	SWI/SNF-related matrix-associated actin-dependent regulator of c	41.33	-1.29	-2.45	0.00	0.00
106599400	mrps14	mitochondrial ribosomal protein S14	238.37	-1.29	-2.45	0.00	0.01
106610355	LOC106610355	inorganic pyrophosphatase-like	73.68	-1.29	-2.45	0.00	0.01
100846962	dad1	defender against cell death 1	1172.76	-1.29	-2.45	0.00	0.01
106604298	pin4	NA	265.39	-1.29	-2.45	0.00	0.01
100195168	pura1	Adenylosuccinate synthetase isozyme 1	890.58	-1.29	-2.45	0.00	0.00
106579710	LOC106579710	decaprenyl-diphosphate synthase subunit 1-like	47.98	-1.29	-2.45	0.00	0.01
106606416	LOC106606416	cyclin-F-like	25.15	-1.29	-2.45	0.00	0.00
106608956	LOC106608956	sentrin-specific protease 3-like	43.01	-1.29	-2.45	0.00	0.00
106584399	LOC106584399	chromatin assembly factor 1 subunit A-like	40.16	-1.29	-2.45	0.00	0.00
106568767	LOC106568767	uncharacterized LOC106568767	5.09	-1.29	-2.44	0.00	0.00
106578241	slc25a11	solute carrier family 25 member 11	397.79	-1.29	-2.44	0.00	0.00
106588568	LOC106588568	lanosterol 14-alpha demethylase	1300.34	-1.29	-2.44	0.00	0.00
106607059	LOC106607059	uncharacterized protein C16orf45 homolog	8.56	-1.29	-2.44	0.00	0.00
106574863	LOC106574863	pyridoxal phosphate phosphatase PHOSPHO2-like	41.60	-1.29	-2.44	0.00	0.00
106585148	LOC106585148	39S ribosomal protein L41, mitochondrial-like	30.55	-1.29	-2.44	0.00	0.00
106570899	LOC106570899	progestin and adipoQ receptor family member 3-like	15.52	-1.29	-2.44	0.00	0.02
100196810	rad1	RAD1 checkpoint DNA exonuclease	21.21	-1.29	-2.44	0.00	0.00
106588760	LOC106588760	small nuclear ribonucleoprotein Sm D1	189.05	-1.29	-2.44	0.00	0.00
106606624	LOC106606624	small ubiquitin-related modifier 2	105.31	-1.28	-2.44	0.00	0.00
100195654	kcrt	Creatine kinase, testis isozyme	11.97	-1.28	-2.43	0.00	0.00
106607066	LOC106607066	importin subunit alpha-1-like	141.76	-1.28	-2.43	0.00	0.00
106563479	LOC106563479	steroid hormone receptor ERR1-like	113.79	-1.28	-2.43	0.01	0.03
106579279	LOC106579279	redox-regulatory protein FAM213A-like	5.31	-1.28	-2.43	0.01	0.05
106583903	LOC106583903	uncharacterized LOC106583903	142.37	-1.28	-2.43	0.00	0.00
106586240	LOC106586240	uncharacterized LOC106586240	96.32	-1.28	-2.43	0.00	0.00
106576953	fam98a	family with sequence similarity 98 member A	147.73	-1.28	-2.42	0.00	0.00
106590036	LOC106590036	zinc finger CCCH domain-containing protein 15-like	1597.39	-1.28	-2.42	0.00	0.01
106609190	LOC106609190	uncharacterized LOC106609190	6.54	-1.27	-2.42	0.00	0.00
106600427	LOC106600427	Niemann-Pick C1 protein-like	897.52	-1.27	-2.42	0.00	0.00
100196112	cdkn3	cyclin dependent kinase inhibitor 3	43.74	-1.27	-2.42	0.00	0.01
106571633	LOC106571633	3'(2'),5'-bisphosphate nucleotidase 1-like	364.25	-1.27	-2.42	0.00	0.00
106569628	LOC106569628	tropomyosin alpha-1 chain-like	77.47	-1.27	-2.41	0.01	0.04
106573213	LOC106573213	aryl hydrocarbon receptor nuclear translocator-like protein 1	109.70	-1.27	-2.41	0.00	0.00
106576735	LOC106576735	nuclear receptor coactivator 4-like	2554.07	-1.27	-2.41	0.00	0.00
106561721	LOC106561721	cytochrome c oxidase subunit 5A, mitochondrial-like	94.39	-1.27	-2.41	0.00	0.01
106584318	LOC106584318	voltage-dependent R-type calcium channel subunit alpha-1E-like	35.45	-1.27	-2.41	0.00	0.00
106587628	LOC106587628	guanosine-3',5'-bis(diphosphate) 3'-pyrophosphohydrolase MESH	43.35	-1.26	-2.40	0.00	0.01
106577697	LOC106577697	transmembrane protein 256-like	65.19	-1.26	-2.40	0.00	0.00
106607811	LOC106607811	lymphokine-activated killer T-cell-originated protein kinase homo	68.84	-1.26	-2.40	0.00	0.00
106603066	LOC106603066	eukaryotic translation initiation factor 1A, X-chromosomal-like	553.54	-1.26	-2.40	0.01	0.03
100195770	chch1	Coiled-coil-helix-coiled-coil-helix domain-containing protein 1	98.27	-1.26	-2.40	0.00	0.00
106576621	LOC106576621	transcription factor E2F7-like	27.37	-1.26	-2.40	0.00	0.00

106573105	LOC106573105	aurora kinase B-like	65.70	-1.26	-2.40	0.00	0.02
106563197	fam136a	family with sequence similarity 136 member A	179.20	-1.26	-2.39	0.01	0.04
106600020	LOC106600020	endophilin-A2-like	19.57	-1.26	-2.39	0.00	0.00
106604459	mars2	methionyl-tRNA synthetase 2, mitochondrial	45.49	-1.26	-2.39	0.01	0.04
106603189	LOC106603189	28S ribosomal protein S12, mitochondrial-like	92.66	-1.26	-2.39	0.00	0.00
106573338	gdpgp1	GDP-D-glucose phosphorylase 1	22.49	-1.26	-2.39	0.00	0.00
106591833	LOC106591833	NAD-dependent protein deacetylase sirtuin-7-like	18.61	-1.25	-2.39	0.00	0.00
106602305	LOC106602305	PC4 and SFRS1-interacting protein-like	13.29	-1.25	-2.39	0.00	0.00
106586601	LOC106586601	cytochrome c oxidase copper chaperone	198.99	-1.25	-2.39	0.00	0.00
106610881	pole2	NA	41.14	-1.25	-2.38	0.01	0.03
100380530	gats	opposite strand transcription unit to Stag3	4.35	-1.25	-2.38	0.00	0.00
106564049	LOC106564049	zinc finger protein 501-like	4.63	-1.25	-2.38	0.00	0.00
100196672	LOC100196672	general transcription factor IIE, polypeptide 2-2	41.68	-1.25	-2.38	0.00	0.00
100195172	r51a1	NA	16.88	-1.25	-2.38	0.00	0.00
106589648	LOC106589648	U6 snRNA-associated Sm-like protein LSm8	366.36	-1.25	-2.38	0.01	0.03
106576419	LOC106576419	forkhead box protein M1-like	26.52	-1.25	-2.38	0.00	0.00
106611357	cenpf	centromere protein F	87.27	-1.25	-2.38	0.00	0.00
106612394	dna2	DNA replication helicase/nuclease 2	61.55	-1.25	-2.38	0.00	0.00
106582878	LOC106582878	host cell factor 1-like	75.17	-1.25	-2.38	0.00	0.00
106566758	LOC106566758	cat eye syndrome critical region protein 5-like	15.61	-1.25	-2.38	0.00	0.00
106571497	LOC106571497	centromere protein W-like	31.97	-1.25	-2.38	0.00	0.00
106562504	LOC106562504	NAD(P) transhydrogenase, mitochondrial-like	56.92	-1.25	-2.38	0.00	0.00
106585818	LOC106585818	COMM domain-containing protein 1-like	51.35	-1.25	-2.38	0.00	0.00
106568355	LOC106568355	claudin-5-like	5.77	-1.25	-2.37	0.00	0.00
106591329	LOC106591329	aspartate aminotransferase, cytoplasmic-like	69.69	-1.25	-2.37	0.00	0.00
106604942	spdl1	NA	47.81	-1.25	-2.37	0.00	0.00
100195262	lyric	LYRIC protein	1664.88	-1.25	-2.37	0.01	0.03
106597610	LOC106597610	39S ribosomal protein L35, mitochondrial-like	254.61	-1.24	-2.37	0.00	0.00
106603062	sat1	NA	200.80	-1.24	-2.37	0.00	0.00
106563680	LOC106563680	G2/mitotic-specific cyclin-B3-like	20.13	-1.24	-2.37	0.00	0.00
106562758	LOC106562758	splicing factor U2AF 35 kDa subunit-like	290.95	-1.24	-2.36	0.00	0.00
106571942	LOC106571942	DNA primase small subunit-like	9.30	-1.24	-2.36	0.00	0.00
100195885	s22bb	Vesicle-trafficking protein SEC22b-B	20.42	-1.24	-2.36	0.00	0.00
106573897	LOC106573897	cAMP-specific 3',5'-cyclic phosphodiesterase 4C-like	61.54	-1.24	-2.36	0.00	0.00
106560578	LOC106560578	prohibitin-2-like	747.93	-1.24	-2.36	0.00	0.02
106566812	LOC106566812	rho guanine nucleotide exchange factor 10-like protein	43.61	-1.24	-2.36	0.00	0.00
100195316	wbscr16	RCC1 like	322.12	-1.24	-2.36	0.00	0.00
106589387	LOC106589387	actin, aortic smooth muscle	46.84	-1.24	-2.36	0.00	0.00
106584469	adck2	aarF domain containing kinase 2	34.88	-1.24	-2.36	0.00	0.00
106582294	LOC106582294	BRCA1-associated RING domain protein 1-like	19.59	-1.24	-2.36	0.00	0.00
106591234	LOC106591234	uncharacterized LOC106591234	28.05	-1.24	-2.36	0.00	0.00
106581179	LOC106581179	glyoxalase domain-containing protein 4-like	1181.31	-1.24	-2.36	0.00	0.01
106560679	LOC106560679	ATP synthase subunit g, mitochondrial-like	687.27	-1.24	-2.36	0.00	0.00
106577778	mettl3	methyltransferase like 3	85.91	-1.24	-2.36	0.00	0.00
106606306	LOC106606306	nuclear distribution protein nudE-like 1-B	64.74	-1.24	-2.36	0.00	0.02
100196866	psde	26S proteasome non-ATPase regulatory subunit 14	208.32	-1.24	-2.35	0.00	0.00
106566185	LOC106566185	coiled-coil domain-containing protein 51-like	22.58	-1.24	-2.35	0.00	0.00
106587886	LOC106587886	26S proteasome non-ATPase regulatory subunit 13-like	90.10	-1.24	-2.35	0.00	0.01
106609950	LOC106609950	N-acyl-aromatic-L-amino acid amidohydrolase (carboxylate-formi	692.90	-1.24	-2.35	0.00	0.01
106571470	LOC106571470	uncharacterized LOC106571470	82.40	-1.23	-2.35	0.00	0.00
106565519	LOC106565519	elongation factor Ts, mitochondrial	180.06	-1.23	-2.35	0.00	0.01

100196511	sld5	DNA replication complex GINS protein SLD5	173.10	-1.23	-2.35	0.00	0.00
106600830	LOC106600830	ATP synthase F(0) complex subunit C3, mitochondrial-like	1384.43	-1.23	-2.35	0.00	0.00
106575285	LOC106575285	uncharacterized LOC106575285	11.12	-1.23	-2.35	0.00	0.01
106563640	LOC106563640	putative defense protein Hdd11	21.02	-1.23	-2.35	0.00	0.02
106607749	LOC106607749	nucleoporin Nup43-like	51.10	-1.23	-2.35	0.00	0.00
100195646	dtd1	D-tyrosyl-tRNATyr deacylase 1	31.21	-1.23	-2.35	0.00	0.00
106578798	cssa19h6orf118	chromosome ssa19 open reading frame, human C6orf118	27.53	-1.23	-2.34	0.00	0.00
106586273	LOC106586273	pyridoxal kinase-like	11.63	-1.23	-2.34	0.00	0.00
100286684	haus1	HAUS augmin like complex subunit 1	75.93	-1.23	-2.34	0.00	0.00
106605312	LOC106605312	nestin-like	5.74	-1.23	-2.34	0.00	0.00
100380852	LOC100380852	ectonucleotide pyrophosphatase/phosphodiesterase family mem	9.39	-1.23	-2.34	0.00	0.00
106574334	LOC106574334	ATP synthase-coupling factor 6, mitochondrial-like	414.84	-1.23	-2.34	0.00	0.00
106567406	LOC106567406	complement component 1 Q subcomponent-binding protein, mit	569.86	-1.23	-2.34	0.00	0.01
106576033	LOC106576033	leukotriene A-4 hydrolase-like	45.00	-1.23	-2.34	0.00	0.01
106589580	LOC106589580	class A basic helix-loop-helix protein 15-like	5.10	-1.23	-2.34	0.00	0.00
106564769	mtif2	mitochondrial translational initiation factor 2	90.66	-1.23	-2.34	0.00	0.00
106570325	LOC106570325	uncharacterized LOC106570325	63.67	-1.23	-2.34	0.00	0.02
106562484	dut	deoxyuridine triphosphatase	188.21	-1.23	-2.34	0.00	0.01
106609290	LOC106609290	centrosomal protein of 83 kDa-like	36.16	-1.22	-2.34	0.01	0.04
100380396	atad3	ATPase family AAA domain-containing protein 3	429.91	-1.22	-2.34	0.00	0.01
106560975	LOC106560975	NUAK family SNF1-like kinase 1	10.29	-1.22	-2.34	0.00	0.00
100196508	spc24	SPC24, NDC80 kinetochore complex component, homolog (S. cere	29.55	-1.22	-2.33	0.01	0.03
106577590	LOC106577590	histone H2A.Z	587.93	-1.22	-2.33	0.00	0.01
106585835	smarcad1	NA	117.58	-1.22	-2.33	0.00	0.00
100194628	calm2	calmodulin 2	594.47	-1.22	-2.33	0.00	0.00
106610501	LOC106610501	uncharacterized LOC106610501	12.88	-1.22	-2.33	0.00	0.00
106583199	LOC106583199	protein transport protein Sec61 subunit alpha isoform B-like	394.80	-1.22	-2.33	0.00	0.00
106610469	timm10	NA	115.76	-1.22	-2.33	0.00	0.00
106589821	LOC106589821	mitochondrial pyruvate carrier 1-like	481.70	-1.22	-2.33	0.00	0.00
106562380	LOC106562380	HEAT repeat-containing protein 3-like	50.61	-1.22	-2.33	0.00	0.00
106578881	LOC106578881	uncharacterized protein C17orf62 homolog	100.44	-1.22	-2.33	0.00	0.00
106577983	LOC106577983	alpha-crystallin B chain-like	15.07	-1.22	-2.33	0.00	0.00
106578473	LOC106578473	F-box/SPRY domain-containing protein 1-like	9.44	-1.22	-2.33	0.00	0.00
106586236	LOC106586236	cell division cycle-associated protein 7-like	125.89	-1.22	-2.33	0.00	0.00
106575653	LOC106575653	uncharacterized LOC106575653	15.15	-1.22	-2.33	0.00	0.00
100380324	pp2aa	Serine/threonine-protein phosphatase 2A catalytic subunit alpha	153.89	-1.22	-2.32	0.00	0.00
106578814	mrps6	mitochondrial ribosomal protein S6	105.89	-1.21	-2.32	0.00	0.00
106609141	LOC106609141	V-type proton ATPase subunit F	61.95	-1.21	-2.32	0.00	0.00
106584598	gtse1	G2 and S-phase expressed 1	41.21	-1.21	-2.32	0.00	0.00
100196806	g12	Gastrulation-specific protein G12	35.77	-1.21	-2.32	0.00	0.00
100195971	pfd2	Prefoldin subunit 2	301.53	-1.21	-2.32	0.00	0.00
106587773	LOC106587773	transaldolase-like	491.22	-1.21	-2.32	0.00	0.00
106560548	LOC106560548	abnormal spindle-like microcephaly-associated protein homolog	126.65	-1.21	-2.32	0.00	0.00
100196133	abracl	ABRA C-terminal like	663.90	-1.21	-2.31	0.00	0.00
106573223	LOC106573223	NADH-cytochrome b5 reductase 2-like	1532.99	-1.21	-2.31	0.00	0.00
106561604	LOC106561604	aurora kinase B-like	99.37	-1.21	-2.31	0.00	0.01
106564218	LOC106564218	NACHT, LRR and PYD domains-containing protein 6-like	322.24	-1.21	-2.31	0.01	0.02
106564868	LOC106564868	importin subunit alpha-1-like	222.76	-1.21	-2.31	0.00	0.00
100195623	imp3	IMP3, U3 small nucleolar ribonucleoprotein	294.17	-1.21	-2.31	0.00	0.00
100136433	elvol5a	polyunsaturated fatty acid elongase	2373.84	-1.21	-2.31	0.00	0.00
106604799	nanp	N-acetylneuraminic acid phosphatase	22.52	-1.21	-2.31	0.00	0.00

106576202	LOC106576202	ATP synthase subunit gamma, mitochondrial-like	767.80	-1.21	-2.31	0.00	0.00
100196530	dimt1	DIM1 dimethyladenosine transferase 1 homolog	73.39	-1.21	-2.31	0.00	0.00
106610763	rtn4ip1	NA	66.70	-1.21	-2.31	0.00	0.01
106606992	LOC106606992	envoplakin-like	9.66	-1.21	-2.31	0.00	0.00
106602874	LOC106602874	histone H2AX	1106.45	-1.21	-2.31	0.01	0.03
106605288	LOC106605288	uncharacterized LOC106605288	18.84	-1.21	-2.31	0.00	0.00
106586248	LOC106586248	uncharacterized LOC106586248	89.01	-1.20	-2.30	0.00	0.00
106605450	LOC106605450	ras GTPase-activating-like protein IQGAP3	28.07	-1.20	-2.30	0.00	0.00
100196014	psa4	Proteasome subunit alpha type-4	215.07	-1.20	-2.30	0.00	0.00
100195087	sc5d	sterol-C5-desaturase	2760.40	-1.20	-2.30	0.00	0.00
106587076	LOC106587076	transcription factor E2F8-like	21.84	-1.20	-2.30	0.00	0.00
106607651	LOC106607651	activator of 90 kDa heat shock protein ATPase homolog 1-like	246.58	-1.20	-2.30	0.00	0.01
106603037	LOC106603037	bromodomain-containing protein 8-like	16.96	-1.20	-2.30	0.00	0.00
100194750	cdc20	cell division cycle 20	116.05	-1.20	-2.29	0.00	0.01
106608241	LOC106608241	cyclin-K-like	34.73	-1.20	-2.29	0.00	0.00
106607888	LOC106607888	DNA repair protein RAD51 homolog 1	39.82	-1.20	-2.29	0.00	0.00
106577117	LOC106577117	prolyl-tRNA synthetase associated domain-containing protein 1-lil	154.49	-1.20	-2.29	0.00	0.00
106574624	LOC106574624	lipoyltransferase 1, mitochondrial-like	13.36	-1.19	-2.29	0.00	0.00
106603147	LOC106603147	barrier-to-autointegration factor	443.42	-1.19	-2.29	0.00	0.00
106604228	LOC106604228	heat shock 70 kDa protein 4-like	88.07	-1.19	-2.29	0.01	0.03
106609447	LOC106609447	tyrosine 3-monooxygenase-like	10.42	-1.19	-2.29	0.00	0.00
106583637	LOC106583637	Krueppel-like factor 15	97.95	-1.19	-2.29	0.00	0.00
106568201	LOC106568201	coronin-1C-like	33.74	-1.19	-2.29	0.00	0.00
106567831	LOC106567831	transgelin-2-like	45.55	-1.19	-2.29	0.01	0.04
106580795	blmh	bleomycin hydrolase	313.09	-1.19	-2.29	0.00	0.00
106584939	LOC106584939	adipocyte enhancer-binding protein 1-like	7.14	-1.19	-2.29	0.01	0.04
106607585	LOC106607585	ezrin-like	648.49	-1.19	-2.29	0.00	0.00
106602938	LOC106602938	NADH dehydrogenase [ubiquinone] 1 alpha subcomplex subunit 2	162.56	-1.19	-2.28	0.00	0.00
106609956	LOC106609956	uncharacterized LOC106609956	14.47	-1.19	-2.28	0.00	0.00
100196679	LOC100196679	ATP synthase subunit beta, mitochondrial	5548.99	-1.19	-2.28	0.00	0.01
106584489	LOC106584489	peroxisome proliferator-activated receptor alpha-like	68.59	-1.19	-2.28	0.00	0.00
106571922	LOC106571922	proteasome subunit alpha type-7-like	283.75	-1.19	-2.28	0.01	0.04
106599389	LOC106599389	kinesin-like protein KIF2C	94.68	-1.19	-2.28	0.00	0.00
106601733	LOC106601733	cytochrome b-c1 complex subunit 2, mitochondrial-like	125.48	-1.19	-2.28	0.00	0.00
106563616	wdr55	NA	75.82	-1.19	-2.28	0.00	0.01
106612188	LOC106612188	E3 ubiquitin-protein ligase Midline-1-like	8.95	-1.19	-2.28	0.00	0.00
106565058	LOC106565058	eukaryotic translation initiation factor 2 subunit 2-like	1241.61	-1.19	-2.27	0.00	0.00
106566439	LOC106566439	NSFL1 cofactor p47-like	180.81	-1.19	-2.27	0.01	0.04
106580763	LOC106580763	mitochondrial import inner membrane translocase subunit TIM50	100.61	-1.19	-2.27	0.00	0.01
106612836	LOC106612836	isoaspartyl peptidase/L-asparaginase-like	37.82	-1.19	-2.27	0.00	0.00
106564629	LOC106564629	glial fibrillary acidic protein-like	13.75	-1.18	-2.27	0.00	0.00
106583263	LOC106583263	yrdC domain-containing protein, mitochondrial-like	35.44	-1.18	-2.27	0.00	0.00
106611781	LOC106611781	nocturnin-like	8.79	-1.18	-2.27	0.00	0.00
106574234	LOC106574234	keratinocyte-associated protein 2-like	257.44	-1.18	-2.27	0.00	0.00
106588330	LOC106588330	glycosylated lysosomal membrane protein-like	11.49	-1.18	-2.27	0.00	0.00
106565348	LOC106565348	serine/arginine-rich splicing factor 3-like	868.89	-1.18	-2.27	0.00	0.02
106589769	LOC106589769	MORN repeat-containing protein 4-like	73.69	-1.18	-2.27	0.00	0.02
106609350	LOC106609350	PHD finger-like domain-containing protein 5A	61.12	-1.18	-2.27	0.01	0.03
106583769	LOC106583769	uncharacterized LOC106583769	19.20	-1.18	-2.27	0.01	0.03
100195856	nr2c2ap	nuclear receptor 2C2 associated protein	35.39	-1.18	-2.27	0.00	0.00
106603275	LOC106603275	PHD finger protein 12-like	97.69	-1.18	-2.26	0.00	0.00

100195906	ndub7	NADH dehydrogenase 1 beta subcomplex subunit 7	161.00	-1.18	-2.26	0.00	0.00
106589520	LOC106589520	cerebellar degeneration-related protein 2-like	15.69	-1.18	-2.26	0.00	0.00
100196345	cssa01h2orf50	chromosome ssa01 open reading frame, human C2orf50	14.31	-1.18	-2.26	0.01	0.03
106611716	LOC106611716	centromere protein I-like	44.59	-1.18	-2.26	0.00	0.00
106577792	LOC106577792	39S ribosomal protein L52, mitochondrial-like	52.00	-1.18	-2.26	0.00	0.00
106563302	LOC106563302	B box and SPRY domain-containing protein-like	19.69	-1.18	-2.26	0.00	0.00
100380795	LOC100380795	26S proteasome non-ATPase regulatory subunit 11B	296.00	-1.18	-2.26	0.00	0.00
106587097	LOC106587097	CUGBP Elav-like family member 1	335.96	-1.18	-2.26	0.00	0.00
106570634	LOC106570634	syndecan-3-like	23.42	-1.18	-2.26	0.00	0.00
106605360	LOC106605360	U11/U12 small nuclear ribonucleoprotein 48 kDa protein-like	24.55	-1.18	-2.26	0.00	0.02
106590096	ncapg2	non-SMC condensin II complex subunit G2	38.40	-1.18	-2.26	0.00	0.00
106562005	shcbp1	NA	80.71	-1.18	-2.26	0.01	0.04
106597112	LOC106597112	dnaJ homolog subfamily C member 1-like	218.13	-1.17	-2.26	0.00	0.00
100380358	sfrs1	Splicing factor, arginine/serine-rich 1	102.15	-1.17	-2.25	0.01	0.04
100194700	thtpa	thiamine triphosphatase	42.70	-1.17	-2.25	0.00	0.02
106561754	LOC106561754	eukaryotic translation initiation factor 3 subunit J-A-like	257.78	-1.17	-2.25	0.00	0.00
106572599	LOC106572599	SAP domain-containing ribonucleoprotein-like	126.51	-1.17	-2.25	0.00	0.01
100195367	trm61	TRM61 protein	62.15	-1.17	-2.25	0.00	0.01
100306807	egln2	Egl nine homolog 2	165.98	-1.17	-2.25	0.00	0.00
106566647	LOC106566647	uncharacterized LOC106566647	35.29	-1.17	-2.25	0.00	0.00
106572841	LOC106572841	small nuclear ribonucleoprotein G	175.11	-1.17	-2.25	0.00	0.00
106581779	ppig	NA	246.34	-1.17	-2.24	0.00	0.01
106580738	sertad3	NA	7.18	-1.17	-2.24	0.00	0.00
100196476	apitd1	apoptosis-inducing, TAF9-like domain 1	58.95	-1.16	-2.24	0.01	0.03
106608027	ccdc167	coiled-coil domain containing 167	42.70	-1.16	-2.24	0.01	0.03
106579929	LOC106579929	4-hydroxyphenylpyruvate dioxygenase-like	7.51	-1.16	-2.24	0.00	0.01
106588407	LOC106588407	carboxy-terminal kinesin 2-like	153.88	-1.16	-2.24	0.00	0.00
106579789	LOC106579789	uncharacterized LOC106579789	36.60	-1.16	-2.24	0.00	0.00
106578804	LOC106578804	kinesin-like protein KIF20B	42.75	-1.16	-2.24	0.00	0.00
106588652	LOC106588652	uncharacterized LOC106588652	1034.59	-1.16	-2.24	0.01	0.03
106578644	LOC106578644	ubiquitin-like modifier-activating enzyme 5	488.78	-1.16	-2.24	0.01	0.05
106587062	LOC106587062	malignant fibrous histiocytoma-amplified sequence 1-like	48.81	-1.16	-2.24	0.00	0.00
106597138	lyrm4	LYR motif containing 4	82.06	-1.16	-2.24	0.00	0.00
106563191	LOC106563191	S-adenosylmethionine synthase isoform type-2	75.53	-1.16	-2.24	0.00	0.00
106583097	LOC106583097	MICOS complex subunit Mic10-like	156.73	-1.16	-2.23	0.00	0.00
106608146	LOC106608146	kelch repeat and BTB domain-containing protein 11-like	12.99	-1.16	-2.23	0.00	0.01
106575755	LOC106575755	splicing factor U2AF 35 kDa subunit-like	121.95	-1.16	-2.23	0.00	0.00
100196567	dyl1	Dynein light chain 1, cytoplasmic	449.85	-1.16	-2.23	0.01	0.03
106575194	LOC106575194	phospholipase A2 inhibitor 31 kDa subunit-like	2606.66	-1.16	-2.23	0.01	0.04
106585031	LOC106585031	28S ribosomal protein S36, mitochondrial-like	207.98	-1.16	-2.23	0.00	0.00
106565050	LOC106565050	endoplasmic reticulum-Golgi intermediate compartment protein [illegible]	178.29	-1.16	-2.23	0.00	0.00
106571661	LOC106571661	uncharacterized LOC106571661	188.11	-1.16	-2.23	0.00	0.00
100196241	snrpa	small nuclear ribonucleoprotein polypeptide A	144.60	-1.16	-2.23	0.00	0.00
100286592	ndufb3	NADH:ubiquinone oxidoreductase subunit B3	629.85	-1.16	-2.23	0.00	0.00
100194724	LOC100194724	ATP synthase subunit O, mitochondrial	963.40	-1.16	-2.23	0.00	0.00
106573541	LOC106573541	39S ribosomal protein L54, mitochondrial-like	170.95	-1.16	-2.23	0.01	0.03
106570036	psmb2	proteasome subunit beta 2	532.92	-1.16	-2.23	0.00	0.00
100136538	LOC100136538	protein S100-A1	114.75	-1.15	-2.23	0.01	0.03
106602302	LOC106602302	PC4 and SFRS1-interacting protein-like	93.61	-1.15	-2.23	0.00	0.00
106577387	LOC106577387	malate dehydrogenase, cytoplasmic-like	94.02	-1.15	-2.22	0.00	0.00
106573940	sass6	NA	25.28	-1.15	-2.22	0.00	0.00

106604229	LOC106604229	cytochrome b-c1 complex subunit 8-like	194.50	-1.15	-2.22	0.00	0.00
100195375	dpp3	dipeptidyl peptidase 3	480.65	-1.15	-2.22	0.00	0.00
106575027	LOC106575027	mitochondrial ribonuclease P protein 1-like	30.08	-1.15	-2.22	0.00	0.00
106603098	LOC106603098	proteasome subunit beta type-6-like	106.46	-1.15	-2.22	0.00	0.00
106577408	LOC106577408	uncharacterized LOC106577408	53.73	-1.15	-2.22	0.00	0.00
106578474	LOC106578474	negative regulator of reactive oxygen species-like	42.45	-1.15	-2.22	0.01	0.03
106572524	LOC106572524	citrate synthase, mitochondrial-like	311.34	-1.15	-2.22	0.00	0.02
106601073	LOC106601073	hemoglobin subunit beta-1-like	1455.66	-1.15	-2.22	0.00	0.00
106609174	LOC106609174	ADP-ribosylation factor 4-like	11.79	-1.15	-2.22	0.00	0.01
106564953	LOC106564953	insulin-like growth factor-binding protein 4	57.06	-1.15	-2.22	0.01	0.03
100195853	prs10	26S protease regulatory subunit S10B	370.01	-1.15	-2.22	0.00	0.00
106605917	LOC106605917	CKLF-like MARVEL transmembrane domain-containing protein 8	19.33	-1.15	-2.22	0.00	0.00
106583210	LOC106583210	ruvB-like 1	312.59	-1.15	-2.22	0.00	0.01
106607109	ndufa6	NADH:ubiquinone oxidoreductase subunit A6	60.08	-1.15	-2.22	0.00	0.00
106582932	LOC106582932	cell death activator CIDE-3-like	31.11	-1.15	-2.22	0.00	0.00
106569871	LOC106569871	probable 39S ribosomal protein L24, mitochondrial	272.48	-1.15	-2.22	0.00	0.01
106578308	LOC106578308	putative pleckstrin homology domain-containing family M membe	37.22	-1.15	-2.22	0.00	0.00
106577524	ecsit	ECSIT signalling integrator	77.37	-1.15	-2.22	0.00	0.02
106610155	LOC106610155	uncharacterized LOC106610155	12.20	-1.15	-2.22	0.00	0.02
106566902	LOC106566902	beta-catenin-like protein 1	115.13	-1.15	-2.22	0.00	0.01
106588750	LOC106588750	biotinidase-like	32959.69	-1.15	-2.22	0.00	0.01
106604364	LOC106604364	thiosulfate sulfurtransferase/rhodanese-like domain-containing p	193.98	-1.15	-2.22	0.00	0.01
100194708	LOC100194708	aminoacyl tRNA synthase complex-interacting multifunctional pro	144.18	-1.15	-2.21	0.00	0.02
106573277	LOC106573277	fibroblast growth factor 23-like	706.98	-1.15	-2.21	0.00	0.00
100217347	tpm3	tropomyosin 3	243.39	-1.15	-2.21	0.00	0.00
106586674	LOC106586674	inhibitor of growth protein 1-like	34.28	-1.15	-2.21	0.00	0.02
106583419	LOC106583419	prolyl 3-hydroxylase 3-like	55.80	-1.15	-2.21	0.00	0.00
106602126	LOC106602126	structural maintenance of chromosomes protein 2-like	44.09	-1.15	-2.21	0.00	0.00
106590647	LOC106590647	ATP-binding cassette sub-family D member 3	941.89	-1.15	-2.21	0.00	0.00
106571756	LOC106571756	uncharacterized LOC106571756	12.16	-1.14	-2.21	0.01	0.05
106578975	LOC106578975	cone cGMP-specific 3',5'-cyclic phosphodiesterase subunit alpha'-	152.68	-1.14	-2.21	0.00	0.00
100286532	cenph	centromere protein H	23.04	-1.14	-2.21	0.00	0.02
106574151	LOC106574151	PDZ domain-containing protein GIPC3-like	7.46	-1.14	-2.21	0.00	0.00
106583830	LOC106583830	dual specificity protein kinase Ttk-like	69.17	-1.14	-2.21	0.00	0.00
106588903	aunip	aurora kinase A and ninein interacting protein	6.68	-1.14	-2.20	0.00	0.00
106601305	LOC106601305	DNA replication licensing factor mcm5-like	52.97	-1.14	-2.20	0.00	0.00
100194940	esco2	establishment of sister chromatid cohesion N-acetyltransferase 2	72.57	-1.14	-2.20	0.00	0.02
106605109	LOC106605109	replication protein A 32 kDa subunit-like	99.16	-1.14	-2.20	0.00	0.00
106581855	LOC106581855	type-4 ice-structuring protein LS-12-like	27582.30	-1.14	-2.20	0.00	0.02
106561512	LOC106561512	transmembrane protein 168-like	41.35	-1.14	-2.20	0.01	0.03
106598820	b3gnt5	UDP-GlcNAc:betaGal beta-1,3-N-acetylglucosaminyltransferase 5	16.61	-1.14	-2.20	0.00	0.01
106565008	LOC106565008	calpain-1 catalytic subunit-like	418.60	-1.14	-2.20	0.00	0.00
100196438	tba1a	Tubulin alpha-1A chain	236.99	-1.14	-2.20	0.00	0.00
106564267	tecr	NA	439.88	-1.14	-2.20	0.01	0.05
100194810	npl	N-acetylneuraminate pyruvate lyase	163.54	-1.13	-2.20	0.00	0.00
100195269	phop1	Probable phosphatase phospho1	1192.05	-1.13	-2.20	0.00	0.00
106609202	macrod2	MACRO domain containing 2	43.94	-1.13	-2.19	0.00	0.00
106577940	LOC106577940	H/ACA ribonucleoprotein complex subunit 3-like	469.98	-1.13	-2.19	0.00	0.00
106601868	LOC106601868	cyclin-F-like	35.20	-1.13	-2.19	0.00	0.00
106613340	LOC106613340	THAP domain-containing protein 5-like	33.40	-1.13	-2.19	0.00	0.01
100306805	dcam	S-adenosylmethionine decarboxylase proenzyme	1069.38	-1.13	-2.19	0.00	0.00

106606779	LOC106606779	uncharacterized protein C19orf52-like	80.75	-1.13	-2.19	0.00	0.00
106613224	anapc15	anaphase promoting complex subunit 15	76.57	-1.13	-2.19	0.00	0.00
106566865	LOC106566865	pleckstrin homology domain-containing family M member 2-like	113.44	-1.13	-2.19	0.00	0.00
106570202	mtx1	metaxin 1	103.23	-1.13	-2.19	0.00	0.00
106564497	LOC106564497	low-density lipoprotein receptor-like	79.49	-1.13	-2.19	0.00	0.00
106588923	shfm1	split hand/foot malformation (ectrodactyly) type 1	430.51	-1.13	-2.19	0.00	0.00
106587574	LOC106587574	nuclear envelope phosphatase-regulatory subunit 1	157.57	-1.13	-2.19	0.00	0.00
106583717	LOC106583717	translation machinery-associated protein 7-like	188.23	-1.13	-2.19	0.01	0.04
106568974	LOC106568974	growth arrest and DNA damage-inducible protein GADD45 alpha-	26.46	-1.13	-2.19	0.00	0.00
106565437	LOC106565437	cyclin-dependent kinase 2-like	38.50	-1.13	-2.19	0.00	0.02
106582919	LOC106582919	probable G-protein coupled receptor 22	41.26	-1.13	-2.19	0.01	0.03
106566749	LOC106566749	5-aminolevulinate synthase, nonspecific, mitochondrial-like	191.18	-1.13	-2.19	0.00	0.00
106588058	mrps11	mitochondrial ribosomal protein S11	245.76	-1.13	-2.18	0.00	0.00
106579876	cenpe	centromere protein E	155.83	-1.13	-2.18	0.00	0.00
106584507	slc41a2	NA	14.30	-1.13	-2.18	0.00	0.01
100195803	exos4	Exosome complex exonuclease RRP41	110.02	-1.13	-2.18	0.00	0.00
106613952	LOC106613952	mucin-2-like	35.24	-1.12	-2.18	0.00	0.00
106610273	LOC106610273	acidic leucine-rich nuclear phosphoprotein 32 family member B-lil	77.58	-1.12	-2.18	0.00	0.00
106613700	LOC106613700	tax1-binding protein 3-like	42.83	-1.12	-2.18	0.00	0.00
100286678	if4e	Eukaryotic translation initiation factor 4E	308.07	-1.12	-2.18	0.00	0.01
106581362	LOC106581362	von Willebrand factor A domain-containing protein 5A-like	18.10	-1.12	-2.18	0.00	0.02
106612239	LOC106612239	39S ribosomal protein L27, mitochondrial-like	43.64	-1.12	-2.18	0.01	0.03
106589237	adprm	ADP-ribose/CDP-alcohol diphosphatase, manganese dependent	11.30	-1.12	-2.18	0.00	0.01
106566598	LOC106566598	40S ribosomal protein S23	174.00	-1.12	-2.18	0.00	0.01
106563517	LOC106563517	60S acidic ribosomal protein P2-like	2064.81	-1.12	-2.18	0.00	0.00
106575037	mrpl16	mitochondrial ribosomal protein L16	351.81	-1.12	-2.18	0.00	0.00
106611490	LOC106611490	cytochrome c oxidase assembly protein COX16 homolog, mitocho	70.95	-1.12	-2.18	0.00	0.00
106583034	LOC106583034	monocarboxylate transporter 2-like	58.30	-1.12	-2.18	0.00	0.00
106582672	LOC106582672	inhibin beta B chain-like	1540.07	-1.12	-2.17	0.00	0.01
106563511	LOC106563511	protein regulator of cytokinesis 1-like	69.97	-1.12	-2.17	0.00	0.00
106602244	LOC106602244	C-type lectin domain family 4 member M-like	18.18	-1.12	-2.17	0.00	0.00
106583154	LOC106583154	musculoskeletal embryonic nuclear protein 1-like	1011.69	-1.12	-2.17	0.00	0.01
106586371	LOC106586371	coenzyme Q-binding protein COQ10 homolog B, mitochondrial-lik	66.58	-1.12	-2.17	0.00	0.00
106571379	purg	NA	28.85	-1.12	-2.17	0.00	0.00
100136366	LOC100136366	cystic fibrosis transmembrane conductance regulator II	39.02	-1.12	-2.17	0.00	0.00
106583944	LOC106583944	serine/threonine-protein kinase NLK2-like	35.67	-1.12	-2.17	0.00	0.00
106565824	LOC106565824	transmembrane protein 110-like	80.12	-1.11	-2.17	0.01	0.05
100286580	hs020	HSPC020 homolog	32.12	-1.11	-2.17	0.00	0.00
106601377	LOC106601377	major facilitator superfamily domain-containing protein 6-like	115.98	-1.11	-2.17	0.00	0.00
106577403	LOC106577403	sphingomyelin phosphodiesterase 3-like	51.52	-1.11	-2.16	0.00	0.02
106583771	gmppb	GDP-mannose pyrophosphorylase B	442.03	-1.11	-2.16	0.00	0.00
106610653	iqcc	IQ motif containing C	11.47	-1.11	-2.16	0.00	0.00
106609177	LOC106609177	ADP-ribosylation factor-like protein 1	108.12	-1.11	-2.16	0.00	0.00
106581927	fkbp7	FK506 binding protein 7	74.22	-1.11	-2.16	0.00	0.01
106589469	LOC106589469	uncharacterized LOC106589469	17.41	-1.11	-2.16	0.00	0.00
106591939	LOC106591939	histone-arginine methyltransferase CARM1-like	35.63	-1.11	-2.16	0.00	0.01
106605515	LOC106605515	frizzled-1-like	23.05	-1.11	-2.16	0.00	0.00
106566721	LOC106566721	prostaglandin E synthase 3-like	676.06	-1.11	-2.16	0.00	0.01
106586074	ndufaf5	NADH:ubiquinone oxidoreductase complex assembly factor 5	55.63	-1.11	-2.16	0.00	0.00
100380509	prr11	Proline-rich protein 11	42.70	-1.11	-2.16	0.00	0.00
106576205	LOC106576205	filamin-C-like	8.21	-1.11	-2.16	0.00	0.01

106569348	LOC106569348	cyclin-dependent-like kinase 5	30.63	-1.11	-2.16	0.00	0.00
106589036	LOC106589036	mpv17-like protein	157.57	-1.11	-2.16	0.00	0.00
106610844	LOC106610844	rho GTPase-activating protein 11A-like	56.91	-1.11	-2.16	0.00	0.00
106613642	LOC106613642	solute carrier family 35 member E1-like	55.83	-1.11	-2.16	0.00	0.02
100194641	LOC100194641	malate dehydrogenase 2-2, NAD (mitochondrial)	1048.34	-1.11	-2.16	0.01	0.04
106613741	LOC106613741	cold-inducible RNA-binding protein B-like	2882.10	-1.11	-2.16	0.00	0.00
106563393	LOC106563393	H/ACA ribonucleoprotein complex subunit 4-like	435.40	-1.11	-2.16	0.00	0.00
106566373	LOC106566373	ATP synthase F(0) complex subunit B1, mitochondrial-like	834.09	-1.11	-2.16	0.00	0.00
106587846	LOC106587846	fumarylacetoacetase-like	1109.23	-1.11	-2.16	0.00	0.00
106579850	LOC106579850	ubiquitin-like protein 4A-B	115.09	-1.11	-2.16	0.00	0.00
106565677	LOC106565677	methylosome protein 50-like	254.91	-1.11	-2.16	0.00	0.00
106561457	LOC106561457	uncharacterized LOC106561457	8.97	-1.11	-2.16	0.00	0.00
106580643	LOC106580643	ATP-binding cassette sub-family F member 3-like	198.19	-1.11	-2.16	0.00	0.00
106611176	LOC106611176	proteasome subunit alpha type-6-like	551.68	-1.11	-2.16	0.00	0.00
106571471	casc5	kinetochore scaffold 1	78.77	-1.11	-2.15	0.00	0.00
106607556	LOC106607556	prolyl endopeptidase-like	114.79	-1.11	-2.15	0.00	0.00
106573997	LOC106573997	transmembrane gamma-carboxyglutamic acid protein 1-like	31.39	-1.11	-2.15	0.00	0.00
106612556	LOC106612556	succinate dehydrogenase [ubiquinone] cytochrome b small subun	442.02	-1.11	-2.15	0.00	0.00
106584748	LOC106584748	protein regulator of cytokinesis 1-like	21.84	-1.11	-2.15	0.01	0.04
106582373	LOC106582373	uncharacterized LOC106582373	44.59	-1.11	-2.15	0.00	0.00
106605151	LOC106605151	protein argonaute-3-like	31.32	-1.11	-2.15	0.00	0.00
106590589	LOC106590589	arylamine N-acetyltransferase, pineal gland isozyme NAT-10-like	55.70	-1.10	-2.15	0.00	0.00
100196551	cssa28h17orf89	NADH:ubiquinone oxidoreductase complex assembly factor 8	159.52	-1.10	-2.15	0.00	0.00
106569296	LOC106569296	histone H2A.V	618.67	-1.10	-2.15	0.00	0.00
106564271	LOC106564271	histone-arginine methyltransferase CARM1-like	43.09	-1.10	-2.15	0.00	0.00
106610468	mis18bp1	MIS18 binding protein 1	35.20	-1.10	-2.15	0.00	0.00
106588679	LOC106588679	actin-binding protein anillin-like	47.98	-1.10	-2.15	0.00	0.02
106584001	psmg4	NA	79.18	-1.10	-2.15	0.00	0.02
106603739	LOC106603739	high affinity cGMP-specific 3',5'-cyclic phosphodiesterase 9A-like	29.25	-1.10	-2.15	0.01	0.03
106589518	LOC106589518	ER lumen protein-retaining receptor 2	946.49	-1.10	-2.15	0.00	0.01
106587682	LOC106587682	NAD(P) transhydrogenase, mitochondrial-like	42.58	-1.10	-2.15	0.01	0.03
106560346	LOC106560346	proteasome subunit alpha type-7-like	524.16	-1.10	-2.15	0.01	0.02
100286553	LOC100286553	heterogeneous nuclear ribonucleoprotein Q	187.73	-1.10	-2.15	0.00	0.00
100194574	atp5a1	ATP synthase, H+ transporting, mitochondrial F1 complex, alpha s	3365.27	-1.10	-2.15	0.00	0.00
106601617	LOC106601617	uncharacterized LOC106601617	7.96	-1.10	-2.15	0.00	0.01
106600084	LOC106600084	integrin alpha-6-like	88.16	-1.10	-2.15	0.00	0.00
106563236	gtf3c5	general transcription factor IIIC subunit 5	25.54	-1.10	-2.14	0.00	0.00
106607803	arv1	ARV1 homolog, fatty acid homeostasis modulator	12.53	-1.10	-2.14	0.00	0.00
100195154	if2a	Eukaryotic translation initiation factor 2 subunit 1	88.21	-1.10	-2.14	0.00	0.00
106570938	psmb7	proteasome subunit beta 7	1021.09	-1.10	-2.14	0.00	0.01
106606385	LOC106606385	elongation factor Tu, mitochondrial-like	130.42	-1.10	-2.14	0.00	0.01
100196000	fen1	flap structure-specific endonuclease 1	531.60	-1.10	-2.14	0.00	0.00
106588160	LOC106588160	26S proteasome non-ATPase regulatory subunit 4-like	365.61	-1.10	-2.14	0.00	0.01
106562592	LOC106562592	fumarylacetoacetase-like	483.18	-1.10	-2.14	0.01	0.03
106562547	LOC106562547	deoxyuridine 5'-triphosphate nucleotidohydrolase-like	53.82	-1.10	-2.14	0.00	0.00
106566834	LOC106566834	NADH dehydrogenase [ubiquinone] 1 beta subcomplex subunit 11	454.68	-1.10	-2.14	0.00	0.00
106577962	LOC106577962	protein lin-37 homolog	6.47	-1.09	-2.14	0.00	0.00
106583156	LOC106583156	serine/threonine-protein kinase 40	117.12	-1.09	-2.14	0.00	0.00
106578183	LOC106578183	UDP-glucose 4-epimerase-like	326.39	-1.09	-2.14	0.00	0.00
100194994	rir2	Ribonucleoside-diphosphate reductase subunit M2	408.50	-1.09	-2.14	0.00	0.00
106585874	mrpl1	mitochondrial ribosomal protein L1	257.59	-1.09	-2.13	0.00	0.00

106605882	LOC106605882	uncharacterized LOC106605882	11.82	-1.09	-2.13	0.00	0.01
106607257	LOC106607257	116 kDa U5 small nuclear ribonucleoprotein component	108.44	-1.09	-2.13	0.00	0.00
106561669	LOC106561669	vesicle transport protein GOT1B-like	178.39	-1.09	-2.13	0.01	0.04
106586335	LOC106586335	isocitrate dehydrogenase [NADP] cytoplasmic-like	3104.07	-1.09	-2.13	0.00	0.00
106605234	LOC106605234	26S protease regulatory subunit 6B	302.69	-1.09	-2.13	0.00	0.00
106580467	LOC106580467	ran-specific GTPase-activating protein-like	300.18	-1.09	-2.13	0.00	0.00
106564860	LOC106564860	heterogeneous nuclear ribonucleoprotein Q	220.84	-1.09	-2.13	0.00	0.00
100195739	sec13	SEC13 homolog, nuclear pore and COPII coat complex component	521.92	-1.09	-2.13	0.01	0.03
106607688	LOC106607688	cytochrome c oxidase subunit 7A2, mitochondrial	44.04	-1.09	-2.13	0.00	0.00
106569545	LOC106569545	E3 ubiquitin-protein ligase RNF126-like	23.59	-1.09	-2.13	0.00	0.00
100195300	paps2	Bifunctional 3-phosphoadenosine 5-phosphosulfate synthetase 2	627.00	-1.09	-2.13	0.00	0.00
106586369	LOC106586369	60 kDa heat shock protein, mitochondrial	1481.05	-1.09	-2.13	0.00	0.00
106565430	LOC106565430	gelsolin-like	92.19	-1.09	-2.12	0.00	0.02
106563463	LOC106563463	sororin-B-like	53.95	-1.09	-2.12	0.00	0.02
106590477	LOC106590477	transmembrane protein 186-like	22.31	-1.09	-2.12	0.00	0.01
106588333	LOC106588333	uncharacterized LOC106588333	17.24	-1.09	-2.12	0.00	0.00
100286658	lsm2	LSM2 homolog, U6 small nuclear RNA and mRNA degradation ass	271.18	-1.09	-2.12	0.00	0.00
101448048	mrpl17	ribosomal protein L17 mitochondrial-like protein	91.47	-1.08	-2.12	0.01	0.02
106579887	LOC106579887	probable cytosolic iron-sulfur protein assembly protein ciao1-A	260.70	-1.08	-2.12	0.01	0.04
106565458	LOC106565458	coatomer subunit zeta-1	33.67	-1.08	-2.12	0.00	0.01
106572098	LOC106572098	uncharacterized LOC106572098	17.54	-1.08	-2.12	0.00	0.00
100196170	dnjc8	DnaJ homolog subfamily C member 8	177.88	-1.08	-2.12	0.00	0.00
106608798	LOC106608798	cytidine deaminase-like	179.38	-1.08	-2.12	0.00	0.00
106601012	LOC106601012	kelch-like protein 11	10.88	-1.08	-2.11	0.00	0.01
106598934	LOC106598934	growth arrest and DNA damage-inducible protein GADD45 alpha-	21.18	-1.08	-2.11	0.01	0.04
106607785	LOC106607785	dehydrodolichyl diphosphate syntase complex subunit nus1-like	30.61	-1.08	-2.11	0.00	0.00
106603018	adck1	aarF domain containing kinase 1	61.03	-1.08	-2.11	0.01	0.03
106561701	LOC106561701	NADH dehydrogenase [ubiquinone] 1 alpha subcomplex subunit 8	226.45	-1.08	-2.11	0.00	0.00
106577208	LOC106577208	hypoxia-inducible factor 1-alpha inhibitor	106.02	-1.08	-2.11	0.00	0.00
106574730	donson	downstream neighbor of SON	18.24	-1.08	-2.11	0.00	0.01
100196195	rpac2	DNA-directed RNA polymerases I and III subunit RPAC2	94.82	-1.08	-2.11	0.00	0.00
100196837	zg14	Gastric cancer antigen Zg14 homolog	54.78	-1.08	-2.11	0.00	0.00
106589652	LOC106589652	uncharacterized LOC106589652	12.13	-1.08	-2.11	0.00	0.00
100196157	cyh1	Cytohesin-1	101.25	-1.08	-2.11	0.00	0.00
106579978	LOC106579978	multiple epidermal growth factor-like domains protein 9	24.97	-1.08	-2.11	0.00	0.00
100136459	LOC100136459	caspase 8-like protein	13.59	-1.07	-2.11	0.00	0.00
100380718	top2a	NA	462.68	-1.07	-2.11	0.00	0.00
106571510	cssa15h14orf2	chromosome ssa15 open reading frame, human C14orf2	1090.44	-1.07	-2.11	0.00	0.02
106567990	LOC106567990	uncharacterized LOC106567990	16.88	-1.07	-2.11	0.00	0.00
106605046	pwwp2b	NA	119.56	-1.07	-2.10	0.01	0.04
106611778	ndufc1	NADH:ubiquinone oxidoreductase subunit C1	435.93	-1.07	-2.10	0.00	0.00
106576160	LOC106576160	uncharacterized LOC106576160	28.81	-1.07	-2.10	0.01	0.04
106601894	LOC106601894	jmjC domain-containing protein 8-like	50.30	-1.07	-2.10	0.00	0.02
106603001	LOC106603001	UPF0568 protein C14orf166 homolog	236.46	-1.07	-2.10	0.00	0.00
100194754	aldh7a1	aldehyde dehydrogenase 7 family member A1	3261.94	-1.07	-2.10	0.00	0.00
106573564	LOC106573564	mitochondrial import inner membrane translocase subunit Tim13	229.93	-1.07	-2.10	0.00	0.00
106606941	LOC106606941	protein SCO1 homolog, mitochondrial-like	33.27	-1.07	-2.10	0.00	0.00
100195113	ehd3	EH-domain containing 3	142.48	-1.07	-2.10	0.00	0.00
106561917	LOC106561917	oxidoreductase HTATIP2-like	104.37	-1.07	-2.10	0.00	0.00
100195137	prim2	primase (DNA) subunit 2	117.37	-1.07	-2.10	0.00	0.00
106576642	LOC106576642	nuclear pore complex protein Nup107-like	14.30	-1.07	-2.10	0.00	0.00

106564930	LOC106564930	THO complex subunit 4-A-like	139.54	-1.07	-2.10	0.00	0.00
100286640	pole3	DNA polymerase epsilon 3, accessory subunit	79.64	-1.07	-2.10	0.00	0.01
106603813	LOC106603813	CDKN2AIP N-terminal-like protein	113.56	-1.07	-2.10	0.00	0.00
106574550	LOC106574550	ATP synthase subunit beta, mitochondrial	4540.40	-1.07	-2.09	0.01	0.03
100195093	erd22	ER lumen protein retaining receptor 2	328.49	-1.07	-2.09	0.00	0.00
100194941	lancl2	LanC like 2	31.25	-1.07	-2.09	0.00	0.01
100196317	cssa14h1orf123	chromosome ssa14 open reading frame, human C1orf123	193.01	-1.06	-2.09	0.00	0.00
106609471	LOC106609471	V-type proton ATPase subunit E 1-like	55.93	-1.06	-2.09	0.01	0.03
106606979	LOC106606979	THO complex subunit 4-like	174.00	-1.06	-2.09	0.00	0.02
106611965	ptcd3	NA	253.45	-1.06	-2.09	0.00	0.01
106606750	LOC106606750	ER lumen protein-retaining receptor 2-like	207.08	-1.06	-2.09	0.00	0.00
100195716	bca3	Proline-rich protein BCA3	12.22	-1.06	-2.09	0.00	0.02
106606510	LOC106606510	heat shock protein 75 kDa, mitochondrial-like	181.75	-1.06	-2.09	0.00	0.01
106568981	LOC106568981	60S ribosomal protein L5-like	371.17	-1.06	-2.09	0.00	0.01
106602892	mrps31	mitochondrial ribosomal protein S31	255.28	-1.06	-2.09	0.01	0.05
100286676	cenpm	Centromere protein M	23.08	-1.06	-2.09	0.00	0.00
106565174	LOC106565174	hyaluronidase-2-like	180.14	-1.06	-2.09	0.00	0.00
106565051	LOC106565051	cytochrome c1, heme protein, mitochondrial-like	589.96	-1.06	-2.09	0.00	0.02
106562111	LOC106562111	lymphatic vessel endothelial hyaluronic acid receptor 1-like	8.39	-1.06	-2.09	0.00	0.00
106607664	rrm2	NA	129.85	-1.06	-2.09	0.00	0.00
106604719	mrpl11	mitochondrial ribosomal protein L11	399.03	-1.06	-2.09	0.00	0.01
106608573	LOC106608573	voltage-dependent anion-selective channel protein 2-like	1065.87	-1.06	-2.09	0.00	0.00
100195946	rae1	ribonucleic acid export 1	68.00	-1.06	-2.09	0.00	0.00
106573170	LOC106573170	protein C19orf12 homolog	126.96	-1.06	-2.09	0.00	0.00
106585457	LOC106585457	prostaglandin E synthase 2-like	306.58	-1.06	-2.08	0.00	0.00
106562054	dhcr7	7-dehydrocholesterol reductase	1972.23	-1.06	-2.08	0.00	0.00
100194518	gatc	glutamyl-tRNA amidotransferase subunit C	39.09	-1.06	-2.08	0.00	0.00
106611343	LOC106611343	uncharacterized LOC106611343	14.62	-1.06	-2.08	0.00	0.01
100194782	abcf2	ATP-binding cassette, sub-family F (GCN20), member 2	591.46	-1.06	-2.08	0.00	0.00
106562063	LOC106562063	adenine phosphoribosyltransferase-like	637.02	-1.06	-2.08	0.00	0.00
106576031	LOC106576031	ADP-ribosylation factor 4	737.39	-1.06	-2.08	0.00	0.00
106613255	LOC106613255	translocon-associated protein subunit gamma	1090.63	-1.06	-2.08	0.00	0.02
106598425	LOC106598425	angiopoietin-related protein 3-like	8518.91	-1.06	-2.08	0.00	0.01
100196498	tmem126a	transmembrane protein 126A	113.12	-1.06	-2.08	0.00	0.00
106603136	mrpl49	mitochondrial ribosomal protein L49	117.61	-1.06	-2.08	0.00	0.00
100196228	yars	NA	267.93	-1.06	-2.08	0.00	0.02
106579957	atp2a2	ATPase sarcoplasmic/endoplasmic reticulum Ca2+ transporting 2	386.63	-1.06	-2.08	0.00	0.00
106560233	LOC106560233	uncharacterized LOC106560233	10.79	-1.06	-2.08	0.00	0.00
106568661	LOC106568661	tetratricopeptide repeat protein 19, mitochondrial-like	107.88	-1.06	-2.08	0.00	0.00
106588385	LOC106588385	histone H3-like centromeric protein A	55.96	-1.05	-2.08	0.01	0.03
106602944	wdhd1	NA	94.20	-1.05	-2.08	0.01	0.04
106604939	LOC106604939	heterogeneous nuclear ribonucleoprotein A0-like	8.22	-1.05	-2.08	0.00	0.00
106611570	mad2l1bp	MAD2L1 binding protein	26.81	-1.05	-2.08	0.00	0.00
106605533	LOC106605533	lysosomal-associated transmembrane protein 4B-like	9.92	-1.05	-2.08	0.00	0.02
106567640	LOC106567640	histone H2A	8.29	-1.05	-2.08	0.00	0.00
106587585	LOC106587585	39S ribosomal protein L4, mitochondrial-like	222.20	-1.05	-2.08	0.00	0.00
100286672	ndua4	NADH dehydrogenase 1 alpha subcomplex subunit 4	836.68	-1.05	-2.07	0.00	0.00
106601291	LOC106601291	growth arrest and DNA damage-inducible proteins-interacting prc	79.38	-1.05	-2.07	0.00	0.00
106564249	LOC106564249	UPF0390 protein zgc136864-like	143.39	-1.05	-2.07	0.00	0.00
106583218	LOC106583218	uncharacterized LOC106583218	15424.27	-1.05	-2.07	0.00	0.00
106586648	LOC106586648	leucine-rich repeat-containing protein 58-like	363.11	-1.05	-2.07	0.00	0.00

106588685	rpa3	replication protein A3	209.34	-1.05	-2.07	0.00	0.02
106612257	atox1	antioxidant 1 copper chaperone	231.43	-1.05	-2.07	0.00	0.01
106571199	LOC106571199	serine/threonine-protein kinase Nek2-like	23.69	-1.05	-2.07	0.00	0.00
106561682	LOC106561682	small nuclear ribonucleoprotein F-like	173.39	-1.05	-2.07	0.00	0.00
100195558	rnh2c	Ribonuclease H2 subunit C	53.10	-1.05	-2.07	0.00	0.00
100195170	ccna2	cyclin A2	68.92	-1.05	-2.07	0.00	0.00
106587952	LOC106587952	proteasome subunit beta type-3	403.71	-1.05	-2.07	0.00	0.00
106590255	ipo4	importin 4	103.02	-1.05	-2.07	0.01	0.02
106608224	LOC106608224	26S protease regulatory subunit 4-like	238.88	-1.05	-2.07	0.00	0.00
106567171	racgap1	NA	42.25	-1.05	-2.07	0.00	0.00
106568980	LOC106568980	protein FAM69A-like	54.96	-1.05	-2.07	0.00	0.00
106603166	LOC106603166	chromosome-associated kinesin KIF4-like	62.10	-1.05	-2.07	0.01	0.05
106601759	LOC106601759	uncharacterized LOC106601759	45.61	-1.05	-2.07	0.00	0.00
106570923	ndufs4	NADH:ubiquinone oxidoreductase subunit S4	470.53	-1.05	-2.07	0.00	0.00
106582038	LOC106582038	adrenodoxin-like	120.46	-1.05	-2.06	0.00	0.00
106587241	eif3j	eukaryotic translation initiation factor 3 subunit J	193.38	-1.05	-2.06	0.00	0.00
106587073	LOC106587073	transmembrane protein 208-like	41.36	-1.04	-2.06	0.00	0.00
106562275	LOC106562275	diphosphomevalonate decarboxylase-like	300.05	-1.04	-2.06	0.00	0.00
106581253	LOC106581253	SH3 domain-binding protein 5-like	23.93	-1.04	-2.06	0.00	0.00
106560480	wars	NA	173.35	-1.04	-2.06	0.00	0.00
106570619	LOC106570619	calcium-binding mitochondrial carrier protein SCaMC-2-B	10.29	-1.04	-2.06	0.00	0.00
106584632	LOC106584632	nucleobindin-2-like	215.58	-1.04	-2.06	0.00	0.01
106562095	LOC106562095	beta,beta-carotene 15,15'-monooxygenase-like	68.72	-1.04	-2.06	0.00	0.00
100196695	htra1	Serine protease HTRA1	31.45	-1.04	-2.06	0.00	0.00
106587369	LOC106587369	ER membrane protein complex subunit 8-like	64.66	-1.04	-2.06	0.00	0.01
106608339	LOC106608339	PIN2/TERF1-interacting telomerase inhibitor 1-like	38.81	-1.04	-2.06	0.00	0.00
106573152	LOC106573152	uncharacterized LOC106573152	360.15	-1.04	-2.06	0.00	0.01
106580678	LOC106580678	RNA-binding protein FUS-like	538.29	-1.04	-2.06	0.00	0.00
106576109	LOC106576109	transmembrane protein 229A-like	71.81	-1.04	-2.06	0.00	0.02
106608593	LOC106608593	RNA-binding protein 4.1-like	130.27	-1.04	-2.06	0.00	0.01
106600828	LOC106600828	insulin-like growth factor 2 mRNA-binding protein 1	72.28	-1.04	-2.06	0.00	0.00
106589606	rac3	NA	27.97	-1.04	-2.06	0.00	0.01
106599105	arg2	arginase 2	51.89	-1.04	-2.06	0.00	0.00
106605350	LOC106605350	dynactin subunit 3-like	193.02	-1.04	-2.06	0.00	0.00
106564948	LOC106564948	proteasome subunit beta type-3-like	341.99	-1.04	-2.06	0.00	0.00
106563022	LOC106563022	uncharacterized LOC106563022	22.79	-1.04	-2.06	0.00	0.00
106612672	LOC106612672	bone morphogenetic protein 5-like	200.91	-1.04	-2.06	0.01	0.05
106561318	lrrc56	leucine rich repeat containing 56	229.72	-1.04	-2.06	0.00	0.00
106588914	LOC106588914	cytochrome b-c1 complex subunit 7-like	546.56	-1.04	-2.06	0.00	0.00
106578967	LOC106578967	kinesin-like protein KIF20B	22.53	-1.04	-2.06	0.01	0.03
100196835	tm167	Transmembrane protein 167	179.08	-1.04	-2.05	0.00	0.01
106577624	LOC106577624	leucine rich adaptor protein 1-like	204.40	-1.04	-2.05	0.00	0.00
106566285	acad9	acyl-CoA dehydrogenase family member 9	308.35	-1.04	-2.05	0.00	0.00
106575942	LOC106575942	glyceraldehyde-3-phosphate dehydrogenase	4366.57	-1.04	-2.05	0.00	0.00
106605110	mecr	mitochondrial trans-2-enoyl-CoA reductase	153.26	-1.04	-2.05	0.00	0.00
106594936	LOC106594936	nucleolar and spindle-associated protein 1-like	75.50	-1.04	-2.05	0.00	0.01
106609575	LOC106609575	uncharacterized LOC106609575	20.30	-1.04	-2.05	0.00	0.01
106609140	LOC106609140	ADP-ribosylation factor 4	632.65	-1.04	-2.05	0.00	0.00
106578486	LOC106578486	ATP-binding cassette sub-family D member 3-like	1093.76	-1.04	-2.05	0.00	0.00
100196767	sar1a	SAR1 gene homolog A (S. cerevisiae)	1349.87	-1.04	-2.05	0.00	0.01
106570933	LOC106570933	activated RNA polymerase II transcriptional coactivator p15-like	139.50	-1.04	-2.05	0.01	0.04

106613672	LOC106613672	coatomer subunit epsilon-like	722.39	-1.04	-2.05	0.00	0.01
106602378	LOC106602378	signal peptidase complex subunit 3	399.92	-1.04	-2.05	0.00	0.01
106604531	LOC106604531	peptidyl-prolyl cis-trans isomerase D-like	46.53	-1.03	-2.05	0.00	0.00
106580445	LOC106580445	proliferating cell nuclear antigen-like	441.17	-1.03	-2.05	0.00	0.01
106560988	LOC106560988	28S ribosomal protein S35, mitochondrial-like	88.14	-1.03	-2.05	0.00	0.01
106571326	LOC106571326	NADH dehydrogenase [ubiquinone] 1 alpha subcomplex assembly	27.21	-1.03	-2.05	0.00	0.00
106562989	LOC106562989	tubulin alpha chain, testis-specific-like	177.83	-1.03	-2.05	0.00	0.02
100194701	LOC100194701	uncharacterized LOC100194701	605.99	-1.03	-2.05	0.00	0.00
106590108	insig1	insulin induced gene 1	1008.45	-1.03	-2.05	0.00	0.00
106583604	LOC106583604	keratin, type II cytoskeletal 8-like	138.71	-1.03	-2.05	0.00	0.01
106612789	LOC106612789	serine/arginine-rich splicing factor 1B-like	417.44	-1.03	-2.04	0.00	0.00
106580684	gpatch4	G-patch domain containing 4	192.10	-1.03	-2.04	0.00	0.00
106602394	LOC106602394	dCTP pyrophosphatase 1 pseudogene	55.63	-1.03	-2.04	0.00	0.02
106567863	LOC106567863	ribonucleoside-diphosphate reductase large subunit	190.13	-1.03	-2.04	0.00	0.00
106572696	LOC106572696	prostaglandin E synthase 3-like	186.57	-1.03	-2.04	0.00	0.01
100286498	cnih1	cornichon family AMPA receptor auxiliary protein 1	186.97	-1.03	-2.04	0.00	0.00
106573685	LOC106573685	cytochrome c oxidase subunit 7A2, mitochondrial-like	302.33	-1.03	-2.04	0.00	0.00
106611674	LOC106611674	selenoprotein H-like	154.82	-1.03	-2.04	0.01	0.03
106567977	LOC106567977	mitochondrial import inner membrane translocase subunit Tim22	92.21	-1.03	-2.04	0.00	0.00
106605369	LOC106605369	uncharacterized LOC106605369	136.81	-1.03	-2.04	0.00	0.00
106580170	LOC106580170	SWI/SNF-related matrix-associated actin-dependent regulator of c	35.22	-1.03	-2.04	0.00	0.00
100194914	sgk2	serum/glucocorticoid regulated kinase 2	240.07	-1.03	-2.04	0.00	0.01
100306816	ncoa4	nuclear receptor coactivator 4	641.34	-1.03	-2.04	0.00	0.00
106609216	LOC106609216	single-stranded DNA-binding protein, mitochondrial-like	142.42	-1.03	-2.04	0.00	0.00
100195042	ybx2a	Y-box-binding protein 2-A	1089.60	-1.03	-2.04	0.01	0.03
106608829	LOC106608829	transmembrane protein 88-like	188.71	-1.03	-2.04	0.00	0.00
106581567	thrsp	NA	62773.83	-1.03	-2.04	0.00	0.00
106580568	LOC106580568	ATP synthase subunit alpha, mitochondrial-like	2964.76	-1.03	-2.04	0.00	0.02
106571672	LOC106571672	eukaryotic translation initiation factor 2 subunit 1	514.57	-1.03	-2.04	0.00	0.00
106583751	LOC106583751	mesencephalic astrocyte-derived neurotrophic factor-like	1889.77	-1.03	-2.04	0.00	0.00
106576587	LOC106576587	zinc finger protein 420-like	47.82	-1.03	-2.04	0.00	0.00
100196481	pfd1	Prefoldin subunit 1	359.94	-1.03	-2.04	0.00	0.00
100196004	ssna1	SS nuclear autoantigen 1	18.72	-1.03	-2.04	0.00	0.00
106570573	mrpl53	mitochondrial ribosomal protein L53	22.91	-1.03	-2.04	0.00	0.00
100195632	cx32	Gap junction Cx32.2 protein	1839.19	-1.03	-2.04	0.00	0.00
100195188	psf2	DNA replication complex GINS protein PSF2	46.69	-1.02	-2.03	0.01	0.03
106583173	LOC106583173	disco-interacting protein 2 homolog B-A-like	45.60	-1.02	-2.03	0.00	0.00
106577100	LOC106577100	atlastin-2-like	404.08	-1.02	-2.03	0.00	0.00
106562324	LOC106562324	DNA-directed RNA polymerases I, II, and III subunit RPABC5	54.18	-1.02	-2.03	0.01	0.02
106610500	LOC106610500	uncharacterized LOC106610500	17.61	-1.02	-2.03	0.00	0.00
106588468	LOC106588468	claspin-like	69.81	-1.02	-2.03	0.00	0.00
106562237	LOC106562237	nestin-like	93.79	-1.02	-2.03	0.00	0.01
106586502	LOC106586502	serine/arginine repetitive matrix protein 2-like	45.60	-1.02	-2.03	0.01	0.03
100196357	tdx	Peroxiredoxin	6407.12	-1.02	-2.03	0.00	0.00
106580952	LOC106580952	39S ribosomal protein L44, mitochondrial-like	18.19	-1.02	-2.03	0.00	0.00
100196308	dnajc25	DnaJ (Hsp40) homolog, subfamily C , member 25	73.50	-1.02	-2.03	0.00	0.00
100380646	LOC100380646	cat eye syndrome critical region protein 5 homolog	119.51	-1.02	-2.03	0.00	0.01
106583328	LOC106583328	prenylated Rab acceptor protein 1-like	192.33	-1.02	-2.03	0.00	0.00
106565229	LOC106565229	keratin, type I cytoskeletal 18-like	57.68	-1.02	-2.03	0.00	0.00
106569664	LOC106569664	calcium/calmodulin-dependent protein kinase II inhibitor 2-like	16.73	-1.02	-2.03	0.00	0.00
100196124	cc001	CC001 protein	111.05	-1.02	-2.02	0.00	0.01

106564829	LOC106564829	mitochondrial import receptor subunit TOM20 homolog	242.66	-1.02	-2.02	0.00	0.00
100194533	eif4e1a	eukaryotic translation initiation factor 4e 1a	293.19	-1.02	-2.02	0.00	0.00
106603799	LOC106603799	small nuclear ribonucleoprotein Sm D2-like	347.67	-1.02	-2.02	0.01	0.04
106604138	LOC106604138	THO complex subunit 3-like	25.41	-1.02	-2.02	0.01	0.04
100306723	spcs1	signal peptidase complex subunit 1 homolog (S. cerevisiae)	549.07	-1.02	-2.02	0.00	0.00
106613717	LOC106613717	E3 ubiquitin-protein ligase MARCH2	151.64	-1.02	-2.02	0.00	0.01
106609227	LOC106609227	dynamin-1-like protein	11.43	-1.02	-2.02	0.00	0.02
106581844	LOC106581844	RNA-binding protein 8A	413.16	-1.02	-2.02	0.00	0.01
100286654	LOC100286654	peptidyl-prolyl cis-trans isomerase FKBP14	96.14	-1.02	-2.02	0.00	0.00
100195682	md2l1	Mitotic spindle assembly checkpoint protein MAD2A	58.26	-1.01	-2.02	0.00	0.00
106562664	LOC106562664	proteasome subunit alpha type-4-like	183.90	-1.01	-2.02	0.00	0.01
106606640	LOC106606640	60S ribosomal protein L3-like	13.74	-1.01	-2.02	0.00	0.00
106581094	LOC106581094	histone-binding protein RBBP4-like	37.71	-1.01	-2.02	0.00	0.02
106575112	LOC106575112	ATP synthase-coupling factor 6, mitochondrial-like	178.35	-1.01	-2.02	0.00	0.00
106571422	nsl1	NSL1, MIS12 kinetochore complex component	57.51	-1.01	-2.02	0.00	0.00
106580579	aebp1	AE binding protein 1	11.94	-1.01	-2.02	0.00	0.01
100136424	rbx1	ring-box 1	260.91	-1.01	-2.02	0.00	0.00
106570330	LOC106570330	39S ribosomal protein L36, mitochondrial-like	80.72	-1.01	-2.02	0.00	0.00
106575108	LOC106575108	39S ribosomal protein L39, mitochondrial-like	122.63	-1.01	-2.02	0.01	0.03
106610985	kif15	kinesin family member 15	62.43	-1.01	-2.02	0.00	0.00
100195337	tpd52l1	tumor protein D52-like 1	485.80	-1.01	-2.02	0.01	0.04
106613497	LOC106613497	39S ribosomal protein L47, mitochondrial-like	163.32	-1.01	-2.02	0.00	0.00
106587944	LOC106587944	very-long-chain (3R)-3-hydroxyacyl-CoA dehydratase-like	98.59	-1.01	-2.02	0.00	0.00
106585073	LOC106585073	fumarylacetoacetate hydrolase domain-containing protein 2-like	1211.78	-1.01	-2.02	0.00	0.02
106613176	slc25a16	NA	88.40	-1.01	-2.02	0.00	0.01
106582726	LOC106582726	mitochondrial glutamate carrier 1-like	47.75	-1.01	-2.02	0.00	0.00
106570811	LOC106570811	histone H2A.V	339.47	-1.01	-2.02	0.00	0.00
106599520	LOC106599520	long-chain-fatty-acid--CoA ligase 3-like	24.98	-1.01	-2.01	0.00	0.00
106610773	LOC106610773	collagen alpha-1(XII) chain-like	40.77	-1.01	-2.01	0.00	0.00
106568193	LOC106568193	TP53-regulated inhibitor of apoptosis 1-like	32.59	-1.01	-2.01	0.00	0.00
100195041	rbbp4	Histone-binding protein RBBP4	255.47	-1.01	-2.01	0.00	0.00
106590419	LOC106590419	translocon-associated protein subunit alpha-like	547.58	-1.01	-2.01	0.00	0.00
100286615	phs	Pterin-4-alpha-carbinolamine dehydratase	1238.85	-1.01	-2.01	0.00	0.00
100196415	tmm49	Transmembrane protein 49	381.94	-1.01	-2.01	0.00	0.00
106585995	LOC106585995	threonine--tRNA ligase, cytoplasmic pseudogene	193.78	-1.01	-2.01	0.00	0.00
100194614	ppdpf	c20orf149 protein	394.06	-1.01	-2.01	0.00	0.00
106586169	LOC106586169	polypeptide N-acetylgalactosaminyltransferase 5-like	24.43	-1.01	-2.01	0.01	0.05
106563612	LOC106563612	probetacellulin-like	32.05	-1.01	-2.01	0.00	0.00
100196499	estd	S-formylglutathione hydrolase	365.87	-1.01	-2.01	0.00	0.01
106563623	brd8	bromodomain containing 8	11.16	-1.01	-2.01	0.00	0.01
106569220	LOC106569220	protein transport protein Sec61 subunit gamma-like	1531.54	-1.01	-2.01	0.00	0.00
106576117	LOC106576117	NADH dehydrogenase [ubiquinone] 1 alpha subcomplex subunit 5	260.05	-1.01	-2.01	0.00	0.02
106562120	LOC106562120	cytochrome c oxidase subunit 5A, mitochondrial-like	662.48	-1.01	-2.01	0.00	0.02
106561938	LOC106561938	39S ribosomal protein L21, mitochondrial-like	57.96	-1.00	-2.01	0.01	0.03
106565211	LOC106565211	rac GTPase-activating protein 1-like	17.32	-1.00	-2.01	0.00	0.01
106576773	LOC106576773	PWWP domain-containing protein 2B-like	34.60	-1.00	-2.00	0.00	0.00
106603810	atp5j2	ATP synthase, H+ transporting, mitochondrial Fo complex subunit	2643.98	-1.00	-2.00	0.00	0.01
106607432	LOC106607432	nuclear body protein SP140-like protein	76.38	-1.00	-2.00	0.00	0.00
106568029	LOC106568029	60S ribosomal protein L36a-like	423.60	-1.00	-2.00	0.00	0.01
106604938	LOC106604938	heterogeneous nuclear ribonucleoprotein A0-like	162.69	-1.00	-2.00	0.00	0.00
106607812	ccdc25	coiled-coil domain containing 25	351.47	-1.00	-2.00	0.00	0.00

100195585	znf593	zinc finger protein 593	407.79	-1.00	-2.00	0.00	0.00
106605159	LOC106605159	electron transfer flavoprotein subunit beta-like	1319.74	-1.00	-2.00	0.01	0.03
106611241	LOC106611241	mitochondrial import inner membrane translocase subunit Tim9-l	14.59	-1.00	-2.00	0.01	0.03
106571580	LOC106571580	ADP-ribosylation factor 6	839.70	-1.00	-2.00	0.01	0.04
100195599	ube2c	ubiquitin-conjugating enzyme E2C	115.60	-1.00	-2.00	0.00	0.00
106606732	LOC106606732	cytochrome b-c1 complex subunit 2, mitochondrial-like	200.13	-1.00	-2.00	0.00	0.01
106613010	LOC106613010	phosphatidylinositol-binding clathrin assembly protein-like	47.62	-1.00	-2.00	0.00	0.00
106574372	pnpla4	NA	27.69	-1.00	-2.00	0.00	0.00

Ploidy group: Diploid

Up-regulated DEGs in parr compared to smolts

NCBI ID	Gene IDs/Locus	Gene name	baseMean	log2FoldChange	Fold change	pvalue	padj
100195420	pck1	phosphoenolpyruvate carboxykinase 1	51.71	3.26	9.55	0.00	0.03
106566064	LOC106566064	guanine nucleotide-binding protein G(i) subunit alpha-2	84.04	3.17	9.00	0.00	0.00
106566987	LOC106566987	uncharacterized LOC106566987	89.04	2.69	6.46	0.00	0.00
106570052	LOC106570052	phosphatidate phosphatase LPIN1-like	41.89	2.67	6.38	0.00	0.00
106600595	odc1	ornithine decarboxylase 1	1141.83	2.66	6.34	0.00	0.01
106562333	LOC106562333	vitamin D 25-hydroxylase-like	26.93	2.61	6.12	0.00	0.00
106599162	LOC106599162	cholesterol 7-alpha-monooxygenase-like	1614.21	2.48	5.57	0.00	0.00
100194634	4ebp	eukaryotic translation initiation factor 4E binding protein	627.88	2.30	4.94	0.00	0.00
106577763	LOC106577763	erythropoietin-like	8.22	2.26	4.78	0.00	0.00
106573930	LOC106573930	growth arrest and DNA damage-inducible protein GADD4	78.96	2.24	4.72	0.00	0.02
106611197	ankrd9	ankyrin repeat domain 9	184.85	2.22	4.67	0.00	0.01
106600984	LOC106600984	ADP-ribosylation factor-like protein 5B	41.85	2.20	4.59	0.00	0.00
106571601	LOC106571601	type III iodothyronine deiodinase-like	265.07	2.15	4.43	0.00	0.00
106584808	LOC106584808	heterogeneous nuclear ribonucleoprotein L-like	27.68	2.14	4.40	0.00	0.00
100136444	fabp3	fatty acid binding protein 3	53.58	2.11	4.32	0.00	0.00
106604583	LOC106604583	leukocyte cell-derived chemotaxin-2-like	4389.14	2.09	4.26	0.00	0.00
106569990	LOC106569990	intermediate filament family orphan 1-like	15.80	2.08	4.22	0.00	0.00
100136442	ghr1	growth hormone receptor isoform 1 precursor	452.56	2.06	4.17	0.00	0.04
106613803	LOC106613803	C2 calcium-dependent domain-containing protein 4C-like	364.50	2.06	4.16	0.00	0.02
106560267	LOC106560267	ras-like protein family member 11B	67.52	1.99	3.97	0.00	0.04
106572755	LOC106572755	cytochrome P450 2K1-like	1282.88	1.96	3.90	0.00	0.00
106609979	LOC106609979	spermine oxidase-like	126.30	1.96	3.89	0.00	0.00
106568115	LOC106568115	betaine--homocysteine S-methyltransferase 1-like	25864.37	1.96	3.88	0.00	0.00
100196052	fkbp5	FK506 binding protein 5	14.57	1.93	3.81	0.00	0.02
106565112	LOC106565112	cryptochrome-1-like	61.07	1.87	3.65	0.00	0.00
100196184	cish	cytokine inducible SH2 containing protein	221.93	1.87	3.64	0.00	0.00
106572462	LOC106572462	pancreatic progenitor cell differentiation and proliferatio	35.96	1.84	3.58	0.00	0.00
106577211	LOC106577211	DNA damage-inducible transcript 4 protein-like	66.53	1.82	3.54	0.00	0.04
106562806	LOC106562806	CCAAT/enhancer-binding protein alpha-like	450.36	1.77	3.41	0.00	0.00
106601356	LOC106601356	uncharacterized LOC106601356	183.31	1.77	3.40	0.00	0.00
106566600	LOC106566600	complement component C7-like	27.18	1.75	3.36	0.00	0.01
106585014	dnajb5	DnaJ heat shock protein family (Hsp40) member B5	11.84	1.75	3.36	0.00	0.05
106603923	LOC106603923	magnesium transporter NIPA2-like	183.76	1.75	3.36	0.00	0.00
106586529	tsc22d1	NA	73.84	1.74	3.34	0.00	0.01
106588135	LOC106588135	chymotrypsin A-like	19.45	1.70	3.26	0.00	0.02
106584507	slc41a2	NA	30.94	1.70	3.24	0.00	0.02
106571818	slc27a6	NA	298.96	1.67	3.19	0.00	0.00
106611136	LOC106611136	ornithine decarboxylase 1-like	312.79	1.67	3.18	0.00	0.01
106568980	LOC106568980	protein FAM69A-like	71.30	1.66	3.16	0.00	0.00
106580024	LOC106580024	SH2 domain-containing protein 3C-like	39.77	1.65	3.15	0.00	0.03
106601411	LOC106601411	ras-related protein Rap-2a-like	15.40	1.64	3.11	0.00	0.00
106580452	LOC106580452	purpurin-like	40.50	1.63	3.10	0.00	0.02
106611368	LOC106611368	type II iodothyronine deiodinase-like	159.61	1.60	3.03	0.00	0.02
100196225	cbpb1	Carboxypeptidase B	10.79	1.58	2.99	0.00	0.02
106572076	myh7b	myosin heavy chain 7B	7.28	1.56	2.95	0.00	0.00
106577654	LOC106577654	uncharacterized LOC106577654	16.48	1.55	2.92	0.00	0.00
106587531	LOC106587531	vitamin D 25-hydroxylase-like	117.61	1.54	2.92	0.00	0.01
106565346	LOC106565346	peptidyl-prolyl cis-trans isomerase FKBP5-like	393.46	1.54	2.90	0.00	0.00

100196390	saca4	Sperm acrosome membrane-associated protein 4	19.59	1.53	2.89	0.00	0.01
106567948	LOC106567948	diacylglycerol O-acyltransferase 2-like	2038.29	1.53	2.89	0.00	0.01
100195204	s2533	Solute carrier family 25 member 33	91.06	1.53	2.88	0.00	0.04
106584489	LOC106584489	peroxisome proliferator-activated receptor alpha-like	82.77	1.52	2.87	0.00	0.02
106563261	LOC106563261	nucleus accumbens-associated protein 2-like	182.26	1.52	2.87	0.00	0.03
106611397	LOC106611397	uncharacterized LOC106611397	35.73	1.49	2.82	0.00	0.02
106578378	LOC106578378	lipocalin-like	14389.83	1.49	2.81	0.00	0.00
106583156	LOC106583156	serine/threonine-protein kinase 40	135.55	1.49	2.81	0.00	0.04
106567289	LOC106567289	suppressor of cytokine signaling 2-like	136.36	1.47	2.78	0.00	0.03
106562381	LOC106562381	arylamine N-acetyltransferase, pineal gland isozyme NAT	18.19	1.47	2.77	0.00	0.02
106573277	LOC106573277	fibroblast growth factor 23-like	744.11	1.47	2.77	0.00	0.00
100137024	trp-iii	trypsin III	20.01	1.46	2.75	0.00	0.00
106586120	LOC106586120	histone deacetylase 4-like	5.83	1.46	2.75	0.00	0.00
106567009	LOC106567009	probable E3 ubiquitin-protein ligase DTX3	64.25	1.46	2.74	0.00	0.00
106567727	LOC106567727	zinc finger and BTB domain-containing protein 16-A-like	29.26	1.45	2.74	0.00	0.03
106606961	LOC106606961	5-hydroxytryptamine receptor 3A-like	5.20	1.45	2.73	0.00	0.00
106569244	LOC106569244	cadherin-6-like	90.92	1.44	2.72	0.00	0.02
106606191	LOC106606191	monocarboxylate transporter 7-like	13.99	1.43	2.70	0.00	0.04
106571726	LOC106571726	alpha-(1,3)-fucosyltransferase 9-like	51.81	1.42	2.67	0.00	0.00
106569629	LOC106569629	lipoprotein lipase-like	14.58	1.41	2.67	0.00	0.00
106602764	LOC106602764	spermine oxidase-like	168.92	1.40	2.64	0.00	0.01
106572163	LOC106572163	chymotrypsin-like elastase family member 2A	12.57	1.40	2.64	0.00	0.00
106568062	LOC106568062	arsenite methyltransferase-like	451.92	1.40	2.63	0.00	0.02
106566634	LOC106566634	cytosolic sulfotransferase 3-like	25.98	1.39	2.62	0.00	0.00
106585939	LOC106585939	endothelial lipase-like	244.53	1.39	2.61	0.00	0.00
106608090	LOC106608090	CLOCK-interacting pacemaker-like	15.45	1.37	2.59	0.00	0.00
106586588	tmem37	NA	23.37	1.37	2.59	0.00	0.00
106566182	LOC106566182	sodium- and chloride-dependent taurine transporter-like	359.17	1.37	2.58	0.00	0.00
106568987	LOC106568987	volume-regulated anion channel subunit LRRC8D-like	89.53	1.34	2.54	0.00	0.05
106605533	LOC106605533	lysosomal-associated transmembrane protein 4B-like	11.10	1.33	2.52	0.00	0.00
106606194	LOC106606194	monocarboxylate transporter 7-like	16.49	1.33	2.52	0.00	0.02
106612672	LOC106612672	bone morphogenetic protein 5-like	214.76	1.32	2.51	0.00	0.00
106592803	ccdc176	basal body orientation factor 1	11.87	1.32	2.49	0.00	0.00
100194597	ahcy	adenosylhomocysteinase	13029.84	1.32	2.49	0.00	0.00
106565889	LOC106565889	endothelin-converting enzyme 1-like	69.81	1.32	2.49	0.00	0.04
106575940	LOC106575940	cytochrome P450 27C1	198.23	1.31	2.48	0.00	0.00
100195269	phop1	Probable phosphatase phospho1	1230.45	1.31	2.48	0.00	0.00
106588017	LOC106588017	galactose-specific lectin nattectin-like	10.71	1.31	2.48	0.00	0.01
100192341	elovl2	ELOVL fatty acid elongase 2	3339.19	1.30	2.47	0.00	0.00
106589674	ifngr1	interferon gamma receptor 1	10.28	1.30	2.45	0.00	0.02
100194902	cebpa	CCAAT/enhancer binding protein (C/EBP), alpha	545.03	1.29	2.45	0.00	0.05
106578473	LOC106578473	F-box/SPRY domain-containing protein 1-like	9.38	1.29	2.45	0.00	0.00
106590330	LOC106590330	heme oxygenase-like	94.35	1.29	2.44	0.00	0.01
106575194	LOC106575194	phospholipase A2 inhibitor 31 kDa subunit-like	2646.25	1.28	2.43	0.00	0.00
100195160	spon2	spondin 2	17.67	1.28	2.43	0.00	0.01
106589620	LOC106589620	Krueppel-like factor 1	10.74	1.28	2.42	0.00	0.00
106589802	fam195a	MAPK regulated corepressor interacting protein 2	277.21	1.27	2.42	0.00	0.00
106572274	LOC106572274	interferon-related developmental regulator 2-like	203.93	1.27	2.41	0.00	0.01
106606514	LOC106606514	testis-expressed sequence 2 protein-like	10.91	1.27	2.41	0.00	0.00
106599887	LOC106599887	5-beta-cholestane-3-alpha,7-alpha-diol 12-alpha-hydroxy	2750.85	1.27	2.40	0.00	0.04
106609884	LOC106609884	tropomodulin-1-like	46.45	1.27	2.40	0.00	0.00

106565579	LOC106565579	uncharacterized LOC106565579	28.95	1.26	2.40	0.00	0.04
106569334	LOC106569334	HIG1 domain family member 1A, mitochondrial-like	170.72	1.26	2.40	0.00	0.01
106567691	LOC106567691	uncharacterized LOC106567691	62.73	1.26	2.39	0.00	0.00
100306807	egln2	Egl nine homolog 2	154.39	1.25	2.38	0.00	0.02
106612829	LOC106612829	phosphoserine phosphatase-like	208.41	1.24	2.36	0.00	0.00
106560734	faxdc2	fatty acid hydroxylase domain containing 2	3015.39	1.24	2.36	0.00	0.01
106565174	LOC106565174	hyaluronidase-2-like	183.22	1.24	2.36	0.00	0.01
100380506	rqcd1	CCR4-NOT transcription complex subunit 9	139.39	1.24	2.35	0.00	0.00
106602999	LOC106602999	uncharacterized LOC106602999	9.08	1.23	2.34	0.00	0.00
106577008	LOC106577008	bifunctional 3'-phosphoadenosine 5'-phosphosulfate syn	157.46	1.23	2.34	0.00	0.00
106589124	LOC106589124	tribbles homolog 1-like	11.96	1.23	2.34	0.00	0.00
106569711	tceanc2	NA	5.50	1.22	2.33	0.00	0.00
106576205	LOC106576205	filamin-C-like	8.92	1.22	2.33	0.00	0.00
106586842	LOC106586842	interferon alpha/beta receptor 1a-like	6.80	1.22	2.32	0.00	0.00
106604461	LOC106604461	multidrug resistance-associated protein 5-like	1365.71	1.21	2.32	0.00	0.00
106565089	LOC106565089	zinc finger and SCAN domain-containing protein 2-like	26.00	1.21	2.32	0.00	0.00
106565809	LOC106565809	mitochondrial intermembrane space import and assembl	24.90	1.21	2.31	0.00	0.00
106613409	LOC106613409	E3 ubiquitin-protein ligase HERC2-like	11.24	1.21	2.31	0.00	0.00
106609709	LOC106609709	complement C1q-like protein 2	232.02	1.20	2.30	0.00	0.04
100306816	ncoa4	nuclear receptor coactivator 4	633.00	1.19	2.29	0.00	0.05
106569017	LOC106569017	uncharacterized LOC106569017	10.21	1.19	2.29	0.00	0.00
106587712	LOC106587712	proline-serine-threonine phosphatase-interacting protei	9.16	1.19	2.28	0.00	0.02
106571198	LOC106571198	zinc transporter 1-like	332.37	1.19	2.28	0.00	0.00
106571895	LOC106571895	androgen-dependent TFPI-regulating protein-like	68.45	1.19	2.28	0.00	0.01
106590074	LOC106590074	thyroid receptor-interacting protein 11-like	6.23	1.18	2.27	0.00	0.00
106573216	LOC106573216	ras association domain-containing protein 10-like	107.23	1.18	2.26	0.00	0.01
106573152	LOC106573152	uncharacterized LOC106573152	363.83	1.18	2.26	0.00	0.01
106565564	LOC106565564	inositol hexakisphosphate kinase 2-like	774.53	1.17	2.26	0.00	0.00
106587252	LOC106587252	haptoglobin-like	8751.92	1.17	2.26	0.00	0.00
106604578	LOC106604578	sphingosine-1-phosphate lyase 1-like	7.91	1.17	2.26	0.00	0.05
106564748	LOC106564748	39S ribosomal protein L45, mitochondrial-like	8.59	1.17	2.26	0.00	0.00
106574135	LOC106574135	uncharacterized LOC106574135	38.71	1.17	2.25	0.00	0.01
106585451	LOC106585451	calcium-binding mitochondrial carrier protein SCaMC-2-A	11.46	1.17	2.25	0.00	0.03
106609794	LOC106609794	glucoside xylosyltransferase 1-like	46.21	1.17	2.25	0.00	0.04
100196806	g12	Gastrulation-specific protein G12	39.93	1.17	2.24	0.00	0.00
106579710	LOC106579710	decaprenyl-diphosphate synthase subunit 1-like	41.40	1.16	2.24	0.00	0.02
106577505	LOC106577505	apolipoprotein A-IV-like	2111.71	1.16	2.24	0.00	0.01
106585128	LOC106585128	uncharacterized protein C8orf4 homolog	58.26	1.16	2.24	0.00	0.03
106568366	LOC106568366	uncharacterized LOC106568366	1248.05	1.16	2.23	0.00	0.00
106567624	LOC106567624	exocyst complex component 3-like	10.01	1.16	2.23	0.00	0.01
106585173	LOC106585173	BAG family molecular chaperone regulator 4-like	30.71	1.16	2.23	0.00	0.00
106578470	arhgef4	Rho guanine nucleotide exchange factor 4	8.67	1.16	2.23	0.00	0.00
106580732	LOC106580732	HHIP-like protein 1	8.13	1.16	2.23	0.00	0.00
106610155	LOC106610155	uncharacterized LOC106610155	11.89	1.16	2.23	0.00	0.00
100136550	LOC100136550	insulin-like growth factor II	87.00	1.15	2.23	0.00	0.00
106580161	LOC106580161	DNA-directed RNA polymerase III subunit RPC7-like	41.39	1.15	2.23	0.00	0.00
106568833	LOC106568833	frizzled-8-like	29.12	1.15	2.22	0.00	0.00
106609703	LOC106609703	chymotrypsin B-like	7.65	1.15	2.22	0.00	0.01
100380461	ci150	CI150 protein	89.56	1.15	2.22	0.00	0.00
106563191	LOC106563191	S-adenosylmethionine synthase isoform type-2	68.89	1.15	2.22	0.00	0.00
106589769	LOC106589769	MORN repeat-containing protein 4-like	65.64	1.15	2.22	0.00	0.03

106569147	dhx30	DExH-box helicase 30	27.36	1.15	2.22	0.00	0.00
106568345	LOC106568345	arrestin domain-containing protein 3-like	36.14	1.15	2.22	0.00	0.00
106584490	LOC106584490	cyclin-dependent kinase inhibitor 1B-like	66.21	1.15	2.21	0.00	0.00
106580263	LOC106580263	chymotrypsin-like elastase family member 2A	11.59	1.14	2.20	0.00	0.01
106603085	LOC106603085	type II iodothyronine deiodinase-like	140.85	1.14	2.20	0.00	0.03
106591176	LOC106591176	metal transporter CNNM1-like	93.33	1.14	2.20	0.00	0.03
106589464	cssa28h17orf62	chromosome ssa28 open reading frame, human C17orf62	127.09	1.13	2.20	0.00	0.00
106565463	LOC106565463	nuclear receptor subfamily 1 group D member 1-like	119.03	1.13	2.19	0.00	0.00
106590162	LOC106590162	transcriptional regulator Myc	51.25	1.13	2.19	0.00	0.00
106607849	LOC106607849	dnaJ homolog subfamily C member 5-like	59.08	1.13	2.19	0.00	0.01
106562855	LOC106562855	uncharacterized LOC106562855	19.62	1.13	2.18	0.00	0.00
106569090	LOC106569090	Krueppel-like factor 4	9.45	1.13	2.18	0.00	0.00
106564655	LOC106564655	transcription elongation factor, mitochondrial-like	11.84	1.13	2.18	0.00	0.00
106571490	LOC106571490	serine protease 23-like	34.62	1.12	2.18	0.00	0.00
106562960	LOC106562960	protein phosphatase 1 regulatory subunit 26-like	5.44	1.12	2.18	0.00	0.00
106584550	LOC106584550	alpha-aminoadipic semialdehyde synthase, mitochondria	1497.99	1.12	2.18	0.00	0.00
106582467	LOC106582467	cytochrome c	395.97	1.12	2.18	0.00	0.01
106569553	LOC106569553	yjeF N-terminal domain-containing protein 3-like	10.01	1.12	2.17	0.00	0.00
106577800	LOC106577800	tripartite motif-containing protein 34A-like	36.47	1.12	2.17	0.00	0.00
106587269	LOC106587269	transcription elongation factor, mitochondrial-like	6.41	1.12	2.17	0.00	0.00
106575027	LOC106575027	mitochondrial ribonuclease P protein 1-like	27.15	1.12	2.17	0.00	0.00
106598934	LOC106598934	growth arrest and DNA damage-inducible protein GADD4	20.19	1.12	2.17	0.00	0.00
106566781	LOC106566781	solute carrier family 26 member 6-like	13.36	1.11	2.16	0.00	0.00
106564267	tecr	NA	399.02	1.11	2.16	0.00	0.00
106611781	LOC106611781	nocturnin-like	8.15	1.11	2.16	0.00	0.00
106563612	LOC106563612	probetacellulin-like	31.88	1.10	2.15	0.00	0.00
106564532	LOC106564532	nuclear factor interleukin-3-regulated protein-like	105.35	1.10	2.15	0.00	0.00
106581861	LOC106581861	uncharacterized LOC106581861	40.53	1.10	2.15	0.00	0.00
106578457	LOC106578457	uncharacterized LOC106578457	121.56	1.10	2.15	0.00	0.00
106612955	LOC106612955	tsukushin-like	385.49	1.10	2.14	0.00	0.00
106564859	LOC106564859	N-acetyllactosaminide beta-1,3-N-acetylglucosaminyltrar	32.37	1.10	2.14	0.00	0.01
100380357	s26a2	Sulfate transporter	46.44	1.10	2.14	0.00	0.00
106579105	LOC106579105	glycylpeptide N-tetradecanoyltransferase 1-like	12.33	1.09	2.13	0.00	0.00
106609524	pkp2	NA	23.51	1.09	2.13	0.00	0.00
106580774	LOC106580774	tubulin-specific chaperone cofactor E-like protein	37.28	1.09	2.12	0.00	0.00
106580382	LOC106580382	ubiquitin-conjugating enzyme E2 D4	30.82	1.09	2.12	0.00	0.00
106602596	LOC106602596	tropomodulin-1-like	51.04	1.09	2.12	0.00	0.00
106583947	LOC106583947	inactive serine protease 35-like	13.47	1.08	2.12	0.00	0.02
106604687	LOC106604687	nocturnin-like	125.06	1.08	2.12	0.00	0.00
106603348	LOC106603348	mitochondrial uncoupling protein 2	593.15	1.08	2.12	0.00	0.00
106565623	LOC106565623	ethanolamine kinase 1-like	129.83	1.08	2.11	0.00	0.00
106612251	LOC106612251	solute carrier family 25 member 48-like	28.38	1.08	2.11	0.00	0.01
106611534	LOC106611534	glutathione S-transferase P-like	11.13	1.08	2.11	0.00	0.04
106589887	LOC106589887	fatty acid desaturase 6-like	250.68	1.08	2.11	0.00	0.00
106563018	LOC106563018	phosphatidylinositol 3-kinase regulatory subunit alpha-lik	1242.83	1.08	2.11	0.00	0.00
100196111	apoa1	apolipoprotein A-I	49023.85	1.07	2.10	0.00	0.00
106576588	LOC106576588	oocyte zinc finger protein XlCOF6-like	233.77	1.07	2.10	0.00	0.00
106603033	LOC106603033	CXXC-type zinc finger protein 5-like	23.18	1.06	2.09	0.00	0.00
106565430	LOC106565430	gelsolin-like	80.91	1.06	2.09	0.00	0.04
106613963	LOC106613963	transcription factor AP-1-like	407.02	1.06	2.08	0.00	0.05
106578486	LOC106578486	ATP-binding cassette sub-family D member 3-like	996.31	1.06	2.08	0.00	0.01

106569545	LOC106569545	E3 ubiquitin-protein ligase RNF126-like	20.44	1.06	2.08	0.00	0.00
106607726	LOC106607726	NADH dehydrogenase [ubiquinone] 1 alpha subcomplex a	25.94	1.06	2.08	0.00	0.00
100380627	LOC100380627	neuronal pentraxin-1	55.80	1.05	2.07	0.00	0.00
106605689	LOC106605689	apolipoprotein Eb-like	8540.21	1.05	2.07	0.00	0.01
106608500	LOC106608500	acyl-CoA desaturase-like	581.65	1.05	2.07	0.00	0.01
106568897	LOC106568897	dihydropyrimidine dehydrogenase [NADP(+)]	2090.21	1.05	2.07	0.00	0.03
106577462	LOC106577462	trypsin-like	8.76	1.05	2.07	0.00	0.05
106588330	LOC106588330	glycosylated lysosomal membrane protein-like	10.15	1.05	2.07	0.00	0.04
106612131	cystm1	cysteine rich transmembrane module containing 1	16.54	1.04	2.06	0.00	0.00
106584709	LOC106584709	cytochrome c oxidase subunit 5A, mitochondrial-like	409.83	1.04	2.06	0.00	0.00
106590153	LOC106590153	transcription factor Sox-17-alpha-A-like	11.79	1.04	2.06	0.00	0.04
106612265	LOC106612265	TSC22 domain family protein 3-like	449.86	1.04	2.05	0.00	0.00
106569447	LOC106569447	uncharacterized LOC106569447	15.06	1.03	2.05	0.00	0.00
106584632	LOC106584632	nucleobindin-2-like	201.99	1.03	2.05	0.00	0.01
106587180	LOC106587180	cytochrome b5-like	426.76	1.03	2.05	0.00	0.00
106599706	LOC106599706	cadherin-2-like	820.12	1.03	2.04	0.00	0.00
106576773	LOC106576773	PWWP domain-containing protein 2B-like	31.37	1.03	2.04	0.00	0.03
106602919	LOC106602919	putative monooxygenase p33MONOX	305.95	1.03	2.04	0.00	0.00
106567558	LOC106567558	uncharacterized LOC106567558	863.99	1.03	2.04	0.00	0.04
106607787	LOC106607787	Golgi-associated PDZ and coiled-coil motif-containing prc	9.76	1.03	2.04	0.00	0.00
106603870	LOC106603870	cytoplasmic polyadenylation element-binding protein 4-l	247.89	1.03	2.04	0.00	0.00
106573007	LOC106573007	3-beta-hydroxysteroid-Delta(8),Delta(7)-isomerase-like	157.28	1.03	2.04	0.00	0.01
100136433	elvol5a	polyunsaturated fatty acid elongase	1971.26	1.02	2.03	0.00	0.00
106560877	LOC106560877	monocarboxylate transporter 2-like	31.36	1.02	2.03	0.00	0.00
106565778	LOC106565778	zinc fingers and homeoboxes protein 3-like	14.86	1.02	2.03	0.00	0.03
106606413	LOC106606413	lysoplasmalogenase-like	647.27	1.02	2.03	0.00	0.02
106593767	LOC106593767	zinc finger protein 883-like	103.78	1.02	2.03	0.00	0.00
106591668	LOC106591668	very-long-chain enoyl-CoA reductase-like	33.19	1.02	2.02	0.00	0.01
106612619	LOC106612619	kelch-like protein 12	23.62	1.01	2.02	0.00	0.00
100380829	tf2l1	Transcription factor CP2-like protein 1	1159.25	1.01	2.02	0.00	0.00
106584447	LOC106584447	protein QIL1-like	141.79	1.01	2.02	0.00	0.01
100196783	eki1	Ethanolamine kinase 1	3325.37	1.01	2.02	0.00	0.01
106575197	sts	NA	252.35	1.01	2.01	0.00	0.00
106584028	LOC106584028	angiopoietin-related protein 3-like	2727.62	1.01	2.01	0.00	0.00
106584725	slc27a2	NA	2732.84	1.01	2.01	0.00	0.02
106613700	LOC106613700	tax1-binding protein 3-like	36.98	1.01	2.01	0.00	0.00
100195866	tryp	Trypsin	30.90	1.01	2.01	0.00	0.01
106590647	LOC106590647	ATP-binding cassette sub-family D member 3	806.99	1.00	2.00	0.00	0.02
106610324	LOC106610324	E3 ubiquitin-protein ligase Topors-like	19.35	1.00	2.00	0.00	0.00

Down-regulated DEGs in parr compared to smolts

NCBI ID	Gene locus	Gene name	baseMean	log2FoldChange	Fold change	pvalue	padj
106564531	LOC106564531	GATA zinc finger domain-containing protein 10-like	130.17	-2.43	-5.41	0.00	0.00
106563358	LOC106563358	C-C motif chemokine 19-like	23.04	-2.40	-5.28	0.00	0.00
106607153	LOC106607153	interferon-induced very large GTPase 1-like	138.92	-2.34	-5.06	0.00	0.00
106583167	LOC106583167	class E basic helix-loop-helix protein 40-like	333.34	-2.05	-4.13	0.00	0.00
100196194	scyb7	Platelet basic protein	13.42	-2.04	-4.12	0.00	0.00
106604926	LOC106604926	early growth response protein 1-like	102.60	-2.02	-4.04	0.00	0.00
106560485	LOC106560485	ficolin-1-like	710.63	-1.97	-3.92	0.00	0.01
106575521	LOC106575521	uncharacterized LOC106575521	16.71	-1.97	-3.91	0.00	0.00
100136548	irf-1	interferon regulatory factor 1	165.01	-1.93	-3.80	0.00	0.01
106585857	LOC106585857	arrestin domain-containing protein 3-like	905.07	-1.84	-3.57	0.00	0.00

106565855	LOC106565855	C-C motif chemokine 19-like	155.38	-1.80	-3.48	0.00	0.01
100194767	pim1	pim-1 oncogene	155.30	-1.74	-3.35	0.00	0.00
106561069	LOC106561069	protein FAM60A-like	16.87	-1.73	-3.31	0.00	0.00
106560487	angptl3	angiopoietin like 3	251.66	-1.72	-3.29	0.00	0.01
106589615	LOC106589615	filaggrin-2-like	23.52	-1.71	-3.28	0.00	0.00
106570607	LOC106570607	E3 ubiquitin-protein ligase ZNRF2-like	15.89	-1.69	-3.23	0.00	0.01
106582240	LOC106582240	interferon-induced guanylate-binding protein 1-like	84.00	-1.69	-3.22	0.00	0.00
106585649	LOC106585649	complement C1q-like protein 2	535.77	-1.69	-3.22	0.00	0.00
106603906	LOC106603906	early growth response protein 1-like	214.80	-1.68	-3.21	0.00	0.00
106581629	LOC106581629	uncharacterized LOC106581629	5.81	-1.66	-3.16	0.00	0.00
106565029	LOC106565029	uncharacterized LOC106565029	19.83	-1.66	-3.16	0.00	0.00
106586891	LOC106586891	interferon-induced GTP-binding protein Mx2-like	7.38	-1.66	-3.15	0.00	0.00
106583431	LOC106583431	interferon-induced protein 44-like	99.58	-1.65	-3.15	0.00	0.00
106602156	LOC106602156	uncharacterized LOC106602156	10.71	-1.65	-3.15	0.00	0.02
106583433	LOC106583433	interferon-induced protein 44-like	20.66	-1.65	-3.13	0.00	0.05
106567335	LOC106567335	zinc finger protein 395-like	157.60	-1.63	-3.10	0.00	0.00
106576872	LOC106576872	C-X-C motif chemokine 9-like	155.02	-1.61	-3.04	0.00	0.00
106575578	LOC106575578	uncharacterized LOC106575578	167.98	-1.60	-3.03	0.00	0.00
106611988	LOC106611988	LON peptidase N-terminal domain and RING finger prote	61.26	-1.56	-2.95	0.00	0.01
106564204	LOC106564204	saposin-C-like	9.58	-1.56	-2.94	0.00	0.01
106565734	LOC106565734	probable G-protein coupled receptor 173	166.11	-1.55	-2.93	0.00	0.00
106583939	LOC106583939	nuclear protein 1-like	2180.11	-1.54	-2.91	0.00	0.00
100196737	tri39	Tripartite motif-containing protein 39	32.12	-1.54	-2.91	0.00	0.02
106567759	LOC106567759	uncharacterized LOC106567759	62.69	-1.50	-2.83	0.00	0.00
100196207	tnr5	Tumor necrosis factor receptor superfamily member 5	81.92	-1.47	-2.78	0.00	0.01
106577467	LOC106577467	procollagen C-endopeptidase enhancer 2-like	32.60	-1.47	-2.77	0.00	0.00
106564129	LOC106564129	uncharacterized LOC106564129	105.11	-1.47	-2.77	0.00	0.00
106577111	LOC106577111	forkhead box protein G1-like	5.90	-1.46	-2.76	0.00	0.00
106568147	LOC106568147	dual specificity protein phosphatase 4-like	13.33	-1.46	-2.76	0.00	0.02
106571437	LOC106571437	uncharacterized LOC106571437	7.48	-1.46	-2.75	0.00	0.03
106583358	LOC106583358	isocitrate dehydrogenase [NAD] subunit beta, mitochonc	38.34	-1.46	-2.75	0.00	0.00
106610706	LOC106610706	antimicrobial peptide NK-lysin-like	6.35	-1.46	-2.75	0.00	0.01
106573758	LOC106573758	choline kinase alpha-like	306.80	-1.44	-2.72	0.00	0.01
106565027	LOC106565027	CMP-N-acetylneuraminate-beta-galactosamide-alpha-2,3	343.09	-1.44	-2.71	0.00	0.00
106583959	LOC106583959	uncharacterized LOC106583959	5.93	-1.44	-2.71	0.00	0.00
106575700	LOC106575700	uncharacterized LOC106575700	32.43	-1.44	-2.70	0.00	0.02
106576861	LOC106576861	uncharacterized LOC106576861	11.01	-1.41	-2.66	0.00	0.05
106573182	LOC106573182	protein bicaudal D homolog 1-like	35.60	-1.40	-2.64	0.00	0.00
100306791	i12r2	Interleukin-12 receptor beta-2 chain	13.45	-1.40	-2.63	0.00	0.01
106574341	LOC106574341	insulin receptor substrate 2-like	132.92	-1.39	-2.62	0.00	0.00
100195250	typh	Thymidine phosphorylase	136.51	-1.39	-2.62	0.00	0.02
106560813	LOC106560813	choline kinase alpha-like	52.11	-1.39	-2.61	0.00	0.00
106566489	LOC106566489	scavenger receptor cysteine-rich type 1 protein M130-lik	8.88	-1.38	-2.61	0.00	0.04
106586142	LOC106586142	signal transducer and activator of transcription 1-alpha/b	46.51	-1.38	-2.61	0.00	0.00
100286536	cl012	CL012 protein	291.21	-1.38	-2.61	0.00	0.01
106582432	LOC106582432	trace amine-associated receptor 13c-like	55.56	-1.38	-2.60	0.00	0.01
106585863	rasef	NA	51.02	-1.37	-2.58	0.00	0.02
106577279	LOC106577279	interferon-induced very large GTPase 1-like	33.13	-1.36	-2.57	0.00	0.00
106589454	LOC106589454	probable ATP-dependent RNA helicase DDX17	33.40	-1.35	-2.56	0.00	0.02
106589292	LOC106589292	eomesodermin-like	7.65	-1.35	-2.55	0.00	0.01
106601284	LOC106601284	proton-coupled folate transporter-like	5.72	-1.33	-2.51	0.00	0.01

106578984	LOC106578984	uncharacterized LOC106578984	12.83	-1.32	-2.49	0.00	0.00
106586170	LOC106586170	cytohesin-interacting protein-like	13.27	-1.32	-2.49	0.00	0.01
106606968	LOC106606968	butyrophilin subfamily 1 member A1-like	5.88	-1.31	-2.49	0.00	0.00
106581655	LOC106581655	P2Y purinoceptor 8-like	6.36	-1.31	-2.48	0.00	0.00
106577783	LOC106577783	putative E3 ubiquitin-protein ligase ARI6	6.78	-1.31	-2.48	0.00	0.00
106578083	LOC106578083	basic leucine zipper transcriptional factor ATF-like 2	6.78	-1.30	-2.47	0.00	0.00
106613695	LOC106613695	lipoprotein lipase-like	889.80	-1.30	-2.47	0.00	0.00
106590411	LOC106590411	interferon regulatory factor 8-like	97.03	-1.30	-2.47	0.00	0.00
106564218	LOC106564218	NACHT, LRR and PYD domains-containing protein 6-like	112.21	-1.30	-2.47	0.00	0.00
106577762	LOC106577762	perforin-1-like	18.47	-1.30	-2.46	0.00	0.01
106567906	LOC106567906	neuronal acetylcholine receptor subunit alpha-9-like	5.22	-1.30	-2.46	0.00	0.00
106580938	LOC106580938	uncharacterized LOC106580938	5.34	-1.29	-2.45	0.00	0.00
106565108	LOC106565108	bombesin receptor-activated protein C6orf89 homolog	593.98	-1.29	-2.45	0.00	0.00
106587845	LOC106587845	major histocompatibility complex class I-related gene prc	20.67	-1.29	-2.45	0.00	0.01
106576222	LOC106576222	uncharacterized LOC106576222	44.46	-1.29	-2.44	0.00	0.00
106563993	LOC106563993	Golgi apparatus membrane protein TVP23 homolog A-lik	6.79	-1.29	-2.44	0.00	0.00
106602581	LOC106602581	lectin-like	25.51	-1.28	-2.43	0.00	0.04
106605366	LOC106605366	CD83 antigen-like	30.47	-1.28	-2.43	0.00	0.00
106565156	LOC106565156	uncharacterized LOC106565156	25.10	-1.28	-2.43	0.00	0.02
106588908	LOC106588908	zinc finger FYVE domain-containing protein 1-like	97.99	-1.28	-2.43	0.00	0.01
106577998	LOC106577998	protein NDRG2-like	1160.61	-1.28	-2.43	0.00	0.01
106570833	LOC106570833	titin-like	215.24	-1.27	-2.41	0.00	0.00
100195695	urah	HIU hydrolase	70.38	-1.27	-2.41	0.00	0.01
106560732	LOC106560732	membrane-associated guanylate kinase, WW and PDZ dc	120.94	-1.27	-2.41	0.00	0.00
106568351	LOC106568351	protein prune homolog 2-like	77.22	-1.27	-2.41	0.00	0.02
106611637	LOC106611637	polycomb group RING finger protein 3	51.44	-1.27	-2.41	0.00	0.00
106576109	LOC106576109	transmembrane protein 229A-like	26.25	-1.26	-2.40	0.00	0.00
106605364	LOC106605364	CD83 antigen-like	17.70	-1.26	-2.39	0.00	0.02
106575706	LOC106575706	uncharacterized LOC106575706	32.28	-1.26	-2.39	0.00	0.02
100136371	LOC100136371	vitamin D3 receptor B	56.47	-1.26	-2.39	0.00	0.01
106576926	tmem72	NA	22.89	-1.25	-2.38	0.00	0.05
106612301	LOC106612301	uncharacterized LOC106612301	5.70	-1.25	-2.38	0.00	0.03
106602716	LOC106602716	pleckstrin homology domain-containing family H membe	19.23	-1.25	-2.38	0.00	0.00
106603875	LOC106603875	CREB3 regulatory factor-like	150.14	-1.25	-2.38	0.00	0.00
106590377	LOC106590377	NEDD8 ultimate buster 1-like	31.57	-1.25	-2.37	0.00	0.00
106573833	LOC106573833	sphingosine 1-phosphate receptor 4-like	7.27	-1.25	-2.37	0.00	0.02
106562736	LOC106562736	b(0,+)-type amino acid transporter 1-like	13.00	-1.24	-2.36	0.00	0.03
106562007	LOC106562007	BCL2/adenovirus E1B 19 kDa protein-interacting protein	579.13	-1.23	-2.35	0.00	0.00
100194912	angl4	Angiopoietin-related protein 4	374.08	-1.23	-2.35	0.00	0.00
106564953	LOC106564953	insulin-like growth factor-binding protein 4	19.44	-1.23	-2.34	0.00	0.04
106573445	LOC106573445	uncharacterized LOC106573445	97.04	-1.22	-2.34	0.00	0.05
106612462	LOC106612462	uncharacterized LOC106612462	71.83	-1.22	-2.33	0.00	0.00
106568527	LOC106568527	cyclin-G2-like	38.81	-1.22	-2.32	0.00	0.00
106600740	LOC106600740	linker for activation of T-cells family member 1-like	44.87	-1.21	-2.32	0.00	0.00
106579933	LOC106579933	gamma-glutamyltranspeptidase 1-like	18.94	-1.21	-2.32	0.00	0.03
106605019	LOC106605019	cysteine sulfinic acid decarboxylase-like	142.58	-1.21	-2.31	0.00	0.01
106588235	LOC106588235	GTP-binding protein Rhes pseudogene	27.83	-1.21	-2.31	0.00	0.01
106602643	LOC106602643	lectin-like	8.12	-1.20	-2.31	0.00	0.01
106572200	tp73	NA	17.16	-1.20	-2.30	0.00	0.00
106577579	LOC106577579	protein NLRC3-like	69.88	-1.20	-2.30	0.00	0.00
100846970	irf1-2	interferon regulatory factor 1 isoform 2	163.67	-1.20	-2.29	0.00	0.01

106605986	LOC106605986	RUN domain-containing protein 3B-like	13.91	-1.20	-2.29	0.00	0.00
106585878	LOC106585878	C-C motif chemokine 19-like	42.78	-1.20	-2.29	0.00	0.00
106561608	LOC106561608	SH2 domain-containing protein 3C-like	5.40	-1.19	-2.29	0.00	0.04
106606554	LOC106606554	poly [ADP-ribose] polymerase 14-like	17.49	-1.19	-2.29	0.00	0.02
106605567	LOC106605567	C-X-C chemokine receptor type 3-like	6.45	-1.19	-2.28	0.00	0.00
106578021	LOC106578021	purine nucleoside phosphorylase-like	146.64	-1.19	-2.28	0.00	0.01
106592827	LOC106592827	uncharacterized LOC106592827	5.46	-1.19	-2.28	0.00	0.00
106590089	LOC106590089	protein phosphatase 1 regulatory subunit 3G-like	329.51	-1.19	-2.28	0.00	0.00
100380534	bat1	HLA-B associated transcript 1	20.50	-1.18	-2.27	0.00	0.03
106598137	LOC106598137	uncharacterized LOC106598137	39.83	-1.18	-2.27	0.00	0.01
106602655	LOC106602655	SLAM family member 5-like	9.66	-1.18	-2.27	0.00	0.01
106575701	LOC106575701	uncharacterized LOC106575701	22.38	-1.18	-2.27	0.00	0.02
106582864	LOC106582864	class E basic helix-loop-helix protein 40-like	190.75	-1.18	-2.27	0.00	0.02
106572213	LOC106572213	ERBB receptor feedback inhibitor 1-like	19.97	-1.18	-2.27	0.00	0.00
106609917	LOC106609917	uncharacterized LOC106609917	5.35	-1.18	-2.26	0.00	0.00
106573381	LOC106573381	genetic suppressor element 1-like	36.90	-1.18	-2.26	0.00	0.00
106571131	LOC106571131	zinc finger and BTB domain-containing protein 25-like	18.85	-1.18	-2.26	0.00	0.02
106606790	LOC106606790	Ig mu chain C region membrane-bound form-like	20.26	-1.18	-2.26	0.00	0.00
106600565	LOC106600565	calcium-binding mitochondrial carrier protein SCaMC-1-li	10.04	-1.17	-2.26	0.00	0.00
106608311	LOC106608311	SLC2A4 regulator-like	41.09	-1.17	-2.25	0.00	0.00
100195746	cd37	Leukocyte antigen CD37	14.30	-1.17	-2.25	0.00	0.00
106603178	LOC106603178	protein eva-1 homolog A-like	5.32	-1.17	-2.25	0.00	0.00
106588224	LOC106588224	programmed cell death 1 ligand 1-like	6.71	-1.17	-2.25	0.00	0.00
106567151	LOC106567151	CDP-diacylglycerol--serine O-phosphatidyltransferase-like	18.34	-1.17	-2.25	0.00	0.00
106584741	myo5c	myosin VC	83.05	-1.17	-2.25	0.00	0.00
106578860	LOC106578860	protein shisa-6 homolog	18.25	-1.16	-2.24	0.00	0.02
106601734	vwa3a	NA	21.67	-1.16	-2.24	0.00	0.00
106575540	LOC106575540	T-cell surface antigen CD2-like	14.24	-1.16	-2.24	0.00	0.00
106560723	LOC106560723	membrane-associated guanylate kinase, WW and PDZ dc	44.35	-1.16	-2.24	0.00	0.00
106608897	LOC106608897	uncharacterized LOC106608897	6.88	-1.16	-2.23	0.00	0.00
106580723	LOC106580723	tristetraprolin-like	330.30	-1.16	-2.23	0.00	0.02
106609164	LOC106609164	small subunit processome component 20 homolog	9.39	-1.15	-2.23	0.00	0.01
106561555	LOC106561555	caveolin-2-like	101.39	-1.15	-2.22	0.00	0.01
100194852	grp2	RAS guanyl-releasing protein 2	35.64	-1.15	-2.22	0.00	0.05
106567355	LOC106567355	PTB domain-containing engulfment adapter protein 1-like	72.06	-1.14	-2.21	0.00	0.01
106601717	LOC106601717	Ig mu chain C region membrane-bound form-like	6.32	-1.14	-2.21	0.00	0.00
106603553	LOC106603553	SAM domain-containing protein SAMSN-1-like	24.48	-1.14	-2.20	0.00	0.02
100196093	chp2	calcineurin-like EF hand protein 2	74.61	-1.13	-2.19	0.00	0.02
106587061	LOC106587061	ankyrin repeat and BTB/POZ domain-containing protein 2	27.71	-1.13	-2.19	0.00	0.00
106562261	LOC106562261	cytochrome P450 2C8-like	17.30	-1.13	-2.18	0.00	0.00
106584302	LOC106584302	lysosome-associated membrane glycoprotein 3-like	24.36	-1.13	-2.18	0.00	0.00
106610162	LOC106610162	ankyrin repeat and SOCS box protein 5-like	6.53	-1.13	-2.18	0.00	0.00
106583965	LOC106583965	uncharacterized LOC106583965	9.66	-1.12	-2.18	0.00	0.00
106602549	LOC106602549	uncharacterized LOC106602549	16.01	-1.12	-2.18	0.00	0.00
106574975	LOC106574975	OX-2 membrane glycoprotein-like	8.47	-1.12	-2.18	0.00	0.01
106574767	LOC106574767	adhesion G-protein coupled receptor G7-like	8.28	-1.12	-2.18	0.00	0.04
106614017	LOC106614017	BCL2/adenovirus E1B 19 kDa protein-interacting protein	85.11	-1.12	-2.17	0.00	0.00
106562030	LOC106562030	uncharacterized LOC106562030	22.03	-1.12	-2.17	0.00	0.00
106578237	LOC106578237	sialic acid-binding Ig-like lectin 16	124.83	-1.12	-2.17	0.00	0.00
106576193	LOC106576193	protein kinase C theta type-like	9.42	-1.12	-2.17	0.00	0.01
106565851	LOC106565851	nicotinamide phosphoribosyltransferase-like	58.19	-1.12	-2.17	0.00	0.00

100195999	cxl10	C-X-C motif chemokine 10	14.41	-1.12	-2.17	0.00	0.01
106607819	LOC106607819	gap junction epsilon-1 protein-like	24.03	-1.12	-2.17	0.00	0.04
106567867	LOC106567867	uncharacterized LOC106567867	12.51	-1.11	-2.16	0.00	0.02
106587891	LOC106587891	poly [ADP-ribose] polymerase 14-like	6.19	-1.11	-2.16	0.00	0.00
100195495	mmp13	Collagenase 3	14.11	-1.10	-2.15	0.00	0.00
106568793	LOC106568793	uncharacterized LOC106568793	7.81	-1.10	-2.14	0.00	0.00
106591866	LOC106591866	guanine nucleotide-binding protein G(o) subunit alpha-lil	11.97	-1.10	-2.14	0.00	0.01
106608841	LOC106608841	probable E3 ubiquitin-protein ligase RNF144A-A	6.88	-1.10	-2.14	0.00	0.00
100380564	ptn7	Tyrosine-protein phosphatase non-receptor type 7	9.90	-1.10	-2.14	0.00	0.00
106581203	LOC106581203	diablo homolog, mitochondrial-like	751.36	-1.10	-2.14	0.00	0.02
106584289	LOC106584289	E3 ubiquitin-protein ligase RNF170-like	80.10	-1.09	-2.14	0.00	0.00
100286697	cxb5	Gap junction beta-5 protein	5.10	-1.09	-2.13	0.00	0.00
106603974	LOC106603974	uncharacterized LOC106603974	495.43	-1.09	-2.13	0.00	0.00
106579830	LOC106579830	triple functional domain protein-like	24.03	-1.09	-2.13	0.00	0.01
106573249	LOC106573249	mitochondrial inner membrane protease subunit 2-like	821.42	-1.09	-2.13	0.00	0.03
106600124	LOC106600124	ephrin type-A receptor 3-like	26.47	-1.09	-2.13	0.00	0.00
106574040	LOC106574040	PDZK1-interacting protein 1-like	97.75	-1.09	-2.13	0.00	0.01
106583905	LOC106583905	protein yippee-like 3	159.85	-1.09	-2.13	0.00	0.02
106608776	LOC106608776	E3 ubiquitin-protein ligase KEG-like	21.19	-1.09	-2.13	0.00	0.00
106609411	LOC106609411	liprin-beta-1-like	460.21	-1.09	-2.12	0.00	0.04
106611011	LOC106611011	uncharacterized LOC106611011	441.57	-1.09	-2.12	0.00	0.02
106612986	LOC106612986	tumor necrosis factor receptor superfamily member 19-li	31.96	-1.09	-2.12	0.00	0.00
106602102	LOC106602102	uncharacterized LOC106602102	15.75	-1.09	-2.12	0.00	0.01
106585060	LOC106585060	B-cell linker protein-like	39.96	-1.09	-2.12	0.00	0.05
100196572	ddn1	Duodenase-1	28.14	-1.09	-2.12	0.00	0.00
106562753	LOC106562753	b(0,+)-type amino acid transporter 1-like	10.26	-1.08	-2.12	0.00	0.01
106577966	LOC106577966	nuclear factor 7, brain-like	9.77	-1.08	-2.12	0.00	0.01
106603693	LOC106603693	protein-tyrosine kinase 2-beta-like	19.09	-1.08	-2.12	0.00	0.04
100196256	stat1	Signal transducer and activator of transcription 1	5.82	-1.08	-2.11	0.00	0.00
106574576	LOC106574576	hemopexin-like	43.42	-1.08	-2.11	0.00	0.01
106606373	LOC106606373	uncharacterized LOC106606373	8.47	-1.08	-2.11	0.00	0.00
106602940	LOC106602940	mitogen-activated protein kinase kinase kinase 2-l	15.62	-1.07	-2.10	0.00	0.00
106612380	LOC106612380	uncharacterized LOC106612380	8.90	-1.07	-2.10	0.00	0.00
106583449	LOC106583449	voltage-dependent L-type calcium channel subunit alpha	69.58	-1.07	-2.10	0.00	0.02
106561493	LOC106561493	neuron navigator 2-like	142.18	-1.07	-2.10	0.00	0.00
106561918	LOC106561918	neuron navigator 2-like	97.74	-1.07	-2.10	0.00	0.00
106613835	LOC106613835	leupaxin-like	26.05	-1.07	-2.09	0.00	0.00
106582237	LOC106582237	interferon-induced guanylate-binding protein 1-like	53.53	-1.06	-2.09	0.00	0.03
100270810	ncf1	neutrophil cytosolic factor 1	12.21	-1.06	-2.09	0.00	0.00
106607842	LOC106607842	collagen alpha-1(XVI) chain-like	15.25	-1.06	-2.09	0.00	0.00
106567852	LOC106567852	calphotin-like	6.23	-1.06	-2.09	0.00	0.02
106611800	LOC106611800	uncharacterized LOC106611800	57.38	-1.06	-2.08	0.00	0.00
106572043	LOC106572043	E3 ubiquitin-protein ligase RNF213-like	34.15	-1.06	-2.08	0.00	0.03
106608837	LOC106608837	uncharacterized protein DDB_G0290685-like	120.75	-1.06	-2.08	0.00	0.02
106564565	LOC106564565	probable ATP-dependent RNA helicase DDX17	112.08	-1.06	-2.08	0.00	0.00
106613068	LOC106613068	uncharacterized LOC106613068	7.06	-1.05	-2.08	0.00	0.00
106564314	LOC106564314	transmembrane channel-like protein 8	68.81	-1.05	-2.07	0.00	0.01
106576332	LOC106576332	myosin-binding protein C, slow-type-like	16.50	-1.05	-2.07	0.00	0.00
106589545	LOC106589545	transcription factor MafF-like	113.85	-1.05	-2.07	0.00	0.03
106613637	LOC106613637	interleukin-6 receptor subunit beta-like	6.53	-1.05	-2.07	0.00	0.01
106571278	LOC106571278	uncharacterized LOC106571278	42.35	-1.05	-2.07	0.00	0.01

106568343	LOC106568343	calcium/calmodulin-dependent protein kinase type IV-lik	5.52	-1.05	-2.07	0.00	0.01
106608989	LOC106608989	SLAM family member 9-like	14.66	-1.05	-2.07	0.00	0.00
106578584	LOC106578584	protein phosphatase 1 regulatory subunit 3G-like	88.80	-1.05	-2.07	0.00	0.02
106561874	LOC106561874	uncharacterized LOC106561874	11.63	-1.04	-2.06	0.00	0.00
106587141	LOC106587141	adhesion G-protein coupled receptor G5-like	14.72	-1.04	-2.06	0.00	0.00
106603652	micu2	mitochondrial calcium uptake 2	74.18	-1.04	-2.05	0.00	0.02
106583450	LOC106583450	uncharacterized LOC106583450	136.73	-1.04	-2.05	0.00	0.01
106603097	LOC106603097	voltage-gated potassium channel subunit beta-3-like	63.51	-1.04	-2.05	0.00	0.00
106566613	LOC106566613	uncharacterized LOC106566613	18.64	-1.04	-2.05	0.00	0.03
106584293	ptpru	NA	15.13	-1.04	-2.05	0.00	0.00
106579832	LOC106579832	AT-rich interactive domain-containing protein 5A-like	10.47	-1.04	-2.05	0.00	0.00
106569420	LOC106569420	rho GTPase-activating protein 30-like	21.20	-1.04	-2.05	0.00	0.00
106564646	LOC106564646	rab11 family-interacting protein 4A-like	17.94	-1.04	-2.05	0.00	0.05
106568752	LOC106568752	eukaryotic initiation factor 4A-II	155.29	-1.03	-2.05	0.00	0.00
106579597	LOC106579597	5'-AMP-activated protein kinase subunit gamma-2-like	62.09	-1.03	-2.05	0.00	0.01
106562346	LOC106562346	la-related protein 6-like	8.02	-1.03	-2.05	0.00	0.00
106568165	LOC106568165	interleukin-7 receptor subunit alpha-like	17.21	-1.03	-2.05	0.00	0.04
106584392	LOC106584392	nuclear receptor ROR-beta-like	193.47	-1.03	-2.05	0.00	0.00
106610842	LOC106610842	eukaryotic translation initiation factor 2-alpha kinase 4-li	65.91	-1.03	-2.04	0.00	0.00
106568487	sema4d	NA	19.78	-1.03	-2.04	0.00	0.00
106585459	LOC106585459	stromelysin-3-like	321.86	-1.03	-2.04	0.00	0.02
106587528	LOC106587528	transcription factor SOX-6-like	5.45	-1.03	-2.04	0.00	0.00
106563276	LOC106563276	HAUS augmin-like complex subunit 4	53.39	-1.03	-2.04	0.00	0.01
106563312	LOC106563312	atrial natriuretic peptide receptor 2-like	22.65	-1.03	-2.04	0.00	0.00
106596550	LOC106596550	ankyrin repeat and SOCS box protein 2-like	7.28	-1.03	-2.04	0.00	0.01
106589322	LOC106589322	delta-1-pyrroline-5-carboxylate synthase-like	12.81	-1.03	-2.04	0.00	0.00
106605276	LOC106605276	neurogenic locus notch homolog protein 1-like	10.99	-1.03	-2.04	0.00	0.00
106584294	LOC106584294	uncharacterized LOC106584294	135.98	-1.03	-2.04	0.00	0.02
100136479	LOC100136479	CD83 antigen	11.80	-1.03	-2.04	0.00	0.00
106609582	LOC106609582	hepatocyte growth factor-like	17.60	-1.02	-2.03	0.00	0.00
106583087	LOC106583087	complement C3-like	573.53	-1.02	-2.03	0.00	0.01
106584635	LOC106584635	UPF0676 protein C1494.01-like	29.81	-1.02	-2.03	0.00	0.00
106582258	LOC106582258	interleukin-1 receptor type 1-like	11.10	-1.02	-2.03	0.00	0.00
106560847	LOC106560847	UDP-GlcNAc:betaGal beta-1,3-N-acetylglucosaminyltrans	6.68	-1.02	-2.03	0.00	0.03
106573179	LOC106573179	ras and EF-hand domain-containing protein homolog	17.67	-1.02	-2.02	0.00	0.01
106583740	LOC106583740	stonustoxin subunit beta-like	56.65	-1.02	-2.02	0.00	0.01
106562798	fsd2	fibronectin type III and SPRY domain containing 2	7.98	-1.02	-2.02	0.00	0.00
106604524	tdo2	NA	6226.70	-1.01	-2.02	0.00	0.00
106569300	LOC106569300	uncharacterized LOC106569300	111.75	-1.01	-2.02	0.00	0.01
106594372	LOC106594372	zinc finger protein 568-like	19.43	-1.01	-2.02	0.00	0.01
106567073	LOC106567073	grainyhead-like protein 1 homolog	11.82	-1.01	-2.02	0.00	0.02
106564788	srsf7	NA	1215.20	-1.01	-2.02	0.00	0.00
100286470	ecop	EGFR-coamplified and overexpressed protein	33.53	-1.01	-2.01	0.00	0.02
106600494	LOC106600494	methionine aminopeptidase 1	463.08	-1.01	-2.01	0.00	0.00
106605167	LOC106605167	pleckstrin homology-like domain family B member 3	55.91	-1.01	-2.01	0.00	0.01
106566437	LOC106566437	cytochrome c oxidase subunit 4 isoform 2, mitochondrial	840.53	-1.01	-2.01	0.00	0.01
106599714	LOC106599714	solute carrier family 22 member 23-like	132.13	-1.01	-2.01	0.00	0.01
106599149	LOC106599149	piggyBac transposable element-derived protein 4-like	13.80	-1.01	-2.01	0.00	0.00
106575296	LOC106575296	dipeptidyl peptidase 4-like	235.28	-1.01	-2.01	0.00	0.02
106590112	LOC106590112	homeobox protein TGIF1-like	36.84	-1.01	-2.01	0.00	0.00
106582117	lrrc58	leucine rich repeat containing 58	132.04	-1.00	-2.00	0.00	0.01

106575562	LOC106575562	T-cell surface antigen CD2-like	8.56	-1.00	-2.00	0.00	0.00

Supplementary Table S2b

Ploidy group: Triploid

Up-regulated DEGs in fry compared to parr

NCBI ID	Gene IDs/Locus	Gene name	baseMean	log2FoldChange	Fold change	pvalue	padj
106600509	LOC106600509	alcohol dehydrogenase 1-like	1975.00	5.47	44.21	0.00	0.00
106610407	LOC106610407	up-regulator of cell proliferation-like	12.86	3.63	12.36	0.00	0.00
106584294	LOC106584294	uncharacterized LOC106584294	72.04	3.50	11.34	0.00	0.00
106568683	LOC106568683	inhibin beta A chain-like	31.04	3.44	10.86	0.01	0.04
106589887	LOC106589887	fatty acid desaturase 6-like	16.90	3.36	10.25	0.00	0.00
106582432	LOC106582432	trace amine-associated receptor 13c-like	14.35	3.11	8.61	0.00	0.00
106585649	LOC106585649	complement C1q-like protein 2	267.74	3.02	8.10	0.00	0.01
106605225	LOC106605225	cytochrome c oxidase subunit 7A-related protei	102.05	2.94	7.65	0.00	0.00
106613140	LOC106613140	sodium- and chloride-dependent GABA transpo	5.56	2.87	7.31	0.00	0.01
106565178	LOC106565178	interferon-induced protein 44-like	84.16	2.73	6.62	0.00	0.00
106568567	LOC106568567	UDP-glucuronosyltransferase 2A1-like	54.77	2.68	6.41	0.00	0.00
106610457	LOC106610457	interferon-induced very large GTPase 1-like	22.70	2.64	6.24	0.00	0.00
100169854	LOC100169854	uncharacterized LOC100169854	11.33	2.60	6.07	0.00	0.00
106573520	LOC106573520	zona pellucida sperm-binding protein 4-like	1438.50	2.58	5.97	0.00	0.00
106608992	LOC106608992	purine nucleoside phosphorylase-like	651.24	2.55	5.88	0.00	0.01
100136450	LOC100136450	CD8 alpha	6.95	2.54	5.80	0.00	0.03
106571128	LOC106571128	uncharacterized LOC106571128	1417.35	2.48	5.60	0.00	0.00
106570607	LOC106570607	E3 ubiquitin-protein ligase ZNRF2-like	12.85	2.46	5.51	0.00	0.02
106564531	LOC106564531	GATA zinc finger domain-containing protein 10-	63.78	2.46	5.49	0.00	0.01
106574341	LOC106574341	insulin receptor substrate 2-like	58.66	2.41	5.32	0.00	0.00
106604696	LOC106604696	saxitoxin and tetrodotoxin-binding protein 2-lik	88.44	2.40	5.27	0.00	0.00
106565117	LOC106565117	differentially expressed in FDCP 6 homolog	18.56	2.39	5.24	0.00	0.01
100196604	apof	apolipoprotein F	684.95	2.37	5.19	0.00	0.00
106577515	LOC106577515	uncharacterized LOC106577515	10.63	2.35	5.09	0.00	0.01
106562007	LOC106562007	BCL2/adenovirus E1B 19 kDa protein-interacting	352.37	2.35	5.09	0.00	0.02
106573415	LOC106573415	carnitine O-palmitoyltransferase 1, liver isoform	134.48	2.33	5.05	0.00	0.00
100195250	typh	Thymidine phosphorylase	87.78	2.33	5.01	0.00	0.00
106609502	LOC106609502	tubby-related protein 3-like	26.47	2.27	4.82	0.00	0.03
106599151	LOC106599151	acyl-coenzyme A thioesterase 11-like	30.14	2.26	4.78	0.00	0.01
106612462	LOC106612462	uncharacterized LOC106612462	27.01	2.26	4.78	0.00	0.01
106565647	LOC106565647	von Willebrand factor A domain-containing pro	3.87	2.24	4.71	0.00	0.00
106567252	LOC106567252	collagen alpha-1(I) chain-like	12.25	2.23	4.70	0.00	0.00
106609060	LOC106609060	rho GTPase-activating protein 6-like	16.21	2.23	4.68	0.00	0.01
106571115	LOC106571115	adenylate kinase 7-like	4.17	2.22	4.67	0.00	0.00
106608238	LOC106608238	cholesterol 24-hydroxylase-like	20.75	2.22	4.67	0.00	0.00
106579828	LOC106579828	uncharacterized LOC106579828	24.45	2.21	4.61	0.00	0.00
106608989	LOC106608989	SLAM family member 9-like	18.75	2.20	4.60	0.00	0.00

106566106	LOC106566106	mitogen-activated protein kinase 14A-like	7.85	2.19	4.58	0.00	0.00
106568688	LOC106568688	uncharacterized LOC106568688	14.46	2.19	4.56	0.00	0.00
106585878	LOC106585878	C-C motif chemokine 19-like	38.93	2.19	4.55	0.00	0.00
106569872	LOC106569872	semaphorin-4A-like	38.57	2.18	4.52	0.00	0.00
100136451	LOC100136451	CD8 beta	5.26	2.18	4.52	0.00	0.02
106613803	LOC106613803	C2 calcium-dependent domain-containing prote	63.41	2.17	4.50	0.00	0.00
106581655	LOC106581655	P2Y purinoceptor 8-like	6.28	2.15	4.44	0.00	0.00
106577998	LOC106577998	protein NDRG2-like	828.09	2.15	4.44	0.00	0.00
106600841	LOC106600841	nuclear factor erythroid 2-related factor 1-like	55.88	2.14	4.39	0.00	0.01
106607758	lama2	laminin subunit alpha 2	44.53	2.14	4.39	0.00	0.01
106582601	LOC106582601	pleckstrin homology-like domain family B meml	17.54	2.14	4.39	0.00	0.00
106604583	LOC106604583	leukocyte cell-derived chemotaxin-2-like	778.88	2.13	4.39	0.00	0.00
106602644	LOC106602644	uncharacterized LOC106602644	15.01	2.11	4.33	0.00	0.02
106568147	LOC106568147	dual specificity protein phosphatase 4-like	5.51	2.10	4.30	0.00	0.00
106613695	LOC106613695	lipoprotein lipase-like	646.68	2.10	4.30	0.00	0.00
106605425	LOC106605425	metalloreductase STEAP4-like	26.35	2.09	4.26	0.00	0.00
106583167	LOC106583167	class E basic helix-loop-helix protein 40-like	121.07	2.08	4.22	0.00	0.00
106562616	LOC106562616	probable DNA polymerase	8.03	2.06	4.17	0.00	0.00
106605673	LOC106605673	B-cell antigen receptor complex-associated prot	33.12	2.06	4.16	0.00	0.03
106600948	LOC106600948	synaptotagmin-2-like	9.92	2.05	4.15	0.00	0.00
106606790	LOC106606790	Ig mu chain C region membrane-bound form-lik	42.47	2.04	4.12	0.00	0.00
106560599	LOC106560599	ectoderm-neural cortex protein 1-like	21.20	2.00	4.01	0.00	0.00
106589414	LOC106589414	inactive ubiquitin carboxyl-terminal hydrolase 5	11.37	1.99	3.96	0.00	0.00
100136518	igfbp-1b1	IGF binding protein 1	101.23	1.98	3.94	0.00	0.00
106583020	LOC106583020	receptor-type tyrosine-protein phosphatase gar	71.42	1.98	3.93	0.00	0.00
106607477	LOC106607477	pyruvate dehydrogenase (acetyl-transferring) ki	128.64	1.97	3.92	0.00	0.00
106588240	LOC106588240	free fatty acid receptor 2-like	5.67	1.97	3.92	0.00	0.00
106561069	LOC106561069	protein FAM60A-like	9.63	1.96	3.89	0.00	0.01
106578023	LOC106578023	uncharacterized LOC106578023	8.51	1.96	3.88	0.00	0.00
106569984	LOC106569984	C-type lectin domain family 5 member A-like	75.34	1.95	3.87	0.00	0.00
106603875	LOC106603875	CREB3 regulatory factor-like	112.74	1.95	3.87	0.00	0.00
100136365	LOC100136365	lysozyme C II	181.39	1.93	3.81	0.00	0.00
106566501	LOC106566501	uncharacterized LOC106566501	25.45	1.93	3.81	0.00	0.01
100195050	plbl1	phospholipase B-like 1	103.90	1.93	3.80	0.00	0.00
106609985	LOC106609985	uncharacterized LOC106609985	44.67	1.92	3.79	0.00	0.00
106565246	LOC106565246	coiled-coil domain-containing protein 37-like	7.72	1.91	3.77	0.00	0.00
106565782	LOC106565782	granzyme K-like	12.12	1.91	3.76	0.00	0.01
106565146	LOC106565146	uncharacterized LOC106565146	16.39	1.90	3.72	0.00	0.01
106564975	LOC106564975	integrin alpha-X-like	32.33	1.89	3.71	0.00	0.00
106600856	LOC106600856	deleted in malignant brain tumors 1 protein-like	4.52	1.89	3.70	0.00	0.01

106611011	LOC106611011	uncharacterized LOC106611011	316.56	1.89	3.70	0.00	0.01
106576186	LOC106576186	hemagglutinin/amebocyte aggregation factor-li	95.59	1.89	3.69	0.00	0.00
106585857	LOC106585857	arrestin domain-containing protein 3-like	432.70	1.88	3.69	0.00	0.00
106566489	LOC106566489	scavenger receptor cysteine-rich type 1 protein	9.10	1.87	3.66	0.00	0.03
106577374	LOC106577374	BCL2/adenovirus E1B 19 kDa protein-interacting	59.43	1.87	3.66	0.01	0.05
106605167	LOC106605167	pleckstrin homology-like domain family B meml	41.88	1.87	3.65	0.00	0.00
106610794	LOC106610794	serine/threonine-protein kinase D3-like	18.22	1.87	3.64	0.00	0.00
106577640	LOC106577640	uncharacterized LOC106577640	57.35	1.86	3.63	0.00	0.00
106563192	LOC106563192	proactivator polypeptide-like	152.70	1.86	3.62	0.00	0.01
106613930	LOC106613930	calcium-activated potassium channel subunit be	132.95	1.84	3.59	0.00	0.00
106614017	LOC106614017	BCL2/adenovirus E1B 19 kDa protein-interacting	64.42	1.84	3.58	0.00	0.00
106565094	LOC106565094	contactin-2-like	7.03	1.84	3.58	0.00	0.00
106584062	LOC106584062	cytochrome P450 2J2-like	534.14	1.84	3.57	0.00	0.02
100380321	pisd	phosphatidylserine decarboxylase	881.57	1.83	3.57	0.00	0.01
106575805	LOC106575805	ETS translocation variant 5-like	4.42	1.83	3.57	0.00	0.00
106582482	LOC106582482	trace amine-associated receptor 13c-like	74.98	1.83	3.56	0.00	0.00
106584587	LOC106584587	hepcidin-1	729.01	1.83	3.56	0.00	0.00
106613130	LOC106613130	solute carrier family 25 member 36-A-like	106.92	1.83	3.56	0.00	0.00
106560615	cldn18	claudin 18	5.60	1.83	3.55	0.00	0.00
100195257	ugpa	UTP--glucose-1-phosphate uridylyltransferase	199.86	1.83	3.55	0.00	0.00
106568062	LOC106568062	arsenite methyltransferase-like	58.69	1.83	3.55	0.00	0.01
106582019	LOC106582019	guanine nucleotide exchange factor DBS-like	46.60	1.83	3.55	0.00	0.00
106583905	LOC106583905	protein yippee-like 3	104.19	1.83	3.54	0.00	0.00
106578237	LOC106578237	sialic acid-binding Ig-like lectin 16	126.34	1.82	3.54	0.00	0.01
106581859	LOC106581859	integrin beta-2-like	81.32	1.82	3.53	0.00	0.01
106582117	lrrc58	leucine rich repeat containing 58	83.69	1.81	3.52	0.00	0.01
106562531	LOC106562531	E3 ubiquitin-protein ligase TRIM21-like	4.03	1.81	3.52	0.00	0.00
106575435	LOC106575435	Ig kappa chain V-V region MOPC 21-like	12.35	1.81	3.51	0.00	0.00
106578152	LOC106578152	dachshund homolog 2-like	4.41	1.81	3.50	0.00	0.00
106560477	LOC106560477	phosphoglucomutase-1-like	169.91	1.80	3.49	0.00	0.00
106570315	LOC106570315	adseverin-like	9.33	1.80	3.48	0.00	0.04
106610718	LOC106610718	zinc finger protein 395-like	18.18	1.80	3.48	0.00	0.01
106589283	LOC106589283	src kinase-associated phosphoprotein 1-like	4.54	1.80	3.47	0.00	0.00
106585105	LOC106585105	max dimerization protein 1-like	84.47	1.80	3.47	0.00	0.00
106584291	LOC106584291	cytochrome P450 2J2-like	3584.94	1.79	3.47	0.00	0.03
106611397	LOC106611397	uncharacterized LOC106611397	11.01	1.79	3.46	0.00	0.00
106610167	LOC106610167	15-hydroxyprostaglandin dehydrogenase [NAD(	367.77	1.78	3.43	0.00	0.00
106606554	LOC106606554	poly [ADP-ribose] polymerase 14-like	16.17	1.77	3.42	0.00	0.00
100195746	cd37	Leukocyte antigen CD37	23.71	1.77	3.41	0.00	0.02
106582421	LOC106582421	sodium channel subunit beta-1-like	5.26	1.76	3.39	0.00	0.03

106607817	heca	hdc homolog, cell cycle regulator	10.69	1.76	3.39	0.00	0.01
100196218	glrx1	Glutaredoxin-1	50.19	1.76	3.38	0.00	0.00
106563255	LOC106563255	endothelial differentiation-related factor 1 hom	61.20	1.76	3.38	0.00	0.00
106581262	LOC106581262	uncharacterized LOC106581262	24.09	1.76	3.38	0.00	0.02
106612585	LOC106612585	uncharacterized LOC106612585	12.03	1.75	3.37	0.00	0.03
106567876	LOC106567876	acetylcholine receptor subunit gamma-like	6.54	1.75	3.36	0.00	0.02
106601108	LOC106601108	uncharacterized LOC106601108	39.08	1.74	3.35	0.00	0.00
106612143	LOC106612143	syntaxin-binding protein 4-like	19.40	1.74	3.35	0.00	0.00
106590089	LOC106590089	protein phosphatase 1 regulatory subunit 3G-lik	241.43	1.74	3.35	0.00	0.01
100194621	adssl2	adenylosuccinate synthase like 2	21.99	1.74	3.34	0.00	0.00
106561509	LOC106561509	E3 ubiquitin/ISG15 ligase TRIM25-like	60.77	1.73	3.33	0.00	0.00
106577198	LOC106577198	catechol O-methyltransferase domain-containir	75.10	1.73	3.32	0.00	0.01
100194912	angl4	Angiopoietin-related protein 4	225.92	1.73	3.32	0.00	0.00
106585087	LOC106585087	ankyrin repeat and LEM domain-containing pro	60.49	1.73	3.32	0.00	0.00
106569199	LOC106569199	glutamine synthetase-like	4564.37	1.73	3.31	0.00	0.00
106566619	LOC106566619	interleukin-17 receptor E-like	13.42	1.72	3.30	0.00	0.00
106594449	LOC106594449	bifunctional apoptosis regulator-like	32.94	1.72	3.30	0.00	0.00
106576339	LOC106576339	Ig mu chain C region-like	7.04	1.72	3.30	0.00	0.01
106611104	LOC106611104	intron-binding protein aquarius-like	33.17	1.71	3.27	0.00	0.00
106587550	LOC106587550	switch-associated protein 70-like	22.46	1.71	3.27	0.00	0.00
106572192	LOC106572192	intelectin	88.65	1.71	3.26	0.00	0.00
106567901	LOC106567901	solute carrier family 13 member 5-like	24.42	1.70	3.25	0.00	0.00
106576903	LOC106576903	Ig gamma-2 chain C region-like	4.56	1.70	3.25	0.00	0.00
106607353	LOC106607353	3-phosphoinositide-dependent protein kinase 1	14.00	1.69	3.24	0.00	0.00
106563276	LOC106563276	HAUS augmin-like complex subunit 4	36.61	1.69	3.23	0.00	0.00
106610796	LOC106610796	adenylyltransferase and sulfurtransferase MOC	4.18	1.69	3.23	0.00	0.00
106560822	LOC106560822	BCL2/adenovirus E1B 19 kDa protein-interacting	11.99	1.68	3.21	0.00	0.00
106587033	LOC106587033	uncharacterized LOC106587033	22.58	1.68	3.20	0.00	0.00
106571710	LOC106571710	transcription factor IIIB 90 kDa subunit-like	69.83	1.68	3.20	0.00	0.00
106573414	LOC106573414	kelch repeat and BTB domain-containing protei	8.81	1.67	3.19	0.00	0.00
100195489	ccr9	C-C chemokine receptor type 9	10.35	1.67	3.18	0.00	0.01
106601045	LOC106601045	histone-lysine N-methyltransferase EZH1-like	26.22	1.66	3.17	0.00	0.00
106567020	LOC106567020	serine/threonine-protein kinase WNK2-like	100.47	1.66	3.17	0.00	0.00
100306791	i12r2	Interleukin-12 receptor beta-2 chain	14.62	1.66	3.16	0.00	0.01
106603974	LOC106603974	uncharacterized LOC106603974	275.94	1.66	3.16	0.00	0.00
100136527	slc6a6	solute carrier family 6 (neurotransmitter transp	36.65	1.66	3.15	0.00	0.01
106571232	LOC106571232	TATA box-binding protein-associated factor RN/	14.35	1.65	3.14	0.00	0.00
106569337	LOC106569337	synaptosomal-associated protein 47-like	9.62	1.65	3.14	0.00	0.00
106576229	LOC106576229	ankyrin repeat and SOCS box protein 13-like	9.65	1.65	3.14	0.00	0.01
106588235	LOC106588235	GTP-binding protein Rhes pseudogene	14.83	1.64	3.12	0.00	0.01

106585165	LOC106585165	glycerol-3-phosphate acyltransferase 3-like	8.82	1.64	3.12	0.00	0.00
106575701	LOC106575701	uncharacterized LOC106575701	26.97	1.64	3.11	0.00	0.00
106587975	LOC106587975	C-C motif chemokine 13-like	5.13	1.63	3.09	0.00	0.00
106577791	LOC106577791	matrix metalloproteinase-14-like	230.84	1.63	3.09	0.00	0.00
106573352	LOC106573352	inactive serine protease PAMR1-like	52.15	1.62	3.08	0.01	0.05
106565147	LOC106565147	uncharacterized LOC106565147	3.91	1.62	3.07	0.00	0.01
100526661	foxp3	forkhead box P3	4.49	1.62	3.07	0.00	0.00
106565611	LOC106565611	probable G-protein coupled receptor	6.54	1.62	3.07	0.00	0.00
106569300	LOC106569300	uncharacterized LOC106569300	88.62	1.61	3.06	0.00	0.02
106601234	LOC106601234	uncharacterized LOC106601234	7.11	1.61	3.06	0.00	0.01
106565699	LOC106565699	H-2 class II histocompatibility antigen, A-U alpha	705.52	1.61	3.05	0.00	0.00
106609552	LOC106609552	Ig kappa-b4 chain C region-like	6.69	1.61	3.05	0.00	0.00
100380613	stea4	Metalloreductase STEAP4	634.52	1.61	3.05	0.00	0.00
100136511	LOC100136511	CCL4-like chemokine	8.97	1.61	3.05	0.00	0.00
106606997	LOC106606997	SEC14-like protein 1	132.24	1.60	3.04	0.00	0.00
106589454	LOC106589454	probable ATP-dependent RNA helicase DDX17	33.65	1.60	3.04	0.00	0.02
106588908	LOC106588908	zinc finger FYVE domain-containing protein 1-lik	64.02	1.60	3.04	0.00	0.00
106562071	LOC106562071	uncharacterized LOC106562071	23.53	1.60	3.02	0.00	0.00
106568131	lhfpl2	lipoma HMGIC fusion partner-like 2	31.93	1.59	3.02	0.00	0.00
106565959	LOC106565959	taste receptor type 1 member 3-like	8.64	1.59	3.02	0.00	0.00
106566034	LOC106566034	uncharacterized LOC106566034	31.43	1.59	3.00	0.00	0.00
100195997	mgp	matrix Gla protein	42.30	1.58	3.00	0.00	0.01
106561729	LOC106561729	tumor protein p53-inducible protein 11-like	85.53	1.58	2.98	0.00	0.00
106613137	LOC106613137	sphingosine-1-phosphate phosphatase 2-like	21.67	1.57	2.97	0.00	0.00
106585608	LOC106585608	vinexin-like	19.85	1.57	2.97	0.00	0.00
106582291	LOC106582291	sclerostin domain-containing protein 1-like	8.24	1.57	2.96	0.00	0.00
106583939	LOC106583939	nuclear protein 1-like	777.54	1.57	2.96	0.00	0.01
106576235	LOC106576235	ADM2-like	39.41	1.56	2.96	0.00	0.00
106569780	LOC106569780	T-cell differentiation antigen CD6-like	27.35	1.56	2.96	0.00	0.00
106584838	neil1	nei like DNA glycosylase 1	63.28	1.56	2.95	0.00	0.02
106583433	LOC106583433	interferon-induced protein 44-like	15.16	1.56	2.95	0.00	0.03
106607198	LOC106607198	UPF0515 protein C19orf66 homolog	33.25	1.56	2.94	0.00	0.01
106586030	LOC106586030	guanine nucleotide-binding protein G(I)/G(S)/G	6.13	1.55	2.94	0.00	0.00
100195724	sh21a	SH2 domain-containing protein 1A	14.91	1.55	2.93	0.00	0.02
106603300	LOC106603300	E3 ubiquitin-protein ligase RNF128-like	5.92	1.55	2.93	0.00	0.00
106589384	LOC106589384	pantothenate kinase 1-like	77.08	1.55	2.93	0.00	0.00
106561703	hbp1	HMG-box transcription factor 1	108.07	1.55	2.92	0.00	0.00
106590371	LOC106590371	sodium channel protein type 4 subunit alpha-lik	3.92	1.55	2.92	0.00	0.00
106609356	LOC106609356	endoplasmin-like	392.27	1.54	2.92	0.00	0.02
106604416	LOC106604416	HRAS-like suppressor 3	18.85	1.54	2.90	0.00	0.01

106589061	LOC106589061	transcription factor SOX-4-like	84.74	1.53	2.89	0.00	0.01
106568725	LOC106568725	UDP-N-acetylglucosamine/UDP-glucose/GDP-m	114.70	1.53	2.89	0.00	0.00
106612540	LOC106612540	alpha-2-macroglobulin-like	793.05	1.53	2.89	0.00	0.00
106568124	LOC106568124	complement C1q-like protein 2	8849.79	1.53	2.89	0.00	0.01
106602264	LOC106602264	beclin 1-associated autophagy-related key regu	26.57	1.53	2.88	0.00	0.00
106567883	LOC106567883	uncharacterized LOC106567883	19.25	1.53	2.88	0.00	0.00
106612779	ift80	intraflagellar transport 80	3.87	1.52	2.88	0.00	0.03
106575303	LOC106575303	xin actin-binding repeat-containing protein 2-lik	259.25	1.52	2.87	0.00	0.00
106584163	LOC106584163	uncharacterized protein KIAA1107-like	33.63	1.52	2.87	0.00	0.00
106611874	LOC106611874	macrophage colony-stimulating factor 1 recepto	103.43	1.52	2.86	0.00	0.00
106609923	LOC106609923	fibrinogen gamma chain-like	36.30	1.52	2.86	0.00	0.02
106575700	LOC106575700	uncharacterized LOC106575700	25.21	1.52	2.86	0.00	0.00
106572146	LOC106572146	perlwapin-like	54.75	1.51	2.86	0.01	0.04
106611185	LOC106611185	uncharacterized LOC106611185	5.23	1.51	2.85	0.00	0.01
106586555	LOC106586555	uncharacterized LOC106586555	8.32	1.51	2.85	0.00	0.00
106573758	LOC106573758	choline kinase alpha-like	284.88	1.51	2.85	0.00	0.00
106589372	lgi1	leucine rich glioma inactivated 1	24.73	1.51	2.85	0.01	0.05
106601031	LOC106601031	coenzyme Q-binding protein COQ10 homolog, r	203.40	1.51	2.84	0.00	0.01
106613733	LOC106613733	mitochondrial coenzyme A transporter SLC25A4	13.02	1.50	2.84	0.00	0.00
106603069	LOC106603069	lipase member H-like	130.44	1.50	2.83	0.00	0.01
106568356	LOC106568356	fructose-1,6-bisphosphatase 1-like	1153.91	1.50	2.83	0.00	0.01
106600906	LOC106600906	peroxisomal membrane protein PMP34-like	18.77	1.50	2.83	0.00	0.00
100196757	c1qc	complement component 1, q subcomponent, C	136.79	1.50	2.83	0.00	0.00
106608772	LOC106608772	probable G-protein coupled receptor 148	5.69	1.50	2.82	0.00	0.00
106578437	LOC106578437	semaphorin-5A-like	11.45	1.50	2.82	0.00	0.00
106580086	LOC106580086	guanine nucleotide-binding protein G(q) subuni	18.05	1.49	2.82	0.00	0.00
106602030	LOC106602030	toll-like receptor 13	4.16	1.49	2.81	0.00	0.00
106609586	LOC106609586	T-complex protein 11-like protein 2	67.40	1.49	2.81	0.00	0.00
106610720	grhl1	grainyhead like transcription factor 1	5.13	1.49	2.81	0.00	0.00
106571437	LOC106571437	uncharacterized LOC106571437	8.16	1.49	2.81	0.00	0.02
106612986	LOC106612986	tumor necrosis factor receptor superfamily mer	31.93	1.49	2.80	0.00	0.02
106611907	LOC106611907	G-protein coupled receptor family C group 5 me	111.17	1.48	2.80	0.00	0.02
106582621	LOC106582621	tumor necrosis factor receptor superfamily mer	5.40	1.48	2.80	0.00	0.00
106579791	cldn1	claudin 1	147.80	1.48	2.79	0.00	0.00
106609917	LOC106609917	uncharacterized LOC106609917	4.56	1.48	2.79	0.00	0.02
106601033	LOC106601033	complement C1q-like protein 2	496.23	1.48	2.78	0.00	0.00
106571436	LOC106571436	frizzled-3-like	4.74	1.48	2.78	0.00	0.00
106604522	tenm3	NA	108.52	1.47	2.78	0.00	0.00
106611589	LOC106611589	leukocyte cell-derived chemotaxin-2-like	50.32	1.47	2.78	0.00	0.00
100194666	LOC100194666	BCL2/adenovirus E1B 19 kDa protein-interacting	694.83	1.47	2.77	0.00	0.02

106563597	LOC106563597	uncharacterized LOC106563597	328.66	1.47	2.77	0.01	0.05
106573648	LOC106573648	olfactomedin-4-like	61.34	1.47	2.77	0.00	0.00
106571979	LOC106571979	uncharacterized LOC106571979	9.05	1.47	2.76	0.00	0.01
106580528	LOC106580528	leukocyte surface antigen CD53-like	10.07	1.46	2.76	0.00	0.00
106582135	LOC106582135	ras-related protein Rab-20-like	192.82	1.46	2.75	0.00	0.00
106572285	LOC106572285	tyrosine-protein phosphatase non-receptor typ	18.61	1.46	2.75	0.00	0.00
106589615	LOC106589615	filaggrin-2-like	25.57	1.46	2.75	0.00	0.00
106564353	LOC106564353	antimicrobial peptide NK-lysin-like	4.80	1.46	2.75	0.00	0.00
106562499	LOC106562499	spermatogenesis-associated protein 5-like prot	13.06	1.45	2.74	0.00	0.00
100136478	LOC100136478	alpha amylase	2112.04	1.45	2.74	0.00	0.00
106583133	LOC106583133	period circadian protein homolog 3-like	36.18	1.45	2.74	0.00	0.02
106586898	LOC106586898	uncharacterized LOC106586898	6.23	1.45	2.73	0.01	0.05
106562907	LOC106562907	aftiphilin-like	11.05	1.45	2.73	0.00	0.00
106604524	tdo2	NA	4158.77	1.45	2.73	0.00	0.00
106606762	LOC106606762	Ig mu chain C region membrane-bound form-lik	9.55	1.45	2.73	0.00	0.01
106603573	slc13a5	NA	144.57	1.44	2.72	0.00	0.00
106562736	LOC106562736	b(0,+)-type amino acid transporter 1-like	7.07	1.44	2.72	0.00	0.00
106598999	LOC106598999	uncharacterized LOC106598999	21.11	1.44	2.72	0.00	0.00
106578866	LOC106578866	forkhead box protein K2-like	191.28	1.44	2.72	0.00	0.00
106565108	LOC106565108	bombesin receptor-activated protein C6orf89 h	428.49	1.44	2.71	0.00	0.00
106567759	LOC106567759	uncharacterized LOC106567759	29.26	1.44	2.71	0.00	0.00
100306785	itb2	Integrin beta-2	138.81	1.44	2.71	0.00	0.02
106568165	LOC106568165	interleukin-7 receptor subunit alpha-like	14.13	1.44	2.71	0.00	0.00
106566064	LOC106566064	guanine nucleotide-binding protein G(i) subunit	6.46	1.43	2.70	0.00	0.00
100380715	relb	NA	132.75	1.43	2.70	0.00	0.00
106560917	LOC106560917	olfactomedin-4-like	46.84	1.43	2.70	0.00	0.00
106579999	LOC106579999	folylpolyglutamate synthase, mitochondrial-like	243.81	1.43	2.70	0.00	0.01
100195217	ppm1h	protein phosphatase, Mg2+/Mn2+ dependent,	8.74	1.43	2.70	0.00	0.01
106566105	LOC106566105	mitogen-activated protein kinase 14A-like	89.74	1.43	2.70	0.00	0.03
106564356	LOC106564356	rano class II histocompatibility antigen, A beta c	9.46	1.43	2.70	0.00	0.00
106584369	LOC106584369	tyrosine-protein kinase ZAP-70-like	22.74	1.43	2.69	0.00	0.03
106605529	LOC106605529	Krueppel-like factor 10	42.48	1.43	2.69	0.00	0.00
106565537	LOC106565537	receptor tyrosine-protein kinase erbB-3-like	86.98	1.42	2.68	0.01	0.04
106577984	LOC106577984	serine/threonine-protein kinase Nek1-like	4.58	1.42	2.68	0.00	0.00
106607200	LOC106607200	retinol dehydrogenase 8-like	8.39	1.42	2.68	0.00	0.00
106570194	LOC106570194	uncharacterized LOC106570194	22.43	1.42	2.68	0.00	0.00
106604982	LOC106604982	receptor-type tyrosine-protein phosphatase U-l	14.82	1.42	2.67	0.00	0.00
106583826	LOC106583826	phospholipase D4-like	76.98	1.42	2.67	0.00	0.00
106578860	LOC106578860	protein shisa-6 homolog	10.83	1.42	2.67	0.00	0.01
106562261	LOC106562261	cytochrome P450 2C8-like	10.78	1.42	2.67	0.00	0.01

106612535	LOC106612535	alpha-2-macroglobulin-like	65.14	1.42	2.67	0.00	0.00
100136448	LOC100136448	C type lectin receptor C	1837.32	1.42	2.67	0.00	0.00
106565723	LOC106565723	bactericidal permeability-increasing protein-like	40.92	1.42	2.67	0.00	0.02
100195196	ebi2	EBV-induced G-protein coupled receptor 2	18.26	1.42	2.67	0.00	0.00
106601675	LOC106601675	uncharacterized LOC106601675	15.72	1.42	2.67	0.00	0.00
106613694	LOC106613694	lipoprotein lipase-like	22.12	1.41	2.66	0.00	0.01
106567571	LOC106567571	H-2 class II histocompatibility antigen, A-K alpha	6.10	1.41	2.66	0.00	0.03
106607672	cnksr3	CNKSR family member 3	27.29	1.41	2.65	0.00	0.03
106600728	LOC106600728	protein yippee-like 3	19.90	1.41	2.65	0.00	0.03
106564565	LOC106564565	probable ATP-dependent RNA helicase DDX17	108.54	1.41	2.65	0.00	0.00
106565675	LOC106565675	uncharacterized LOC106565675	55.45	1.41	2.65	0.00	0.00
106587141	LOC106587141	adhesion G-protein coupled receptor G5-like	7.49	1.41	2.65	0.00	0.00
106575134	LOC106575134	integrin alpha-4-like	18.10	1.40	2.65	0.01	0.05
106582378	LOC106582378	frizzled-5-like	37.35	1.40	2.65	0.00	0.00
100380427	errfi	ERBB receptor feedback inhibitor 1	181.94	1.40	2.64	0.00	0.00
106579241	LOC106579241	uncharacterized LOC106579241	117.22	1.40	2.64	0.00	0.00
106602440	LOC106602440	calmin-like	42.36	1.40	2.64	0.00	0.02
106567447	LOC106567447	CREB3 regulatory factor-like	32.11	1.40	2.63	0.00	0.00
106603793	LOC106603793	dapper homolog 3-like	32.84	1.40	2.63	0.00	0.00
106564971	LOC106564971	myeloid-associated differentiation marker-like	15.27	1.39	2.63	0.00	0.01
106577579	LOC106577579	protein NLRC3-like	44.54	1.39	2.62	0.00	0.01
106612469	LOC106612469	nucleolar RNA helicase 2-like	344.51	1.39	2.62	0.00	0.01
106588912	lrrc14b	leucine rich repeat containing 14B	8.07	1.39	2.62	0.00	0.00
106576894	cdh23	cadherin related 23	6.32	1.39	2.62	0.01	0.04
106567362	LOC106567362	uncharacterized LOC106567362	17.24	1.39	2.62	0.00	0.00
106611750	LOC106611750	uncharacterized LOC106611750	19.12	1.39	2.62	0.00	0.02
106599812	pcmtd1	protein-L-isoaspartate (D-aspartate) O-methyltr	32.80	1.39	2.61	0.00	0.03
100195718	tp8l2	Tumor necrosis factor, alpha-induced protein 8-	28.47	1.38	2.61	0.00	0.02
106585516	LOC106585516	E3 ubiquitin-protein ligase DTX1-like	10.53	1.38	2.61	0.00	0.00
106572200	tp73	NA	14.84	1.38	2.61	0.00	0.00
106612780	LOC106612780	uncharacterized LOC106612780	25.93	1.38	2.61	0.00	0.00
106613765	LOC106613765	cyclic AMP-responsive element-binding protein	540.20	1.38	2.60	0.00	0.00
106603981	LOC106603981	von Willebrand factor A domain-containing pro	87.67	1.38	2.60	0.00	0.00
100136926	cyp1a	cytochrome P450 1A	2301.57	1.38	2.60	0.00	0.03
106589305	LOC106589305	glucose-6-phosphatase-like	451.08	1.38	2.60	0.00	0.00
106564108	LOC106564108	bifunctional apoptosis regulator-like	73.10	1.38	2.60	0.00	0.00
106609487	LOC106609487	Ig kappa chain C region, B allele-like	12.14	1.37	2.59	0.00	0.00
106587398	LOC106587398	cytochrome P450 2D15-like	3575.36	1.37	2.59	0.00	0.01
106563416	LOC106563416	suppressor of tumorigenicity 14 protein-like	151.83	1.37	2.59	0.00	0.00
106565694	LOC106565694	transcription factor EB-like	5.70	1.37	2.59	0.00	0.02

106588303	LOC106588303	Ig kappa chain V-I region Walker-like	29.15	1.37	2.59	0.00	0.00
106566524	LOC106566524	protein Wnt-5a-like	34.63	1.37	2.59	0.00	0.00
100380371	mald2	MARVEL domain-containing protein 2	41.53	1.37	2.59	0.00	0.00
106607110	smdt1	NA	21.22	1.37	2.59	0.00	0.00
106608166	LOC106608166	GTPase IMAP family member 7-like	5.96	1.37	2.58	0.00	0.00
106597311	LOC106597311	C-C chemokine receptor type 9-like	5.86	1.37	2.58	0.00	0.00
100196705	bpi	Bactericidal permeability-increasing protein	175.09	1.37	2.58	0.00	0.03
106609551	LOC106609551	Ig kappa chain V-III region MOPC 63-like	6.64	1.37	2.58	0.00	0.04
106598452	LOC106598452	cAMP-specific 3',5'-cyclic phosphodiesterase 4B	12.74	1.37	2.58	0.00	0.00
106582501	LOC106582501	interferon-induced guanylate-binding protein 1	27.25	1.36	2.58	0.00	0.02
100196655	hsp70-3	heat shock protein 70	44.60	1.36	2.58	0.00	0.01
106610443	LOC106610443	CD209 antigen-like protein C	7.62	1.36	2.57	0.00	0.00
106599240	LOC106599240	nucleolar GTP-binding protein 1-like	19.30	1.36	2.57	0.00	0.01
106579929	LOC106579929	4-hydroxyphenylpyruvate dioxygenase-like	8.25	1.36	2.57	0.00	0.00
106602253	cdkl1	cyclin dependent kinase like 1	10.03	1.36	2.57	0.00	0.00
106564448	LOC106564448	cobra venom factor-like	121.75	1.36	2.56	0.01	0.04
106582403	LOC106582403	rho-related GTP-binding protein RhoE-like	43.04	1.36	2.56	0.00	0.03
106607151	LOC106607151	interferon-induced very large GTPase 1-like	49.01	1.36	2.56	0.00	0.00
106609289	LOC106609289	plexin-C1-like	28.28	1.36	2.56	0.00	0.00
106576386	LOC106576386	uncharacterized LOC106576386	53.34	1.35	2.55	0.00	0.00
106603177	LOC106603177	thiosulfate sulfurtransferase/rhodanese-like do	1011.41	1.35	2.55	0.00	0.00
106583965	LOC106583965	uncharacterized LOC106583965	6.76	1.35	2.55	0.00	0.02
106599714	LOC106599714	solute carrier family 22 member 23-like	95.58	1.35	2.54	0.00	0.00
106611608	LOC106611608	uncharacterized LOC106611608	5.74	1.35	2.54	0.00	0.00
106565679	LOC106565679	integrin beta-7-like	33.85	1.35	2.54	0.00	0.00
106606324	LOC106606324	RING finger protein 122-like	17.79	1.34	2.54	0.00	0.00
106564996	LOC106564996	C-C chemokine receptor type 7-like	3.98	1.34	2.54	0.01	0.04
106563598	LOC106563598	uncharacterized LOC106563598	1164.46	1.34	2.54	0.00	0.00
106607026	LOC106607026	zinc finger protein Gfi-1b-like	3.99	1.34	2.53	0.01	0.05
106604844	LOC106604844	H-2 class II histocompatibility antigen gamma cl	2367.88	1.34	2.53	0.00	0.00
106585402	LOC106585402	adhesion G protein-coupled receptor E2-like	5.95	1.34	2.53	0.00	0.00
106575090	cpo	carboxypeptidase O	130.47	1.34	2.53	0.00	0.00
106588784	snrk	NA	13.74	1.34	2.53	0.00	0.03
106573469	LOC106573469	RNA polymerase II elongation factor ELL2-like	6.71	1.34	2.53	0.01	0.04
106575703	LOC106575703	uncharacterized LOC106575703	82.53	1.34	2.53	0.00	0.03
106589068	LOC106589068	low density lipoprotein receptor adapter protei	415.63	1.34	2.52	0.00	0.03
106602774	LOC106602774	protein NLRC3-like	5.63	1.33	2.52	0.00	0.00
106611871	LOC106611871	HLA class II histocompatibility antigen gamma c	727.36	1.33	2.52	0.00	0.00
106578012	LOC106578012	urokinase plasminogen activator surface recept	205.74	1.33	2.52	0.00	0.00
106610559	LOC106610559	uncharacterized LOC106610559	22.89	1.33	2.51	0.00	0.00

106587421	LOC106587421	muscarinic acetylcholine receptor M5-like	16.32	1.33	2.51	0.00	0.00
106566127	LOC106566127	inhibin beta B chain-like	73.21	1.33	2.51	0.00	0.00
106563097	LOC106563097	dimethylglycine dehydrogenase, mitochondrial-	74.52	1.33	2.51	0.00	0.00
100195516	g137a	Integral membrane protein GPR137	77.19	1.33	2.51	0.00	0.00
106612514	LOC106612514	alpha-2-macroglobulin-like	4.81	1.33	2.51	0.00	0.00
106568752	LOC106568752	eukaryotic initiation factor 4A-II	113.13	1.32	2.50	0.00	0.00
106606005	LOC106606005	peroxisomal carnitine O-octanoyltransferase-lik	20.53	1.32	2.50	0.00	0.00
106613637	LOC106613637	interleukin-6 receptor subunit beta-like	5.91	1.32	2.50	0.00	0.00
106579001	LOC106579001	growth/differentiation factor 2-like	75.79	1.32	2.50	0.00	0.00
106613396	LOC106613396	trehalase-like	27.67	1.32	2.50	0.00	0.00
106611581	LOC106611581	teneurin-3-like	6.98	1.32	2.50	0.00	0.00
106579065	LOC106579065	sarcoplasmic/endoplasmic reticulum calcium AT	13.99	1.32	2.49	0.00	0.03
100195074	papl	acid phosphatase 7, tartrate resistant (putative)	358.18	1.32	2.49	0.00	0.00
106583398	LOC106583398	uncharacterized LOC106583398	12.91	1.32	2.49	0.00	0.00
106575674	LOC106575674	cytochrome b-245 heavy chain-like	18.03	1.32	2.49	0.00	0.00
106590142	LOC106590142	EMILIN-2-like	17.04	1.32	2.49	0.00	0.00
106588466	LOC106588466	asialoglycoprotein receptor 1-like	6.58	1.32	2.49	0.00	0.00
106581251	LOC106581251	WD repeat and SOCS box-containing protein 1-l	141.03	1.32	2.49	0.00	0.03
106562030	LOC106562030	uncharacterized LOC106562030	15.42	1.31	2.48	0.00	0.03
106602597	LOC106602597	uncharacterized LOC106602597	34.43	1.31	2.48	0.00	0.02
106602649	LOC106602649	T-cell surface glycoprotein CD5-like	7.85	1.31	2.48	0.00	0.00
106610016	LOC106610016	TNFAIP3-interacting protein 2-like	15.86	1.31	2.48	0.00	0.00
106572058	masp2	mannan binding lectin serine peptidase 2	1114.12	1.31	2.48	0.00	0.00
106605438	LOC106605438	semaphorin-4A-like	14.81	1.31	2.48	0.00	0.00
106578070	LOC106578070	zinc finger protein 813-like	25.27	1.31	2.48	0.00	0.00
106612385	LOC106612385	suppressor of cytokine signaling 3-like	30.41	1.31	2.48	0.00	0.00
106573988	klhl30	kelch like family member 30	15.95	1.31	2.47	0.00	0.00
106601786	LOC106601786	extracellular serine/threonine protein kinase FA	37.65	1.31	2.47	0.00	0.00
106575735	LOC106575735	uncharacterized LOC106575735	74.66	1.31	2.47	0.00	0.00
106583080	LOC106583080	tripartite motif-containing protein 16-like prote	404.37	1.30	2.47	0.00	0.00
106584914	LOC106584914	uncharacterized LOC106584914	14.42	1.30	2.47	0.00	0.03
106579620	LOC106579620	myeloid cell surface antigen CD33-like	20.14	1.30	2.47	0.00	0.00
106613685	LOC106613685	calreticulin-like	3217.46	1.30	2.47	0.00	0.02
106563730	LOC106563730	uncharacterized LOC106563730	657.50	1.30	2.47	0.00	0.00
106612315	LOC106612315	catenin alpha-1-like	8.84	1.30	2.47	0.00	0.00
106587531	LOC106587531	vitamin D 25-hydroxylase-like	11.59	1.30	2.46	0.00	0.00
106612959	LOC106612959	connector enhancer of kinase suppressor of ras	47.92	1.30	2.46	0.00	0.01
106566146	LOC106566146	sterol O-acyltransferase 2-like	417.02	1.30	2.46	0.00	0.00
100380645	LOC100380645	nuclear receptor subfamily 2 group F member 5	82.19	1.30	2.46	0.00	0.00
106566966	renbp	NA	18.96	1.30	2.46	0.00	0.00

106611413	LOC106611413	uncharacterized LOC106611413	38.62	1.30	2.46	0.00	0.00
106578394	pkd1l1	NA	14.09	1.30	2.46	0.00	0.00
106561096	LOC106561096	abhydrolase domain-containing protein 2-B-like	6.62	1.30	2.46	0.00	0.00
106566613	LOC106566613	uncharacterized LOC106566613	19.22	1.30	2.46	0.00	0.00
106585831	mpeg1	macrophage expressed 1	309.40	1.30	2.46	0.00	0.00
106586999	LOC106586999	G-protein coupled receptor 56-like	27.18	1.30	2.45	0.00	0.00
106564646	LOC106564646	rab11 family-interacting protein 4A-like	13.14	1.29	2.45	0.00	0.03
106565851	LOC106565851	nicotinamide phosphoribosyltransferase-like	38.12	1.29	2.45	0.00	0.01
106573818	LOC106573818	calreticulin-like	7257.04	1.29	2.45	0.00	0.02
106589597	fam20a	FAM20A, golgi associated secretory pathway ps	22.16	1.29	2.45	0.00	0.00
106605375	tp53inp1	NA	98.72	1.29	2.45	0.00	0.01
106562666	LOC106562666	E3 SUMO-protein ligase PIAS1-like	5.42	1.29	2.45	0.00	0.00
106578999	LOC106578999	retinol-binding protein 3-like	90.19	1.29	2.45	0.00	0.00
106570109	LOC106570109	pre-B-cell leukemia transcription factor-interact	93.63	1.29	2.44	0.00	0.00
106611254	LOC106611254	B-cell lymphoma/leukemia 11B-like	8.88	1.29	2.44	0.00	0.00
106575306	LOC106575306	bile salt export pump-like	5641.03	1.29	2.44	0.00	0.00
106601599	sos2	NA	9.83	1.29	2.44	0.00	0.00
106563046	LOC106563046	junction-mediating and -regulatory protein-like	67.34	1.29	2.44	0.00	0.00
106582148	LOC106582148	cystathionine beta-synthase-like	262.46	1.29	2.44	0.00	0.00
106569469	LOC106569469	uncharacterized LOC106569469	43.27	1.29	2.44	0.00	0.00
100194971	aqp1	Aquaporin-1	47.04	1.29	2.44	0.00	0.00
100195230	rnd3	Rho-related GTP-binding protein RhoE	21.86	1.29	2.44	0.01	0.05
106577690	LOC106577690	UDP-glucuronosyltransferase 1-2-like	76.46	1.28	2.44	0.00	0.00
106570596	LOC106570596	SLIT-ROBO Rho GTPase-activating protein 3-like	6.95	1.28	2.44	0.00	0.01
106612131	cystm1	cysteine rich transmembrane module containin	29.78	1.28	2.44	0.00	0.00
106568527	LOC106568527	cyclin-G2-like	33.08	1.28	2.44	0.00	0.00
100195321	arsk	arylsulfatase family member K	44.94	1.28	2.44	0.00	0.04
106573833	LOC106573833	sphingosine 1-phosphate receptor 4-like	7.87	1.28	2.43	0.00	0.00
100136551	igfbp-2b1	IGF binding protein 3	85.58	1.28	2.43	0.00	0.00
106586330	LOC106586330	rho GTPase-activating protein 15-like	34.16	1.28	2.43	0.00	0.00
106576376	LOC106576376	differentially expressed in FDCP 6-like	49.41	1.28	2.43	0.00	0.00
106566297	LOC106566297	translation initiation factor IF-2-like	14.15	1.28	2.43	0.00	0.02
106571891	LOC106571891	uncharacterized LOC106571891	7.44	1.28	2.43	0.01	0.04
106562131	LOC106562131	UDP-glucuronosyltransferase 2A1 pseudogene	21.79	1.28	2.43	0.00	0.00
101448065	igfbp-1b2	insulin-like growth factor binding protein 1 para	45.94	1.28	2.43	0.00	0.01
106604806	LOC106604806	uncharacterized LOC106604806	13.48	1.28	2.43	0.00	0.02
106567524	LOC106567524	transcription factor 7-like 2	14.39	1.28	2.43	0.00	0.00
106603196	LOC106603196	calphotin-like	4.68	1.28	2.43	0.00	0.00
106605595	LOC106605595	RNA polymerase II degradation factor 1-like	10.97	1.28	2.43	0.00	0.00
100194635	4ebp	eukaryotic translation initiation factor 4E bindir	121.57	1.28	2.43	0.00	0.00

106606551	LOC106606551	nuclear GTPase SLIP-GC-like	46.80	1.28	2.43	0.00	0.01
106575093	LOC106575093	T-cell-specific surface glycoprotein CD28-like	14.18	1.28	2.42	0.00	0.01
106603980	LOC106603980	coronin-6-like	8.44	1.28	2.42	0.00	0.00
106573722	herpud1	homocysteine inducible ER protein with ubiquit	57.53	1.28	2.42	0.00	0.00
106568576	LOC106568576	SOSS complex subunit C-like	11.43	1.27	2.42	0.00	0.00
106576058	strip2	NA	51.53	1.27	2.42	0.00	0.00
106606746	LOC106606746	interferon-induced protein 44-like	24.70	1.27	2.42	0.00	0.00
100136378	LOC100136378	nuclear receptor subfamily 1 group D member 2	61.84	1.27	2.42	0.00	0.00
106606374	LOC106606374	immunoglobulin lambda-like polypeptide 1	17.75	1.27	2.42	0.00	0.03
106576222	LOC106576222	uncharacterized LOC106576222	25.77	1.27	2.42	0.00	0.00
100136501	LOC100136501	hemicentin-1	6.32	1.27	2.41	0.01	0.04
106600810	LOC106600810	perforin-1-like	39.58	1.27	2.41	0.00	0.02
106601178	LOC106601178	NF-kappa-B inhibitor alpha-like	115.36	1.27	2.41	0.00	0.00
106613575	LOC106613575	ephrin-A2-like	7.95	1.27	2.41	0.00	0.03
106578584	LOC106578584	protein phosphatase 1 regulatory subunit 3G-lik	86.51	1.27	2.41	0.01	0.04
106587534	LOC106587534	ras-related protein R-Ras2	162.12	1.27	2.41	0.00	0.00
106569814	LOC106569814	guanine nucleotide exchange factor VAV3-like	6.94	1.27	2.41	0.00	0.00
106585856	tle4	NA	13.47	1.27	2.41	0.00	0.01
106608688	LOC106608688	neurexin-2-like	9.00	1.27	2.41	0.00	0.02
106605693	LOC106605693	meprin A subunit beta-like	9.55	1.27	2.41	0.00	0.02
106580765	LOC106580765	calcineurin B homologous protein 2-like	10.20	1.27	2.41	0.00	0.01
106584208	prkaa2	NA	12.06	1.27	2.40	0.00	0.02
106588373	LOC106588373	uncharacterized LOC106588373	33.14	1.26	2.40	0.00	0.01
106612062	LOC106612062	lymphocyte cytosolic protein 2-like	9.82	1.26	2.40	0.00	0.00
106577197	LOC106577197	catechol O-methyltransferase domain-containir	173.99	1.26	2.40	0.00	0.00
106611251	LOC106611251	HHIP-like protein 1	31.41	1.26	2.40	0.00	0.00
106578585	lck	LCK proto-oncogene, Src family tyrosine kinase	5.37	1.26	2.40	0.00	0.00
106610795	LOC106610795	glutaminyl-peptide cyclotransferase-like	1636.74	1.26	2.40	0.00	0.00
106608585	LOC106608585	echinoderm microtubule-associated protein-like	18.90	1.26	2.40	0.00	0.00
106582614	LOC106582614	uncharacterized LOC106582614	4.60	1.26	2.40	0.00	0.00
106608028	LOC106608028	heme-binding protein 2-like	21.31	1.26	2.40	0.00	0.00
106576895	cssa18h10orf54	chromosome ssa18 open reading frame, humar	24.13	1.26	2.40	0.00	0.00
106574569	LOC106574569	Ig kappa chain C region-like	9.18	1.26	2.39	0.00	0.00
106581305	LOC106581305	solute carrier family 25 member 36-A-like	59.82	1.26	2.39	0.00	0.00
106603835	LOC106603835	solute carrier family 22 member 5-like	16.84	1.26	2.39	0.00	0.00
106566219	LOC106566219	complement C1q-like protein 2	1065.78	1.26	2.39	0.00	0.00
106576207	LOC106576207	protein MCM10 homolog	64.20	1.25	2.39	0.00	0.00
106580932	LOC106580932	zinc finger and BTB domain-containing protein 4	32.35	1.25	2.38	0.00	0.00
106588537	LOC106588537	C-C chemokine receptor type 7-like	40.30	1.25	2.38	0.00	0.03
106584635	LOC106584635	UPF0676 protein C1494.01-like	30.78	1.25	2.38	0.00	0.00

106588197	LOC106588197	eomesodermin-like	6.49	1.25	2.38	0.00	0.00
106568533	onecut2	one cut homeobox 2	7.41	1.25	2.38	0.00	0.00
106609893	LOC106609893	membrane-spanning 4-domains subfamily A me	720.06	1.25	2.38	0.00	0.00
106576185	LOC106576185	hemagglutinin/amebocyte aggregation factor-li	1797.02	1.25	2.38	0.00	0.00
106609083	ccdc166	coiled-coil domain containing 166	20.88	1.25	2.38	0.00	0.00
106562810	LOC106562810	multidrug resistance-associated protein 9-like	17.15	1.25	2.38	0.00	0.03
106610669	LOC106610669	cathepsin L1-like	9.09	1.25	2.37	0.00	0.02
100195689	mog2a	2-acylglycerol O-acyltransferase 2-A	55.01	1.25	2.37	0.00	0.00
106609550	LOC106609550	angiopoietin-related protein 5-like	14.65	1.25	2.37	0.00	0.00
100141317	fxyd5a	FXYD domain containing ion transport regulato	24.67	1.24	2.37	0.00	0.02
106583577	LOC106583577	Fc receptor-like protein 5	9.75	1.24	2.37	0.00	0.00
106585474	LOC106585474	homeobox protein cut-like 2	12.57	1.24	2.37	0.00	0.00
100306796	tmc7	Transmembrane channel-like protein 7	7.46	1.24	2.36	0.00	0.00
106601558	LOC106601558	solute carrier family 25 member 39-like	187.49	1.24	2.36	0.00	0.00
100270807	cybb	cytochrome b-245, beta polypeptide	5.76	1.24	2.36	0.00	0.00
106609354	LOC106609354	ankyrin repeat and SOCS box protein 13-like	13.72	1.24	2.36	0.00	0.02
106589645	LOC106589645	rho GTPase-activating protein 12-like	29.47	1.24	2.36	0.00	0.03
106570212	LOC106570212	alpha-N-acetylgalactosaminide alpha-2,6-sialylt	11.51	1.24	2.36	0.00	0.00
106577910	LOC106577910	SH3 domain and tetratricopeptide repeat-conta	168.70	1.24	2.36	0.00	0.01
106602382	LOC106602382	15-hydroxyprostaglandin dehydrogenase [NAD(	254.95	1.24	2.36	0.00	0.00
100194961	stoml3	stomatin like 3	65.35	1.24	2.36	0.00	0.00
106600931	LOC106600931	nuclear receptor subfamily 5 group A member 2	184.22	1.24	2.36	0.00	0.02
106570091	LOC106570091	secretory carrier-associated membrane protein	14.91	1.23	2.35	0.00	0.00
106566614	LOC106566614	bcl10-interacting CARD protein-like	6.27	1.23	2.35	0.00	0.01
106560553	LOC106560553	complement factor H-like	3163.67	1.23	2.35	0.00	0.00
106607313	LOC106607313	histone-lysine N-methyltransferase EZH1-like	33.82	1.23	2.35	0.00	0.03
106584368	LOC106584368	MAP kinase-interacting serine/threonine-protei	61.13	1.23	2.35	0.00	0.00
106564678	LOC106564678	phosphoinositide 3-kinase regulatory subunit 5-	8.43	1.23	2.35	0.00	0.03
106589602	wipi1	NA	91.58	1.23	2.34	0.00	0.01
106612524	LOC106612524	ribonuclease T2-like	315.13	1.23	2.34	0.00	0.00
106588414	LOC106588414	sodium/potassium-transporting ATPase subunit	9.32	1.23	2.34	0.00	0.00
106578026	LOC106578026	uncharacterized LOC106578026	6.08	1.23	2.34	0.00	0.00
106575036	pros1	NA	775.25	1.23	2.34	0.00	0.00
100286497	nbl1	neuroblastoma, suppression of tumorigenicity 1	101.11	1.23	2.34	0.00	0.04
106567211	LOC106567211	oxysterol-binding protein-related protein 2-like	37.66	1.22	2.33	0.00	0.00
106610616	LOC106610616	integral membrane protein GPR137B-like	58.94	1.22	2.33	0.00	0.00
106567650	LOC106567650	interleukin-31 receptor subunit alpha-like	6.42	1.22	2.33	0.00	0.00
106562750	LOC106562750	cystathionine beta-synthase-like	407.25	1.22	2.33	0.00	0.00
106575663	LOC106575663	uncharacterized LOC106575663	13.23	1.22	2.33	0.00	0.03
106608776	LOC106608776	E3 ubiquitin-protein ligase KEG-like	24.05	1.22	2.33	0.00	0.00

106573891	LOC106573891	uncharacterized LOC106573891	8.94	1.22	2.33	0.00	0.00
106570873	LOC106570873	sonic hedgehog protein-like	1430.59	1.22	2.33	0.01	0.04
106582062	LOC106582062	CD97 antigen-like	17.06	1.22	2.33	0.00	0.02
106561493	LOC106561493	neuron navigator 2-like	124.18	1.22	2.32	0.00	0.00
106601851	LOC106601851	cytochrome P450 2K1-like	7.41	1.22	2.32	0.00	0.02
106574223	LOC106574223	probable serine carboxypeptidase CPVL	12.32	1.22	2.32	0.00	0.03
106603096	chrdl2	chordin like 2	214.36	1.22	2.32	0.00	0.01
106601901	LOC106601901	Ig lambda-2 chain C region-like	9.56	1.22	2.32	0.00	0.00
106575161	LOC106575161	bromodomain adjacent to zinc finger domain pı	39.60	1.22	2.32	0.00	0.00
106570251	LOC106570251	nuclear receptor subfamily 0 group B member 2	795.89	1.21	2.32	0.00	0.01
106576260	LOC106576260	transcription factor ETV6-like	27.70	1.21	2.32	0.00	0.00
106573908	LOC106573908	pyroglutamyl-peptidase 1-like	9.36	1.21	2.32	0.00	0.00
106580864	LOC106580864	cathepsin K-like	163.46	1.21	2.32	0.00	0.02
106562619	LOC106562619	ras GTPase-activating-like protein IQGAP1	10.57	1.21	2.32	0.00	0.00
106613166	LOC106613166	lysophosphatidic acid receptor 4-like	7.13	1.21	2.32	0.00	0.00
106581032	LOC106581032	sterile alpha and TIR motif-containing protein 1	11.78	1.21	2.32	0.00	0.00
100526765	LOC100526765	vascular cell adhesion molecule-like protein	8.07	1.21	2.32	0.01	0.04
106569936	LOC106569936	H-2 class I histocompatibility antigen, Q10 alpha	50.76	1.21	2.32	0.00	0.00
106604665	LOC106604665	large neutral amino acids transporter small sub	14.47	1.21	2.31	0.00	0.00
106598656	LOC106598656	complement factor H-related protein 5-like	25454.24	1.21	2.31	0.00	0.00
106603693	LOC106603693	protein-tyrosine kinase 2-beta-like	21.82	1.21	2.31	0.00	0.04
106580893	map4k1	mitogen-activated protein kinase kinase kinase	29.12	1.21	2.31	0.00	0.00
106571745	LOC106571745	beta-soluble NSF attachment protein	24.28	1.21	2.31	0.00	0.00
100196322	pdl1	Programmed cell death 1 ligand 1	15.79	1.21	2.31	0.00	0.00
106602643	LOC106602643	lectin-like	8.72	1.21	2.31	0.00	0.00
106613816	LOC106613816	nuclear receptor ROR-beta-like	73.06	1.21	2.31	0.00	0.00
106578761	LOC106578761	microfibril-associated glycoprotein 4-like	37.08	1.21	2.31	0.00	0.01
106573028	LOC106573028	tyrosine-protein kinase CSK-like	18.19	1.21	2.31	0.00	0.00
106580234	kctd10	potassium channel tetramerization domain con	23.25	1.20	2.30	0.00	0.00
106577453	LOC106577453	uncharacterized LOC106577453	7.86	1.20	2.30	0.01	0.05
106603289	LOC106603289	oligodendrocyte-myelin glycoprotein-like	21.28	1.20	2.30	0.00	0.00
106590177	LOC106590177	nuclear receptor-binding protein 2-like	7.74	1.20	2.30	0.00	0.03
106579933	LOC106579933	gamma-glutamyltranspeptidase 1-like	13.95	1.20	2.30	0.00	0.00
106610967	LOC106610967	sacsin-like	33.91	1.20	2.30	0.00	0.00
106572414	LOC106572414	FERM domain-containing protein 4B-like	27.64	1.20	2.30	0.00	0.00
106580961	LOC106580961	high affinity immunoglobulin gamma Fc recepto	80.48	1.20	2.30	0.00	0.00
106575707	LOC106575707	tumor necrosis factor receptor superfamily mer	22.19	1.20	2.30	0.01	0.04
106594272	LOC106594272	mitochondrial dynamics protein MID51-like	21.48	1.20	2.29	0.00	0.00
100194515	polr3gl	polymerase (RNA) III (DNA directed) polypeptid	23.79	1.20	2.29	0.00	0.01
106612242	c1qtnf2	C1q and tumor necrosis factor related protein 2	100.09	1.20	2.29	0.00	0.00

106577691	LOC106577691	probable G-protein coupled receptor 132	47.31	1.19	2.29	0.00	0.00
106567335	LOC106567335	zinc finger protein 395-like	116.99	1.19	2.29	0.00	0.00
106602558	LOC106602558	NACHT, LRR and PYD domains-containing prote	89.47	1.19	2.29	0.00	0.00
106571219	nfkbie	NFKB inhibitor epsilon	22.60	1.19	2.29	0.01	0.05
106610861	LOC106610861	phospholipase D4-like	18.42	1.19	2.28	0.00	0.03
106571305	LOC106571305	ribonuclease ZC3H12A-like	22.56	1.19	2.28	0.00	0.00
106604970	LOC106604970	protein-tyrosine kinase 2-beta-like	30.01	1.19	2.28	0.00	0.00
106571703	LOC106571703	protein Z-dependent protease inhibitor-like	177.61	1.19	2.28	0.00	0.00
106581614	LOC106581614	cytochrome P450 2K1-like	68.45	1.19	2.28	0.00	0.03
100194774	pdli2	PDZ and LIM domain protein 2	36.71	1.19	2.28	0.00	0.00
106595288	LOC106595288	60 kDa lysophospholipase-like	142.70	1.19	2.28	0.00	0.00
106611715	LOC106611715	transmembrane protein 35-like	6.12	1.19	2.28	0.00	0.00
106588581	LOC106588581	protein Jumonji-like	54.26	1.19	2.28	0.00	0.01
106588400	LOC106588400	proteasome subunit beta type-8-like	40.96	1.19	2.28	0.00	0.00
100195400	ds22a	Dual specificity protein phosphatase 22-A	9.20	1.19	2.27	0.01	0.04
106590607	LOC106590607	uncharacterized LOC106590607	78.17	1.19	2.27	0.00	0.00
106577132	LOC106577132	protein phosphatase 1 regulatory subunit 3C-B-	212.95	1.18	2.27	0.00	0.00
106572625	LOC106572625	protein-methionine sulfoxide oxidase mical1-lik	25.92	1.18	2.27	0.00	0.00
106571499	LOC106571499	keratinocyte-associated protein 3-like	9.99	1.18	2.27	0.00	0.00
106588354	LOC106588354	tumor necrosis factor alpha-induced protein 8-l	19.30	1.18	2.27	0.00	0.00
106567083	LOC106567083	carboxy-terminal domain RNA polymerase II po	80.85	1.18	2.27	0.00	0.00
100194992	ripl1	RILP-like protein 1	36.07	1.18	2.27	0.00	0.00
106566214	LOC106566214	semaphorin-3D-like	25.30	1.18	2.26	0.00	0.00
106588300	LOC106588300	zinc finger protein 516-like	105.76	1.18	2.26	0.00	0.02
106564592	kdm2b	lysine demethylase 2B	13.62	1.18	2.26	0.00	0.00
106603649	LOC106603649	vacuolar protein sorting-associated protein 11 h	54.05	1.18	2.26	0.00	0.01
106563272	LOC106563272	regulator of G-protein signaling 3-like	107.50	1.18	2.26	0.00	0.00
106562530	LOC106562530	uncharacterized LOC106562530	30.31	1.17	2.26	0.00	0.00
106563698	LOC106563698	complement factor B-like	17109.24	1.17	2.26	0.00	0.00
106588446	LOC106588446	thyrotroph embryonic factor-like	63.10	1.17	2.26	0.00	0.00
106567542	LOC106567542	vasodilator-stimulated phosphoprotein-like	13.96	1.17	2.25	0.00	0.00
106613085	LOC106613085	protein EVI2B-like	15.88	1.17	2.25	0.00	0.01
106604151	LOC106604151	glutathione peroxidase 3-like	147.78	1.17	2.25	0.00	0.00
106609445	LOC106609445	adenosine deaminase CECR1-A-like	12.03	1.17	2.25	0.00	0.00
106610386	LOC106610386	adapter protein CIKS-like	20.84	1.17	2.25	0.00	0.00
106606027	LOC106606027	magnesium transporter MRS2 homolog, mitoch	22.99	1.17	2.25	0.00	0.04
106573491	LOC106573491	mucosa-associated lymphoid tissue lymphoma	33.29	1.17	2.25	0.00	0.00
106605920	LOC106605920	complement C4-like	14980.30	1.17	2.25	0.00	0.00
106577279	LOC106577279	interferon-induced very large GTPase 1-like	37.13	1.17	2.25	0.00	0.02
106568351	LOC106568351	protein prune homolog 2-like	50.39	1.17	2.25	0.01	0.04

106607310	LOC106607310	NACHT, LRR and PYD domains-containing prote	9.11	1.17	2.25	0.00	0.02
106585699	LOC106585699	alpha-1-antitrypsin-like	9639.31	1.17	2.24	0.01	0.04
106561328	LOC106561328	ubiquitin-conjugating enzyme E2 H-like	100.02	1.17	2.24	0.00	0.00
106588304	LOC106588304	Ig kappa chain V region Mem5-like	38.74	1.16	2.24	0.00	0.00
106576384	LOC106576384	Ig kappa-b4 chain C region-like	11.28	1.16	2.24	0.00	0.00
106570897	LOC106570897	probable bifunctional methylenetetrahydrofola	31.44	1.16	2.24	0.00	0.00
106581592	LOC106581592	protein NLRC3-like	10.41	1.16	2.24	0.00	0.00
106573445	LOC106573445	uncharacterized LOC106573445	61.22	1.16	2.24	0.00	0.00
100136577	LOC100136577	H-2 class II histocompatibility antigen, I-E beta c	632.44	1.16	2.24	0.01	0.04
100286569	rpc7l	DNA-directed RNA polymerase III subunit RPC7-	67.90	1.16	2.24	0.00	0.00
106582073	LOC106582073	phospholipase A1 member A-like	916.17	1.16	2.24	0.00	0.00
106607854	LOC106607854	uncharacterized LOC106607854	91.79	1.16	2.24	0.00	0.00
106604722	LOC106604722	solute carrier family 25 member 43-like	31.62	1.16	2.24	0.00	0.03
106604703	LOC106604703	sestrin-3-like	35.73	1.16	2.24	0.00	0.00
106572207	arhgef16	Rho guanine nucleotide exchange factor 16	16.02	1.16	2.23	0.00	0.02
106578930	LOC106578930	NACHT, LRR and PYD domains-containing prote	11.45	1.16	2.23	0.00	0.00
106569947	LOC106569947	major histocompatibility complex class I-relatec	710.69	1.16	2.23	0.00	0.00
106608695	LOC106608695	uncharacterized LOC106608695	18.72	1.16	2.23	0.00	0.00
106561275	LOC106561275	uncharacterized LOC106561275	146.01	1.16	2.23	0.00	0.00
106572492	LOC106572492	LETM1 domain-containing protein 1-like	43.50	1.15	2.22	0.00	0.00
106607245	manba	mannosidase beta	15.51	1.15	2.22	0.00	0.00
106588380	LOC106588380	class I histocompatibility antigen, F10 alpha cha	842.86	1.15	2.22	0.00	0.01
106605836	LOC106605836	CD209 antigen-like protein E	8.38	1.15	2.22	0.00	0.00
106578021	LOC106578021	purine nucleoside phosphorylase-like	90.02	1.15	2.22	0.00	0.00
106613902	LOC106613902	calreticulin-like	398.00	1.15	2.22	0.00	0.00
106611471	vps39	NA	47.76	1.15	2.22	0.00	0.02
106560275	LOC106560275	exocyst complex component 1-like	80.78	1.15	2.22	0.00	0.00
106567248	LOC106567248	wiskott-Aldrich syndrome protein-like	26.44	1.15	2.22	0.00	0.00
106566443	LOC106566443	nuclear factor 7, brain-like	17.03	1.15	2.22	0.00	0.00
106609846	LOC106609846	beta-2-microglobulin-like	130.29	1.15	2.21	0.00	0.00
100136932	p38a	mitogen activated protein kinase p38a	77.76	1.15	2.21	0.00	0.01
106563938	LOC106563938	serine/arginine-rich splicing factor 1B-like	46.36	1.15	2.21	0.00	0.00
106571879	LOC106571879	interleukin-17 receptor E-like	31.35	1.15	2.21	0.00	0.00
106574872	LOC106574872	zinc finger protein 345-like	27.00	1.14	2.21	0.00	0.00
106609751	LOC106609751	tetraspanin-8-like	52.45	1.14	2.21	0.00	0.00
106600234	LOC106600234	leucine-rich repeat-containing protein 16B-like	14.04	1.14	2.21	0.00	0.00
100194991	tm163	Transmembrane protein 163	14.99	1.14	2.21	0.00	0.02
106578760	LOC106578760	microfibril-associated glycoprotein 4-like	82.99	1.14	2.21	0.00	0.00
106588596	LOC106588596	CMP-N-acetylneuraminate-beta-galactosamide-	536.30	1.14	2.21	0.00	0.00
106602392	LOC106602392	transmembrane protein 154-like	8.13	1.14	2.21	0.00	0.03

106574850	LOC106574850	long-chain specific acyl-CoA dehydrogenase, mi	10.56	1.14	2.21	0.00	0.00
106575749	LOC106575749	mannan-binding lectin serine protease 1-like	1098.69	1.14	2.20	0.00	0.02
106562881	LOC106562881	aftiphilin-like	20.93	1.14	2.20	0.00	0.00
106581876	LOC106581876	UDP-glucuronosyltransferase-like	82.33	1.14	2.20	0.00	0.02
106607255	LOC106607255	alpha-2,8-sialyltransferase 8E-like	260.35	1.14	2.20	0.00	0.00
106613581	LOC106613581	syntaxin-6-like	25.16	1.14	2.20	0.00	0.01
106574320	LOC106574320	ephrin-A1-like	60.33	1.14	2.20	0.00	0.01
106608986	LOC106608986	protein NDRG2	35.47	1.14	2.20	0.00	0.00
106606451	LOC106606451	cytochrome P450 2K1-like	289.10	1.13	2.19	0.00	0.02
106612115	LOC106612115	SH2 domain-containing protein 4A-like	13.60	1.13	2.19	0.01	0.04
106598526	LOC106598526	regulator of G-protein signaling 21-like	40.77	1.13	2.19	0.00	0.02
106573398	LOC106573398	complement C4-B	49.71	1.13	2.19	0.00	0.00
106577639	LOC106577639	active breakpoint cluster region-related protein	30.27	1.13	2.19	0.00	0.01
106565283	LOC106565283	beta-1,3-galactosyl-O-glycosyl-glycoprotein beta	11.16	1.13	2.19	0.00	0.00
106606375	LOC106606375	Ig heavy chain Mem5-like	34.96	1.13	2.19	0.00	0.00
106572888	LOC106572888	epidermal growth factor receptor kinase substr	46.30	1.13	2.18	0.00	0.00
106569604	LOC106569604	insulin-like growth factor 1 receptor	14.01	1.13	2.18	0.00	0.00
106605280	LOC106605280	deoxyribonuclease-2-alpha-like	46.92	1.13	2.18	0.00	0.00
106582088	LOC106582088	poly [ADP-ribose] polymerase 4-like	287.46	1.13	2.18	0.01	0.04
106604509	LOC106604509	uncharacterized LOC106604509	26.93	1.13	2.18	0.00	0.00
106565467	LOC106565467	cholesterol desaturase daf-36-like	99.19	1.13	2.18	0.00	0.01
100195695	urah	HIU hydrolase	87.36	1.13	2.18	0.00	0.00
106585863	rasef	NA	48.67	1.12	2.18	0.00	0.00
106577575	LOC106577575	proline-rich receptor-like protein kinase PERK2	89.06	1.12	2.18	0.00	0.00
106607261	LOC106607261	uncharacterized LOC106607261	20.25	1.12	2.18	0.00	0.00
106573132	LOC106573132	endoplasmin-like	157.58	1.12	2.18	0.00	0.00
106578993	LOC106578993	SUN domain-containing protein 1-like	191.95	1.12	2.18	0.00	0.00
100194878	lck	lymphocyte-specific protein tyrosine kinase	17.53	1.12	2.18	0.00	0.01
106579390	LOC106579390	nucleotide-binding oligomerization domain-con	30.10	1.12	2.17	0.00	0.00
106562605	LOC106562605	glutamine and serine-rich protein 1-like	11.36	1.12	2.17	0.00	0.01
106572807	LOC106572807	tumor necrosis factor receptor superfamily mer	34.08	1.12	2.17	0.00	0.01
106580289	LOC106580289	alcohol dehydrogenase [NADP(+)] A-like	79.58	1.12	2.17	0.00	0.00
106564951	LOC106564951	protein AF-17-like	14.37	1.12	2.17	0.00	0.00
106582992	LOC106582992	endothelin-converting enzyme 1-like	187.96	1.12	2.17	0.00	0.00
106569193	LOC106569193	rho GTPase-activating protein 21-like	234.07	1.12	2.17	0.00	0.00
106560381	LOC106560381	maternal DNA replication licensing factor mcm3	17.95	1.12	2.17	0.00	0.00
106579322	LOC106579322	uncharacterized LOC106579322	22.12	1.12	2.17	0.00	0.00
106611569	LOC106611569	probable carboxypeptidase X1	25.32	1.12	2.17	0.00	0.00
106610290	LOC106610290	protein FAM13A-like	120.47	1.11	2.16	0.01	0.04
100380298	nfkb2	nuclear factor of kappa light polypeptide gene e	62.65	1.11	2.16	0.01	0.04

106561918	LOC106561918	neuron navigator 2-like	75.24	1.11	2.16	0.00	0.02
106602019	LOC106602019	glucagon receptor-like	22.44	1.11	2.16	0.01	0.04
100137055	csf3r	granulocyte colony-stimulating factor receptor	46.70	1.11	2.16	0.00	0.00
106562835	LOC106562835	probable palmitoyltransferase ZDHHC12	13.55	1.11	2.16	0.00	0.00
106563799	LOC106563799	telomerase-binding protein EST1A-like	20.10	1.11	2.16	0.00	0.02
106611733	LOC106611733	coagulation factor IX-like	2784.12	1.11	2.16	0.00	0.00
106601960	LOC106601960	rho GDP-dissociation inhibitor 2-like	57.21	1.11	2.16	0.00	0.00
106609962	LOC106609962	plasma protease C1 inhibitor-like	14190.81	1.11	2.16	0.00	0.00
106579125	cd79b	CD79b molecule	36.80	1.11	2.16	0.00	0.00
106584226	LOC106584226	SUN domain-containing ossification factor-like	12.72	1.11	2.15	0.00	0.00
106567569	LOC106567569	hepatocyte cell adhesion molecule-like	22.75	1.11	2.15	0.00	0.00
106560893	LOC106560893	tyrosine aminotransferase-like	6965.88	1.11	2.15	0.00	0.00
100195547	cd53	Leukocyte surface antigen CD53	113.70	1.11	2.15	0.00	0.00
106561892	LOC106561892	glycoprotein endo-alpha-1,2-mannosidase-like	58.34	1.11	2.15	0.00	0.03
106568211	LOC106568211	uncharacterized threonine-rich GPI-anchored gl	23.39	1.10	2.15	0.01	0.04
100306863	mmp25	Matrix metalloproteinase-25	8.86	1.10	2.15	0.00	0.00
106573817	LOC106573817	UV excision repair protein RAD23 homolog B-lik	25.33	1.10	2.15	0.00	0.00
106601527	ubald2	NA	24.37	1.10	2.15	0.00	0.01
106562284	LOC106562284	interferon regulatory factor 8-like	33.54	1.10	2.15	0.00	0.01
106589380	LOC106589380	polycomb group RING finger protein 5-B-like	19.84	1.10	2.15	0.00	0.02
106601029	LOC106601029	serine/threonine-protein kinase 17A-like	54.51	1.10	2.15	0.01	0.04
106564993	LOC106564993	N-acetylmuramoyl-L-alanine amidase-like	906.49	1.10	2.15	0.00	0.00
100196627	cideb	cell death-inducing DFFA-like effector b	363.31	1.10	2.15	0.00	0.00
106573426	LOC106573426	NACHT, LRR and PYD domains-containing prote	21.43	1.10	2.15	0.00	0.00
106582662	LOC106582662	uncharacterized LOC106582662	18.49	1.10	2.15	0.00	0.00
106587004	LOC106587004	far upstream element-binding protein 3-like	113.72	1.10	2.15	0.00	0.01
106571258	LOC106571258	CCAAT/enhancer-binding protein zeta-like	36.33	1.10	2.14	0.00	0.00
106608199	LOC106608199	exostosin-like 3	64.98	1.10	2.14	0.00	0.00
106585519	LOC106585519	filamin-interacting protein FAM101A-like	81.26	1.10	2.14	0.00	0.00
106565889	LOC106565889	endothelin-converting enzyme 1-like	19.15	1.10	2.14	0.00	0.00
106610446	LOC106610446	C-type lectin domain family 4 member E-like	30.14	1.10	2.14	0.00	0.00
106564802	LOC106564802	REST corepressor 3-like	13.59	1.10	2.14	0.00	0.00
106583402	LOC106583402	low affinity immunoglobulin gamma Fc region r	11.92	1.10	2.14	0.00	0.00
106566739	psma7	proteasome subunit alpha 7	101.86	1.10	2.14	0.00	0.01
106603155	LOC106603155	GC-rich sequence DNA-binding factor 2-like	14.38	1.10	2.14	0.00	0.02
106573986	LOC106573986	RING finger protein 186-like	13.59	1.10	2.14	0.00	0.00
106612144	LOC106612144	SH3 domain and tetratricopeptide repeat-conta	59.29	1.10	2.14	0.00	0.04
106573632	LOC106573632	microtubule-associated proteins 1A/1B light cha	212.20	1.09	2.14	0.00	0.01
106582667	LOC106582667	rho GTPase-activating protein 15-like	20.46	1.09	2.14	0.00	0.00
106581519	LOC106581519	small G protein signaling modulator 2-like	16.27	1.09	2.13	0.00	0.00

106612915	LOC106612915	thioredoxin domain-containing protein 17-like	12.63	1.09	2.13	0.00	0.03
106612677	LOC106612677	uncharacterized LOC106612677	14.20	1.09	2.13	0.00	0.00
106585671	LOC106585671	ARF GTPase-activating protein GIT2-like	21.78	1.09	2.13	0.00	0.02
106570361	LOC106570361	zinc finger protein 40-like	21.45	1.09	2.13	0.00	0.03
106590377	LOC106590377	NEDD8 ultimate buster 1-like	32.62	1.09	2.13	0.00	0.00
106602134	LOC106602134	alpha-2-macroglobulin receptor-associated prot	26.63	1.09	2.12	0.00	0.00
106588312	LOC106588312	semaphorin-4A-like	152.09	1.09	2.12	0.00	0.00
106588637	LOC106588637	palmitoyltransferase ZDHHC18-like	14.09	1.09	2.12	0.00	0.00
106589754	LOC106589754	ZP domain-containing protein-like	28.15	1.09	2.12	0.00	0.00
106613397	LOC106613397	trehalase-like	62.27	1.09	2.12	0.00	0.00
106576448	LOC106576448	T-complex protein 11-like protein 2	62.39	1.09	2.12	0.00	0.00
106567405	LOC106567405	carbohydrate-responsive element-binding prote	18.36	1.09	2.12	0.00	0.00
100194694	sqstm1	NA	222.99	1.09	2.12	0.00	0.00
106578982	LOC106578982	retinol-binding protein 4-B	16165.70	1.08	2.12	0.00	0.03
106573249	LOC106573249	mitochondrial inner membrane protease subun	310.36	1.08	2.12	0.00	0.00
106562340	LOC106562340	transmembrane protein 150C-like	48.81	1.08	2.12	0.00	0.03
106607153	LOC106607153	interferon-induced very large GTPase 1-like	126.44	1.08	2.11	0.00	0.00
106580940	LOC106580940	tumor necrosis factor, alpha-induced protein 8-	19.92	1.08	2.11	0.00	0.01
106612599	LOC106612599	mitogen-activated protein kinase kinase kinase	71.57	1.08	2.11	0.00	0.00
106564407	LOC106564407	Ig kappa chain V-IV region JI-like	17.38	1.08	2.11	0.00	0.00
100195198	vps37a	VPS37A, ESCRT-I subunit	222.69	1.08	2.11	0.00	0.00
100136583	LOC100136583	pentraxin	13515.19	1.07	2.11	0.00	0.00
106593876	LOC106593876	cytochrome P450 3A27-like	27.50	1.07	2.10	0.00	0.00
106612823	LOC106612823	claudin-3-like	246.14	1.07	2.10	0.00	0.01
106576265	LOC106576265	chromodomain-helicase-DNA-binding protein 4	147.27	1.07	2.10	0.00	0.00
106587061	LOC106587061	ankyrin repeat and BTB/POZ domain-containing	16.60	1.07	2.10	0.00	0.03
106585399	LOC106585399	lipocalin-like	1512.07	1.07	2.10	0.00	0.00
106610660	LOC106610660	ribosomal protein S6 kinase 2 alpha-like	38.46	1.07	2.10	0.01	0.04
106580790	LOC106580790	inositol polyphosphate 5-phosphatase K-like	73.83	1.07	2.10	0.00	0.01
106567494	LOC106567494	protein sprouty homolog 4-like	20.03	1.07	2.10	0.00	0.00
106578142	LOC106578142	prospero homeobox protein 1-like	72.82	1.07	2.10	0.00	0.00
106567382	LOC106567382	large neutral amino acids transporter small sub	16.36	1.07	2.10	0.00	0.00
106588184	LOC106588184	secretory carrier-associated membrane protein	14.94	1.07	2.10	0.00	0.00
106581235	LOC106581235	dehydrogenase/reductase SDR family member	79.18	1.07	2.10	0.00	0.00
106567101	LOC106567101	WNT1-inducible-signaling pathway protein 2-lik	24.70	1.07	2.09	0.01	0.04
100380390	herp1	Homocysteine-responsive endoplasmic reticulu	274.75	1.07	2.09	0.00	0.00
106585107	LOC106585107	rho GTPase-activating protein 25-like	34.03	1.07	2.09	0.00	0.00
106609518	LOC106609518	sodium-coupled neutral amino acid transporter	1835.26	1.07	2.09	0.00	0.00
106584853	LOC106584853	death-associated protein kinase 2-like	19.18	1.06	2.09	0.00	0.04
106582838	bpifb2	BPI fold containing family B member 2	71.99	1.06	2.09	0.00	0.03

106598945	LOC106598945	CMP-N-acetylneuraminate-beta-1,4-galactoside	730.79	1.06	2.09	0.00	0.00
100195235	acd10	Acyl-CoA dehydrogenase family member 10	38.49	1.06	2.09	0.00	0.00
106566186	LOC106566186	ATP-dependent RNA helicase DDX54-like	27.72	1.06	2.09	0.00	0.00
106565972	LOC106565972	vacuolar fusion protein MON1 homolog B-like	42.99	1.06	2.09	0.00	0.01
100195198	vps37a	VPS37A, ESCRT-I subunit	97.76	1.06	2.09	0.00	0.00
106611057	LOC106611057	protein angel homolog 1-like	47.39	1.06	2.09	0.00	0.01
106612913	LOC106612913	schwannomin-interacting protein 1-like	44.10	1.06	2.09	0.00	0.00
106570183	LOC106570183	protein Jumonji-like	184.03	1.06	2.08	0.00	0.01
100196890	calcoco2	calcium binding and coiled-coil domain 2	1628.27	1.06	2.08	0.00	0.00
106604826	LOC106604826	dedicator of cytokinesis protein 2-like	38.49	1.06	2.08	0.00	0.02
106569441	LOC106569441	myosin heavy chain, striated muscle-like	5315.57	1.06	2.08	0.00	0.00
106606455	LOC106606455	cytochrome P450 2K3-like	2090.72	1.06	2.08	0.00	0.01
106563917	LOC106563917	T-cell surface protein tactile-like	27.92	1.06	2.08	0.00	0.00
106589649	fam120b	family with sequence similarity 120B	28.35	1.06	2.08	0.00	0.00
106579758	LOC106579758	interferon-induced guanylate-binding protein 1	109.74	1.06	2.08	0.00	0.00
106566589	lime1	Lck interacting transmembrane adaptor 1	13.29	1.06	2.08	0.00	0.00
106611349	LOC106611349	sodium-dependent multivitamin transporter-lik	276.07	1.06	2.08	0.00	0.00
100306743	mrgbp	MRG-binding protein	45.05	1.06	2.08	0.00	0.00
106581857	LOC106581857	peroxisomal N(1)-acetyl-spermine/spermidine c	62.21	1.06	2.08	0.00	0.02
106574315	cpb2	carboxypeptidase B2	1549.44	1.06	2.08	0.00	0.00
106575328	LOC106575328	autophagy-related protein 9A-like	21.90	1.06	2.08	0.00	0.00
106581762	LOC106581762	pyruvate dehydrogenase (acetyl-transferring) ki	24.53	1.05	2.08	0.00	0.03
106586664	LOC106586664	insulin receptor substrate 2-like	97.60	1.05	2.08	0.00	0.00
106589339	LOC106589339	somatostatin receptor type 2-like	14.97	1.05	2.07	0.00	0.02
106569076	LOC106569076	uncharacterized LOC106569076	28.91	1.05	2.07	0.00	0.01
106573229	tcp11l1	NA	44.19	1.05	2.07	0.00	0.02
106587488	LOC106587488	fibrinogen-like protein 1-like protein	1251.75	1.05	2.07	0.00	0.00
106588086	LOC106588086	integrin alpha-L-like	26.38	1.05	2.07	0.00	0.00
100194817	lypa3	1-O-acylceramide synthase	1327.39	1.05	2.07	0.00	0.00
106576019	cldn12	claudin 12	15.69	1.05	2.07	0.00	0.01
106607317	LOC106607317	formin-like protein 1	34.23	1.05	2.07	0.00	0.02
100195444	ripk4	receptor-interacting serine-threonine kinase 4	27.84	1.05	2.07	0.00	0.00
106566126	LOC106566126	R3H domain-containing protein 2-like	35.59	1.05	2.07	0.00	0.00
106611988	LOC106611988	LON peptidase N-terminal domain and RING fin	32.59	1.05	2.07	0.00	0.00
106583643	LOC106583643	bridging integrator 2-like	57.59	1.05	2.07	0.00	0.00
106590081	LOC106590081	succinate--hydroxymethylglutarate CoA-transfe	27.04	1.05	2.07	0.00	0.01
106564667	LOC106564667	uncharacterized LOC106564667	49.19	1.04	2.06	0.00	0.00
106562860	LOC106562860	very long-chain acyl-CoA synthetase-like	193.00	1.04	2.06	0.00	0.00
106605290	LOC106605290	myotubularin-related protein 11-like	26.34	1.04	2.06	0.00	0.03
100194852	grp2	RAS guanyl-releasing protein 2	36.85	1.04	2.06	0.00	0.00

106581031	LOC106581031	vitronectin-like	2658.67	1.04	2.06	0.00	0.00
106566697	smc5	NA	243.09	1.04	2.06	0.00	0.00
106581528	tnfaip1	NA	26.96	1.04	2.06	0.00	0.00
106562108	LOC106562108	ras association domain-containing protein 10-lil	31.12	1.04	2.06	0.00	0.00
100195473	arhgap18	Rho GTPase activating protein 18	292.41	1.04	2.06	0.00	0.01
100526824	tbx21	T-box 21	13.30	1.04	2.06	0.00	0.00
106573436	LOC106573436	complement C3-like	427.85	1.04	2.05	0.00	0.00
106573699	LOC106573699	PQ-loop repeat-containing protein 1-like	130.79	1.04	2.05	0.00	0.02
106603954	LOC106603954	E3 ubiquitin-protein ligase RNF43-like	15.00	1.04	2.05	0.00	0.01
106603538	LOC106603538	diacylglycerol O-acyltransferase 2-like	1010.87	1.04	2.05	0.00	0.01
106607748	LOC106607748	low-density lipoprotein receptor-related protei	37.05	1.04	2.05	0.00	0.00
106590884	LOC106590884	GRB2-related adapter protein-like	18.35	1.04	2.05	0.00	0.00
106585113	LOC106585113	zinc finger protein 703-like	40.98	1.03	2.05	0.00	0.00
106609328	LOC106609328	transcription factor SOX-5-like	93.51	1.03	2.05	0.00	0.00
100196738	tn13b	Tumor necrosis factor ligand superfamily memb	20.41	1.03	2.05	0.00	0.00
106608356	slc2a12	NA	57.72	1.03	2.05	0.00	0.01
106570652	LOC106570652	1-acylglycerol-3-phosphate O-acyltransferase A	60.54	1.03	2.04	0.00	0.01
106580972	LOC106580972	integrator complex subunit 4-like	25.94	1.03	2.04	0.00	0.00
106603586	LOC106603586	E3 ubiquitin-protein ligase Siah2	12.75	1.03	2.04	0.00	0.00
106612651	LOC106612651	sodium-dependent serotonin transporter-like	117.32	1.03	2.04	0.00	0.01
100195203	lcp2	lymphocyte cytosolic protein 2	23.69	1.03	2.04	0.00	0.01
100196216	mfap4	microfibrillar-associated protein 4	249.04	1.03	2.04	0.00	0.00
106570284	ube2e1	ubiquitin conjugating enzyme E2 E1	41.36	1.03	2.04	0.00	0.00
106585263	LOC106585263	proactivator polypeptide-like	331.33	1.03	2.04	0.00	0.01
106594163	LOC106594163	cytochrome P450 3A27-like	252.37	1.03	2.04	0.00	0.00
100380309	tmm34	Transmembrane protein 34	20.38	1.03	2.04	0.00	0.00
106564477	LOC106564477	programmed cell death protein 4-like	26.79	1.02	2.03	0.00	0.00
106565098	LOC106565098	uncharacterized LOC106565098	32.92	1.02	2.03	0.00	0.00
106580824	LOC106580824	membrane frizzled-related protein-like	151.46	1.02	2.03	0.00	0.01
106561059	LOC106561059	NACHT, LRR and PYD domains-containing prote	23.80	1.02	2.03	0.00	0.01
106588341	LOC106588341	protein FAM63A-like	36.87	1.02	2.03	0.00	0.00
106570867	LOC106570867	stromal cell-derived factor 2-like	237.61	1.02	2.03	0.00	0.02
100194875	mfsd2	Major facilitator superfamily domain-containing	18.07	1.02	2.03	0.00	0.01
106606070	LOC106606070	nuclear factor NF-kappa-B p100 subunit-like	114.68	1.02	2.03	0.00	0.00
106585005	LOC106585005	pleckstrin homology domain-containing family /	25.27	1.02	2.03	0.00	0.00
100196462	cats	Cathepsin S	228.91	1.02	2.03	0.00	0.00
106566283	LOC106566283	cellular nucleic acid-binding protein-like	19.69	1.02	2.03	0.00	0.00
106569529	LOC106569529	methyl-CpG-binding domain protein 3-like	23.81	1.02	2.03	0.00	0.00
100195626	zfn2b	AN1-type zinc finger protein 2B	60.84	1.02	2.03	0.00	0.00
106562571	LOC106562571	leucine-rich repeat serine/threonine-protein kir	15.82	1.02	2.02	0.00	0.00

106581831	LOC106581831	kalirin-like	35.60	1.02	2.02	0.00	0.00
106601135	LOC106601135	formin-like protein 1	27.57	1.02	2.02	0.00	0.02
106583228	LOC106583228	protein OS-9-like	426.32	1.02	2.02	0.00	0.00
106562977	LOC106562977	heterogeneous nuclear ribonucleoprotein R	37.28	1.02	2.02	0.00	0.02
106570282	LOC106570282	ubiquitin-conjugating enzyme E2 E2-like	40.79	1.01	2.02	0.00	0.00
106612814	LOC106612814	flotillin-2-like	13.95	1.01	2.02	0.00	0.00
106561913	epb41l4a	erythrocyte membrane protein band 4.1 like 4A	34.96	1.01	2.02	0.01	0.04
106612055	LOC106612055	tyrosine-protein kinase ITK/TSK-like	18.02	1.01	2.02	0.00	0.00
106566120	shmt2	serine hydroxymethyltransferase 2	2739.61	1.01	2.02	0.00	0.00
106585067	LOC106585067	uncharacterized LOC106585067	39.92	1.01	2.02	0.00	0.02
100196878	m4a12	Membrane-spanning 4-domains subfamily A me	1182.31	1.01	2.02	0.00	0.03
106564149	LOC106564149	perforin-1-like	23.38	1.01	2.01	0.00	0.03
100195387	gtpbp4	GTP binding protein 4	798.40	1.01	2.01	0.00	0.01
106570689	LOC106570689	MARCKS-related protein-like	149.27	1.01	2.01	0.00	0.00
106573636	LOC106573636	carbonic anhydrase 5B, mitochondrial-like	116.39	1.01	2.01	0.00	0.00
100194752	kcnk6	potassium two pore domain channel subfamily	162.09	1.01	2.01	0.00	0.00
106565859	LOC106565859	serine/threonine-protein kinase Nek4-like	18.52	1.01	2.01	0.00	0.00
100136496	LOC100136496	FIP2-like	19.22	1.01	2.01	0.00	0.00
106570047	LOC106570047	angiopoietin-1-like	32.90	1.01	2.01	0.00	0.00
106585459	LOC106585459	stromelysin-3-like	230.07	1.01	2.01	0.00	0.00
106571205	tagap	NA	23.56	1.01	2.01	0.00	0.00
106578810	LOC106578810	prosaposin-like	1091.83	1.00	2.01	0.00	0.03
106576316	LOC106576316	PRKC apoptosis WT1 regulator protein-like	174.79	1.00	2.00	0.00	0.00
100380737	LOC100380737	liprin-beta-2	84.28	1.00	2.00	0.00	0.00
106578421	LOC106578421	death-associated protein 1-like	195.46	1.00	2.00	0.00	0.00
106612625	LOC106612625	zinc finger FYVE domain-containing protein 1-lik	65.54	1.00	2.00	0.00	0.03
106610680	LOC106610680	SH3 and cysteine-rich domain-containing protei	43.08	1.00	2.00	0.00	0.00
106611175	LOC106611175	NF-kappa-B inhibitor alpha-like	96.02	1.00	2.00	0.00	0.02
100196506	glipr1	GLI pathogenesis related 1	17.57	1.00	2.00	0.00	0.00
100195398	al3a2	Fatty aldehyde dehydrogenase	31.48	1.00	2.00	0.01	0.04
106583092	LOC106583092	dual serine/threonine and tyrosine protein kina	34.58	1.00	2.00	0.00	0.00
106602690	LOC106602690	plasma protease C1 inhibitor-like	15337.42	1.00	2.00	0.01	0.04
106575986	LOC106575986	kinesin-like protein KIF21A	34.91	1.00	2.00	0.00	0.00
106560901	LOC106560901	vacuolar protein sorting-associated protein 4A	38.26	1.00	2.00	0.00	0.00
106607212	LOC106607212	sphingosine 1-phosphate receptor 2-like	19.28	1.00	2.00	0.00	0.00
100196725	cytip	cytohesin 1 interacting protein	14.07	1.00	2.00	0.00	0.00

Down-regulated DEGs in fry compared to parr

NCBI ID	Gene IDs/Locus	Gene name	baseMean	log2FoldChange	Fold change	pvalue	padj
106587343	LOC106587343	hepatic triacylglycerol lipase-like	90.11	-2.78	-6.86	0.00	0.00
106611136	LOC106611136	ornithine decarboxylase 1-like	477.56	-2.44	-5.44	0.00	0.00

106607066	LOC106607066	importin subunit alpha-1-like	196.78	-2.29	-4.88	0.00	0.02
106601402	LOC106601402	cytoglobin-2-like	13.68	-2.24	-4.72	0.00	0.00
106570339	tubb4b	NA	133.41	-2.23	-4.68	0.00	0.01
106573903	LOC106573903	gamma-interferon-inducible lysosomal thiol rec	23.90	-2.20	-4.60	0.00	0.03
106601395	LOC106601395	importin subunit alpha-1-like	268.96	-2.10	-4.30	0.00	0.00
106582767	LOC106582767	retinoid-binding protein 7-like	1162.40	-2.09	-4.26	0.00	0.00
106568163	ppp1r3b	NA	14.22	-2.09	-4.25	0.00	0.00
106573105	LOC106573105	aurora kinase B-like	81.97	-2.08	-4.23	0.00	0.00
106586917	LOC106586917	tubulin alpha chain	17.98	-2.07	-4.20	0.00	0.01
106570560	LOC106570560	protein FAM65B-like	27.93	-2.07	-4.19	0.00	0.00
106564883	LOC106564883	vitamin K-dependent gamma-carboxylase-like	15.82	-2.07	-4.18	0.01	0.04
106574634	LOC106574634	uncharacterized protein KIAA1211-like	88.53	-2.04	-4.11	0.00	0.00
106598941	pif1	NA	20.13	-2.03	-4.09	0.00	0.00
106578920	LOC106578920	uncharacterized LOC106578920	309.48	-2.03	-4.08	0.00	0.00
106571818	slc27a6	NA	383.01	-2.02	-4.05	0.00	0.00
106612829	LOC106612829	phosphoserine phosphatase-like	375.54	-1.99	-3.98	0.00	0.00
106599162	LOC106599162	cholesterol 7-alpha-monooxygenase-like	1921.45	-1.98	-3.95	0.00	0.00
106590084	LOC106590084	cell death activator CIDE-A-like	40.54	-1.93	-3.81	0.00	0.01
106589612	LOC106589612	fatty acid synthase-like	14603.56	-1.93	-3.80	0.00	0.00
106601868	LOC106601868	cyclin-F-like	38.29	-1.93	-3.80	0.00	0.00
106582230	LOC106582230	arylsulfatase D-like	58.82	-1.92	-3.79	0.00	0.01
106604515	LOC106604515	elongation of very long chain fatty acids protein	4235.24	-1.91	-3.76	0.00	0.00
100195420	pck1	phosphoenolpyruvate carboxykinase 1	22.69	-1.91	-3.76	0.00	0.03
106589095	LOC106589095	sodium-dependent lysophosphatidylcholine syr	83.14	-1.91	-3.76	0.00	0.00
106608861	LOC106608861	prostaglandin reductase 1-like	50.83	-1.91	-3.75	0.00	0.00
106561604	LOC106561604	aurora kinase B-like	127.20	-1.90	-3.74	0.00	0.00
106568948	LOC106568948	mpv17-like protein 2	16.24	-1.90	-3.73	0.00	0.01
106578804	LOC106578804	kinesin-like protein KIF20B	50.12	-1.89	-3.72	0.00	0.00
100196879	dhfr	dihydrofolate reductase	193.17	-1.89	-3.71	0.00	0.00
100195978	hdd1a	Haloacid dehalogenase-like hydrolase domain-c	230.32	-1.89	-3.70	0.00	0.00
106561008	LOC106561008	AFG3-like protein 1	9.90	-1.88	-3.67	0.00	0.00
100196484	topk	Lymphokine-activated killer T-cell-originated pr	166.31	-1.87	-3.67	0.00	0.00
106580039	LOC106580039	long-chain-fatty-acid--CoA ligase ACSBG2-like	669.00	-1.86	-3.63	0.00	0.00
106601288	LOC106601288	ribonucleoprotein PTB-binding 1-like	17.86	-1.86	-3.62	0.00	0.00
100195587	borea	Borealin	126.40	-1.86	-3.62	0.00	0.00
106582050	LOC106582050	potassium voltage-gated channel subfamily E m	9.14	-1.86	-3.62	0.00	0.00
106611586	elovl6	ELOVL fatty acid elongase 6	8752.73	-1.85	-3.60	0.00	0.00
106608632	LOC106608632	histone H2A.Z	207.85	-1.85	-3.60	0.00	0.00
106573666	LOC106573666	carbohydrate sulfotransferase 6-like	44.33	-1.84	-3.59	0.00	0.03
106577852	LOC106577852	vang-like protein 2	12.50	-1.84	-3.58	0.00	0.00

106613922	ect2	epithelial cell transforming 2	54.27	-1.83	-3.56	0.00	0.00
106567090	LOC106567090	tubulin alpha chain	44.21	-1.83	-3.55	0.00	0.00
106600932	LOC106600932	heme-binding protein 2-like	15.87	-1.82	-3.54	0.00	0.01
106607702	LOC106607702	mitotic checkpoint serine/threonine-protein kir	55.65	-1.82	-3.54	0.00	0.00
100195990	cenpn	centromere protein N	31.20	-1.80	-3.48	0.00	0.00
106599713	LOC106599713	tubulin beta-2A chain-like	46.52	-1.79	-3.46	0.01	0.05
106611710	LOC106611710	mid1-interacting protein 1-like	225.90	-1.79	-3.45	0.00	0.02
106607826	LOC106607826	cyclin-dependent kinase 1-like	179.26	-1.77	-3.42	0.00	0.01
106610005	birc5	baculoviral IAP repeat containing 5	119.71	-1.77	-3.40	0.00	0.01
106587299	LOC106587299	SHC SH2 domain-binding protein 1-like	27.38	-1.77	-3.40	0.00	0.01
106566708	LOC106566708	G2/mitotic-specific cyclin-B1-like	183.05	-1.76	-3.38	0.00	0.00
106564629	LOC106564629	glial fibrillary acidic protein-like	14.06	-1.75	-3.36	0.00	0.00
106599389	LOC106599389	kinesin-like protein KIF2C	121.75	-1.75	-3.36	0.01	0.04
106567172	LOC106567172	bridging integrator 2-like	5.92	-1.74	-3.33	0.00	0.00
106613883	LOC106613883	DEP domain-containing protein 1A-like	29.20	-1.74	-3.33	0.00	0.00
106565291	LOC106565291	ubiquitin-conjugating enzyme E2 C-like	162.43	-1.73	-3.32	0.00	0.00
106607886	LOC106607886	uncharacterized LOC106607886	122.90	-1.73	-3.32	0.00	0.00
106569965	LOC106569965	UDP-glucuronosyltransferase 2B20-like	6.14	-1.72	-3.30	0.00	0.00
106605090	LOC106605090	cell division cycle-associated protein 3-like	160.10	-1.72	-3.29	0.00	0.00
106567984	p2rx5	purinergic receptor P2X 5	5.75	-1.71	-3.26	0.00	0.00
106574707	LOC106574707	folate transporter 1-like	16.00	-1.71	-3.26	0.00	0.00
106612658	LOC106612658	phosphatidylinositol transfer protein alpha isof	18.13	-1.71	-3.26	0.01	0.05
106613791	LOC106613791	cyclin-dependent kinases regulatory subunit 1-l	51.62	-1.70	-3.26	0.00	0.00
106579552	oplah	5-oxoprolinase (ATP-hydrolysing)	136.32	-1.70	-3.25	0.00	0.01
106595274	LOC106595274	OX-2 membrane glycoprotein-like	67.94	-1.70	-3.24	0.00	0.00
106585234	LOC106585234	calcineurin B homologous protein 3-like	192.07	-1.69	-3.23	0.00	0.00
100196122	cdc2	Cell division control protein 2 homolog	266.04	-1.69	-3.22	0.00	0.01
106573334	LOC106573334	PCNA-associated factor-like	121.72	-1.69	-3.22	0.00	0.02
106584736	LOC106584736	transcription factor 12-like	10.53	-1.68	-3.21	0.00	0.00
106584748	LOC106584748	protein regulator of cytokinesis 1-like	28.51	-1.68	-3.20	0.00	0.00
106583280	LOC106583280	tubulin alpha-1B chain-like	61.07	-1.67	-3.19	0.00	0.00
100192341	elovl2	ELOVL fatty acid elongase 2	4315.06	-1.67	-3.18	0.01	0.04
106601215	LOC106601215	coiled-coil domain-containing protein 134-like	33.58	-1.67	-3.18	0.00	0.00
106607871	LOC106607871	uncharacterized LOC106607871	20.31	-1.67	-3.17	0.00	0.00
100195204	s2533	Solute carrier family 25 member 33	133.92	-1.66	-3.16	0.00	0.00
106577478	LOC106577478	mitochondrial import receptor subunit TOM40	277.61	-1.65	-3.15	0.00	0.00
106584418	LOC106584418	cyclin-dependent kinases regulatory subunit 1-l	30.39	-1.65	-3.15	0.00	0.00
106583386	LOC106583386	cell division cycle-associated protein 3-like	113.95	-1.65	-3.14	0.00	0.00
106583830	LOC106583830	dual specificity protein kinase Ttk-like	79.04	-1.65	-3.14	0.00	0.00
106610986	LOC106610986	transforming acidic coiled-coil-containing prote	93.74	-1.64	-3.12	0.00	0.00

106612554	LOC106612554	1-acyl-sn-glycerol-3-phosphate acyltransferase	71.05	-1.64	-3.11	0.00	0.00
106582847	LOC106582847	dehydrodolichyl diphosphate syntase complex s	69.95	-1.63	-3.09	0.00	0.00
106602733	LOC106602733	fatty acid-binding protein, intestinal-like	418.74	-1.63	-3.09	0.00	0.00
106606941	LOC106606941	protein SCO1 homolog, mitochondrial-like	37.66	-1.62	-3.08	0.00	0.01
106605431	LOC106605431	thrombospondin-3a-like	10.69	-1.62	-3.07	0.00	0.02
100286631	ube2t	ubiquitin conjugating enzyme E2 T	85.41	-1.61	-3.06	0.00	0.00
106576293	LOC106576293	small nuclear ribonucleoprotein F-like	79.55	-1.61	-3.06	0.01	0.05
106609503	LOC106609503	forkhead box protein M1-like	20.70	-1.61	-3.05	0.00	0.00
106587338	ccnb2	cyclin B2	71.56	-1.60	-3.04	0.00	0.00
100195917	ppcs	phosphopantothenoylcysteine synthetase	99.50	-1.60	-3.03	0.00	0.00
106563301	LOC106563301	zinc finger MYND domain-containing protein 19	49.22	-1.60	-3.03	0.00	0.00
106603038	LOC106603038	kinesin-like protein KIF20A	78.13	-1.59	-3.02	0.00	0.00
106571470	LOC106571470	uncharacterized LOC106571470	88.06	-1.59	-3.02	0.00	0.02
106571726	LOC106571726	alpha-(1,3)-fucosyltransferase 9-like	65.80	-1.59	-3.02	0.00	0.00
106563463	LOC106563463	sororin-B-like	64.41	-1.59	-3.01	0.00	0.00
106588679	LOC106588679	actin-binding protein anillin-like	50.19	-1.59	-3.01	0.00	0.00
106603375	ercc6l	ERCC excision repair 6 like, spindle assembly ch	70.42	-1.59	-3.00	0.00	0.00
106562296	LOC106562296	centromere protein N-B-like	16.91	-1.58	-2.99	0.00	0.00
106604942	spdl1	NA	57.43	-1.57	-2.98	0.00	0.01
106560548	LOC106560548	abnormal spindle-like microcephaly-associated	144.35	-1.57	-2.97	0.00	0.00
100195170	ccna2	cyclin A2	83.16	-1.57	-2.97	0.00	0.00
106589569	LOC106589569	zinc finger BED domain-containing protein 1-like	10.78	-1.57	-2.97	0.00	0.00
106588407	LOC106588407	carboxy-terminal kinesin 2-like	190.09	-1.57	-2.97	0.01	0.04
100380757	pfkfb3	6-phosphofructo-2-kinase/fructose-2,6-biphosp	29.22	-1.57	-2.96	0.00	0.00
106564655	LOC106564655	transcription elongation factor, mitochondrial-li	23.86	-1.57	-2.96	0.00	0.00
106567171	racgap1	NA	48.55	-1.56	-2.96	0.00	0.00
106611545	LOC106611545	cyclin-A2-like	105.40	-1.56	-2.95	0.00	0.00
106589124	LOC106589124	tribbles homolog 1-like	18.90	-1.56	-2.94	0.00	0.02
106599551	LOC106599551	period circadian protein homolog 2-like	20.79	-1.56	-2.94	0.00	0.01
106610734	LOC106610734	phosphatidate phosphatase LPIN1-like	95.58	-1.54	-2.91	0.00	0.02
106603271	LOC106603271	acetyl-CoA carboxylase	8215.43	-1.54	-2.91	0.00	0.00
106584447	LOC106584447	protein QIL1-like	277.69	-1.54	-2.91	0.00	0.00
106579188	LOC106579188	SPRY domain-containing SOCS box protein 3-like	6.71	-1.54	-2.90	0.00	0.01
106560846	LOC106560846	leucine-rich repeat-containing protein 20-like	17.02	-1.53	-2.90	0.00	0.00
106568166	LOC106568166	calcyphosin-like protein	5.82	-1.53	-2.90	0.01	0.05
106565839	slc25a26	solute carrier family 25 member 26	178.69	-1.53	-2.90	0.00	0.00
100306857	inceb	Inner centromere protein B	317.08	-1.53	-2.90	0.00	0.03
106605866	LOC106605866	zinc finger protein 664-like	29.68	-1.53	-2.89	0.00	0.00
106590033	LOC106590033	ATP synthase F(0) complex subunit C3, mitocho	1011.61	-1.53	-2.89	0.00	0.04
100286727	ska1	spindle and kinetochore associated complex sul	34.10	-1.53	-2.88	0.01	0.04

106572230	LOC106572230	targeting protein for Xklp2-A-like	124.60	-1.52	-2.88	0.00	0.00
106605545	LOC106605545	xylose isomerase-like	17.28	-1.52	-2.87	0.00	0.01
106570296	LOC106570296	actin-binding protein anillin-like	45.98	-1.52	-2.87	0.00	0.01
106581789	spc25	NA	38.34	-1.52	-2.86	0.00	0.00
106580450	LOC106580450	uncharacterized LOC106580450	97.19	-1.51	-2.85	0.00	0.01
106567942	LOC106567942	cytoskeleton-associated protein 2-like	250.88	-1.51	-2.85	0.00	0.04
106589369	cep55	centrosomal protein 55	27.69	-1.51	-2.85	0.00	0.00
100195599	ube2c	ubiquitin-conjugating enzyme E2C	135.13	-1.50	-2.83	0.00	0.00
106610271	LOC106610271	fatty acid synthase-like	2826.74	-1.50	-2.83	0.00	0.00
106560912	LOC106560912	actin-binding protein anillin-like	112.47	-1.50	-2.82	0.00	0.01
106577983	LOC106577983	alpha-crystallin B chain-like	7.65	-1.49	-2.82	0.00	0.00
106598937	LOC106598937	cytochrome b-c1 complex subunit 6, mitochonc	178.94	-1.49	-2.82	0.01	0.04
106612394	dna2	DNA replication helicase/nuclease 2	57.93	-1.49	-2.81	0.00	0.02
106604765	LOC106604765	tubulin beta-4B chain-like	1219.13	-1.49	-2.81	0.00	0.00
106574437	LOC106574437	glycerol-3-phosphate dehydrogenase 1-like pro	970.85	-1.49	-2.81	0.00	0.00
106568170	LOC106568170	S-phase kinase-associated protein 2-like	15.07	-1.49	-2.81	0.00	0.00
100286532	cenph	centromere protein H	28.52	-1.49	-2.80	0.00	0.00
106563510	LOC106563510	chromosome-associated kinesin KIF4-like	102.83	-1.49	-2.80	0.00	0.01
106579956	pptc7	NA	231.31	-1.48	-2.80	0.00	0.02
106580744	LOC106580744	uncharacterized LOC106580744	6.60	-1.48	-2.80	0.01	0.05
106585273	setd8	lysine methyltransferase 5A	93.48	-1.48	-2.79	0.00	0.00
106561782	LOC106561782	protein regulator of cytokinesis 1-like	30.50	-1.48	-2.79	0.00	0.01
106608242	LOC106608242	coiled-coil domain-containing protein 85C-A-lik	32.87	-1.48	-2.79	0.00	0.00
106582108	LOC106582108	mitotic checkpoint serine/threonine-protein kir	34.49	-1.48	-2.79	0.00	0.00
106569458	LOC106569458	ephrin type-A receptor 4-like	11.07	-1.48	-2.78	0.00	0.00
100194750	cdc20	cell division cycle 20	165.61	-1.48	-2.78	0.00	0.00
106610844	LOC106610844	rho GTPase-activating protein 11A-like	54.57	-1.47	-2.78	0.00	0.01
106586378	LOC106586378	uncharacterized LOC106586378	21.21	-1.47	-2.77	0.00	0.00
100286676	cenpm	Centromere protein M	30.88	-1.47	-2.77	0.00	0.00
106562381	LOC106562381	arylamine N-acetyltransferase, pineal gland isoz	13.95	-1.47	-2.77	0.00	0.00
106584535	LOC106584535	E3 ubiquitin-protein ligase RFWD3-like	24.41	-1.47	-2.77	0.00	0.00
106611889	LOC106611889	uncharacterized LOC106611889	5.40	-1.47	-2.77	0.00	0.01
100286580	hs020	HSPC020 homolog	29.78	-1.47	-2.76	0.00	0.00
106578758	LOC106578758	peptidyl-prolyl cis-trans isomerase A-like	621.74	-1.47	-2.76	0.00	0.00
106607864	LOC106607864	kinesin-like protein KIF11	87.23	-1.47	-2.76	0.00	0.02
106562705	LOC106562705	stAR-related lipid transfer protein 5-like	38.69	-1.46	-2.75	0.00	0.03
106606416	LOC106606416	cyclin-F-like	24.31	-1.46	-2.75	0.00	0.00
106566486	LOC106566486	extracellular sulfatase Sulf-2-like	90.38	-1.46	-2.75	0.00	0.00
106608956	LOC106608956	sentrin-specific protease 3-like	41.48	-1.46	-2.74	0.00	0.00
106610336	papss1	3'-phosphoadenosine 5'-phosphosulfate syntha	47.65	-1.45	-2.74	0.00	0.00

106567765	LOC106567765	nuclear fragile X mental retardation-interacting	149.94	-1.45	-2.74	0.00	0.00
106579876	cenpe	centromere protein E	164.31	-1.45	-2.73	0.00	0.02
106588889	LOC106588889	shugoshin-like 1	18.49	-1.45	-2.73	0.00	0.00
106586099	LOC106586099	GTPase IMAP family member 8-like	23.57	-1.44	-2.72	0.00	0.00
100380551	ifrd2	interferon related developmental regulator 2	224.81	-1.44	-2.72	0.00	0.03
100196670	atp5g3	ATP synthase, H+ transporting, mitochondrial F(	516.48	-1.44	-2.71	0.00	0.01
106584298	LOC106584298	polymeric immunoglobulin receptor-like	17.83	-1.43	-2.70	0.00	0.01
106563511	LOC106563511	protein regulator of cytokinesis 1-like	86.27	-1.43	-2.70	0.00	0.03
106566651	LOC106566651	embryonic polyadenylate-binding protein-like	73.73	-1.43	-2.70	0.00	0.00
106604960	cfap46	cilia and flagella associated protein 46	12.74	-1.43	-2.70	0.00	0.01
106589699	LOC106589699	ras-related protein Rab-3D-like	13.65	-1.43	-2.70	0.00	0.02
106602330	LOC106602330	uncharacterized LOC106602330	27.29	-1.43	-2.69	0.00	0.00
100196476	apitd1	apoptosis-inducing, TAF9-like domain 1	61.02	-1.43	-2.69	0.00	0.00
106588345	LOC106588345	CDC42 small effector protein 1-like	74.10	-1.43	-2.69	0.00	0.02
106610569	melk	maternal embryonic leucine zipper kinase	7.87	-1.42	-2.68	0.00	0.01
100194593	nt5c3	5'-nucleotidase, cytosolic III	3306.27	-1.42	-2.68	0.00	0.00
106609447	LOC106609447	tyrosine 3-monooxygenase-like	10.52	-1.42	-2.68	0.01	0.04
106570829	hmgcr	3-hydroxy-3-methylglutaryl-CoA reductase	2184.45	-1.42	-2.68	0.00	0.00
106582467	LOC106582467	cytochrome c	714.44	-1.42	-2.67	0.00	0.00
106594932	LOC106594932	piggyBac transposable element-derived protein	19.45	-1.41	-2.67	0.00	0.00
106585868	LOC106585868	malate dehydrogenase, cytoplasmic-like	1351.98	-1.41	-2.66	0.00	0.03
100499254	sell	NA	134.90	-1.41	-2.66	0.00	0.01
106576587	LOC106576587	zinc finger protein 420-like	38.80	-1.41	-2.66	0.00	0.00
100196320	rtkn2	rhotekin 2	39.39	-1.41	-2.66	0.00	0.02
100380723	LOC100380723	exportin-1	59.11	-1.41	-2.65	0.00	0.00
106611299	oip5	Opa interacting protein 5	59.74	-1.40	-2.64	0.00	0.00
106573841	LOC106573841	nectin-4-like	135.41	-1.40	-2.64	0.00	0.01
100286659	pmf1	Polyamine-modulated factor 1	61.55	-1.40	-2.64	0.00	0.00
106570342	papd7	poly(A) RNA polymerase D7, non-canonical	25.67	-1.40	-2.63	0.00	0.00
106585451	LOC106585451	calcium-binding mitochondrial carrier protein S(	19.43	-1.40	-2.63	0.00	0.00
106613715	LOC106613715	mitochondrial import inner membrane transloc	67.48	-1.39	-2.63	0.00	0.00
106587023	nudt18	nudix hydrolase 18	38.56	-1.39	-2.63	0.00	0.00
100195680	LOC100195680	transforming acidic coiled-coil-containing prote	249.94	-1.39	-2.62	0.00	0.00
106601894	LOC106601894	jmjC domain-containing protein 8-like	63.39	-1.39	-2.62	0.00	0.00
106563680	LOC106563680	G2/mitotic-specific cyclin-B3-like	21.53	-1.39	-2.61	0.00	0.01
106566439	LOC106566439	NSFL1 cofactor p47-like	207.10	-1.39	-2.61	0.00	0.00
100195132	ima2	Importin subunit alpha-2	309.43	-1.38	-2.61	0.00	0.02
100380819	wee1	WEE1 homolog (S. pombe)	34.87	-1.38	-2.61	0.00	0.02
106584307	LOC106584307	protein ECT2-like	41.23	-1.38	-2.60	0.00	0.03
106607811	LOC106607811	lymphokine-activated killer T-cell-originated pro	76.85	-1.38	-2.60	0.00	0.00

106586766	LOC106586766	CST complex subunit STN1-like	7.99	-1.38	-2.60	0.00	0.00
106587056	LOC106587056	cytoskeleton-associated protein 5-like	104.72	-1.38	-2.60	0.00	0.00
106613710	LOC106613710	mitochondrial import inner membrane transloc	150.97	-1.38	-2.60	0.00	0.00
100306816	ncoa4	nuclear receptor coactivator 4	1088.58	-1.38	-2.60	0.00	0.01
106563621	LOC106563621	kinesin-like protein KIF20A	102.67	-1.38	-2.59	0.00	0.00
100195812	tbb2c	Tubulin beta-2C chain	533.46	-1.37	-2.59	0.00	0.02
106577506	LOC106577506	apolipoprotein Eb-like	524.11	-1.37	-2.59	0.00	0.00
106590205	LOC106590205	uncharacterized LOC106590205	121.96	-1.37	-2.59	0.00	0.01
106573213	LOC106573213	aryl hydrocarbon receptor nuclear translocator-	127.86	-1.37	-2.58	0.01	0.04
106583508	bora	bora, aurora kinase A activator	28.04	-1.37	-2.58	0.00	0.00
106574542	poglut1	NA	16.59	-1.36	-2.57	0.00	0.00
106586637	LOC106586637	lysophosphatidic acid phosphatase type 6-like	64.46	-1.36	-2.57	0.00	0.01
106607838	LOC106607838	uncharacterized LOC106607838	22.59	-1.36	-2.57	0.00	0.01
106564868	LOC106564868	importin subunit alpha-1-like	270.22	-1.36	-2.57	0.00	0.00
106587682	LOC106587682	NAD(P) transhydrogenase, mitochondrial-like	41.62	-1.36	-2.56	0.00	0.00
106612580	LOC106612580	frizzled-9-like	5.15	-1.36	-2.56	0.00	0.00
100195150	tpx2	Targeting protein for Xklp2	233.86	-1.36	-2.56	0.00	0.00
100286508	cetn3	centrin 3	44.67	-1.36	-2.56	0.00	0.03
106602813	frmd7	FERM domain containing 7	9.75	-1.36	-2.56	0.00	0.00
100306807	egln2	Egl nine homolog 2	163.12	-1.35	-2.56	0.00	0.00
106565481	LOC106565481	U3 small nucleolar RNA-interacting protein 2-lik	15.18	-1.35	-2.55	0.00	0.00
100195682	md2l1	Mitotic spindle assembly checkpoint protein M/	65.41	-1.35	-2.55	0.00	0.00
106578092	LOC106578092	uncharacterized LOC106578092	11.79	-1.35	-2.55	0.00	0.00
106612016	LOC106612016	centromere protein U-like	66.98	-1.35	-2.55	0.00	0.03
106588409	LOC106588409	tubulin beta chain	1411.66	-1.35	-2.54	0.00	0.00
106607046	LOC106607046	kinetochore protein Spc24-like	41.16	-1.35	-2.54	0.00	0.00
106588586	LOC106588586	nuclear transport factor 2-like	56.25	-1.34	-2.54	0.01	0.04
106582077	LOC106582077	uncharacterized LOC106582077	6.85	-1.34	-2.54	0.00	0.00
106605152	LOC106605152	dehydrodolichyl diphosphate syntase complex s	61.11	-1.34	-2.53	0.00	0.00
100380718	top2a	NA	481.19	-1.34	-2.53	0.00	0.03
106586169	LOC106586169	polypeptide N-acetylgalactosaminyltransferase	31.22	-1.34	-2.52	0.00	0.00
106576588	LOC106576588	oocyte zinc finger protein XlCOF6-like	208.60	-1.33	-2.52	0.00	0.00
106577137	LOC106577137	kinesin-like protein KIF11-B	110.35	-1.33	-2.52	0.00	0.00
106599121	LOC106599121	uncharacterized LOC106599121	13.91	-1.33	-2.52	0.00	0.00
106606342	LOC106606342	elongation factor Tu, mitochondrial-like	21.73	-1.33	-2.52	0.00	0.00
106571471	casc5	kinetochore scaffold 1	83.39	-1.33	-2.52	0.00	0.00
106588901	LOC106588901	putative serine/threonine-protein kinase C05D1	32.48	-1.33	-2.52	0.00	0.00
106607495	LOC106607495	uncharacterized LOC106607495	19.80	-1.33	-2.51	0.00	0.00
106610772	LOC106610772	cytochrome c oxidase subunit 7A2, mitochondri	522.08	-1.33	-2.51	0.00	0.01
106572274	LOC106572274	interferon-related developmental regulator 2-li	291.45	-1.33	-2.51	0.00	0.01

106603543	LOC106603543	cytoskeleton-associated protein 2-like	89.36	-1.32	-2.50	0.00	0.00
106585276	LOC106585276	cyclin-dependent kinase 2-associated protein 1-	125.04	-1.32	-2.50	0.01	0.05
106579221	LOC106579221	uncharacterized LOC106579221	21.99	-1.32	-2.50	0.00	0.01
106588009	LOC106588009	calcium and integrin-binding family member 2-l	10.54	-1.32	-2.49	0.00	0.00
106583326	trib3	NA	71.49	-1.32	-2.49	0.00	0.00
106602585	LOC106602585	pre-mRNA-processing factor 19-like	27.37	-1.31	-2.49	0.00	0.00
106568532	LOC106568532	asparagine--tRNA ligase, cytoplasmic-like	196.79	-1.31	-2.49	0.00	0.01
106611793	LOC106611793	signal-transducing adaptor protein 1-like	13.53	-1.31	-2.49	0.00	0.00
106568366	LOC106568366	uncharacterized LOC106568366	1288.49	-1.31	-2.49	0.00	0.00
106565446	LOC106565446	25-hydroxyvitamin D-1 alpha hydroxylase, mito	41.48	-1.31	-2.49	0.00	0.01
106605450	LOC106605450	ras GTPase-activating-like protein IQGAP3	28.25	-1.31	-2.49	0.00	0.00
106606306	LOC106606306	nuclear distribution protein nudE-like 1-B	58.93	-1.31	-2.48	0.00	0.00
106603166	LOC106603166	chromosome-associated kinesin KIF4-like	59.14	-1.31	-2.48	0.01	0.05
106565809	LOC106565809	mitochondrial intermembrane space import and	36.89	-1.31	-2.48	0.00	0.00
106562111	LOC106562111	lymphatic vessel endothelial hyaluronic acid rec	11.45	-1.31	-2.47	0.00	0.00
106586580	LOC106586580	uncharacterized LOC106586580	10.14	-1.31	-2.47	0.00	0.03
106571422	nsl1	NSL1, MIS12 kinetochore complex component	54.21	-1.31	-2.47	0.00	0.02
106609453	LOC106609453	RAD51-associated protein 1-like	28.22	-1.30	-2.47	0.00	0.00
106566779	LOC106566779	monoglyceride lipase-like	148.35	-1.30	-2.47	0.00	0.00
106563318	ccnb1	cyclin B1	184.60	-1.30	-2.47	0.00	0.00
106562005	shcbp1	NA	93.78	-1.30	-2.47	0.00	0.00
100195237	gfpt1	glutamine-fructose-6-phosphate transaminase :	367.38	-1.30	-2.46	0.00	0.00
100194810	npl	N-acetylneuraminate pyruvate lyase	139.61	-1.30	-2.46	0.00	0.00
106611716	LOC106611716	centromere protein I-like	45.54	-1.30	-2.46	0.00	0.00
106566558	top1	NA	106.97	-1.30	-2.46	0.00	0.00
106562710	LOC106562710	very-long-chain 3-oxoacyl-CoA reductase-B-like	536.29	-1.30	-2.46	0.00	0.03
100195775	hce1	High choriolytic enzyme 1	20.56	-1.30	-2.46	0.00	0.00
100380717	LOC100380717	heat shock 70 kDa protein 4	83.62	-1.30	-2.46	0.00	0.00
106591329	LOC106591329	aspartate aminotransferase, cytoplasmic-like	66.47	-1.30	-2.46	0.00	0.00
106574630	LOC106574630	lipoyltransferase 1, mitochondrial-like	9.23	-1.30	-2.46	0.00	0.02
100196160	dck	deoxycytidine kinase	101.74	-1.30	-2.46	0.01	0.04
106578378	LOC106578378	lipocalin-like	14523.99	-1.30	-2.45	0.00	0.00
106585098	LOC106585098	cell division cycle-associated protein 2-like	45.68	-1.29	-2.45	0.00	0.00
106568761	LOC106568761	UMP-CMP kinase	483.46	-1.29	-2.45	0.00	0.03
106566185	LOC106566185	coiled-coil domain-containing protein 51-like	19.30	-1.29	-2.45	0.00	0.00
106577153	LOC106577153	putative ATP-dependent RNA helicase DHX57	33.26	-1.29	-2.45	0.01	0.04
106562504	LOC106562504	NAD(P) transhydrogenase, mitochondrial-like	51.87	-1.29	-2.45	0.00	0.01
106584498	LOC106584498	sodium- and chloride-dependent GABA transpo	151.17	-1.29	-2.44	0.00	0.00
100194600	bhmt	betaine-homocysteine methyltransferase	1598.15	-1.28	-2.44	0.00	0.00
106565678	espl1	extra spindle pole bodies like 1, separase	54.60	-1.28	-2.44	0.00	0.00

106571947	LOC106571947	glucose-6-phosphate 1-dehydrogenase-like	536.02	-1.28	-2.44	0.00	0.02
106609043	LOC106609043	DEP domain-containing protein 7-like	31.22	-1.28	-2.44	0.00	0.00
100194626	atp5g3	ATP synthase, H+ transporting, mitochondrial F(	1850.15	-1.28	-2.43	0.00	0.00
106580858	LOC106580858	WD repeat-containing protein 20-like	19.91	-1.28	-2.43	0.00	0.01
106609479	LOC106609479	small nuclear ribonucleoprotein F	62.77	-1.28	-2.43	0.01	0.04
106610763	rtn4ip1	NA	70.78	-1.28	-2.43	0.00	0.00
106584598	gtse1	G2 and S-phase expressed 1	53.26	-1.27	-2.42	0.00	0.00
100196609	stk6	Serine/threonine-protein kinase 6	92.99	-1.27	-2.42	0.00	0.00
106605338	LOC106605338	sterol regulatory element-binding protein cleav	515.01	-1.27	-2.42	0.00	0.00
106561512	LOC106561512	transmembrane protein 168-like	38.05	-1.27	-2.41	0.00	0.00
106613897	LOC106613897	cyclin-dependent kinase 4 inhibitor C-like	9.22	-1.27	-2.41	0.00	0.01
106573925	LOC106573925	beta-chimaerin-like	9.82	-1.27	-2.41	0.00	0.00
106603085	LOC106603085	type II iodothyronine deiodinase-like	297.39	-1.27	-2.41	0.00	0.00
106603145	LOC106603145	peroxiredoxin-5, mitochondrial-like	182.70	-1.27	-2.41	0.00	0.00
100196702	g6pd	glucose-6-phosphate dehydrogenase	883.73	-1.27	-2.41	0.00	0.00
100196629	serb	Phosphoserine phosphatase	200.29	-1.27	-2.41	0.00	0.00
100195529	prc1	regulator of cytokinesis 1	67.60	-1.27	-2.40	0.00	0.00
100380681	LOC100380681	tubulin alpha-1C chain	644.89	-1.26	-2.40	0.00	0.00
106562156	LOC106562156	methylmalonyl-CoA epimerase, mitochondrial-l	33.74	-1.26	-2.40	0.00	0.00
106577388	LOC106577388	malate dehydrogenase, cytoplasmic-like	376.51	-1.26	-2.40	0.00	0.00
100380465	cor1c	Coronin-1C	72.72	-1.26	-2.40	0.00	0.00
106567204	LOC106567204	tubulin alpha-8 chain-like	110.54	-1.26	-2.40	0.00	0.00
100195113	ehd3	EH-domain containing 3	160.84	-1.26	-2.40	0.00	0.00
106604510	LOC106604510	NAD-dependent protein deacetylase sirtuin-1-li	19.67	-1.26	-2.40	0.00	0.00
106602126	LOC106602126	structural maintenance of chromosomes protei	47.20	-1.26	-2.40	0.00	0.01
106570182	dnlz	DNL-type zinc finger	5.75	-1.26	-2.39	0.00	0.00
106603037	LOC106603037	bromodomain-containing protein 8-like	14.20	-1.26	-2.39	0.00	0.00
100194862	pgm3	phosphoglucomutase 3	329.44	-1.26	-2.39	0.00	0.02
106565657	LOC106565657	cyclin-dependent kinase inhibitor 1-like	11.04	-1.26	-2.39	0.00	0.01
106609884	LOC106609884	tropomodulin-1-like	34.49	-1.25	-2.39	0.00	0.00
106567696	LOC106567696	proline-rich protein 11-like	18.96	-1.25	-2.39	0.00	0.00
106571601	LOC106571601	type III iodothyronine deiodinase-like	300.80	-1.25	-2.39	0.00	0.00
106577215	ppa1	NA	1150.32	-1.25	-2.38	0.00	0.00
106582973	LOC106582973	draxin-B	6.96	-1.25	-2.38	0.00	0.02
106601796	g2e3	G2/M-phase specific E3 ubiquitin protein ligase	23.63	-1.25	-2.38	0.00	0.00
106565020	LOC106565020	guanine nucleotide-binding protein G(z) subuni	58.89	-1.25	-2.38	0.00	0.00
106579107	LOC106579107	1-phosphatidylinositol 4,5-bisphosphate phospl	6.29	-1.25	-2.38	0.00	0.00
106580945	LOC106580945	multiple epidermal growth factor-like domains	8.84	-1.25	-2.37	0.00	0.00
106602347	LOC106602347	SWI/SNF-related matrix-associated actin-depen	40.97	-1.24	-2.37	0.00	0.00
106582498	LOC106582498	nucleoporin NUP53-like	62.98	-1.24	-2.37	0.00	0.00

100195559	pgd	phosphogluconate dehydrogenase	2133.32	-1.24	-2.37	0.00	0.03
100194586	hsp10	heat shock protein 10	694.78	-1.24	-2.36	0.00	0.02
106576723	apmap	adipocyte plasma membrane associated protein	2108.56	-1.24	-2.36	0.00	0.00
106562286	LOC106562286	cytochrome c oxidase subunit 4 isoform 1, mito	484.78	-1.24	-2.36	0.00	0.00
106589469	LOC106589469	uncharacterized LOC106589469	26.28	-1.24	-2.36	0.00	0.00
106573900	LOC106573900	phosphatidylinositol 3-kinase regulatory subuni	135.59	-1.23	-2.35	0.00	0.00
106602328	LOC106602328	coronin-2A-like	115.47	-1.23	-2.35	0.00	0.03
100194920	acat2	acetyl-CoA acetyltransferase 2	1632.84	-1.23	-2.35	0.00	0.01
106611357	cenpf	centromere protein F	107.80	-1.23	-2.35	0.00	0.00
106569884	LOC106569884	interleukin enhancer-binding factor 2 homolog	315.07	-1.23	-2.35	0.00	0.02
106571961	atp5e	ATP synthase, H+ transporting, mitochondrial F	1119.36	-1.23	-2.35	0.00	0.00
106585173	LOC106585173	BAG family molecular chaperone regulator 4-lik	59.55	-1.23	-2.34	0.00	0.00
106588685	rpa3	replication protein A3	214.10	-1.23	-2.34	0.00	0.00
106600827	LOC106600827	uncharacterized LOC106600827	34.23	-1.23	-2.34	0.01	0.04
106609774	dnajc2	DnaJ heat shock protein family (Hsp40) membe	96.92	-1.23	-2.34	0.00	0.00
106565373	LOC106565373	citron Rho-interacting kinase-like	51.80	-1.23	-2.34	0.00	0.00
106562833	LOC106562833	outer dense fiber protein 2-like	15.63	-1.22	-2.34	0.00	0.01
106576759	LOC106576759	alpha-2,8-sialyltransferase 8E-like	125.02	-1.22	-2.34	0.00	0.02
106612704	LOC106612704	SERTA domain-containing protein 3-like	26.74	-1.22	-2.34	0.00	0.02
106589802	fam195a	MAPK regulated corepressor interacting protein	352.12	-1.22	-2.34	0.00	0.00
106585119	LOC106585119	condensin complex subunit 2-like	65.12	-1.22	-2.33	0.00	0.01
100196145	pgp	phosphoglycolate phosphatase	240.60	-1.22	-2.33	0.00	0.00
100195487	enosf1	enolase superfamily member 1	87.93	-1.22	-2.33	0.00	0.02
106589036	LOC106589036	mpv17-like protein	164.26	-1.22	-2.33	0.00	0.00
106560578	LOC106560578	prohibitin-2-like	767.57	-1.22	-2.33	0.00	0.00
106613010	LOC106613010	phosphatidylinositol-binding clathrin assembly	46.75	-1.22	-2.33	0.00	0.00
106567641	LOC106567641	heat shock cognate 70 kDa protein-like	114.06	-1.22	-2.33	0.00	0.00
106612247	LOC106612247	leucine-rich repeat-containing protein 59-like	241.18	-1.22	-2.33	0.00	0.02
106607146	LOC106607146	transmembrane protein 184B-like	12.86	-1.22	-2.33	0.00	0.00
106584422	fam46b	family with sequence similarity 46 member B	68.72	-1.22	-2.33	0.00	0.00
106583199	LOC106583199	protein transport protein Sec61 subunit alpha is	449.80	-1.22	-2.32	0.00	0.00
106571687	LOC106571687	uncharacterized LOC106571687	12.90	-1.22	-2.32	0.00	0.01
106606992	LOC106606992	envoplakin-like	8.46	-1.22	-2.32	0.00	0.00
106565437	LOC106565437	cyclin-dependent kinase 2-like	37.77	-1.21	-2.32	0.00	0.00
106568158	LOC106568158	heparanase-like	21.75	-1.21	-2.32	0.00	0.00
100136483	LOC100136483	tubulin beta-1 chain	1464.24	-1.21	-2.32	0.00	0.00
106588385	LOC106588385	histone H3-like centromeric protein A	57.10	-1.21	-2.32	0.00	0.01
106602730	map9	microtubule associated protein 9	15.44	-1.21	-2.32	0.00	0.00
100195337	tpd52l1	tumor protein D52-like 1	426.21	-1.21	-2.31	0.00	0.01
106590647	LOC106590647	ATP-binding cassette sub-family D member 3	849.62	-1.21	-2.31	0.00	0.00

106567863	LOC106567863	ribonucleoside-diphosphate reductase large sul	165.59	-1.21	-2.31	0.00	0.00
106578798	cssa19h6orf118	chromosome ssa19 open reading frame, humar	21.90	-1.21	-2.31	0.00	0.00
106560276	LOC106560276	carbohydrate sulfotransferase 14-like	9.76	-1.21	-2.31	0.00	0.00
100136477	LOC100136477	peroxisome proliferator-activated receptor gan	130.35	-1.21	-2.31	0.00	0.00
106561666	LOC106561666	neuroepithelial cell-transforming gene 1 protei	46.82	-1.21	-2.31	0.00	0.00
106563197	fam136a	family with sequence similarity 136 member A	205.07	-1.21	-2.31	0.00	0.00
106610270	LOC106610270	sperm-associated antigen 5-like	19.90	-1.20	-2.31	0.00	0.00
106586371	LOC106586371	coenzyme Q-binding protein COQ10 homolog B	68.36	-1.20	-2.30	0.00	0.01
100380815	sephs1	selenophosphate synthetase 1	295.18	-1.20	-2.30	0.00	0.01
106580925	LOC106580925	uncharacterized LOC106580925	6.88	-1.20	-2.30	0.00	0.00
100195726	gsta3	glutathione S-transferase alpha 3	704.12	-1.20	-2.30	0.00	0.00
106579978	LOC106579978	multiple epidermal growth factor-like domains	20.96	-1.20	-2.30	0.00	0.00
106583862	mkrn2os	MKRN2 opposite strand	33.77	-1.20	-2.30	0.00	0.00
106612990	LOC106612990	high mobility group nucleosome-binding domai	607.91	-1.20	-2.30	0.00	0.02
106602584	LOC106602584	V-type proton ATPase 116 kDa subunit a isofori	14.71	-1.20	-2.29	0.00	0.00
106609975	LOC106609975	early growth response protein 2b-like	7.99	-1.20	-2.29	0.00	0.00
100196498	tmem126a	transmembrane protein 126A	112.68	-1.20	-2.29	0.01	0.04
106562484	dut	deoxyuridine triphosphatase	199.58	-1.20	-2.29	0.00	0.03
106606750	LOC106606750	ER lumen protein-retaining receptor 2-like	222.96	-1.19	-2.29	0.00	0.01
106603131	LOC106603131	sororin-like	23.72	-1.19	-2.29	0.00	0.00
106602590	xpa	NA	28.87	-1.19	-2.29	0.00	0.00
106568941	LOC106568941	mitochondrial import inner membrane transloc	12.07	-1.19	-2.29	0.00	0.00
106564497	LOC106564497	low-density lipoprotein receptor-like	112.57	-1.19	-2.29	0.00	0.00
106572219	LOC106572219	coiled-coil domain-containing protein 22-like	112.70	-1.19	-2.28	0.00	0.00
106613551	LOC106613551	cytochrome b-c1 complex subunit 6, mitochonc	235.86	-1.19	-2.28	0.00	0.00
106564257	LOC106564257	proline dehydrogenase 1, mitochondrial-like	31.41	-1.19	-2.28	0.01	0.04
106584724	LOC106584724	centrosomal protein of 152 kDa-like	16.28	-1.19	-2.28	0.00	0.00
106584374	LOC106584374	long-chain-fatty-acid--CoA ligase ACSBG2-like	63.38	-1.19	-2.28	0.01	0.04
100196617	dh12b	Estradiol 17-beta-dehydrogenase 12-B	2147.54	-1.19	-2.28	0.00	0.01
101448017	sp30l	Histone deacetylase complex subunit SAP30L	48.54	-1.18	-2.27	0.00	0.00
106573068	LOC106573068	vesicle transport protein GOT1B	162.51	-1.18	-2.27	0.00	0.01
106565357	LOC106565357	mitochondrial import receptor subunit TOM6 h	455.92	-1.18	-2.27	0.00	0.00
106563520	LOC106563520	kinesin-like protein KIF18A	12.83	-1.18	-2.27	0.00	0.00
106566758	LOC106566758	cat eye syndrome critical region protein 5-like	14.88	-1.18	-2.27	0.00	0.03
106588330	LOC106588330	glycosylated lysosomal membrane protein-like	13.32	-1.18	-2.26	0.00	0.00
106577387	LOC106577387	malate dehydrogenase, cytoplasmic-like	95.98	-1.18	-2.26	0.00	0.00
106584709	LOC106584709	cytochrome c oxidase subunit 5A, mitochondria	615.64	-1.17	-2.26	0.00	0.00
106612053	hmmr	hyaluronan mediated motility receptor	74.97	-1.17	-2.26	0.00	0.00
106569070	LOC106569070	centromere protein L-like	22.86	-1.17	-2.25	0.00	0.02
106610468	mis18bp1	MIS18 binding protein 1	37.91	-1.17	-2.25	0.00	0.00

106564539	LOC106564539	serine hydrolase-like protein	49.51	-1.17	-2.25	0.00	0.00
106583156	LOC106583156	serine/threonine-protein kinase 40	115.66	-1.17	-2.25	0.00	0.03
106580001	LOC106580001	tubulin beta-1 chain	1566.46	-1.17	-2.25	0.00	0.00
106570899	LOC106570899	progestin and adipoQ receptor family member :	12.21	-1.17	-2.25	0.00	0.00
106568460	LOC106568460	zinc finger protein 462-like	9.06	-1.17	-2.25	0.00	0.00
100196409	cks1	Cyclin-dependent kinases regulatory subunit 1	33.36	-1.17	-2.25	0.00	0.00
106588646	LOC106588646	stathmin-like	160.11	-1.17	-2.24	0.00	0.01
100196714	stml2	Stomatin-like protein 2	150.31	-1.17	-2.24	0.00	0.00
106582509	LOC106582509	tubulin alpha chain-like	31.89	-1.17	-2.24	0.00	0.01
106569038	LOC106569038	myb-related protein A-like	64.70	-1.17	-2.24	0.00	0.00
100195137	prim2	primase (DNA) subunit 2	107.97	-1.16	-2.24	0.00	0.02
106583918	LOC106583918	ras-related protein R-Ras-like	57.93	-1.16	-2.24	0.00	0.00
106602876	LOC106602876	ATP synthase subunit g, mitochondrial-like	619.77	-1.16	-2.24	0.00	0.00
106604799	nanp	N-acetylneuraminic acid phosphatase	20.69	-1.16	-2.24	0.00	0.00
100380449	stk35	serine/threonine kinase 35	41.61	-1.16	-2.24	0.00	0.00
100196419	kith	Thymidine kinase, cytosolic	80.00	-1.16	-2.23	0.00	0.00
106571922	LOC106571922	proteasome subunit alpha type-7-like	289.89	-1.16	-2.23	0.00	0.00
106607869	LOC106607869	trichohyalin-like	1684.61	-1.16	-2.23	0.00	0.00
106562699	LOC106562699	kinesin-like protein KIF23	15.18	-1.16	-2.23	0.00	0.02
106599488	LOC106599488	presequence protease, mitochondrial-like	280.98	-1.16	-2.23	0.00	0.03
106569234	LOC106569234	protein canopy-1-like	15.89	-1.16	-2.23	0.00	0.00
100196567	dyl1	Dynein light chain 1, cytoplasmic	530.43	-1.15	-2.22	0.00	0.00
106584900	LOC106584900	retinol dehydrogenase 14-like	123.54	-1.15	-2.22	0.00	0.03
106563496	LOC106563496	peptidyl-prolyl cis-trans isomerase FKBP2-like	305.03	-1.15	-2.22	0.00	0.00
106581779	ppig	NA	243.12	-1.15	-2.22	0.00	0.00
106564654	LOC106564654	ATPase family AAA domain-containing protein 5	10.02	-1.15	-2.22	0.00	0.00
106603147	LOC106603147	barrier-to-autointegration factor	434.96	-1.15	-2.22	0.00	0.02
106577211	LOC106577211	DNA damage-inducible transcript 4 protein-like	114.82	-1.15	-2.22	0.00	0.00
106576306	LOC106576306	condensin complex subunit 1-like	16.07	-1.15	-2.22	0.00	0.00
106575911	LOC106575911	growth hormone-regulated TBC protein 1-A-like	39.58	-1.15	-2.22	0.00	0.03
106565623	LOC106565623	ethanolamine kinase 1-like	140.78	-1.15	-2.22	0.00	0.00
106571077	LOC106571077	dolichyl-diphosphooligosaccharide--protein glyc	290.79	-1.15	-2.21	0.00	0.00
106610322	LOC106610322	NADH dehydrogenase [ubiquinone] 1 beta subc	58.80	-1.15	-2.21	0.01	0.05
106611781	LOC106611781	nocturnin-like	13.74	-1.15	-2.21	0.00	0.02
100196832	usmg5	Up-regulated during skeletal muscle growth prc	420.57	-1.14	-2.21	0.00	0.00
106573477	pex11a	peroxisomal biogenesis factor 11 alpha	75.61	-1.14	-2.21	0.00	0.01
106576731	dnph1	2'-deoxynucleoside 5'-phosphate N-hydrolase 1	39.48	-1.14	-2.21	0.00	0.03
106569347	LOC106569347	ATP-binding cassette sub-family B member 8, m	131.88	-1.14	-2.20	0.00	0.00
100196214	tyms	thymidylate synthetase	163.13	-1.14	-2.20	0.00	0.02
106604162	LOC106604162	DNA-directed RNA polymerase II subunit RPB7-l	25.07	-1.14	-2.20	0.00	0.00

106603918	LOC106603918	deoxycytidine kinase-like	53.52	-1.14	-2.20	0.00	0.00
106587253	LOC106587253	putative solute carrier family 22 member 31	32.07	-1.14	-2.20	0.00	0.00
106563557	LOC106563557	uncharacterized LOC106563557	1201.36	-1.14	-2.20	0.00	0.00
106612607	LOC106612607	heterogeneous nuclear ribonucleoprotein L-like	296.62	-1.14	-2.20	0.00	0.00
100286615	phs	Pterin-4-alpha-carbinolamine dehydratase	1244.50	-1.14	-2.20	0.00	0.03
106603098	LOC106603098	proteasome subunit beta type-6-like	99.42	-1.14	-2.20	0.00	0.00
106613229	numa1	nuclear mitotic apparatus protein 1	79.02	-1.14	-2.20	0.00	0.00
106607333	LOC106607333	rRNA methyltransferase 2, mitochondrial-like	22.90	-1.14	-2.20	0.00	0.00
106576419	LOC106576419	forkhead box protein M1-like	27.84	-1.14	-2.20	0.00	0.00
106580452	LOC106580452	purpurin-like	58.64	-1.13	-2.19	0.00	0.00
106602291	LOC106602291	bifunctional 3'-phosphoadenosine 5'-phosphosu	29.61	-1.13	-2.19	0.00	0.00
106585818	LOC106585818	COMM domain-containing protein 1-like	54.27	-1.13	-2.19	0.00	0.02
106574529	LOC106574529	small ubiquitin-related modifier 3	189.87	-1.13	-2.19	0.00	0.00
100306826	tp4a1	tyrosine phosphatase type IVA 1	87.59	-1.13	-2.19	0.00	0.00
106604250	LOC106604250	uncharacterized LOC106604250	17.03	-1.13	-2.18	0.00	0.00
106575037	mrpl16	mitochondrial ribosomal protein L16	372.31	-1.13	-2.18	0.00	0.01
106607219	LOC106607219	ribonucleoprotein PTB-binding 1-like	55.39	-1.12	-2.18	0.00	0.00
100196438	tba1a	Tubulin alpha-1A chain	262.58	-1.12	-2.18	0.00	0.00
106587076	LOC106587076	transcription factor E2F8-like	21.86	-1.12	-2.18	0.00	0.00
106583034	LOC106583034	monocarboxylate transporter 2-like	55.03	-1.12	-2.17	0.00	0.03
100196112	cdkn3	cyclin dependent kinase inhibitor 3	54.18	-1.12	-2.17	0.00	0.02
106601018	LOC106601018	heat shock 70 kDa protein-like	14.86	-1.12	-2.17	0.00	0.03
106565458	LOC106565458	coatomer subunit zeta-1	34.16	-1.12	-2.17	0.00	0.00
100196541	crj1a	Crystallin J1A	245.39	-1.12	-2.17	0.00	0.00
106587101	ndufs3	NADH:ubiquinone oxidoreductase core subunit	452.15	-1.12	-2.17	0.00	0.02
106607888	LOC106607888	DNA repair protein RAD51 homolog 1	33.95	-1.12	-2.17	0.00	0.00
106602580	LOC106602580	NADH dehydrogenase [ubiquinone] iron-sulfur	504.39	-1.12	-2.17	0.00	0.00
106566598	LOC106566598	40S ribosomal protein S23	181.28	-1.12	-2.17	0.00	0.00
106565174	LOC106565174	hyaluronidase-2-like	179.55	-1.12	-2.17	0.00	0.00
106613700	LOC106613700	tax1-binding protein 3-like	46.13	-1.12	-2.17	0.00	0.00
106576642	LOC106576642	nuclear pore complex protein Nup107-like	13.37	-1.11	-2.16	0.00	0.00
100195267	ccnb3	cyclin B3	128.35	-1.11	-2.16	0.00	0.00
106587404	LOC106587404	aspartate aminotransferase, mitochondrial-like	1060.73	-1.11	-2.16	0.00	0.01
106585487	LOC106585487	cytochrome c oxidase subunit 6A1, mitochondri	216.98	-1.11	-2.16	0.00	0.00
106602346	LOC106602346	SWI/SNF-related matrix-associated actin-depen	47.08	-1.11	-2.16	0.00	0.01
106610823	LOC106610823	uncharacterized LOC106610823	12.80	-1.11	-2.16	0.00	0.00
106562237	LOC106562237	nestin-like	88.55	-1.11	-2.16	0.00	0.01
106578534	LOC106578534	mitochondrial folate transporter/carrier-like	12.99	-1.11	-2.16	0.00	0.01
106608798	LOC106608798	cytidine deaminase-like	172.53	-1.11	-2.16	0.00	0.00
106606510	LOC106606510	heat shock protein 75 kDa, mitochondrial-like	156.38	-1.11	-2.16	0.00	0.00

106580963	LOC106580963	structural maintenance of chromosomes protei	151.80	-1.11	-2.15	0.00	0.00
106610985	kif15	kinesin family member 15	60.85	-1.10	-2.15	0.00	0.00
106574554	LOC106574554	retinol dehydrogenase 7-like	93.23	-1.10	-2.15	0.00	0.00
106573897	LOC106573897	cAMP-specific 3',5'-cyclic phosphodiesterase 4C	57.52	-1.10	-2.15	0.00	0.01
100286655	ybey	ybeY metallopeptidase (putative)	76.89	-1.10	-2.15	0.00	0.02
100196854	prim1	primase (DNA) subunit 1	213.37	-1.10	-2.15	0.00	0.00
106577204	LOC106577204	ribosomal protein 63, mitochondrial-like	102.25	-1.10	-2.15	0.00	0.00
106587628	LOC106587628	guanosine-3',5'-bis(diphosphate) 3'-pyrophosph	45.91	-1.10	-2.14	0.00	0.01
100194628	calm2	calmodulin 2	599.56	-1.10	-2.14	0.00	0.00
106611241	LOC106611241	mitochondrial import inner membrane transloc	15.90	-1.10	-2.14	0.00	0.00
100380324	pp2aa	Serine/threonine-protein phosphatase 2A catal	154.23	-1.10	-2.14	0.00	0.00
106571199	LOC106571199	serine/threonine-protein kinase Nek2-like	27.71	-1.10	-2.14	0.00	0.03
100196000	fen1	flap structure-specific endonuclease 1	520.17	-1.10	-2.14	0.00	0.03
106585288	LOC106585288	B-cell CLL/lymphoma 7 protein family member	18.90	-1.10	-2.14	0.00	0.00
106602874	LOC106602874	histone H2AX	1201.93	-1.10	-2.14	0.01	0.04
106580731	LOC106580731	protein YIF1B-like	15.91	-1.10	-2.14	0.00	0.01
106589992	LOC106589992	peptidyl-prolyl cis-trans isomerase A-like	169.03	-1.09	-2.14	0.00	0.01
106569651	pask	PAS domain containing serine/threonine kinase	26.60	-1.09	-2.13	0.00	0.00
106600481	LOC106600481	zinc finger protein 180-like	119.78	-1.09	-2.13	0.00	0.03
106578532	idi1	isopentenyl-diphosphate delta isomerase 1	2409.40	-1.09	-2.13	0.00	0.00
100306840	ivns1abp	influenza virus NS1A binding protein	347.17	-1.09	-2.13	0.00	0.01
106589940	LOC106589940	kinesin-like protein KIF20B	66.50	-1.09	-2.13	0.00	0.00
106583807	cenpp	centromere protein P	13.29	-1.09	-2.13	0.00	0.00
106582890	LOC106582890	DNA topoisomerase 1-like	105.13	-1.09	-2.12	0.00	0.03
106585552	LOC106585552	protein turtle homolog A-like	17.66	-1.09	-2.12	0.00	0.01
106586921	LOC106586921	tubulin alpha chain-like	171.26	-1.09	-2.12	0.00	0.00
106575920	ncapd2	non-SMC condensin I complex subunit D2	181.05	-1.09	-2.12	0.00	0.01
106594936	LOC106594936	nucleolar and spindle-associated protein 1-like	101.78	-1.09	-2.12	0.00	0.00
106571483	LOC106571483	28S ribosomal protein S18a, mitochondrial-like	80.70	-1.08	-2.12	0.00	0.01
106602179	arid4a	AT-rich interaction domain 4A	57.28	-1.08	-2.12	0.00	0.00
100196141	ap1s3	AP-1 complex subunit sigma-3	25.18	-1.08	-2.12	0.00	0.00
100195736	spry4	sprouty (Drosophila) homolog 4	105.82	-1.08	-2.12	0.00	0.03
100380795	LOC100380795	26S proteasome non-ATPase regulatory subunit	340.04	-1.08	-2.12	0.00	0.00
106589464	cssa28h17orf62	chromosome ssa28 open reading frame, humar	182.28	-1.08	-2.11	0.00	0.00
106574334	LOC106574334	ATP synthase-coupling factor 6, mitochondrial-l	450.00	-1.08	-2.11	0.00	0.02
106601051	LOC106601051	hemoglobin subunit beta-like	666.19	-1.08	-2.11	0.00	0.00
100195168	pura1	Adenylosuccinate synthetase isozyme 1	847.88	-1.08	-2.11	0.00	0.01
106581119	LOC106581119	non-histone chromosomal protein HMG-14-like	884.80	-1.08	-2.11	0.00	0.00
100306758	cf166	CF166 protein	113.39	-1.08	-2.11	0.00	0.00
100380335	arhg4	Rho guanine nucleotide exchange factor 4	25.22	-1.07	-2.11	0.00	0.00

106576790	LOC106576790	zinc finger protein 518A-like	33.62	-1.07	-2.10	0.00	0.00
106603873	LOC106603873	biorientation of chromosomes in cell division pr	74.39	-1.07	-2.10	0.00	0.00
106609195	LOC106609195	NADH dehydrogenase [ubiquinone] 1 alpha sub	144.86	-1.07	-2.10	0.00	0.00
106603673	ncapd3	non-SMC condensin II complex subunit D3	121.89	-1.07	-2.10	0.00	0.00
106588465	LOC106588465	adenylate kinase 2, mitochondrial	624.46	-1.07	-2.10	0.00	0.01
106572650	LOC106572650	sentrin-specific protease 1-like	26.80	-1.07	-2.10	0.00	0.00
100194724	LOC100194724	ATP synthase subunit O, mitochondrial	999.30	-1.07	-2.10	0.00	0.00
100194764	tbb5	Tubulin beta chain	956.05	-1.07	-2.10	0.00	0.00
106571352	LOC106571352	protein LEG1 homolog	7536.11	-1.07	-2.10	0.00	0.00
106579779	LOC106579779	abhydrolase domain-containing protein 16A-like	159.74	-1.07	-2.10	0.00	0.00
106602938	LOC106602938	NADH dehydrogenase [ubiquinone] 1 alpha sub	163.34	-1.07	-2.10	0.00	0.00
100196511	sld5	DNA replication complex GINS protein SLD5	150.31	-1.07	-2.09	0.00	0.00
100380509	prr11	Proline-rich protein 11	46.01	-1.07	-2.09	0.00	0.00
106561084	LOC106561084	G/T mismatch-specific thymine DNA glycosylase	72.97	-1.07	-2.09	0.00	0.00
106609509	LOC106609509	aryl hydrocarbon receptor nuclear translocator-	248.97	-1.07	-2.09	0.00	0.00
106571974	LOC106571974	voltage-dependent L-type calcium channel subu	32.97	-1.07	-2.09	0.00	0.00
106589585	dlgap5	DLG associated protein 5	47.79	-1.07	-2.09	0.00	0.02
106580405	LOC106580405	pyridoxal phosphate phosphatase-like	41.93	-1.06	-2.09	0.00	0.00
106576160	LOC106576160	uncharacterized LOC106576160	26.50	-1.06	-2.09	0.00	0.00
106588058	mrps11	mitochondrial ribosomal protein S11	249.80	-1.06	-2.09	0.00	0.01
106602033	LOC106602033	cofilin-2-like	65.89	-1.06	-2.09	0.00	0.00
106580382	LOC106580382	ubiquitin-conjugating enzyme E2 D4	48.40	-1.06	-2.09	0.00	0.01
106578435	LOC106578435	E3 ubiquitin-protein ligase TRIM58-like	249.97	-1.06	-2.08	0.00	0.00
106608593	LOC106608593	RNA-binding protein 4.1-like	117.21	-1.06	-2.08	0.00	0.00
100195367	trm61	TRM61 protein	63.71	-1.06	-2.08	0.00	0.00
106578977	LOC106578977	centrosomal protein of 55 kDa-like	30.64	-1.06	-2.08	0.00	0.00
106587689	LOC106587689	protein regulator of cytokinesis 1-like	52.94	-1.05	-2.08	0.00	0.00
106606629	LOC106606629	glucose-induced degradation protein 4 homolog	25.68	-1.05	-2.08	0.00	0.00
106613367	opa3	optic atrophy 3 (autosomal recessive, with chor	12.05	-1.05	-2.07	0.00	0.00
106569160	LOC106569160	PH domain leucine-rich repeat-containing prote	45.37	-1.05	-2.07	0.00	0.00
106561409	mterf2	mitochondrial transcription termination factor 2	22.29	-1.05	-2.07	0.00	0.00
106591622	LOC106591622	uncharacterized LOC106591622	20.09	-1.05	-2.07	0.00	0.02
106590555	LOC106590555	protein transport protein Sec61 subunit gamma	20.49	-1.05	-2.07	0.00	0.00
106577505	LOC106577505	apolipoprotein A-IV-like	1676.78	-1.05	-2.07	0.00	0.00
100195454	ct054	CT054 protein	24.92	-1.05	-2.07	0.00	0.01
106567351	LOC106567351	O-acetyl-ADP-ribose deacetylase 1-like	89.92	-1.05	-2.07	0.00	0.00
100194616	cf12	cofilin 2	25.75	-1.05	-2.07	0.00	0.00
106586630	LOC106586630	ATP synthase subunit O, mitochondrial-like	700.41	-1.05	-2.07	0.00	0.03
100196188	btg3	B-cell translocation gene 3	33.76	-1.05	-2.07	0.00	0.00
100380573	typx	Tryparedoxin	28.60	-1.05	-2.07	0.00	0.02

100196204	ppih	peptidylprolyl isomerase H	103.56	-1.05	-2.07	0.00	0.00
100196531	rpp30	ribonuclease P/MRP 30 subunit	54.90	-1.04	-2.06	0.00	0.01
106582879	LOC106582879	uncharacterized LOC106582879	86.53	-1.04	-2.06	0.00	0.00
100306724	pttg1	pituitary tumor-transforming 1	81.89	-1.04	-2.06	0.00	0.00
106613676	LOC106613676	nicotinamide riboside kinase 2-like	93.28	-1.04	-2.06	0.00	0.00
106610331	LOC106610331	integrator complex subunit 12-like	41.34	-1.04	-2.06	0.00	0.00
106612199	LOC106612199	sterol-4-alpha-carboxylate 3-dehydrogenase, de	197.26	-1.04	-2.06	0.00	0.00
100846962	dad1	defender against cell death 1	1176.25	-1.04	-2.06	0.00	0.00
106585457	LOC106585457	prostaglandin E synthase 2-like	276.54	-1.04	-2.06	0.00	0.01
106579885	LOC106579885	transmembrane protein 230-like	122.05	-1.04	-2.05	0.00	0.00
106566721	LOC106566721	prostaglandin E synthase 3-like	707.74	-1.04	-2.05	0.01	0.04
100286684	haus1	HAUS augmin like complex subunit 1	80.07	-1.04	-2.05	0.00	0.01
106586370	LOC106586370	MOB-like protein phocein	738.41	-1.03	-2.05	0.00	0.00
106604175	LOC106604175	solute carrier family 25 member 48-like	248.75	-1.03	-2.05	0.00	0.04
106605279	LOC106605279	centromere protein O-like	39.51	-1.03	-2.05	0.00	0.00
100196004	ssna1	SS nuclear autoantigen 1	16.17	-1.03	-2.04	0.00	0.00
106573338	gdpgp1	GDP-D-glucose phosphorylase 1	30.24	-1.03	-2.04	0.01	0.05
106589769	LOC106589769	MORN repeat-containing protein 4-like	63.91	-1.03	-2.04	0.00	0.00
106611697	LOC106611697	mitochondrial import receptor subunit TOM5 h	87.33	-1.03	-2.04	0.00	0.01
106608168	LOC106608168	26S protease regulatory subunit 10B-like	304.40	-1.03	-2.04	0.00	0.01
106584717	scamp5	NA	21.46	-1.03	-2.04	0.00	0.00
106576202	LOC106576202	ATP synthase subunit gamma, mitochondrial-lik	814.05	-1.03	-2.04	0.00	0.01
106560707	LOC106560707	carnitine O-palmitoyltransferase 1, liver isoform	142.63	-1.03	-2.04	0.00	0.00
106563479	LOC106563479	steroid hormone receptor ERR1-like	100.95	-1.03	-2.04	0.00	0.01
106601037	LOC106601037	coiled-coil domain-containing protein 43-like	126.62	-1.03	-2.03	0.00	0.00
106567422	LOC106567422	solute carrier family 23 member 1-like	669.64	-1.02	-2.03	0.00	0.00
106562989	LOC106562989	tubulin alpha chain, testis-specific-like	189.64	-1.02	-2.03	0.00	0.00
106562547	LOC106562547	deoxyuridine 5'-triphosphate nucleotidohydrola	57.30	-1.02	-2.03	0.00	0.00
106564686	LOC106564686	neuropathy target esterase-like	229.17	-1.02	-2.03	0.00	0.00
100195375	dpp3	dipeptidyl peptidase 3	494.89	-1.02	-2.03	0.00	0.00
106564249	LOC106564249	UPF0390 protein zgc136864-like	137.77	-1.02	-2.03	0.01	0.04
106590364	LOC106590364	serine/threonine-protein kinase RIO3-like	174.02	-1.02	-2.03	0.00	0.00
106604579	LOC106604579	AP-1 complex-associated regulatory protein-like	33.05	-1.02	-2.03	0.00	0.00
100194625	atp5g2	ATP synthase, H+ transporting, mitochondrial F(	1509.28	-1.02	-2.03	0.00	0.00
106579710	LOC106579710	decaprenyl-diphosphate synthase subunit 1-like	47.57	-1.02	-2.03	0.00	0.01
106579534	LOC106579534	mitochondrial fission regulator 1-like	67.83	-1.02	-2.03	0.00	0.02
100194994	rir2	Ribonucleoside-diphosphate reductase subunit	387.70	-1.02	-2.03	0.00	0.00
106597078	LOC106597078	uncharacterized LOC106597078	271.66	-1.02	-2.03	0.00	0.00
106604364	LOC106604364	thiosulfate sulfurtransferase/rhodanese-like do	226.24	-1.02	-2.03	0.00	0.02
100195794	prdx1	peroxiredoxin 1	454.48	-1.02	-2.02	0.00	0.00

106578469	pcolce2	procollagen C-endopeptidase enhancer 2	21.00	-1.02	-2.02	0.00	0.00
106603810	atp5j2	ATP synthase, H+ transporting, mitochondrial F(	2900.78	-1.02	-2.02	0.00	0.03
106611131	LOC106611131	forkhead box protein N2-like	218.35	-1.02	-2.02	0.00	0.01
106569348	LOC106569348	cyclin-dependent-like kinase 5	28.65	-1.01	-2.02	0.00	0.00
106606986	LOC106606986	phosphoribosyl pyrophosphate synthase-associ	35.25	-1.01	-2.02	0.00	0.03
106602990	LOC106602990	cytochrome c oxidase subunit 8B, mitochondria	543.70	-1.01	-2.02	0.00	0.01
106586549	LOC106586549	centromere protein K-like	22.50	-1.01	-2.02	0.00	0.03
106570938	psmb7	proteasome subunit beta 7	972.30	-1.01	-2.02	0.00	0.00
106582805	LOC106582805	vacuolar fusion protein MON1 homolog B-like	67.82	-1.01	-2.02	0.00	0.00
106587045	LOC106587045	leukemia inhibitory factor receptor-like	17.74	-1.01	-2.02	0.00	0.01
106564859	LOC106564859	N-acetyllactosaminide beta-1,3-N-acetylglucosa	23.69	-1.01	-2.02	0.00	0.03
100286461	acot13	acyl-CoA thioesterase 13	54.36	-1.01	-2.01	0.00	0.00
106568922	lrrcc1	leucine rich repeat and coiled-coil centrosomal	13.93	-1.01	-2.01	0.00	0.02
100194940	esco2	establishment of sister chromatid cohesion N-a	70.85	-1.01	-2.01	0.00	0.00
106565058	LOC106565058	eukaryotic translation initiation factor 2 subunit	1303.97	-1.01	-2.01	0.00	0.02
106584335	LOC106584335	glutamate--cysteine ligase regulatory subunit-lil	284.02	-1.01	-2.01	0.00	0.00
106570323	LOC106570323	ATP-binding cassette sub-family F member 1-lik	664.65	-1.01	-2.01	0.00	0.00
106572524	LOC106572524	citrate synthase, mitochondrial-like	290.99	-1.01	-2.01	0.00	0.00
100194641	LOC100194641	malate dehydrogenase 2-2, NAD (mitochondria	1013.16	-1.01	-2.01	0.00	0.03
106561701	LOC106561701	NADH dehydrogenase [ubiquinone] 1 alpha sub	228.22	-1.01	-2.01	0.00	0.02
106595807	LOC106595807	eukaryotic translation initiation factor 2-alpha k	22.67	-1.01	-2.01	0.00	0.00
106577590	LOC106577590	histone H2A.Z	688.58	-1.01	-2.01	0.00	0.00
106585061	slc25a1	NA	252.16	-1.00	-2.01	0.00	0.00
106611342	LOC106611342	sex comb on midleg-like protein 4	15.96	-1.00	-2.00	0.00	0.00
106603944	LOC106603944	complement component 1 Q subcomponent-bi	540.81	-1.00	-2.00	0.01	0.04
100195921	ap4s1	adaptor related protein complex 4 sigma 1 subu	15.95	-1.00	-2.00	0.00	0.00
106570496	LOC106570496	D-dopachrome decarboxylase-A-like	3784.94	-1.00	-2.00	0.00	0.02
106564857	LOC106564857	malate dehydrogenase, cytoplasmic-like	5995.88	-1.00	-2.00	0.00	0.01
106570299	LOC106570299	endonuclease/exonuclease/phosphatase family	764.63	-1.00	-2.00	0.00	0.00
106605689	LOC106605689	apolipoprotein Eb-like	6803.48	-1.00	-2.00	0.01	0.04
106589937	LOC106589937	uncharacterized LOC106589937	267.20	-1.00	-2.00	0.00	0.00
106609896	LOC106609896	pre-mRNA-processing factor 19	22.13	-1.00	-2.00	0.00	0.02

Ploidy group: Triploid

Up-regulated DEGs in parr compared to smolts

NCBI ID	Gene IDs/Locus	Gene name	baseMean	log2FoldChange	Fold change	pvalue	padj
106566987	LOC106566987	uncharacterized LOC106566987	118.47	2.58	5.97	0.00	0.00
106613803	LOC106613803	C2 calcium-dependent domain-containing prote	745.05	2.47	5.56	0.00	0.00
100196184	cish	cytokine inducible SH2 containing protein	323.47	2.40	5.30	0.00	0.03
106569244	LOC106569244	cadherin-6-like	214.69	2.33	5.03	0.00	0.03
100196052	fkbp5	FK506 binding protein 5	19.95	2.30	4.94	0.00	0.02
106572755	LOC106572755	cytochrome P450 2K1-like	2509.80	2.26	4.78	0.00	0.03
106568115	LOC106568115	betaine--homocysteine S-methyltransferase 1-li	39375.61	2.25	4.75	0.00	0.02
100194634	4ebp	eukaryotic translation initiation factor 4E bindir	1006.10	2.23	4.68	0.00	0.04
106583283	LOC106583283	25-hydroxyvitamin D-1 alpha hydroxylase, mito	1104.48	2.21	4.62	0.00	0.00
106611136	LOC106611136	ornithine decarboxylase 1-like	566.02	2.16	4.48	0.00	0.01
106599418	brinp2	BMP/retinoic acid inducible neural specific 2	27.57	2.16	4.46	0.00	0.01
100196284	tnni1	troponin I, slow skeletal muscle	55.39	2.16	4.46	0.00	0.01
106585014	dnajb5	DnaJ heat shock protein family (Hsp40) membe	20.20	2.11	4.33	0.00	0.02
106567984	p2rx5	purinergic receptor P2X 5	14.71	2.11	4.32	0.00	0.02
106566754	LOC106566754	peroxisome proliferator-activated receptor gam	19.64	2.02	4.05	0.00	0.00
106572076	myh7b	myosin heavy chain 7B	12.47	1.91	3.75	0.00	0.01
106600595	odc1	ornithine decarboxylase 1	1674.79	1.89	3.70	0.00	0.02
106574897	LOC106574897	phosphorylase b kinase regulatory subunit alph	63.60	1.88	3.68	0.00	0.00
106605477	LOC106605477	GSK-3-binding protein-like	606.32	1.87	3.64	0.00	0.04
106586997	LOC106586997	fibroblast growth factor 19-like	44.61	1.85	3.61	0.00	0.01
106611368	LOC106611368	type II iodothyronine deiodinase-like	205.73	1.83	3.56	0.00	0.00
106599551	LOC106599551	period circadian protein homolog 2-like	32.57	1.82	3.54	0.00	0.01
106577763	LOC106577763	erythropoietin-like	17.07	1.81	3.50	0.00	0.00
106572462	LOC106572462	pancreatic progenitor cell differentiation and pı	42.73	1.81	3.49	0.00	0.00
106574707	LOC106574707	folate transporter 1-like	22.56	1.80	3.49	0.00	0.00
101448021	gbp	GSK-3-binding protein	429.19	1.80	3.47	0.00	0.00
106601166	LOC106601166	ubiquitin carboxyl-terminal hydrolase 37-like	15.69	1.79	3.47	0.00	0.00
106609884	LOC106609884	tropomodulin-1-like	60.56	1.78	3.44	0.00	0.00
106586563	LOC106586563	cysteine-rich motor neuron 1 protein-like	32.79	1.78	3.44	0.00	0.00
106589211	kif19	kinesin family member 19	18.26	1.78	3.43	0.00	0.02
106578378	LOC106578378	lipocalin-like	24575.55	1.76	3.39	0.00	0.00
106613559	LOC106613559	PDZK1-interacting protein 1-like	21.71	1.74	3.35	0.00	0.03
106565139	LOC106565139	cytokine-inducible SH2-containing protein-like	65.97	1.71	3.28	0.00	0.00
106599162	LOC106599162	cholesterol 7-alpha-monooxygenase-like	2084.77	1.71	3.27	0.00	0.00
106571818	slc27a6	NA	364.87	1.70	3.25	0.00	0.02
106606514	LOC106606514	testis-expressed sequence 2 protein-like	8.30	1.69	3.23	0.00	0.00
100380357	s26a2	Sulfate transporter	54.02	1.69	3.22	0.00	0.04

106576177	LOC106576177	suppressor of cytokine signaling 2-like	142.58	1.68	3.21	0.00	0.00
106573930	LOC106573930	growth arrest and DNA damage-inducible prote	112.52	1.65	3.14	0.00	0.02
106584489	LOC106584489	peroxisome proliferator-activated receptor alph	111.71	1.65	3.14	0.00	0.00
106604578	LOC106604578	sphingosine-1-phosphate lyase 1-like	11.51	1.63	3.10	0.00	0.00
106612801	LOC106612801	carbonic anhydrase 4-like	44.54	1.63	3.09	0.00	0.00
106609979	LOC106609979	spermine oxidase-like	177.87	1.62	3.08	0.00	0.04
106604215	LOC106604215	sideroflexin-1-like	5.60	1.61	3.06	0.00	0.01
100194692	LOC100194692	Krueppel-like factor 11	297.67	1.61	3.06	0.00	0.00
106608146	LOC106608146	kelch repeat and BTB domain-containing protei	24.86	1.61	3.05	0.00	0.05
100136442	ghr1	growth hormone receptor isoform 1 precursor	847.97	1.60	3.04	0.00	0.00
106583818	slmap	NA	17.12	1.60	3.03	0.00	0.00
106571601	LOC106571601	type III iodothyronine deiodinase-like	417.60	1.60	3.02	0.00	0.00
106570339	tubb4b	NA	98.77	1.59	3.01	0.00	0.00
106588047	LOC106588047	asc-type amino acid transporter 1-like	6.00	1.59	3.00	0.00	0.01
106568345	LOC106568345	arrestin domain-containing protein 3-like	116.45	1.59	3.00	0.00	0.04
106607574	LOC106607574	zinc transporter 1-like	14.06	1.59	3.00	0.00	0.00
106568987	LOC106568987	volume-regulated anion channel subunit LRRC8	160.63	1.58	2.99	0.00	0.00
100192341	elovl2	ELOVL fatty acid elongase 2	5132.13	1.58	2.98	0.00	0.00
106577245	LOC106577245	S-adenosylmethionine synthase isoform type-1	122.28	1.57	2.98	0.00	0.00
106606032	LOC106606032	carboxypeptidase Q-like	23.02	1.57	2.97	0.00	0.01
100136550	LOC100136550	insulin-like growth factor II	179.05	1.56	2.95	0.00	0.03
106606191	LOC106606191	monocarboxylate transporter 7-like	29.51	1.55	2.93	0.00	0.01
100194600	bhmt	betaine-homocysteine methyltransferase	2531.98	1.54	2.90	0.00	0.05
106563261	LOC106563261	nucleus accumbens-associated protein 2-like	236.75	1.52	2.87	0.00	0.00
100380829	tf2l1	Transcription factor CP2-like protein 1	1713.06	1.52	2.87	0.00	0.00
100136444	fabp3	fatty acid binding protein 3	95.48	1.52	2.87	0.00	0.00
106590087	cssa29h18orf63	chromosome ssa29 open reading frame, humar	8.06	1.52	2.86	0.00	0.00
106576588	LOC106576588	oocyte zinc finger protein XlCOF6-like	283.91	1.52	2.86	0.00	0.00
106610848	LOC106610848	cytochrome P450 1B1 pseudogene	29.66	1.52	2.86	0.00	0.01
106583156	LOC106583156	serine/threonine-protein kinase 40	171.73	1.52	2.86	0.00	0.00
106567289	LOC106567289	suppressor of cytokine signaling 2-like	270.65	1.50	2.83	0.00	0.00
106570052	LOC106570052	phosphatidate phosphatase LPIN1-like	63.97	1.50	2.82	0.00	0.00
100194597	ahcy	adenosylhomocysteinase	21152.16	1.50	2.82	0.00	0.01
106572759	LOC106572759	Krueppel-like factor 15 pseudogene	36.45	1.49	2.82	0.00	0.01
106609509	LOC106609509	aryl hydrocarbon receptor nuclear translocator-	386.61	1.49	2.81	0.00	0.03
106566781	LOC106566781	solute carrier family 26 member 6-like	22.67	1.49	2.80	0.00	0.01
106565889	LOC106565889	endothelin-converting enzyme 1-like	97.25	1.49	2.80	0.00	0.00
106568366	LOC106568366	uncharacterized LOC106568366	1721.25	1.48	2.78	0.00	0.02
106604946	LOC106604946	fibroblast growth factor receptor-like 1	14.82	1.47	2.78	0.00	0.00
106572800	LOC106572800	uncharacterized LOC106572800	84.37	1.47	2.78	0.00	0.00

106589294	LOC106589294	unconventional myosin-Id-like	22.33	1.47	2.78	0.00	0.00
106589124	LOC106589124	tribbles homolog 1-like	21.08	1.47	2.77	0.00	0.01
106603656	LOC106603656	DNA (cytosine-5)-methyltransferase 3A-like	247.19	1.46	2.76	0.00	0.02
106588001	LOC106588001	Krueppel-like factor 13 pseudogene	13.59	1.46	2.75	0.00	0.00
106565979	LOC106565979	cytokine-inducible SH2-containing protein-like	55.22	1.45	2.74	0.00	0.00
106613443	adgrl2	adhesion G protein-coupled receptor L2	14.02	1.45	2.74	0.00	0.02
106603685	LOC106603685	tripartite motif-containing protein 16-like	5.75	1.45	2.73	0.00	0.00
106565346	LOC106565346	peptidyl-prolyl cis-trans isomerase FKBP5-like	547.99	1.45	2.73	0.00	0.00
100306779	ext1c	exostoses (multiple) 1c	15.82	1.45	2.73	0.00	0.00
106562381	LOC106562381	arylamine N-acetyltransferase, pineal gland isoz	18.17	1.44	2.72	0.00	0.00
106563377	LOC106563377	erythropoietin-like	79.25	1.44	2.71	0.00	0.00
106584535	LOC106584535	E3 ubiquitin-protein ligase RFWD3-like	28.48	1.44	2.71	0.00	0.01
106586099	LOC106586099	GTPase IMAP family member 8-like	27.98	1.43	2.70	0.00	0.00
106564859	LOC106564859	N-acetyllactosaminide beta-1,3-N-acetylglucosa	37.96	1.43	2.70	0.00	0.01
106569555	LOC106569555	transcriptional repressor p66 alpha-like	164.34	1.43	2.69	0.00	0.00
100195420	pck1	phosphoenolpyruvate carboxykinase 1	62.20	1.43	2.69	0.00	0.00
106610846	LOC106610846	cysteine-rich motor neuron 1 protein-like	51.18	1.42	2.68	0.00	0.01
106573119	LOC106573119	caveolin-1-like	77.74	1.42	2.68	0.00	0.00
106576759	LOC106576759	alpha-2,8-sialyltransferase 8E-like	148.89	1.42	2.68	0.00	0.01
106563612	LOC106563612	probetacellulin-like	40.82	1.41	2.65	0.00	0.02
106604461	LOC106604461	multidrug resistance-associated protein 5-like	1695.55	1.40	2.65	0.00	0.03
106612881	LOC106612881	serine/threonine-protein kinase PAK 1-like	63.59	1.40	2.65	0.00	0.02
106612829	LOC106612829	phosphoserine phosphatase-like	350.08	1.40	2.64	0.00	0.01
106585128	LOC106585128	uncharacterized protein C8orf4 homolog	139.42	1.40	2.64	0.00	0.00
106584385	LOC106584385	Krueppel-like factor 9	18.50	1.40	2.63	0.00	0.04
106607660	LOC106607660	disintegrin and metalloproteinase domain-conta	45.98	1.40	2.63	0.00	0.01
106588344	LOC106588344	guanine nucleotide-binding protein G(s) subunit	17.68	1.39	2.62	0.00	0.01
106566064	LOC106566064	guanine nucleotide-binding protein G(i) subunit	214.59	1.39	2.62	0.00	0.00
106560734	faxdc2	fatty acid hydroxylase domain containing 2	3871.91	1.39	2.61	0.00	0.04
100195645	st2s2	Cytosolic sulfotransferase 2	375.27	1.39	2.61	0.00	0.00
106586529	tsc22d1	NA	122.04	1.38	2.61	0.00	0.01
106580756	LOC106580756	cytochrome P450 2M1	9761.64	1.38	2.61	0.00	0.04
106589373	slc35g1	NA	11.38	1.38	2.61	0.00	0.00
106566319	LOC106566319	plexin-B1-like	11.32	1.38	2.60	0.00	0.00
106588186	LOC106588186	uncharacterized LOC106588186	78.56	1.38	2.59	0.00	0.00
106604208	LOC106604208	sideroflexin-1-like	7.78	1.37	2.58	0.00	0.02
106585566	LOC106585566	neurofilament light polypeptide-like	7.69	1.37	2.58	0.00	0.00
106584507	slc41a2	NA	30.29	1.36	2.57	0.00	0.00
106571200	lpgat1	lysophosphatidylglycerol acyltransferase 1	38.70	1.36	2.57	0.00	0.04
106586453	LOC106586453	phosphatidylcholine-sterol acyltransferase-like	35.78	1.36	2.57	0.00	0.01

106567009	LOC106567009	probable E3 ubiquitin-protein ligase DTX3	82.08	1.36	2.56	0.00	0.00
100194800	tieg3	Transforming growth factor-beta-inducible earl	419.87	1.35	2.56	0.00	0.02
106612523	LOC106612523	uncharacterized LOC106612523	8.29	1.35	2.55	0.00	0.00
106613922	ect2	epithelial cell transforming 2	45.95	1.35	2.55	0.00	0.02
106565112	LOC106565112	cryptochrome-1-like	85.32	1.35	2.55	0.00	0.02
106563762	LOC106563762	A disintegrin and metalloproteinase with throm	19.86	1.35	2.55	0.00	0.00
106568897	LOC106568897	dihydropyrimidine dehydrogenase [NADP(+)]	2750.67	1.35	2.54	0.00	0.00
106566182	LOC106566182	sodium- and chloride-dependent taurine transp	526.82	1.34	2.54	0.00	0.01
106601411	LOC106601411	ras-related protein Rap-2a-like	18.91	1.34	2.53	0.00	0.00
106590410	LOC106590410	zinc finger protein GLIS2-like	29.36	1.34	2.53	0.00	0.04
106569990	LOC106569990	intermediate filament family orphan 1-like	13.82	1.33	2.52	0.00	0.00
106573091	LOC106573091	prickle-like protein 1	29.16	1.33	2.51	0.00	0.00
106587221	LOC106587221	UDP-glucuronosyltransferase 2C1 pseudogene	66.26	1.33	2.51	0.00	0.00
100380591	hpcl1	Hippocalcin-like protein 1	279.68	1.33	2.51	0.00	0.01
106612672	LOC106612672	bone morphogenetic protein 5-like	284.76	1.32	2.50	0.00	0.00
106565780	LOC106565780	protein FAM83D-like	43.06	1.32	2.50	0.00	0.00
106574898	LOC106574898	phosphorylase b kinase regulatory subunit alph	214.20	1.32	2.50	0.00	0.04
106584550	LOC106584550	alpha-aminoadipic semialdehyde synthase, mito	2067.49	1.32	2.49	0.00	0.01
100195201	aco1	aconitase 1	1691.04	1.32	2.49	0.00	0.01
106565525	LOC106565525	uncharacterized LOC106565525	123.29	1.31	2.48	0.00	0.01
106604175	LOC106604175	solute carrier family 25 member 48-like	349.51	1.30	2.47	0.00	0.04
106563018	LOC106563018	phosphatidylinositol 3-kinase regulatory subuni	1677.37	1.30	2.47	0.00	0.00
106606194	LOC106606194	monocarboxylate transporter 7-like	27.39	1.30	2.46	0.00	0.02
106569160	LOC106569160	PH domain leucine-rich repeat-containing prote	63.38	1.30	2.46	0.00	0.00
106573477	pex11a	peroxisomal biogenesis factor 11 alpha	104.85	1.30	2.46	0.00	0.00
106566558	top1	NA	130.55	1.29	2.45	0.00	0.03
106572957	LOC106572957	transketolase-like	393.35	1.29	2.44	0.00	0.00
100380506	rqcd1	CCR4-NOT transcription complex subunit 9	201.27	1.29	2.44	0.00	0.00
100380618	efhd2	EF-hand domain family member D2	1007.77	1.29	2.44	0.00	0.01
106577502	LOC106577502	mitotic spindle assembly checkpoint protein MA	15.45	1.28	2.44	0.00	0.04
106600825	LOC106600825	nuclear receptor subfamily 1 group D member 1	148.57	1.28	2.43	0.00	0.00
106571312	LOC106571312	S-adenosylmethionine decarboxylase proenzym	1153.52	1.28	2.43	0.00	0.00
106587712	LOC106587712	proline-serine-threonine phosphatase-interacti	16.24	1.28	2.43	0.00	0.00
106566379	LOC106566379	solute carrier family 25 member 33	152.72	1.28	2.43	0.00	0.01
106604510	LOC106604510	NAD-dependent protein deacetylase sirtuin-1-li	24.90	1.28	2.43	0.00	0.00
106595274	LOC106595274	OX-2 membrane glycoprotein-like	60.26	1.28	2.43	0.00	0.00
106571166	LOC106571166	absent in melanoma 1 protein-like	172.86	1.27	2.42	0.00	0.00
100136533	gdh2	glutamate dehydrogenase	6810.03	1.27	2.42	0.00	0.00
106612691	LOC106612691	glucagon receptor-like	65.36	1.27	2.42	0.00	0.00
106566779	LOC106566779	monoglyceride lipase-like	183.80	1.27	2.41	0.00	0.00

106599887	LOC106599887	5-beta-cholestane-3-alpha,7-alpha-diol 12-alpha	4336.90	1.27	2.41	0.00	0.03
106575975	LOC106575975	phosphorylase b kinase regulatory subunit alph	252.23	1.27	2.41	0.00	0.00
106574135	LOC106574135	uncharacterized LOC106574135	67.53	1.26	2.40	0.00	0.00
106586678	otc	ornithine carbamoyltransferase	35.32	1.26	2.40	0.00	0.01
100306807	egln2	Egl nine homolog 2	187.13	1.26	2.40	0.00	0.00
106578804	LOC106578804	kinesin-like protein KIF20B	39.63	1.26	2.39	0.00	0.00
106612800	LOC106612800	transcription elongation factor SPT6-like	40.65	1.25	2.39	0.00	0.00
106607066	LOC106607066	importin subunit alpha-1-like	116.04	1.25	2.38	0.00	0.00
106576259	LOC106576259	protein FAM19A2-like	35.66	1.25	2.38	0.00	0.02
106579956	pptc7	NA	233.98	1.25	2.38	0.00	0.05
106573245	kiaa0196	KIAA0196 ortholog	25.07	1.25	2.38	0.00	0.01
106575607	LOC106575607	folate transporter 1-like	73.74	1.25	2.37	0.00	0.01
106571198	LOC106571198	zinc transporter 1-like	466.23	1.25	2.37	0.00	0.00
106586229	LOC106586229	integrin alpha-6-like	338.67	1.24	2.37	0.00	0.00
106565623	LOC106565623	ethanolamine kinase 1-like	176.74	1.24	2.37	0.00	0.01
106574162	LOC106574162	protein naked cuticle homolog 2-like	20.45	1.24	2.37	0.00	0.00
106610734	LOC106610734	phosphatidate phosphatase LPIN1-like	111.91	1.24	2.36	0.00	0.00
106608056	LOC106608056	glycerophosphocholine phosphodiesterase GPC	436.62	1.24	2.36	0.00	0.00
106578457	LOC106578457	uncharacterized LOC106578457	175.21	1.24	2.36	0.00	0.00
100196783	eki1	Ethanolamine kinase 1	4879.86	1.24	2.36	0.00	0.00
106563294	LOC106563294	ADP-ribosylation factor-like protein 3	14.48	1.23	2.35	0.00	0.00
106607732	LOC106607732	myristoylated alanine-rich C-kinase substrate-lil	469.82	1.23	2.35	0.00	0.00
106601197	LOC106601197	uncharacterized LOC106601197	17.80	1.23	2.35	0.00	0.04
106606941	LOC106606941	protein SCO1 homolog, mitochondrial-like	36.18	1.23	2.34	0.00	0.00
106605689	LOC106605689	apolipoprotein Eb-like	9437.22	1.23	2.34	0.00	0.00
106578978	LOC106578978	solute carrier family 35 member G1-like	147.19	1.23	2.34	0.00	0.00
100196094	LOC100196094	dipeptidyl peptidase 1	426.44	1.23	2.34	0.00	0.01
106568460	LOC106568460	zinc finger protein 462-like	11.35	1.22	2.34	0.00	0.00
106562199	LOC106562199	myocardial zonula adherens protein-like	27.54	1.22	2.33	0.00	0.00
106611197	ankrd9	ankyrin repeat domain 9	273.02	1.22	2.33	0.00	0.00
100380573	typx	Tryparedoxin	38.91	1.22	2.33	0.00	0.04
106584340	LOC106584340	leucine-rich repeat-containing protein 7-like	17.24	1.22	2.32	0.00	0.00
100136393	pparg	peroxisome proliferator activated receptor gam	480.31	1.21	2.32	0.00	0.00
106582731	LOC106582731	mucin-5AC-like	13.68	1.21	2.32	0.00	0.03
106563180	LOC106563180	protein phosphatase PTC7 homolog	29.63	1.21	2.31	0.00	0.00
106587299	LOC106587299	SHC SH2 domain-binding protein 1-like	21.89	1.20	2.30	0.00	0.04
106589802	fam195a	MAPK regulated corepressor interacting proteir	427.16	1.20	2.30	0.00	0.02
106580061	LOC106580061	guanine deaminase-like	172.85	1.20	2.30	0.00	0.00
106583638	LOC106583638	cytosolic 10-formyltetrahydrofolate dehydroger	41.30	1.20	2.29	0.00	0.05
106572339	LOC106572339	high mobility group protein HMG-I/HMG-Y-like	13.93	1.20	2.29	0.00	0.04

106566486	LOC106566486	extracellular sulfatase Sulf-2-like	92.33	1.20	2.29	0.00	0.02
106576182	LOC106576182	host cell factor 2-like	65.23	1.19	2.29	0.00	0.00
106573187	LOC106573187	bcl-2-like protein 13	188.05	1.19	2.29	0.00	0.04
106568158	LOC106568158	heparanase-like	27.40	1.19	2.29	0.00	0.01
106565909	LOC106565909	delta-1-pyrroline-5-carboxylate dehydrogenase	154.08	1.19	2.29	0.00	0.02
106568392	LOC106568392	ras-GEF domain-containing family member 1B-E	36.82	1.19	2.29	0.00	0.04
106613792	LOC106613792	SHC-transforming protein 2-like	60.85	1.19	2.28	0.00	0.00
106572146	LOC106572146	perlwapin-like	235.69	1.19	2.28	0.00	0.00
106605048	LOC106605048	inactive serine protease 35-like	53.42	1.19	2.28	0.00	0.00
106565174	LOC106565174	hyaluronidase-2-like	236.40	1.19	2.27	0.00	0.00
106607162	LOC106607162	thyrotroph embryonic factor-like	89.76	1.18	2.27	0.00	0.02
106601868	LOC106601868	cyclin-F-like	27.96	1.18	2.27	0.00	0.05
106577470	LOC106577470	B-cell lymphoma 3 protein-like	33.66	1.18	2.27	0.00	0.00
106588877	oxnad1	oxidoreductase NAD binding domain containing	42.18	1.18	2.26	0.00	0.02
100195486	ccnd2	G1/S-specific cyclin-D2	173.23	1.17	2.25	0.00	0.01
106609503	LOC106609503	forkhead box protein M1-like	18.99	1.17	2.25	0.00	0.03
106590348	LOC106590348	receptor-type tyrosine-protein phosphatase mu	18.79	1.17	2.25	0.00	0.00
106612433	lrch3	leucine rich repeats and calponin homology dor	14.75	1.17	2.25	0.00	0.01
106585805	LOC106585805	drebrin-like protein B	60.66	1.17	2.25	0.00	0.00
106588345	LOC106588345	CDC42 small effector protein 1-like	76.58	1.16	2.24	0.00	0.01
106607748	LOC106607748	low-density lipoprotein receptor-related protei	132.46	1.16	2.23	0.00	0.00
100196419	kith	Thymidine kinase, cytosolic	96.62	1.16	2.23	0.00	0.00
106585072	LOC106585072	putative aminopeptidase W07G4.4	2299.53	1.16	2.23	0.00	0.00
106589769	LOC106589769	MORN repeat-containing protein 4-like	83.85	1.15	2.23	0.00	0.02
106564234	LOC106564234	mitochondrial dicarboxylate carrier-like	293.62	1.15	2.22	0.00	0.02
106572303	LOC106572303	helicase ARIP4-like	70.64	1.15	2.22	0.00	0.01
106561512	LOC106561512	transmembrane protein 168-like	43.95	1.15	2.22	0.00	0.03
106584144	LOC106584144	regulator of G-protein signaling 5-like	24.97	1.15	2.21	0.00	0.00
106577478	LOC106577478	mitochondrial import receptor subunit TOM40	256.85	1.14	2.21	0.00	0.01
106560949	LOC106560949	aminopeptidase N-like	584.88	1.14	2.21	0.00	0.00
106603543	LOC106603543	cytoskeleton-associated protein 2-like	95.73	1.14	2.20	0.00	0.03
106561617	LOC106561617	G1/S-specific cyclin-D1-like	75.73	1.14	2.20	0.00	0.00
106599389	LOC106599389	kinesin-like protein KIF2C	95.75	1.14	2.20	0.00	0.00
106587076	LOC106587076	transcription factor E2F8-like	27.53	1.14	2.20	0.00	0.00
106580024	LOC106580024	SH2 domain-containing protein 3C-like	48.56	1.14	2.20	0.00	0.01
106607871	LOC106607871	uncharacterized LOC106607871	17.33	1.14	2.20	0.00	0.01
100195587	borea	Borealin	96.89	1.14	2.20	0.00	0.00
106572609	LOC106572609	hepatocyte nuclear factor 4-alpha-like	98.59	1.13	2.19	0.00	0.01
106581110	LOC106581110	vasodilator-stimulated phosphoprotein-like	267.50	1.13	2.19	0.00	0.01
106560912	LOC106560912	actin-binding protein anillin-like	105.71	1.13	2.18	0.00	0.00

106564352	LOC106564352	thymidine kinase, cytosolic-like	226.38	1.12	2.18	0.00	0.00
100196629	serb	Phosphoserine phosphatase	230.42	1.12	2.18	0.00	0.00
106584819	LOC106584819	atlastin-2-like	221.79	1.12	2.18	0.00	0.00
106610321	mxd4	MAX dimerization protein 4	75.29	1.12	2.18	0.00	0.00
100286727	ska1	spindle and kinetochore associated complex sul	31.74	1.12	2.18	0.00	0.00
106608508	LOC106608508	fatty acid-binding protein, intestinal-like	1043.31	1.12	2.17	0.00	0.00
106579350	LOC106579350	Wilms tumor protein 1-interacting protein-like	31.46	1.12	2.17	0.00	0.00
106612955	LOC106612955	tsukushin-like	602.97	1.12	2.17	0.00	0.01
100380723	LOC100380723	exportin-1	60.07	1.12	2.17	0.00	0.04
106599164	LOC106599164	xenotropic and polytropic retrovirus receptor 1	48.80	1.11	2.16	0.00	0.00
106577505	LOC106577505	apolipoprotein A-IV-like	2077.40	1.11	2.16	0.00	0.00
100286615	phs	Pterin-4-alpha-carbinolamine dehydratase	1482.27	1.11	2.16	0.00	0.00
106562460	LOC106562460	NAD(P) transhydrogenase, mitochondrial-like	55.63	1.11	2.16	0.00	0.00
100499254	sell	NA	137.42	1.11	2.15	0.00	0.00
106580581	LOC106580581	glucokinase-like	10960.54	1.10	2.15	0.00	0.00
100380301	clcn3	chloride channel 3	804.01	1.10	2.15	0.00	0.01
106584748	LOC106584748	protein regulator of cytokinesis 1-like	23.91	1.10	2.15	0.00	0.02
106571015	LOC106571015	sorting nexin-17-like	42.25	1.10	2.14	0.00	0.02
106601356	LOC106601356	uncharacterized LOC106601356	258.25	1.10	2.14	0.00	0.00
100136930	LOC100136930	eggshell protein	111.66	1.10	2.14	0.00	0.01
106579552	oplah	5-oxoprolinase (ATP-hydrolysing)	119.00	1.09	2.13	0.00	0.00
106567942	LOC106567942	cytoskeleton-associated protein 2-like	230.45	1.09	2.13	0.00	0.01
106600984	LOC106600984	ADP-ribosylation factor-like protein 5B	72.88	1.09	2.12	0.00	0.00
106609112	LOC106609112	cadherin EGF LAG seven-pass G-type receptor 1	89.45	1.09	2.12	0.00	0.03
106575194	LOC106575194	phospholipase A2 inhibitor 31 kDa subunit-like	3585.00	1.08	2.11	0.00	0.00
106599706	LOC106599706	cadherin-2-like	1305.11	1.08	2.11	0.00	0.00
106567424	pwwp2a	NA	31.30	1.08	2.11	0.00	0.01
106611938	LOC106611938	protein FAM53C-like	130.81	1.08	2.11	0.00	0.01
106579534	LOC106579534	mitochondrial fission regulator 1-like	85.51	1.08	2.11	0.00	0.01
106605032	LOC106605032	NADP-dependent malic enzyme-like	4149.42	1.07	2.11	0.00	0.02
106589036	LOC106589036	mpv17-like protein	182.16	1.07	2.11	0.00	0.00
106603918	LOC106603918	deoxycytidine kinase-like	63.77	1.07	2.11	0.00	0.04
106568486	agxt2	alanine--glyoxylate aminotransferase 2	3267.27	1.07	2.10	0.00	0.00
106578758	LOC106578758	peptidyl-prolyl cis-trans isomerase A-like	607.40	1.07	2.10	0.00	0.00
106603348	LOC106603348	mitochondrial uncoupling protein 2	811.71	1.07	2.10	0.00	0.00
106590647	LOC106590647	ATP-binding cassette sub-family D member 3	956.59	1.07	2.10	0.00	0.01
106584808	LOC106584808	heterogeneous nuclear ribonucleoprotein L-like	44.47	1.07	2.09	0.00	0.00
106567189	LOC106567189	Krueppel-like factor 11	181.94	1.05	2.07	0.00	0.02
106612474	mcam	melanoma cell adhesion molecule	242.63	1.05	2.07	0.00	0.03
106565244	LOC106565244	Krueppel-like factor 15	87.26	1.05	2.07	0.00	0.01

106567863	LOC106567863	ribonucleoside-diphosphate reductase large sul	184.97	1.05	2.07	0.00	0.04
106578486	LOC106578486	ATP-binding cassette sub-family D member 3-lik	1439.23	1.05	2.06	0.00	0.03
106576355	LOC106576355	lysine-specific demethylase 7B-like	21.05	1.04	2.06	0.00	0.03
106585818	LOC106585818	COMM domain-containing protein 1-like	63.64	1.04	2.06	0.00	0.02
106573152	LOC106573152	uncharacterized LOC106573152	466.66	1.04	2.06	0.00	0.00
100380427	errfi	ERBB receptor feedback inhibitor 1	706.77	1.04	2.06	0.00	0.02
100380819	wee1	WEE1 homolog (S. pombe)	33.96	1.04	2.06	0.00	0.00
106589877	LOC106589877	sphingomyelin synthase-related protein 1-like	25.81	1.04	2.06	0.00	0.02
106582972	LOC106582972	pantothenate kinase 4-like	260.67	1.04	2.05	0.00	0.02
100195269	phop1	Probable phosphatase phospho1	1620.28	1.04	2.05	0.00	0.00
106561604	LOC106561604	aurora kinase B-like	86.41	1.03	2.05	0.00	0.00
106571428	LOC106571428	polypeptide N-acetylgalactosaminyltransferase	847.85	1.03	2.05	0.00	0.04
106608778	LOC106608778	insulin receptor substrate 1-B-like	26.31	1.03	2.04	0.00	0.00
106609794	LOC106609794	glucoside xylosyltransferase 1-like	60.52	1.03	2.04	0.00	0.00
106586216	myo3b	myosin IIIB	46.61	1.02	2.03	0.00	0.04
106571314	LOC106571314	monocarboxylate transporter 10-like	391.39	1.02	2.03	0.00	0.01
100306840	ivns1abp	influenza virus NS1A binding protein	409.49	1.02	2.03	0.00	0.00
106560623	LOC106560623	angiomotin-like 2a	25.02	1.02	2.03	0.00	0.00
106570548	LOC106570548	geminin-like	74.28	1.02	2.03	0.00	0.03
100195213	dcup	Uroporphyrinogen decarboxylase	141.67	1.02	2.02	0.00	0.00
106577008	LOC106577008	bifunctional 3'-phosphoadenosine 5'-phosphosu	199.10	1.02	2.02	0.00	0.00
106563565	LOC106563565	carbonyl reductase [NADPH] 1-like	878.78	1.02	2.02	0.00	0.00
106574860	LOC106574860	indian hedgehog B protein-like	57.14	1.01	2.01	0.00	0.00
106601563	LOC106601563	3 beta-hydroxysteroid dehydrogenase type 7-lik	1741.27	1.01	2.01	0.00	0.00
106564855	LOC106564855	E3 ubiquitin-protein ligase pellino homolog 1-lik	74.45	1.01	2.01	0.00	0.01
100380646	LOC100380646	cat eye syndrome critical region protein 5 homc	171.19	1.01	2.01	0.00	0.00
106567006	LOC106567006	probable E3 ubiquitin-protein ligase DTX3	62.21	1.01	2.01	0.00	0.00
106589946	LOC106589946	nuclear factor 1 X-type-like	1046.03	1.01	2.01	0.00	0.00
106573634	LOC106573634	zinc finger protein 708-like	106.36	1.00	2.00	0.00	0.00

Down-regulated DEGs in parr compared to smolt

NCBI ID	Gene IDs/Locus	Gene name	baseMean	log2FoldChange	Fold change	pvalue	padj
106565178	LOC106565178	interferon-induced protein 44-like	108.90	-3.61	-12.22	0.00	0.00
106600509	LOC106600509	alcohol dehydrogenase 1-like	2798.51	-3.09	-8.50	0.00	0.05
106585649	LOC106585649	complement C1q-like protein 2	367.24	-3.01	-8.08	0.00	0.04
106609923	LOC106609923	fibrinogen gamma chain-like	49.44	-3.00	-8.01	0.00	0.03
106585878	LOC106585878	C-C motif chemokine 19-like	49.89	-2.96	-7.78	0.00	0.01
106597213	LOC106597213	uncharacterized LOC106597213	44.83	-2.92	-7.58	0.00	0.00
106610455	LOC106610455	GTPase IMAP family member 4-like	25.25	-2.91	-7.50	0.00	0.02
106607151	LOC106607151	interferon-induced very large GTPase 1-like	58.31	-2.90	-7.44	0.00	0.01
106565855	LOC106565855	C-C motif chemokine 19-like	155.44	-2.86	-7.25	0.00	0.01

100380321	pisd	phosphatidylserine decarboxylase	1007.40	-2.84	-7.16	0.00	0.00
106560487	angptl3	angiopoietin like 3	263.52	-2.83	-7.09	0.00	0.01
100195695	urah	HIU hydrolase	84.07	-2.78	-6.86	0.00	0.00
106568762	LOC106568762	B-cell lymphoma 6 protein-like	77.05	-2.75	-6.74	0.00	0.00
106606375	LOC106606375	Ig heavy chain Mem5-like	34.08	-2.69	-6.45	0.00	0.00
106575701	LOC106575701	uncharacterized LOC106575701	31.10	-2.60	-6.04	0.00	0.01
100136450	LOC100136450	CD8 alpha	9.75	-2.59	-6.03	0.00	0.00
106583431	LOC106583431	interferon-induced protein 44-like	98.33	-2.57	-5.92	0.00	0.00
106580394	LOC106580394	L-serine dehydratase/L-threonine deaminase-lil	15.68	-2.55	-5.86	0.00	0.00
106607153	LOC106607153	interferon-induced very large GTPase 1-like	126.77	-2.55	-5.84	0.00	0.04
106566489	LOC106566489	scavenger receptor cysteine-rich type 1 protein	11.58	-2.54	-5.80	0.00	0.00
100195495	mmp13	Collagenase 3	14.72	-2.52	-5.75	0.00	0.00
106578237	LOC106578237	sialic acid-binding Ig-like lectin 16	152.52	-2.52	-5.72	0.00	0.03
106609356	LOC106609356	endoplasmin-like	436.86	-2.50	-5.65	0.00	0.03
106576386	LOC106576386	uncharacterized LOC106576386	56.69	-2.44	-5.44	0.00	0.02
106560552	LOC106560552	complement factor H-like	1182.04	-2.42	-5.35	0.00	0.01
106576339	LOC106576339	Ig mu chain C region-like	8.41	-2.39	-5.23	0.00	0.01
106605673	LOC106605673	B-cell antigen receptor complex-associated prot	41.80	-2.36	-5.14	0.00	0.00
106575706	LOC106575706	uncharacterized LOC106575706	37.46	-2.36	-5.14	0.00	0.00
106606374	LOC106606374	immunoglobulin lambda-like polypeptide 1	18.63	-2.35	-5.08	0.00	0.01
106563358	LOC106563358	C-C motif chemokine 19-like	14.64	-2.33	-5.03	0.00	0.00
106602156	LOC106602156	uncharacterized LOC106602156	11.59	-2.33	-5.03	0.00	0.00
106563238	LOC106563238	glutamate receptor ionotropic, NMDA 1	10.22	-2.29	-4.87	0.00	0.00
100136451	LOC100136451	CD8 beta	7.10	-2.28	-4.86	0.00	0.00
106610407	LOC106610407	up-regulator of cell proliferation-like	20.97	-2.25	-4.75	0.00	0.03
106613140	LOC106613140	sodium- and chloride-dependent GABA transpo	8.36	-2.21	-4.62	0.00	0.02
100195428	mmp9	matrix metalloproteinase 9	7.49	-2.19	-4.56	0.00	0.03
106560485	LOC106560485	ficolin-1-like	624.49	-2.18	-4.52	0.00	0.00
106608805	LOC106608805	uncharacterized LOC106608805	4560.57	-2.18	-4.52	0.00	0.00
106560917	LOC106560917	olfactomedin-4-like	55.80	-2.15	-4.45	0.00	0.00
100196322	pdl1	Programmed cell death 1 ligand 1	18.03	-2.15	-4.45	0.00	0.00
106577984	LOC106577984	serine/threonine-protein kinase Nek1-like	5.13	-2.15	-4.43	0.00	0.00
106608989	LOC106608989	SLAM family member 9-like	26.30	-2.12	-4.35	0.00	0.04
106578026	LOC106578026	uncharacterized LOC106578026	6.82	-2.11	-4.32	0.00	0.00
106561069	LOC106561069	protein FAM60A-like	12.79	-2.11	-4.32	0.00	0.04
100136548	irf-1	interferon regulatory factor 1	165.47	-2.09	-4.27	0.00	0.00
106575578	LOC106575578	uncharacterized LOC106575578	128.97	-2.07	-4.20	0.00	0.00
100526661	foxp3	forkhead box P3	5.72	-2.06	-4.18	0.00	0.00
106563789	LOC106563789	uncharacterized LOC106563789	236.56	-2.05	-4.15	0.00	0.00
106565734	LOC106565734	probable G-protein coupled receptor 173	193.29	-2.05	-4.13	0.00	0.01

106604696	LOC106604696	saxitoxin and tetrodotoxin-binding protein 2-lik	132.48	-2.03	-4.08	0.00	0.00
106576430	LOC106576430	cytoskeleton-associated protein 4-like	29.13	-2.03	-4.08	0.00	0.01
106606790	LOC106606790	Ig mu chain C region membrane-bound form-lik	57.57	-2.02	-4.07	0.00	0.00
106575435	LOC106575435	Ig kappa chain V-V region MOPC 21-like	15.89	-2.02	-4.06	0.00	0.00
106564407	LOC106564407	Ig kappa chain V-IV region JI-like	17.18	-2.02	-4.05	0.00	0.00
106606551	LOC106606551	nuclear GTPase SLIP-GC-like	52.85	-2.00	-4.01	0.00	0.00
106577998	LOC106577998	protein NDRG2-like	1124.82	-2.00	-4.01	0.00	0.00
106583432	LOC106583432	interferon-induced protein 44-like	7.69	-1.99	-3.97	0.00	0.00
106577462	LOC106577462	trypsin-like	5.94	-1.98	-3.96	0.00	0.01
106588303	LOC106588303	Ig kappa chain V-I region Walker-like	34.25	-1.97	-3.92	0.00	0.00
106579065	LOC106579065	sarcoplasmic/endoplasmic reticulum calcium AT	16.10	-1.97	-3.91	0.00	0.01
106582501	LOC106582501	interferon-induced guanylate-binding protein 1	31.54	-1.97	-3.90	0.00	0.00
106602565	LOC106602565	zinc finger protein RFP-like	5.46	-1.96	-3.90	0.00	0.04
106581164	LOC106581164	glia maturation factor gamma-like	29.56	-1.96	-3.90	0.00	0.03
106575521	LOC106575521	uncharacterized LOC106575521	9.79	-1.95	-3.87	0.00	0.03
106581655	LOC106581655	P2Y purinoceptor 8-like	8.99	-1.95	-3.86	0.00	0.00
100195724	sh21a	SH2 domain-containing protein 1A	18.30	-1.95	-3.86	0.00	0.03
100196468	mbl2	Mannose-binding protein C	129.01	-1.94	-3.83	0.00	0.00
106570863	LOC106570863	DNA-binding protein HU 2-like	11.77	-1.94	-3.82	0.00	0.00
106609550	LOC106609550	angiopoietin-related protein 5-like	16.50	-1.93	-3.82	0.00	0.00
106565156	LOC106565156	uncharacterized LOC106565156	26.32	-1.93	-3.82	0.00	0.00
106577198	LOC106577198	catechol O-methyltransferase domain-containir	98.61	-1.93	-3.81	0.00	0.02
100195999	cxl10	C-X-C motif chemokine 10	21.40	-1.93	-3.81	0.00	0.00
106569469	LOC106569469	uncharacterized LOC106569469	50.13	-1.92	-3.80	0.00	0.01
106582536	LOC106582536	T-cell-specific surface glycoprotein CD28-like	6.73	-1.92	-3.79	0.00	0.02
106578584	LOC106578584	protein phosphatase 1 regulatory subunit 3G-lik	96.82	-1.91	-3.76	0.00	0.00
106598436	LOC106598436	tyrosine-protein kinase JAK1-like	31.69	-1.91	-3.75	0.00	0.00
106606746	LOC106606746	interferon-induced protein 44-like	27.79	-1.91	-3.75	0.00	0.03
106585202	LOC106585202	succinyl-CoA:3-ketoacid coenzyme A transferas	19.12	-1.90	-3.74	0.00	0.00
100136511	LOC100136511	CCL4-like chemokine	10.69	-1.89	-3.70	0.00	0.02
100195746	cd37	Leukocyte antigen CD37	30.51	-1.87	-3.67	0.00	0.01
106583450	LOC106583450	uncharacterized LOC106583450	161.02	-1.87	-3.66	0.00	0.00
106612297	LOC106612297	glutathione peroxidase 3-like	341.20	-1.87	-3.65	0.00	0.00
106579125	cd79b	CD79b molecule	40.01	-1.87	-3.65	0.00	0.00
106581089	LOC106581089	uncharacterized LOC106581089	7.75	-1.86	-3.62	0.00	0.00
106586891	LOC106586891	interferon-induced GTP-binding protein Mx2-lik	4.85	-1.85	-3.61	0.00	0.00
100196542	dapp1	dual adaptor of phosphotyrosine and 3-phosph	18.69	-1.85	-3.60	0.00	0.01
106604665	LOC106604665	large neutral amino acids transporter small sub	16.35	-1.85	-3.60	0.00	0.00
106578890	LOC106578890	snaclec 1-like	20.60	-1.85	-3.59	0.00	0.00
100195284	bcl6	B-cell lymphoma 6 protein homolog	33.04	-1.84	-3.58	0.00	0.00

106578012	LOC106578012	urokinase plasminogen activator surface recept	247.50	-1.84	-3.58	0.00	0.00
106582240	LOC106582240	interferon-induced guanylate-binding protein 1	80.01	-1.83	-3.55	0.00	0.00
106579758	LOC106579758	interferon-induced guanylate-binding protein 1	117.03	-1.82	-3.54	0.00	0.00
106602030	LOC106602030	toll-like receptor 13	4.97	-1.82	-3.53	0.00	0.01
100194861	lpp60	60 kDa lysophospholipase	139.05	-1.82	-3.53	0.00	0.00
106577663	LOC106577663	high affinity immunoglobulin epsilon receptor s	104.70	-1.82	-3.52	0.00	0.00
106572326	LOC106572326	chymotrypsin-like elastase family member 2A	8.41	-1.82	-3.52	0.00	0.01
106611750	LOC106611750	uncharacterized LOC106611750	23.33	-1.82	-3.52	0.00	0.00
106575134	LOC106575134	integrin alpha-4-like	21.83	-1.80	-3.49	0.00	0.03
100194690	cssa17h12orf56	chromosome ssa17 open reading frame, humar	5.49	-1.80	-3.48	0.00	0.01
106611413	LOC106611413	uncharacterized LOC106611413	44.63	-1.80	-3.47	0.00	0.02
106608766	LOC106608766	claudin-15-like	420.23	-1.79	-3.47	0.00	0.02
106590377	LOC106590377	NEDD8 ultimate buster 1-like	36.00	-1.79	-3.45	0.00	0.02
100137024	trp-iii	trypsin III	9.17	-1.79	-3.45	0.00	0.00
106610718	LOC106610718	zinc finger protein 395-like	24.38	-1.78	-3.44	0.00	0.00
106567650	LOC106567650	interleukin-31 receptor subunit alpha-like	7.30	-1.78	-3.43	0.00	0.00
106578240	LOC106578240	profilin-2-like	974.70	-1.78	-3.43	0.00	0.00
106583433	LOC106583433	interferon-induced protein 44-like	18.25	-1.77	-3.42	0.00	0.00
106571174	LOC106571174	leucine-rich alpha-2-glycoprotein-like	859.92	-1.77	-3.42	0.00	0.01
106568131	lhfpl2	lipoma HMGIC fusion partner-like 2	40.71	-1.77	-3.40	0.00	0.04
100195528	dnajc3	DnaJ heat shock protein family (Hsp40) membe	993.14	-1.77	-3.40	0.00	0.00
100169854	LOC100169854	uncharacterized LOC100169854	18.66	-1.76	-3.39	0.00	0.02
106563448	LOC106563448	spectrin beta chain, non-erythrocytic 1-like	6.95	-1.76	-3.39	0.00	0.03
100270810	ncf1	neutrophil cytosolic factor 1	15.50	-1.76	-3.38	0.00	0.00
106601234	LOC106601234	uncharacterized LOC106601234	9.51	-1.76	-3.38	0.00	0.01
106573350	LOC106573350	CD44 antigen-like	21.93	-1.76	-3.38	0.00	0.00
100195196	ebi2	EBV-induced G-protein coupled receptor 2	22.95	-1.74	-3.35	0.00	0.03
106611185	LOC106611185	uncharacterized LOC106611185	6.78	-1.73	-3.33	0.00	0.00
106604645	LOC106604645	CD97 antigen-like	44.32	-1.73	-3.32	0.00	0.02
100136501	LOC100136501	hemicentin-1	7.15	-1.73	-3.32	0.00	0.03
106588304	LOC106588304	Ig kappa chain V region Mem5-like	42.90	-1.73	-3.32	0.00	0.03
106589615	LOC106589615	filaggrin-2-like	32.97	-1.73	-3.31	0.00	0.00
106584369	LOC106584369	tyrosine-protein kinase ZAP-70-like	28.12	-1.73	-3.31	0.00	0.04
106567524	LOC106567524	transcription factor 7-like 2	16.72	-1.73	-3.31	0.00	0.00
100380666	LOC100380666	eosinophil peroxidase	9.17	-1.72	-3.31	0.00	0.03
106603325	LOC106603325	uncharacterized protein CXorf21 homolog	5.45	-1.71	-3.28	0.00	0.00
106563192	LOC106563192	proactivator polypeptide-like	207.65	-1.71	-3.27	0.00	0.05
106577197	LOC106577197	catechol O-methyltransferase domain-containir	210.06	-1.71	-3.27	0.00	0.00
106570315	LOC106570315	adseverin-like	12.41	-1.71	-3.26	0.00	0.00
100137023	trp-ii	trypsin II	18.14	-1.70	-3.26	0.00	0.00

106586893	LOC106586893	C-X-C motif chemokine 11-like	11.19	-1.69	-3.23	0.00	0.00
106579241	LOC106579241	uncharacterized LOC106579241	145.47	-1.69	-3.22	0.00	0.00
106606554	LOC106606554	poly [ADP-ribose] polymerase 14-like	22.30	-1.69	-3.22	0.00	0.00
106588087	LOC106588087	uncharacterized LOC106588087	565.93	-1.68	-3.21	0.00	0.00
106578023	LOC106578023	uncharacterized LOC106578023	11.97	-1.66	-3.17	0.00	0.02
100380857	LOC100380857	protein-arginine deiminase type-2	89.04	-1.66	-3.16	0.00	0.00
100195866	tryp	Trypsin	14.78	-1.66	-3.16	0.00	0.00
106608311	LOC106608311	SLC2A4 regulator-like	59.13	-1.66	-3.16	0.00	0.02
106573817	LOC106573817	UV excision repair protein RAD23 homolog B-lik	28.47	-1.65	-3.15	0.00	0.01
106573833	LOC106573833	sphingosine 1-phosphate receptor 4-like	9.47	-1.65	-3.14	0.00	0.00
100195610	plek	pleckstrin	39.74	-1.65	-3.14	0.00	0.00
106600856	LOC106600856	deleted in malignant brain tumors 1 protein-like	6.72	-1.65	-3.13	0.00	0.00
106606373	LOC106606373	uncharacterized LOC106606373	10.68	-1.65	-3.13	0.00	0.00
106602950	LOC106602950	NHS-like protein 2	8.68	-1.64	-3.12	0.00	0.01
106562007	LOC106562007	BCL2/adenovirus E1B 19 kDa protein-interacting	525.81	-1.64	-3.12	0.00	0.00
106588354	LOC106588354	tumor necrosis factor alpha-induced protein 8-l	22.97	-1.64	-3.12	0.00	0.01
106591918	LOC106591918	cartilage acidic protein 1-like	14.60	-1.64	-3.12	0.00	0.00
106566159	LOC106566159	elastase-1	8.77	-1.64	-3.12	0.00	0.00
106588135	LOC106588135	chymotrypsin A-like	9.25	-1.64	-3.12	0.00	0.00
100196613	grp78	78 kDa glucose-regulated protein	13865.09	-1.64	-3.11	0.00	0.00
106610842	LOC106610842	eukaryotic translation initiation factor 2-alpha k	66.86	-1.63	-3.11	0.00	0.00
106576242	LOC106576242	uncharacterized LOC106576242	6.35	-1.63	-3.10	0.00	0.02
106589652	LOC106589652	uncharacterized LOC106589652	5.96	-1.63	-3.09	0.00	0.02
100195489	ccr9	C-C chemokine receptor type 9	14.10	-1.63	-3.09	0.00	0.05
106588240	LOC106588240	free fatty acid receptor 2-like	8.51	-1.63	-3.09	0.00	0.01
100306785	itb2	Integrin beta-2	176.41	-1.62	-3.08	0.00	0.01
106611589	LOC106611589	leukocyte cell-derived chemotaxin-2-like	63.68	-1.62	-3.07	0.00	0.00
106580940	LOC106580940	tumor necrosis factor, alpha-induced protein 8-	22.71	-1.62	-3.06	0.00	0.00
106561182	LOC106561182	uncharacterized LOC106561182	411.85	-1.61	-3.06	0.00	0.01
106601901	LOC106601901	Ig lambda-2 chain C region-like	11.00	-1.61	-3.06	0.00	0.00
106560300	LOC106560300	regulator of G-protein signaling 1-like	13.50	-1.61	-3.05	0.00	0.01
106560599	LOC106560599	ectoderm-neural cortex protein 1-like	29.87	-1.61	-3.05	0.00	0.00
106565579	LOC106565579	uncharacterized LOC106565579	18.66	-1.61	-3.05	0.00	0.03
106602661	LOC106602661	E3 ISG15--protein ligase HERC5-like	15.98	-1.61	-3.05	0.00	0.03
106582243	LOC106582243	uncharacterized LOC106582243	21.43	-1.61	-3.05	0.00	0.00
106609426	LOC106609426	Ig kappa chain V-III region MOPC 321-like	8.97	-1.61	-3.04	0.00	0.00
106601861	LOC106601861	nuclear GTPase SLIP-GC-like	53.26	-1.60	-3.04	0.00	0.00
100194950	klhl6	kelch like family member 6	39.60	-1.60	-3.04	0.00	0.01
100195230	rnd3	Rho-related GTP-binding protein RhoE	26.14	-1.60	-3.03	0.00	0.01
106602649	LOC106602649	T-cell surface glycoprotein CD5-like	9.31	-1.60	-3.03	0.00	0.00

106587845	LOC106587845	major histocompatibility complex class I-relatec	24.93	-1.60	-3.02	0.00	0.00
106560483	LOC106560483	dedicator of cytokinesis protein 7-like	13.04	-1.59	-3.02	0.00	0.02
106566501	LOC106566501	uncharacterized LOC106566501	35.54	-1.59	-3.01	0.00	0.02
106564531	LOC106564531	GATA zinc finger domain-containing protein 10-	98.55	-1.59	-3.01	0.00	0.03
106606048	LOC106606048	uncharacterized LOC106606048	33.62	-1.59	-3.01	0.00	0.00
106568165	LOC106568165	interleukin-7 receptor subunit alpha-like	19.34	-1.58	-3.00	0.00	0.00
106599105	arg2	arginase 2	36.55	-1.58	-2.99	0.00	0.00
100195790	cor1a	Coronin-1A	377.13	-1.58	-2.99	0.00	0.00
106606762	LOC106606762	Ig mu chain C region membrane-bound form-lik	11.68	-1.58	-2.98	0.00	0.00
106571205	tagap	NA	26.11	-1.58	-2.98	0.00	0.00
106613072	LOC106613072	serpin H1-like	24.97	-1.57	-2.97	0.00	0.00
100196216	mfap4	microfibrillar-associated protein 4	296.57	-1.57	-2.96	0.00	0.00
106575562	LOC106575562	T-cell surface antigen CD2-like	12.91	-1.56	-2.95	0.00	0.00
100217352	i14k	14 kDa transmembrane protein	11.23	-1.56	-2.95	0.00	0.01
106601734	vwa3a	NA	25.58	-1.56	-2.95	0.00	0.01
106601675	LOC106601675	uncharacterized LOC106601675	19.25	-1.56	-2.94	0.00	0.00
100380871	il31r	Interleukin-31 receptor A	17.23	-1.56	-2.94	0.00	0.00
106573818	LOC106573818	calreticulin-like	8919.45	-1.55	-2.94	0.00	0.00
106570622	LOC106570622	CD5 antigen-like	10423.46	-1.55	-2.94	0.00	0.00
106582238	LOC106582238	interferon-induced guanylate-binding protein 1	22.90	-1.55	-2.93	0.00	0.01
106571437	LOC106571437	uncharacterized LOC106571437	11.04	-1.55	-2.93	0.00	0.01
106605364	LOC106605364	CD83 antigen-like	14.50	-1.55	-2.92	0.00	0.01
106568734	LOC106568734	regulator of G-protein signaling 21-like	11.85	-1.54	-2.92	0.00	0.00
106569780	LOC106569780	T-cell differentiation antigen CD6-like	37.10	-1.54	-2.91	0.00	0.01
106582198	LOC106582198	guanylate-binding protein 5-like	6.78	-1.54	-2.91	0.00	0.00
106606886	LOC106606886	coronin-1A-like	247.69	-1.54	-2.91	0.00	0.03
106607353	LOC106607353	3-phosphoinositide-dependent protein kinase 1	19.87	-1.54	-2.91	0.00	0.00
106602643	LOC106602643	lectin-like	10.20	-1.54	-2.91	0.00	0.00
106609487	LOC106609487	Ig kappa chain C region, B allele-like	15.41	-1.54	-2.91	0.00	0.03
106561308	LOC106561308	CD44 antigen-like	32.22	-1.54	-2.91	0.00	0.00
106579620	LOC106579620	myeloid cell surface antigen CD33-like	24.52	-1.54	-2.90	0.00	0.00
100196207	tnr5	Tumor necrosis factor receptor superfamily mer	90.42	-1.54	-2.90	0.00	0.02
106589909	LOC106589909	ribosome-binding protein 1-like	754.45	-1.54	-2.90	0.00	0.01
106566106	LOC106566106	mitogen-activated protein kinase 14A-like	12.18	-1.53	-2.90	0.00	0.00
106581641	itgbl1	integrin subunit beta like 1	5.34	-1.53	-2.89	0.00	0.00
106575700	LOC106575700	uncharacterized LOC106575700	34.05	-1.53	-2.89	0.00	0.04
106577430	LOC106577430	trypsin-2-like	9.98	-1.53	-2.89	0.00	0.00
106601045	LOC106601045	histone-lysine N-methyltransferase EZH1-like	35.51	-1.53	-2.88	0.00	0.00
106588235	LOC106588235	GTP-binding protein Rhes pseudogene	19.93	-1.53	-2.88	0.00	0.04
106572986	LOC106572986	secretagogin-like	12.64	-1.53	-2.88	0.00	0.00

106608396	LOC106608396	profilin-2 pseudogene	151.14	-1.53	-2.88	0.00	0.00
106574583	LOC106574583	Ig kappa chain V-III region MOPC 63-like	8.52	-1.52	-2.88	0.00	0.00
100195203	lcp2	lymphocyte cytosolic protein 2	26.93	-1.52	-2.87	0.00	0.00
106611874	LOC106611874	macrophage colony-stimulating factor 1 recepto	138.99	-1.52	-2.87	0.00	0.00
106587891	LOC106587891	poly [ADP-ribose] polymerase 14-like	5.59	-1.52	-2.87	0.00	0.00
106564353	LOC106564353	antimicrobial peptide NK-lysin-like	6.48	-1.52	-2.87	0.00	0.01
106607975	LOC106607975	interleukin-12 subunit beta-like	7.43	-1.52	-2.86	0.00	0.00
106600810	LOC106600810	perforin-1-like	48.72	-1.52	-2.86	0.00	0.00
106608841	LOC106608841	probable E3 ubiquitin-protein ligase RNF144A-A	6.85	-1.52	-2.86	0.00	0.00
106606997	LOC106606997	SEC14-like protein 1	171.68	-1.52	-2.86	0.00	0.01
106587550	LOC106587550	switch-associated protein 70-like	30.59	-1.51	-2.86	0.00	0.00
106564565	LOC106564565	probable ATP-dependent RNA helicase DDX17	136.01	-1.51	-2.86	0.00	0.03
106600855	LOC106600855	uncharacterized LOC106600855	12.17	-1.51	-2.85	0.00	0.01
106561547	LOC106561547	high choriolytic enzyme 2-like	7.63	-1.51	-2.85	0.00	0.00
106588482	LOC106588482	C-type lectin domain family 4 member E-like	18.02	-1.51	-2.84	0.00	0.00
106578961	LOC106578961	actin, aortic smooth muscle	19.22	-1.51	-2.84	0.00	0.01
106586691	LOC106586691	cystathionine beta-synthase-like	191.42	-1.51	-2.84	0.00	0.00
106577735	LOC106577735	major centromere autoantigen B-like	65.91	-1.50	-2.84	0.00	0.00
106565863	LOC106565863	ankyrin repeat and SAM domain-containing pro	28.19	-1.50	-2.83	0.00	0.00
106607455	LOC106607455	integrin alpha-IIb-like	10.66	-1.50	-2.82	0.00	0.00
106604553	LOC106604553	SAM and SH3 domain-containing protein 3-like	31.91	-1.50	-2.82	0.00	0.01
106575526	LOC106575526	P2Y purinoceptor 8-like	7.17	-1.50	-2.82	0.00	0.00
100195718	tp8l2	Tumor necrosis factor, alpha-induced protein 8-	36.79	-1.49	-2.82	0.00	0.00
106585699	LOC106585699	alpha-1-antitrypsin-like	11658.59	-1.49	-2.82	0.00	0.00
106580864	LOC106580864	cathepsin K-like	199.88	-1.49	-2.81	0.00	0.02
106608695	LOC106608695	uncharacterized LOC106608695	22.05	-1.49	-2.80	0.00	0.00
106597311	LOC106597311	C-C chemokine receptor type 9-like	7.83	-1.48	-2.80	0.00	0.03
106571177	LOC106571177	protein disulfide-isomerase A6-like	4311.04	-1.48	-2.79	0.00	0.01
106572163	LOC106572163	chymotrypsin-like elastase family member 2A	7.75	-1.48	-2.79	0.00	0.01
106582432	LOC106582432	trace amine-associated receptor 13c-like	23.43	-1.48	-2.79	0.00	0.01
106564129	LOC106564129	uncharacterized LOC106564129	110.34	-1.48	-2.78	0.00	0.02
106583087	LOC106583087	complement C3-like	567.71	-1.48	-2.78	0.00	0.03
106565782	LOC106565782	granzyme K-like	18.18	-1.48	-2.78	0.00	0.00
106574569	LOC106574569	Ig kappa chain C region-like	11.06	-1.47	-2.78	0.00	0.00
106582242	LOC106582242	interferon-induced guanylate-binding protein 1	30.44	-1.47	-2.78	0.00	0.04
106588086	LOC106588086	integrin alpha-L-like	30.79	-1.47	-2.78	0.00	0.00
106582662	LOC106582662	uncharacterized LOC106582662	21.70	-1.47	-2.78	0.00	0.00
106589292	LOC106589292	eomesodermin-like	11.20	-1.47	-2.78	0.00	0.02
106588252	LOC106588252	uncharacterized LOC106588252	4.64	-1.47	-2.77	0.00	0.00
106587939	LOC106587939	C-C motif chemokine 13-like	4.37	-1.47	-2.77	0.00	0.02

106561918	LOC106561918	neuron navigator 2-like	88.23	-1.47	-2.77	0.00	0.04
100194971	aqp1	Aquaporin-1	57.96	-1.47	-2.77	0.00	0.01
106599020	LOC106599020	transcription factor jun-D-like	28.40	-1.47	-2.77	0.00	0.00
106601960	LOC106601960	rho GDP-dissociation inhibitor 2-like	69.69	-1.47	-2.76	0.00	0.00
106569300	LOC106569300	uncharacterized LOC106569300	121.48	-1.46	-2.76	0.00	0.02
106560976	LOC106560976	overexpressed in colon carcinoma 1 protein hor	12.07	-1.46	-2.76	0.00	0.01
106613685	LOC106613685	calreticulin-like	4026.81	-1.46	-2.76	0.00	0.00
106585402	LOC106585402	adhesion G protein-coupled receptor E2-like	7.88	-1.46	-2.76	0.00	0.00
106609551	LOC106609551	Ig kappa chain V-III region MOPC 63-like	8.53	-1.46	-2.76	0.00	0.00
106568336	LOC106568336	soluble lamin-associated protein of 75 kDa-like	5.23	-1.46	-2.75	0.00	0.00
106596062	LOC106596062	chemokine XC receptor 1-like	4.33	-1.46	-2.75	0.00	0.00
106584635	LOC106584635	UPF0676 protein C1494.01-like	38.60	-1.46	-2.75	0.00	0.01
100196194	scyb7	Platelet basic protein	13.44	-1.46	-2.75	0.00	0.00
106601717	LOC106601717	Ig mu chain C region membrane-bound form-lik	13.24	-1.46	-2.75	0.00	0.00
106566437	LOC106566437	cytochrome c oxidase subunit 4 isoform 2, mito	915.71	-1.46	-2.74	0.00	0.00
100196325	r1441	Probable E3 ubiquitin-protein ligase RNF144A-A	16.83	-1.45	-2.74	0.00	0.02
106589414	LOC106589414	inactive ubiquitin carboxyl-terminal hydrolase 5	17.58	-1.45	-2.73	0.00	0.00
106607844	LOC106607844	uncharacterized LOC106607844	14.44	-1.45	-2.73	0.00	0.03
106564788	srsf7	NA	1391.58	-1.45	-2.72	0.00	0.00
106612585	LOC106612585	uncharacterized LOC106612585	16.91	-1.45	-2.72	0.00	0.01
106579322	LOC106579322	uncharacterized LOC106579322	26.07	-1.44	-2.72	0.00	0.00
106613695	LOC106613695	lipoprotein lipase-like	876.88	-1.44	-2.72	0.00	0.00
106602940	LOC106602940	mitogen-activated protein kinase kinase kinase	20.32	-1.44	-2.72	0.00	0.00
100195808	nattl	Natterin-like protein	6.90	-1.44	-2.71	0.00	0.02
106586830	LOC106586830	poly [ADP-ribose] polymerase 14-like	26.82	-1.44	-2.71	0.00	0.01
106567153	LOC106567153	uncharacterized LOC106567153	5.49	-1.44	-2.71	0.00	0.00
106590297	LOC106590297	ras-related C3 botulinum toxin substrate 2-like	41.90	-1.44	-2.71	0.00	0.00
106609846	LOC106609846	beta-2-microglobulin-like	157.48	-1.44	-2.71	0.00	0.00
106588950	LOC106588950	granulins-like	66.68	-1.43	-2.70	0.00	0.05
106608776	LOC106608776	E3 ubiquitin-protein ligase KEG-like	30.25	-1.43	-2.70	0.00	0.01
106571175	LOC106571175	PQ-loop repeat-containing protein 3-like	36.49	-1.43	-2.70	0.00	0.03
106564435	LOC106564435	ras-related C3 botulinum toxin substrate 2-like	25.73	-1.43	-2.69	0.00	0.00
100306791	i12r2	Interleukin-12 receptor beta-2 chain	21.57	-1.43	-2.69	0.00	0.05
106582258	LOC106582258	interleukin-1 receptor type 1-like	10.81	-1.43	-2.69	0.00	0.00
106567149	LOC106567149	78 kDa glucose-regulated protein-like	491.14	-1.43	-2.69	0.00	0.04
106598999	LOC106598999	uncharacterized LOC106598999	28.41	-1.43	-2.69	0.00	0.00
106568312	LOC106568312	membrane-associated phosphatidylinositol trar	9.07	-1.43	-2.69	0.00	0.00
106609985	LOC106609985	uncharacterized LOC106609985	67.26	-1.43	-2.69	0.00	0.02
100195547	cd53	Leukocyte surface antigen CD53	134.96	-1.42	-2.68	0.00	0.00
100195371	ifi44	Interferon-induced protein 44	15.37	-1.42	-2.68	0.00	0.00

106611811	LOC106611811	dedicator of cytokinesis protein 2-like	46.34	-1.42	-2.68	0.00	0.00
106608837	LOC106608837	uncharacterized protein DDB_G0290685-like	110.24	-1.42	-2.68	0.00	0.01
106601033	LOC106601033	complement C1q-like protein 2	711.62	-1.42	-2.68	0.00	0.01
106572285	LOC106572285	tyrosine-protein phosphatase non-receptor typ	24.92	-1.42	-2.67	0.00	0.02
106566613	LOC106566613	uncharacterized LOC106566613	24.40	-1.41	-2.66	0.00	0.00
100195046	pou2af1	POU class 2 associating factor 1	8.58	-1.41	-2.66	0.00	0.00
106565963	LOC106565963	uncharacterized LOC106565963	19.66	-1.41	-2.66	0.00	0.00
100194803	lox5	Arachidonate 5-lipoxygenase	19.60	-1.41	-2.65	0.00	0.00
106597258	LOC106597258	E3 ubiquitin-protein ligase RNF213-like	4.47	-1.41	-2.65	0.00	0.01
106584134	LOC106584134	leucine-rich repeat-containing protein 16B-like	20.99	-1.41	-2.65	0.00	0.01
106572799	LOC106572799	differentially expressed in FDCP 6 homolog	32.42	-1.40	-2.65	0.00	0.02
106570194	LOC106570194	uncharacterized LOC106570194	29.68	-1.40	-2.65	0.00	0.00
106613845	LOC106613845	tyrosine-protein kinase ZAP-70-like	9.47	-1.40	-2.64	0.00	0.00
106581970	LOC106581970	T-cell surface glycoprotein CD3 zeta chain-like	8.67	-1.40	-2.64	0.00	0.00
106580597	LOC106580597	cyclic AMP-responsive element-binding protein	10.61	-1.40	-2.64	0.00	0.00
106564583	LOC106564583	B-cell lymphoma/leukemia 11A pseudogene	7.76	-1.40	-2.64	0.00	0.05
106609562	LOC106609562	cytoskeleton-associated protein 4-like	204.95	-1.40	-2.63	0.00	0.04
106562355	LOC106562355	switch-associated protein 70-like	11.34	-1.40	-2.63	0.00	0.02
106583577	LOC106583577	Fc receptor-like protein 5	12.50	-1.39	-2.63	0.00	0.01
106571232	LOC106571232	TATA box-binding protein-associated factor RN/	20.63	-1.39	-2.63	0.00	0.00
106588193	LOC106588193	ATPase inhibitor A, mitochondrial-like	251.43	-1.39	-2.63	0.00	0.03
106612193	LOC106612193	regulator of cell cycle RGCC-like	39.23	-1.39	-2.63	0.00	0.00
106566589	lime1	Lck interacting transmembrane adaptor 1	15.87	-1.39	-2.62	0.00	0.00
100195597	atrap	Type-1 angiotensin II receptor-associated prote	5.17	-1.39	-2.62	0.00	0.00
106566092	LOC106566092	uncharacterized LOC106566092	650.26	-1.39	-2.62	0.00	0.04
106613147	LOC106613147	von Willebrand factor A domain-containing pro	10.77	-1.39	-2.62	0.00	0.00
106564218	LOC106564218	NACHT, LRR and PYD domains-containing prote	165.69	-1.39	-2.62	0.00	0.00
100136365	LOC100136365	lysozyme C II	260.71	-1.39	-2.62	0.00	0.00
100136924	tap2b	NA	29.86	-1.39	-2.62	0.00	0.01
106605366	LOC106605366	CD83 antigen-like	28.39	-1.39	-2.62	0.00	0.01
106583653	LOC106583653	E3 ubiquitin-protein ligase RNF213-like	11.61	-1.39	-2.61	0.00	0.00
106587957	LOC106587957	uncharacterized LOC106587957	10.08	-1.38	-2.61	0.00	0.05
106579830	LOC106579830	triple functional domain protein-like	16.01	-1.38	-2.61	0.00	0.00
106566091	LOC106566091	major histocompatibility complex class I-relatec	103.90	-1.38	-2.61	0.00	0.00
106589454	LOC106589454	probable ATP-dependent RNA helicase DDX17	46.76	-1.38	-2.61	0.00	0.01
106604374	LOC106604374	girdin-like	36.45	-1.38	-2.61	0.00	0.00
106613835	LOC106613835	leupaxin-like	32.93	-1.38	-2.61	0.00	0.01
106607598	LOC106607598	NF-kappa-B inhibitor alpha-like	116.85	-1.38	-2.61	0.00	0.05
106608104	LOC106608104	uncharacterized LOC106608104	5.04	-1.38	-2.60	0.00	0.00
106577579	LOC106577579	protein NLRC3-like	59.38	-1.38	-2.60	0.00	0.00

106589387	LOC106589387	actin, aortic smooth muscle	38.43	-1.38	-2.60	0.00	0.01
106581806	tbr1	T-box, brain 1	11.16	-1.38	-2.60	0.00	0.00
106566176	LOC106566176	sperm-specific antigen 2 homolog	9.67	-1.38	-2.60	0.00	0.00
106588197	LOC106588197	eomesodermin-like	8.31	-1.38	-2.60	0.00	0.00
100141317	fxyd5a	FXYD domain containing ion transport regulator	31.63	-1.38	-2.59	0.00	0.00
106610861	LOC106610861	phospholipase D4-like	23.03	-1.37	-2.59	0.00	0.00
106603814	LOC106603814	transcription factor 7-like 2	9.69	-1.37	-2.59	0.00	0.02
106586330	LOC106586330	rho GTPase-activating protein 15-like	44.24	-1.37	-2.59	0.00	0.00
106570689	LOC106570689	MARCKS-related protein-like	172.00	-1.37	-2.59	0.00	0.03
106610680	LOC106610680	SH3 and cysteine-rich domain-containing protei	51.27	-1.37	-2.59	0.00	0.00
106563105	LOC106563105	complement C1q-like protein 2	10.39	-1.37	-2.59	0.00	0.00
106567873	LOC106567873	asialoglycoprotein receptor 1-like	4.57	-1.37	-2.59	0.00	0.02
106577279	LOC106577279	interferon-induced very large GTPase 1-like	46.02	-1.37	-2.59	0.00	0.00
106564314	LOC106564314	transmembrane channel-like protein 8	28.15	-1.37	-2.58	0.00	0.01
106573182	LOC106573182	protein bicaudal D homolog 1-like	46.38	-1.36	-2.57	0.00	0.00
106560983	parvb	parvin beta	20.37	-1.36	-2.57	0.00	0.00
106565098	LOC106565098	uncharacterized LOC106565098	40.85	-1.36	-2.57	0.00	0.00
106575215	LOC106575215	glutaminase kidney isoform, mitochondrial-like	6.58	-1.36	-2.57	0.00	0.00
106562560	LOC106562560	serine/threonine-protein kinase/endoribonucle	90.62	-1.36	-2.57	0.00	0.02
106573648	LOC106573648	olfactomedin-4-like	83.23	-1.36	-2.57	0.00	0.00
106599011	LOC106599011	gamma-interferon-inducible lysosomal thiol rec	92.58	-1.36	-2.57	0.00	0.03
106589283	LOC106589283	src kinase-associated phosphoprotein 1-like	7.26	-1.36	-2.57	0.00	0.00
106605412	LOC106605412	C-C chemokine receptor type 5-like	6.93	-1.36	-2.56	0.00	0.04
106583912	LOC106583912	mucin-2-like	31.36	-1.36	-2.56	0.00	0.01
100196098	p2ry5	P2Y purinoceptor 5	4.39	-1.36	-2.56	0.00	0.00
106563456	LOC106563456	fermitin family homolog 3-like	53.82	-1.36	-2.56	0.00	0.01
106604605	LOC106604605	adhesion G protein-coupled receptor E3-like	34.29	-1.35	-2.56	0.00	0.00
106578013	LOC106578013	leukotriene B4 receptor 1-like	4.98	-1.35	-2.55	0.00	0.03
106579929	LOC106579929	4-hydroxyphenylpyruvate dioxygenase-like	10.86	-1.35	-2.55	0.00	0.01
106613930	LOC106613930	calcium-activated potassium channel subunit be	195.76	-1.35	-2.55	0.00	0.00
106589322	LOC106589322	delta-1-pyrroline-5-carboxylate synthase-like	22.39	-1.35	-2.55	0.00	0.04
106603693	LOC106603693	protein-tyrosine kinase 2-beta-like	28.25	-1.35	-2.55	0.00	0.00
106602382	LOC106602382	15-hydroxyprostaglandin dehydrogenase [NAD(	321.03	-1.35	-2.55	0.00	0.01
106586483	lcp1	lymphocyte cytosolic protein 1	376.03	-1.35	-2.55	0.00	0.01
106607816	LOC106607816	clusterin-like	39.76	-1.35	-2.55	0.00	0.01
106598820	b3gnt5	UDP-GlcNAc:betaGal beta-1,3-N-acetylglucosan	8.04	-1.35	-2.55	0.00	0.02
106575674	LOC106575674	cytochrome b-245 heavy chain-like	23.94	-1.35	-2.54	0.00	0.00
106605277	LOC106605277	G-protein-signaling modulator 2-like	64.89	-1.35	-2.54	0.00	0.01
106582237	LOC106582237	interferon-induced guanylate-binding protein 1	77.73	-1.34	-2.53	0.00	0.00
106604703	LOC106604703	sestrin-3-like	44.72	-1.34	-2.53	0.00	0.00

106608874	LOC106608874	trichohyalin-like	19.16	-1.34	-2.53	0.00	0.03
106573758	LOC106573758	choline kinase alpha-like	381.78	-1.34	-2.52	0.00	0.00
106570620	LOC106570620	receptor-transporting protein 3-like	104.56	-1.34	-2.52	0.00	0.01
106588637	LOC106588637	palmitoyltransferase ZDHHC18-like	17.18	-1.34	-2.52	0.00	0.00
106588401	LOC106588401	class I histocompatibility antigen, F10 alpha cha	2663.54	-1.33	-2.51	0.00	0.04
106613068	LOC106613068	uncharacterized LOC106613068	10.69	-1.33	-2.51	0.00	0.05
106571891	LOC106571891	uncharacterized LOC106571891	9.69	-1.33	-2.51	0.00	0.00
106605554	LOC106605554	C-X-C chemokine receptor type 3-like	28.94	-1.33	-2.51	0.00	0.00
106586296	LOC106586296	uncharacterized LOC106586296	8.58	-1.33	-2.51	0.00	0.00
106560831	LOC106560831	arachidonate 15-lipoxygenase B-like	52.62	-1.32	-2.51	0.00	0.02
106586221	dcaf17	DDB1 and CUL4 associated factor 17	45.14	-1.32	-2.50	0.00	0.04
106569959	LOC106569959	proteasome subunit beta type-6-B like protein	45.60	-1.32	-2.50	0.00	0.00
106609574	LOC106609574	pollen-specific leucine-rich repeat extensin-like	8.91	-1.32	-2.50	0.00	0.00
106605178	LOC106605178	cornifelin-like	26.34	-1.32	-2.50	0.00	0.04
106603097	LOC106603097	voltage-gated potassium channel subunit beta-:	61.55	-1.32	-2.50	0.00	0.00
106575540	LOC106575540	T-cell surface antigen CD2-like	17.64	-1.32	-2.50	0.00	0.03
106561496	LOC106561496	AMP deaminase 3-like	16.13	-1.32	-2.50	0.00	0.00
106587975	LOC106587975	C-C motif chemokine 13-like	7.57	-1.32	-2.49	0.00	0.04
106609751	LOC106609751	tetraspanin-8-like	65.65	-1.32	-2.49	0.00	0.00
106561493	LOC106561493	neuron navigator 2-like	163.00	-1.32	-2.49	0.00	0.02
106607854	LOC106607854	uncharacterized LOC106607854	114.81	-1.31	-2.49	0.00	0.00
106613687	LOC106613687	mucosa-associated lymphoid tissue lymphoma	26.21	-1.31	-2.49	0.00	0.00
106601467	LOC106601467	ras-related C3 botulinum toxin substrate 2	50.82	-1.31	-2.48	0.00	0.00
106606254	LOC106606254	phosphatidylinositol N-acetylglucosaminyltrans	7.32	-1.31	-2.48	0.00	0.00
106581964	LOC106581964	G-protein-signaling modulator 1-like	40.01	-1.31	-2.48	0.00	0.00
106606764	LOC106606764	uncharacterized LOC106606764	13.60	-1.31	-2.48	0.00	0.00
106581291	LOC106581291	CD83 antigen-like	15.58	-1.31	-2.48	0.00	0.02
106607313	LOC106607313	histone-lysine N-methyltransferase EZH1-like	43.48	-1.31	-2.48	0.00	0.00
106576234	LOC106576234	endoplasmin-like	4.59	-1.31	-2.47	0.00	0.03
100196425	xaf1	XIAP-associated factor 1	10.70	-1.31	-2.47	0.00	0.00
106601466	LOC106601466	ras-related C3 botulinum toxin substrate 2-like	119.28	-1.31	-2.47	0.00	0.00
100194852	grp2	RAS guanyl-releasing protein 2	44.45	-1.30	-2.47	0.00	0.00
106570607	LOC106570607	E3 ubiquitin-protein ligase ZNRF2-like	21.55	-1.30	-2.47	0.00	0.00
106569063	LOC106569063	uncharacterized LOC106569063	19.24	-1.30	-2.47	0.00	0.01
106576030	LOC106576030	uncharacterized LOC106576030	48.29	-1.30	-2.47	0.00	0.00
106587061	LOC106587061	ankyrin repeat and BTB/POZ domain-containing	20.32	-1.30	-2.46	0.00	0.00
106581647	LOC106581647	non-syndromic hearing impairment protein 5 h	9.39	-1.30	-2.46	0.00	0.01
100196737	tri39	Tripartite motif-containing protein 39	57.45	-1.30	-2.46	0.00	0.02
100196506	glipr1	GLI pathogenesis related 1	20.95	-1.30	-2.46	0.00	0.00
106581250	LOC106581250	kinase suppressor of Ras 1-like	178.20	-1.30	-2.46	0.00	0.02

106602581	LOC106602581	lectin-like	20.58	-1.30	-2.46	0.00	0.00
106575093	LOC106575093	T-cell-specific surface glycoprotein CD28-like	18.86	-1.30	-2.45	0.00	0.00
106576384	LOC106576384	Ig kappa-b4 chain C region-like	13.45	-1.29	-2.45	0.00	0.00
100195359	tlr13	Toll-like receptor 13	19.89	-1.29	-2.45	0.00	0.00
106588402	LOC106588402	major histocompatibility complex class I-relatec	364.83	-1.29	-2.45	0.00	0.02
106602706	LOC106602706	uncharacterized LOC106602706	34.52	-1.29	-2.45	0.00	0.01
100196705	bpi	Bactericidal permeability-increasing protein	231.76	-1.29	-2.45	0.00	0.04
106561706	LOC106561706	endoplasmin-like	14181.48	-1.29	-2.44	0.00	0.03
100195708	bin2	Bridging integrator 2	14.36	-1.29	-2.44	0.00	0.00
106604970	LOC106604970	protein-tyrosine kinase 2-beta-like	38.73	-1.29	-2.44	0.00	0.03
106605333	LOC106605333	tumor necrosis factor alpha-induced protein 8-l	24.64	-1.29	-2.44	0.00	0.00
106592054	LOC106592054	cartilage acidic protein 1-like	20.63	-1.29	-2.44	0.00	0.03
106611902	LOC106611902	serine/threonine-protein phosphatase 2A 55 kD	432.79	-1.28	-2.43	0.00	0.01
106575703	LOC106575703	uncharacterized LOC106575703	111.40	-1.28	-2.43	0.00	0.03
100306752	copt2	Probable low affinity copper uptake protein 2	40.39	-1.28	-2.43	0.00	0.00
106610446	LOC106610446	C-type lectin domain family 4 member E-like	37.53	-1.28	-2.43	0.00	0.00
106573870	jak3	Janus kinase 3	14.04	-1.28	-2.43	0.00	0.02
106603875	LOC106603875	CREB3 regulatory factor-like	168.45	-1.28	-2.43	0.00	0.00
106590411	LOC106590411	interferon regulatory factor 8-like	98.28	-1.28	-2.42	0.00	0.00
106604359	LOC106604359	bifunctional epoxide hydrolase 2-like	99.20	-1.28	-2.42	0.00	0.00
106589316	LOC106589316	uncharacterized LOC106589316	17.84	-1.28	-2.42	0.00	0.00
100194856	cssa21hxorf36	chromosome ssa21 open reading frame, humar	14.95	-1.28	-2.42	0.00	0.00
106598734	LOC106598734	eukaryotic initiation factor 4A-II-like	373.53	-1.27	-2.41	0.00	0.00
106610660	LOC106610660	ribosomal protein S6 kinase 2 alpha-like	47.88	-1.27	-2.41	0.00	0.00
106566739	psma7	proteasome subunit alpha 7	128.35	-1.27	-2.41	0.00	0.00
106569939	LOC106569939	proteasome subunit beta type-7-like	119.91	-1.27	-2.41	0.00	0.03
106582276	LOC106582276	alkylglycerol monooxygenase-like	467.21	-1.27	-2.41	0.00	0.02
106564155	LOC106564155	proto-oncogene vav-like	17.99	-1.27	-2.41	0.00	0.01
106585784	LOC106585784	TNF receptor-associated factor 1-like	10.73	-1.27	-2.41	0.00	0.00
106568428	LOC106568428	SH2 domain-containing protein 3C-like	11.44	-1.27	-2.40	0.00	0.00
106581262	LOC106581262	uncharacterized LOC106581262	37.05	-1.27	-2.40	0.00	0.00
106581285	LOC106581285	lysophosphatidic acid receptor 4-like	8.31	-1.26	-2.40	0.00	0.00
106562414	LOC106562414	solute carrier family 12 member 4-like	115.80	-1.26	-2.40	0.00	0.03
106601621	LOC106601621	B-cell receptor CD22-like	11.88	-1.26	-2.39	0.00	0.02
106571305	LOC106571305	ribonuclease ZC3H12A-like	29.11	-1.26	-2.39	0.00	0.00
106567717	LOC106567717	linker for activation of T-cells family member 2-	9.37	-1.25	-2.39	0.00	0.03
106581934	LOC106581934	protein FAM171B-like	37.74	-1.25	-2.39	0.00	0.00
106580893	map4k1	mitogen-activated protein kinase kinase kinase	38.92	-1.25	-2.39	0.00	0.02
106560507	LOC106560507	uncharacterized LOC106560507	4044.44	-1.25	-2.38	0.00	0.03
100194875	mfsd2	Major facilitator superfamily domain-containing	21.99	-1.25	-2.38	0.00	0.02

106589669	LOC106589669	uncharacterized LOC106589669	56.92	-1.25	-2.38	0.00	0.05
100196282	rgs18	regulator of G-protein signaling 18	17.73	-1.25	-2.38	0.00	0.00
106600906	LOC106600906	peroxisomal membrane protein PMP34-like	27.43	-1.25	-2.38	0.00	0.01
106570867	LOC106570867	stromal cell-derived factor 2-like	289.37	-1.25	-2.38	0.00	0.00
106585781	slc2a6	NA	17.69	-1.25	-2.38	0.00	0.01
106609907	LOC106609907	large neutral amino acids transporter small sub	34.99	-1.25	-2.37	0.00	0.00
106601068	LOC106601068	uncharacterized LOC106601068	12.16	-1.24	-2.37	0.00	0.00
106612986	LOC106612986	tumor necrosis factor receptor superfamily mer	43.38	-1.24	-2.37	0.00	0.00
100380715	relb	NA	180.06	-1.24	-2.37	0.00	0.00
106590395	LOC106590395	uncharacterized LOC106590395	17.35	-1.24	-2.36	0.00	0.02
106576109	LOC106576109	transmembrane protein 229A-like	32.73	-1.24	-2.36	0.00	0.00
100136516	cd3e	CD3e molecule	52.73	-1.24	-2.36	0.00	0.00
106608238	LOC106608238	cholesterol 24-hydroxylase-like	34.56	-1.24	-2.36	0.00	0.03
106612640	LOC106612640	ribosomal protein S6 kinase alpha-1	46.44	-1.24	-2.36	0.00	0.00
106566105	LOC106566105	mitogen-activated protein kinase 14A-like	124.18	-1.24	-2.36	0.00	0.00
106569062	LOC106569062	uncharacterized LOC106569062	43.52	-1.24	-2.36	0.00	0.01
106564830	LOC106564830	complement C3-like	23316.21	-1.24	-2.36	0.00	0.03
106564720	LOC106564720	C-X-C motif chemokine 11-like	18.84	-1.24	-2.36	0.00	0.03
106565699	LOC106565699	H-2 class II histocompatibility antigen, A-U alph	999.17	-1.23	-2.35	0.00	0.02
106606767	LOC106606767	uncharacterized LOC106606767	17.63	-1.23	-2.35	0.00	0.00
100286706	mrp	MARCKS-related protein	25.47	-1.23	-2.35	0.00	0.00
106582249	LOC106582249	plastin-2	312.25	-1.23	-2.34	0.00	0.01
106586142	LOC106586142	signal transducer and activator of transcription	41.38	-1.23	-2.34	0.00	0.00
106577850	LOC106577850	SLAM family member 8-like	10.29	-1.23	-2.34	0.00	0.00
106581080	LOC106581080	C5a anaphylatoxin chemotactic receptor 1	21.89	-1.22	-2.34	0.00	0.00
100194666	LOC100194666	BCL2/adenovirus E1B 19 kDa protein-interacting	969.41	-1.22	-2.33	0.00	0.04
106563251	LOC106563251	histone-lysine N-methyltransferase Smyd1-like	27.83	-1.22	-2.33	0.00	0.00
106585856	tle4	NA	18.40	-1.22	-2.33	0.00	0.00
106581002	LOC106581002	complement C4-B-like	78.59	-1.22	-2.32	0.00	0.03
106567447	LOC106567447	CREB3 regulatory factor-like	44.43	-1.21	-2.32	0.00	0.05
106603793	LOC106603793	dapper homolog 3-like	45.99	-1.21	-2.32	0.00	0.00
106563231	LOC106563231	gelsolin-like	89.53	-1.21	-2.31	0.00	0.00
106585067	LOC106585067	uncharacterized LOC106585067	49.29	-1.21	-2.31	0.00	0.02
100380694	ptprc	NA	487.00	-1.20	-2.31	0.00	0.00
106577993	LOC106577993	uncharacterized LOC106577993	18.10	-1.20	-2.30	0.00	0.00
106613694	LOC106613694	lipoprotein lipase-like	30.87	-1.20	-2.30	0.00	0.02
106609366	LOC106609366	BTB/POZ domain-containing protein KCTD5-like	213.61	-1.20	-2.30	0.00	0.01
100195008	pdcd4	Programmed cell death protein 4	45.70	-1.20	-2.30	0.00	0.00
106611970	sil1	NA	125.18	-1.20	-2.30	0.00	0.01
106583314	LOC106583314	serine/threonine-protein kinase 10-like	38.56	-1.20	-2.30	0.00	0.00

106588829	LOC106588829	pro-interleukin-16-like	44.41	-1.20	-2.29	0.00	0.00
100306863	mmp25	Matrix metalloproteinase-25	11.42	-1.20	-2.29	0.00	0.00
106580961	LOC106580961	high affinity immunoglobulin gamma Fc recepto	105.14	-1.20	-2.29	0.00	0.04
106565117	LOC106565117	differentially expressed in FDCP 6 homolog	30.88	-1.19	-2.29	0.00	0.01
100194953	irf4	Interferon regulatory factor 4	13.94	-1.19	-2.29	0.00	0.00
106606976	LOC106606976	ras-related protein Rab-37-like	23.15	-1.19	-2.29	0.00	0.00
106572807	LOC106572807	tumor necrosis factor receptor superfamily mer	43.01	-1.19	-2.28	0.00	0.04
106566776	LOC106566776	hyaluronidase-3-like	46.95	-1.19	-2.28	0.00	0.00
100196462	cats	Cathepsin S	285.00	-1.19	-2.27	0.00	0.03
106603899	LOC106603899	lymphocyte cytosolic protein 2-like	65.29	-1.19	-2.27	0.00	0.00
106584294	LOC106584294	uncharacterized LOC106584294	138.12	-1.18	-2.27	0.00	0.01
100136380	cd9	CD9 molecule	37.37	-1.18	-2.27	0.00	0.04
106586779	LOC106586779	type I inositol 3,4-bisphosphate 4-phosphatase-	11.59	-1.18	-2.27	0.00	0.00
106577368	slc1a4	NA	121.26	-1.18	-2.27	0.00	0.00
106603289	LOC106603289	oligodendrocyte-myelin glycoprotein-like	28.49	-1.18	-2.27	0.00	0.00
100194553	LOC100194553	VHSV-induced protein-like	123.13	-1.18	-2.27	0.00	0.00
106612899	aamdc	adipogenesis associated Mth938 domain contai	126.32	-1.18	-2.26	0.00	0.02
100194885	cd226	CD226 molecule	13.15	-1.18	-2.26	0.00	0.03
106609445	LOC106609445	adenosine deaminase CECR1-A-like	15.85	-1.18	-2.26	0.00	0.00
106605619	LOC106605619	uncharacterized LOC106605619	12.75	-1.18	-2.26	0.00	0.03
100195035	abi1	Abl interactor 1	42.39	-1.18	-2.26	0.00	0.00
106590233	LOC106590233	amyloid beta A4 precursor protein-binding fami	54.12	-1.17	-2.25	0.00	0.01
106586497	LOC106586497	myosin-9-like	16.82	-1.17	-2.25	0.00	0.00
106574341	LOC106574341	insulin receptor substrate 2-like	100.92	-1.17	-2.25	0.00	0.00
106613085	LOC106613085	protein EVI2B-like	21.59	-1.17	-2.25	0.00	0.03
106610834	LOC106610834	thrombospondin-1-like	71.03	-1.17	-2.24	0.00	0.01
100136933	b2m	beta-2-microglobulin	1609.16	-1.16	-2.24	0.00	0.01
106590291	LOC106590291	zinc finger CCHC domain-containing protein 2-li	105.30	-1.16	-2.24	0.00	0.02
106560813	LOC106560813	choline kinase alpha-like	42.17	-1.16	-2.24	0.00	0.02
106563764	LOC106563764	uncharacterized LOC106563764	111.43	-1.16	-2.24	0.00	0.03
106590560	LOC106590560	protein disulfide-isomerase A4-like	4590.80	-1.16	-2.24	0.00	0.05
100286449	fa49a	FAM49A	37.66	-1.16	-2.23	0.00	0.00
106569076	LOC106569076	uncharacterized LOC106569076	37.40	-1.16	-2.23	0.00	0.01
106572200	tp73	NA	20.87	-1.16	-2.23	0.00	0.01
106561505	LOC106561505	uncharacterized LOC106561505	77.23	-1.16	-2.23	0.00	0.00
100195074	papl	acid phosphatase 7, tartrate resistant (putative)	482.87	-1.16	-2.23	0.00	0.00
106560463	pde4b	phosphodiesterase 4B	32.75	-1.16	-2.23	0.00	0.01
100194878	lck	lymphocyte-specific protein tyrosine kinase	22.67	-1.16	-2.23	0.00	0.01
106573445	LOC106573445	uncharacterized LOC106573445	80.77	-1.16	-2.23	0.00	0.00
106607825	LOC106607825	cyclic AMP-dependent transcription factor ATF-	24.76	-1.15	-2.22	0.00	0.00

106567759	LOC106567759	uncharacterized LOC106567759	41.51	-1.15	-2.22	0.00	0.00
106590089	LOC106590089	protein phosphatase 1 regulatory subunit 3G-lik	354.62	-1.15	-2.22	0.00	0.02
106600890	LOC106600890	cathepsin D-like	20.91	-1.15	-2.22	0.00	0.01
106585107	LOC106585107	rho GTPase-activating protein 25-like	43.52	-1.15	-2.22	0.00	0.01
106570284	ube2e1	ubiquitin conjugating enzyme E2 E1	51.58	-1.15	-2.22	0.00	0.00
106586692	LOC106586692	serine/threonine-protein kinase SIK2-like	331.97	-1.15	-2.21	0.00	0.00
106562796	LOC106562796	non-muscle caldesmon-like	49.55	-1.15	-2.21	0.00	0.00
106604151	LOC106604151	glutathione peroxidase 3-like	195.08	-1.14	-2.21	0.00	0.00
106611681	LOC106611681	mucin-22-like	14.43	-1.14	-2.21	0.00	0.00
106583643	LOC106583643	bridging integrator 2-like	73.41	-1.14	-2.21	0.00	0.00
106582088	LOC106582088	poly [ADP-ribose] polymerase 4-like	370.25	-1.14	-2.21	0.00	0.01
106612646	LOC106612646	cylicin-1-like	2596.90	-1.14	-2.21	0.00	0.00
106574474	LOC106574474	tetraspanin-3-like	18.28	-1.14	-2.20	0.00	0.00
106584208	prkaa2	NA	16.58	-1.14	-2.20	0.00	0.01
106585085	LOC106585085	uncharacterized LOC106585085	34.69	-1.14	-2.20	0.00	0.00
106585863	rasef	NA	62.44	-1.14	-2.20	0.00	0.02
106582148	LOC106582148	cystathionine beta-synthase-like	354.19	-1.14	-2.20	0.00	0.00
100195079	klh13	Kelch-like protein 13	44.11	-1.14	-2.20	0.00	0.01
106585103	LOC106585103	hexokinase-2-like	18.41	-1.14	-2.20	0.00	0.00
106607317	LOC106607317	formin-like protein 1	43.38	-1.13	-2.20	0.00	0.01
100194749	kcrb	Creatine kinase B-type	284.99	-1.13	-2.19	0.00	0.03
106585183	LOC106585183	thioredoxin domain-containing protein 11-like	402.08	-1.13	-2.19	0.00	0.01
100286536	cl012	CL012 protein	279.35	-1.13	-2.19	0.00	0.01
106563771	LOC106563771	uncharacterized LOC106563771	230.70	-1.13	-2.19	0.00	0.02
106608944	LOC106608944	uncharacterized LOC106608944	20.15	-1.13	-2.19	0.00	0.00
106562353	LOC106562353	uncharacterized LOC106562353	24.05	-1.13	-2.19	0.00	0.00
106573249	LOC106573249	mitochondrial inner membrane protease subun	390.92	-1.13	-2.18	0.00	0.00
106603424	LOC106603424	integrator complex subunit 2-like	57.74	-1.13	-2.18	0.00	0.00
106589236	LOC106589236	uncharacterized LOC106589236	27.96	-1.13	-2.18	0.00	0.00
106580501	LOC106580501	rho GTPase-activating protein 25-like	71.84	-1.12	-2.18	0.00	0.02
106582437	LOC106582437	major histocompatibility complex class I-relatec	150.07	-1.12	-2.18	0.00	0.01
106569420	LOC106569420	rho GTPase-activating protein 30-like	33.22	-1.12	-2.18	0.00	0.04
106582367	LOC106582367	dnaJ homolog subfamily B member 11-like	930.99	-1.12	-2.17	0.00	0.01
106582667	LOC106582667	rho GTPase-activating protein 15-like	26.72	-1.12	-2.17	0.00	0.01
106611878	LOC106611878	uncharacterized LOC106611878	56.26	-1.12	-2.17	0.00	0.02
106560506	LOC106560506	uncharacterized LOC106560506	45472.52	-1.12	-2.17	0.00	0.04
106589046	LOC106589046	uncharacterized protein C6orf47-like	237.90	-1.12	-2.17	0.00	0.01
100195970	fmr1	fragile X mental retardation 1	18.33	-1.11	-2.16	0.00	0.01
106573132	LOC106573132	endoplasmin-like	209.90	-1.11	-2.16	0.00	0.01
106612334	LOC106612334	catenin alpha-1-like	685.46	-1.11	-2.15	0.00	0.01

106568752	LOC106568752	eukaryotic initiation factor 4A-II	156.12	-1.11	-2.15	0.00	0.00
106583826	LOC106583826	phospholipase D4-like	110.49	-1.11	-2.15	0.00	0.00
106604371	LOC106604371	fermitin family homolog 3-like	52.29	-1.11	-2.15	0.00	0.00
106564992	LOC106564992	uncharacterized protein C17orf67 homolog	189.59	-1.10	-2.15	0.00	0.04
106583606	LOC106583606	keratin, type I cytoskeletal 18-like	134.57	-1.10	-2.15	0.00	0.00
106589024	LOC106589024	RNA-binding protein 43-like	75.38	-1.10	-2.15	0.00	0.02
106565723	LOC106565723	bactericidal permeability-increasing protein-like	57.79	-1.10	-2.15	0.00	0.02
100196279	rhog	Rho-related GTP-binding protein RhoG	64.16	-1.10	-2.15	0.00	0.01
106579127	LOC106579127	endonuclease domain-containing 1 protein-like	43.49	-1.10	-2.14	0.00	0.00
106562850	LOC106562850	lymphocyte-specific protein 1-like	40.01	-1.10	-2.14	0.00	0.04
100380432	ptn12	yrosine-protein phosphatase non-receptor type [illegible]	78.63	-1.10	-2.14	0.00	0.00
100846970	irf1-2	interferon regulatory factor 1 isoform 2	161.43	-1.10	-2.14	0.00	0.00
106607206	LOC106607206	intercellular adhesion molecule 1-like	25.90	-1.09	-2.13	0.00	0.00
106586813	LOC106586813	thymosin beta-12	1117.77	-1.09	-2.13	0.00	0.00
106610434	LOC106610434	semaphorin-4F-like	37.47	-1.09	-2.13	0.00	0.00
106566806	LOC106566806	PH and SEC7 domain-containing protein 4-like	29.65	-1.09	-2.13	0.00	0.00
100196789	hcls1	hematopoietic cell-specific Lyn substrate 1	148.95	-1.09	-2.12	0.00	0.00
106560732	LOC106560732	ed guanylate kinase, WW and PDZ domain-cont	121.27	-1.09	-2.12	0.00	0.03
100380761	ltbp1	nt transforming growth factor beta binding prot	305.77	-1.09	-2.12	0.00	0.00
106595288	LOC106595288	60 kDa lysophospholipase-like	191.68	-1.09	-2.12	0.00	0.00
100194997	rhg15	Rho GTPase-activating protein 15	54.60	-1.09	-2.12	0.00	0.03
106599665	LOC106599665	T-cell receptor beta-1 chain C region-like	25.93	-1.08	-2.12	0.00	0.00
100380284	pcy2	Ethanolamine-phosphate cytidylyltransferase	1014.13	-1.08	-2.12	0.00	0.02
100380360	f13a	Coagulation factor XIII A chain	127.68	-1.08	-2.12	0.00	0.01
106603649	LOC106603649	uolar protein sorting-associated protein 11 hom	71.81	-1.08	-2.11	0.00	0.00
106569442	LOC106569442	zinc-binding protein A33-like	20155.11	-1.08	-2.11	0.00	0.02
106605328	LOC106605328	cathepsin S-like	188.83	-1.08	-2.11	0.00	0.01
106603074	LOC106603074	choline-phosphate cytidylyltransferase B-like	299.60	-1.08	-2.11	0.00	0.04
106581244	LOC106581244	uncharacterized LOC106581244	23.49	-1.08	-2.11	0.00	0.00
106609289	LOC106609289	plexin-C1-like	41.13	-1.07	-2.11	0.00	0.00
106578993	LOC106578993	SUN domain-containing protein 1-like	250.10	-1.07	-2.10	0.00	0.00
106582712	LOC106582712	cysteine and glycine-rich protein 1-like	65.14	-1.07	-2.10	0.00	0.00
106588382	LOC106588382	proteasome subunit beta type-7-like	115.62	-1.07	-2.10	0.00	0.01
100195582	srk2	Tyrosine-protein kinase SRK2	42.76	-1.07	-2.10	0.00	0.00
106565716	LOC106565716	ras association domain-containing protein 5-like	19.55	-1.07	-2.10	0.00	0.00
106612780	LOC106612780	uncharacterized LOC106612780	37.14	-1.07	-2.10	0.00	0.01
106576451	LOC106576451	uncharacterized LOC106576451	64.95	-1.07	-2.10	0.00	0.00
106600711	LOC106600711	uncharacterized LOC106600711	44.84	-1.07	-2.10	0.00	0.00
106562977	LOC106562977	heterogeneous nuclear ribonucleoprotein R	46.27	-1.07	-2.10	0.00	0.01
106576464	LOC106576464	gamma-secretase-activating protein-like	23.51	-1.07	-2.10	0.00	0.00

106613721	LOC106613721	minor histocompatibility protein HA-1-like	121.58	-1.07	-2.10	0.00	0.00
106568211	LOC106568211	zed threonine-rich GPI-anchored glycoprotein P.	31.39	-1.07	-2.09	0.00	0.00
106577132	LOC106577132	rotein phosphatase 1 regulatory subunit 3C-B-lik	281.55	-1.07	-2.09	0.00	0.00
106600740	LOC106600740	ıker for activation of T-cells family member 1-lik	42.35	-1.06	-2.09	0.00	0.00
106561924	LOC106561924	tetraspanin-4-like	74.40	-1.06	-2.09	0.00	0.00
106571173	LOC106571173	leucine-rich alpha-2-glycoprotein-like	3380.76	-1.06	-2.09	0.00	0.05
100196053	srgn	serglycin	216.39	-1.06	-2.09	0.00	0.01
100380684	LOC100380684	unconventional myosin-If	94.18	-1.06	-2.09	0.00	0.00
100195398	al3a2	Fatty aldehyde dehydrogenase	39.93	-1.06	-2.08	0.00	0.04
106567410	LOC106567410	plasmic/endoplasmic reticulum calcium ATPase	62.80	-1.06	-2.08	0.00	0.01
100196627	cideb	cell death-inducing DFFA-like effector b	479.72	-1.06	-2.08	0.00	0.01
106572477	LOC106572477	tubulin alpha-8 chain-like	137.26	-1.06	-2.08	0.00	0.01
100316863	pdia3	protein disulfide isomerase family A, member 3	3110.57	-1.05	-2.08	0.00	0.02
106581951	LOC106581951	ıtor of RNA polymerase II transcription subunit 1	178.29	-1.05	-2.07	0.00	0.01
106605922	LOC106605922	nuclear pore membrane glycoprotein 210-like	42.50	-1.05	-2.07	0.00	0.00
106600828	LOC106600828	sulin-like growth factor 2 mRNA-binding protein	31.80	-1.05	-2.07	0.00	0.01
106588209	LOC106588209	uncharacterized LOC106588209	36.21	-1.05	-2.07	0.00	0.00
106606823	LOC106606823	CD97 antigen-like	31.31	-1.05	-2.06	0.00	0.00
106614017	LOC106614017	denovirus E1B 19 kDa protein-interacting protei	103.29	-1.04	-2.06	0.00	0.00
106563698	LOC106563698	complement factor B-like	23053.23	-1.04	-2.06	0.00	0.01
106570100	LOC106570100	protein S100-A1-like	58.21	-1.04	-2.06	0.00	0.01
106598526	LOC106598526	regulator of G-protein signaling 21-like	54.83	-1.04	-2.06	0.00	0.03
106565108	LOC106565108	nbesin receptor-activated protein C6orf89 homo	619.74	-1.04	-2.06	0.00	0.00
106612599	LOC106612599	en-activated protein kinase kinase kinase kinase	94.01	-1.04	-2.06	0.00	0.04
106601073	LOC106601073	hemoglobin subunit beta-1-like	1381.03	-1.04	-2.06	0.00	0.01
106601388	LOC106601388	growth-regulated alpha protein-like	32.54	-1.04	-2.06	0.00	0.00
106605286	LOC106605286	uncharacterized LOC106605286	215.11	-1.04	-2.06	0.00	0.01
106601108	LOC106601108	uncharacterized LOC106601108	62.12	-1.04	-2.05	0.00	0.02
106599071	LOC106599071	zinc-alpha-2-glycoprotein-like	82.64	-1.04	-2.05	0.00	0.02
106581251	LOC106581251	VD repeat and SOCS box-containing protein 1-lik	198.87	-1.03	-2.05	0.00	0.01
106567637	LOC106567637	hypoxia up-regulated protein 1-like	656.73	-1.03	-2.05	0.00	0.00
106608585	LOC106608585	chinoderm microtubule-associated protein-like	26.90	-1.03	-2.05	0.00	0.00
106606251	LOC106606251	rho GDP-dissociation inhibitor 2-like	47.18	-1.03	-2.04	0.00	0.01
100380749	LOC100380749	hypoxia up-regulated protein 1	2058.08	-1.03	-2.04	0.00	0.03
106582236	LOC106582236	terferon-induced guanylate-binding protein 1-lil	64.33	-1.03	-2.04	0.00	0.04
106591148	LOC106591148	uncharacterized LOC106591148	75.32	-1.03	-2.04	0.00	0.00
106613902	LOC106613902	calreticulin-like	539.93	-1.03	-2.04	0.00	0.02
106574040	LOC106574040	PDZK1-interacting protein 1-like	88.43	-1.02	-2.03	0.00	0.01
106564771	rrbp1	NA	3835.98	-1.02	-2.03	0.00	0.00
106611175	LOC106611175	NF-kappa-B inhibitor alpha-like	125.55	-1.02	-2.03	0.00	0.01

106562284	LOC106562284	interferon regulatory factor 8-like	46.57	-1.02	-2.03	0.00	0.01
106578614	LOC106578614	dnaJ homolog subfamily B member 6-like	43.50	-1.02	-2.03	0.00	0.00
106606371	LOC106606371	uncharacterized LOC106606371	60690.29	-1.02	-2.03	0.00	0.00
106587384	LOC106587384	cytochrome b-245 light chain-like	48.20	-1.02	-2.03	0.00	0.00
106588788	LOC106588788	uncharacterized LOC106588788	69.87	-1.02	-2.03	0.00	0.00
106571222	LOC106571222	NF-kappa-B inhibitor alpha-like	443.58	-1.02	-2.02	0.00	0.01
100137055	csf3r	granulocyte colony-stimulating factor receptor	61.98	-1.02	-2.02	0.00	0.04
106603906	LOC106603906	early growth response protein 1-like	88.93	-1.01	-2.02	0.00	0.01
106560550	LOC106560550	complement factor H-like	52152.14	-1.01	-2.02	0.00	0.00
106584302	LOC106584302	sosome-associated membrane glycoprotein 3-lil	30.25	-1.01	-2.02	0.00	0.00
106580178	LOC106580178	uncharacterized LOC106580178	94.77	-1.01	-2.02	0.00	0.00
106584651	LOC106584651	protein disulfide-isomerase A3-like	4162.69	-1.01	-2.01	0.00	0.00
106609735	LOC106609735	ane-associated progesterone receptor compone	116.84	-1.00	-2.00	0.00	0.00
100195482	tpsnr	Tapasin-related protein	104.72	-1.00	-2.00	0.00	0.01
100194767	pim1	pim-1 oncogene	127.32	-1.00	-2.00	0.00	0.02
106565637	p3h1	prolyl 3-hydroxylase 1	35.48	-1.00	-2.00	0.00	0.00
106568321	LOC106568321	interleukin-6 receptor subunit beta-like	48.58	-1.00	-2.00	0.00	0.02
106582798	nprl2	NPR2-like, GATOR1 complex subunit	38.41	-1.00	-2.00	0.00	0.01
106582344	LOC106582344	ADP-ribosylation factor-like protein 5A	93.70	-1.00	-2.00	0.00	0.00

Supplementary Table S3

Ploidy group: Diploid

GO terms for down regulated DEGs

	**GO terms for down regulated DEGs for fry when compared to parr (Benjamini value ≤0,05)**	**Count**	**Percentage**	**P Value**	**Genes**	**List total**	**Pop hits**	**Pop Total**	**Fold Enrich**	**Bonferroni**	**Benjamini**	**log10 benj**	**FDR**
GOTERM_I	GO:0009206~purine ribonucleoside triphosphate biosynthetic process	16	1.338912	9.77904E-10	106574334	214	30	2694	6.71	0.00	0.00	6.14	0.00
GOTERM_I	GO:0009145~purine nucleoside triphosphate biosynthetic process	16	1.338912	9.77904E-10	106574334	214	30	2694	6.71	0.00	0.00	6.14	0.00
GOTERM_I	GO:0042451~purine nucleoside biosynthetic process	19	1.589958	2.79337E-09	100306805	214	46	2694	5.20	0.00	0.00	5.98	0.00
GOTERM_I	GO:0046129~purine ribonucleoside biosynthetic process	19	1.589958	2.79337E-09	100306805	214	46	2694	5.20	0.00	0.00	5.98	0.00
GOTERM_I	GO:0015986~ATP synthesis coupled proton transport	14	1.171548	7.34734E-09	106574334	214	25	2694	7.05	0.00	0.00	5.74	0.00
GOTERM_I	GO:0015985~energy coupled proton transport, down electrochemical gradient	14	1.171548	7.34734E-09	106574334	214	25	2694	7.05	0.00	0.00	5.74	0.00
GOTERM_I	GO:0006754~ATP biosynthetic process	14	1.171548	7.34734E-09	106574334	214	25	2694	7.05	0.00	0.00	5.74	0.00
GOTERM_I	GO:0009152~purine ribonucleotide biosynthetic process	18	1.506276	9.95479E-09	106574334	214	44	2694	5.15	0.00	0.00	5.73	0.00
GOTERM_I	GO:0009201~ribonucleoside triphosphate biosynthetic process	16	1.338912	1.42962E-08	106574334	214	35	2694	5.75	0.00	0.00	5.67	0.00
GOTERM_I	GO:0006164~purine nucleotide biosynthetic process	18	1.506276	1.48896E-08	106574334	214	45	2694	5.04	0.00	0.00	5.73	0.00
GOTERM_I	GO:0072522~purine-containing compound biosynthetic process	19	1.589958	1.93079E-08	100306805	214	51	2694	4.69	0.00	0.00	5.69	0.00
GOTERM_I	GO:0009142~nucleoside triphosphate biosynthetic process	16	1.338912	3.57004E-08	106574334	214	37	2694	5.44	0.00	0.00	5.48	0.00
GOTERM_I	GO:0009168~purine ribonucleoside monophosphate biosynthetic process	16	1.338912	8.3018E-08	106574334	214	39	2694	5.16	0.00	0.00	5.16	0.00
GOTERM_I	GO:0009127~purine nucleoside monophosphate biosynthetic process	16	1.338912	8.3018E-08	106574334	214	39	2694	5.16	0.00	0.00	5.16	0.00
GOTERM_I	GO:1901659~glycosyl compound biosynthetic process	19	1.589958	1.02596E-07	100306805	214	56	2694	4.27	0.00	0.00	5.12	0.00
GOTERM_I	GO:0009163~nucleoside biosynthetic process	19	1.589958	1.02596E-07	100306805	214	56	2694	4.27	0.00	0.00	5.12	0.00
GOTERM_I	GO:0042455~ribonucleoside biosynthetic process	19	1.589958	1.02596E-07	100306805	214	56	2694	4.27	0.00	0.00	5.12	0.00
GOTERM_I	GO:0009260~ribonucleotide biosynthetic process	18	1.506276	2.45418E-07	106574334	214	53	2694	4.28	0.00	0.00	4.78	0.00
GOTERM_I	GO:0046390~ribose phosphate biosynthetic process	18	1.506276	2.45418E-07	106574334	214	53	2694	4.28	0.00	0.00	4.78	0.00
GOTERM_I	GO:0009205~purine ribonucleoside triphosphate metabolic process	22	1.841004	5.27831E-07	10658663C	214	81	2694	3.42	0.00	0.00	4.48	0.00
GOTERM_I	GO:0009144~purine nucleoside triphosphate metabolic process	22	1.841004	5.27831E-07	10658663C	214	81	2694	3.42	0.00	0.00	4.48	0.00
GOTERM_I	GO:0009165~nucleotide biosynthetic process	20	1.67364	5.37917E-07	106574334	214	68	2694	3.70	0.00	0.00	4.51	0.00
GOTERM_I	GO:1901293~nucleoside phosphate biosynthetic process	20	1.67364	5.37917E-07	106574334	214	68	2694	3.70	0.00	0.00	4.51	0.00
GOTERM_I	GO:0046128~purine ribonucleoside metabolic process	24	2.008368	5.61235E-07	100306805	214	95	2694	3.18	0.00	0.00	4.52	0.00
GOTERM_I	GO:0042278~purine nucleoside metabolic process	24	2.008368	5.61235E-07	100306805	214	95	2694	3.18	0.00	0.00	4.52	0.00
GOTERM_I	GO:0006753~nucleoside phosphate metabolic process	28	2.343096	9.20959E-07	10658663C	214	127	2694	2.78	0.00	0.00	4.34	0.00
GOTERM_I	GO:0009117~nucleotide metabolic process	28	2.343096	9.20959E-07	10658663C	214	127	2694	2.78	0.00	0.00	4.34	0.00
GOTERM_I	GO:0009156~ribonucleoside monophosphate biosynthetic process	16	1.338912	1.02194E-06	106574334	214	46	2694	4.38	0.00	0.00	4.32	0.00
GOTERM_I	GO:0019693~ribose phosphate metabolic process	25	2.09205	1.17914E-06	10658663C	214	106	2694	2.97	0.00	0.00	4.29	0.00
GOTERM_I	GO:0009150~purine ribonucleotide metabolic process	23	1.924686	1.26671E-06	10658663C	214	92	2694	3.15	0.00	0.00	4.28	0.00
GOTERM_I	GO:0009124~nucleoside monophosphate biosynthetic process	16	1.338912	1.39692E-06	106574334	214	47	2694	4.29	0.00	0.00	4.26	0.00
GOTERM_I	GO:0009199~ribonucleoside triphosphate metabolic process	22	1.841004	1.55431E-06	10658663C	214	86	2694	3.22	0.00	0.00	4.24	0.00
GOTERM_I	GO:0055086~nucleobase-containing small molecule metabolic process	29	2.426778	1.55474E-06	100306805	214	138	2694	2.65	0.00	0.00	4.26	0.00
GOTERM_I	GO:0006163~purine nucleotide metabolic process	23	1.924686	1.87658E-06	10658663C	214	94	2694	3.08	0.00	0.00	4.20	0.00
GOTERM_I	GO:0090407~organophosphate biosynthetic process	21	1.757322	1.87952E-06	10658663C	214	80	2694	3.30	0.00	0.00	4.21	0.00
GOTERM_I	GO:0009141~nucleoside triphosphate metabolic process	22	1.841004	1.90688E-06	10658663C	214	87	2694	3.18	0.00	0.00	4.23	0.00
GOTERM_I	GO:0072521~purine-containing compound metabolic process	24	2.008368	2.16477E-06	100306805	214	102	2694	2.96	0.00	0.00	4.19	0.00
GOTERM_I	GO:0046034~ATP metabolic process	20	1.67364	3.45386E-06	10658663C	214	76	2694	3.31	0.00	0.00	4.00	0.01
GOTERM_I	GO:0009119~ribonucleoside metabolic process	24	2.008368	3.69505E-06	100306805	214	105	2694	2.88	0.00	0.00	3.99	0.01
GOTERM_I	GO:0009126~purine nucleoside monophosphate metabolic process	21	1.757322	6.31859E-06	10658663C	214	86	2694	3.07	0.00	0.00	3.77	0.01
GOTERM_I	GO:0009167~purine ribonucleoside monophosphate metabolic process	21	1.757322	6.31859E-06	10658663C	214	86	2694	3.07	0.00	0.00	3.77	0.01
GOTERM_I	GO:0009259~ribonucleotide metabolic process	23	1.924686	6.73587E-06	10658663C	214	101	2694	2.87	0.01	0.00	3.76	0.01
GOTERM_I	GO:1901137~carbohydrate derivative biosynthetic process	22	1.841004	8.65543E-06	100306805	214	95	2694	2.92	0.01	0.00	3.67	0.01
GOTERM_I	GO:0044711~single-organism biosynthetic process	29	2.426778	1.00209E-05	100306805	214	151	2694	2.42	0.01	0.00	3.62	0.02
GOTERM_I	GO:1901657~glycosyl compound metabolic process	24	2.008368	1.37354E-05	100306805	214	113	2694	2.67	0.01	0.00	3.49	0.02
GOTERM_I	GO:0009116~nucleoside metabolic process	24	2.008368	1.37354E-05	100306805	214	113	2694	2.67	0.01	0.00	3.49	0.02
GOTERM_I	GO:1902600~hydrogen ion transmembrane transport	14	1.171548	1.42256E-05	106574334	214	43	2694	4.10	0.01	0.00	3.49	0.02
GOTERM_I	GO:0009161~ribonucleoside monophosphate metabolic process	21	1.757322	2.2207E-05	10658663C	214	93	2694	2.84	0.02	0.00	3.31	0.03
GOTERM_I	GO:0015992~proton transport	14	1.171548	2.46074E-05	106574334	214	45	2694	3.92	0.02	0.00	3.28	0.04
GOTERM_I	GO:0006818~hydrogen transport	14	1.171548	2.46074E-05	106574334	214	45	2694	3.92	0.02	0.00	3.28	0.04

GOTERM_I	GO:0009123~nucleoside monophosphate metabolic process	21	1.757322	2.62509E-05	106586630	214	94	2694	2.81	0.02	0.00	3.26	0.04
GOTERM_I	GO:0019637~organophosphate metabolic process	29	2.426778	2.76864E-05	106586630	214	159	2694	2.30	0.02	0.00	3.25	0.04
GOTERM_I	GO:0044710~single-organism metabolic process	56	4.686192	2.96314E-05	106586630	214	416	2694	1.69	0.02	0.00	3.23	0.05
GOTERM_I	GO:0098655~cation transmembrane transport	14	1.171548	3.1933E-05	106574334	214	46	2694	3.83	0.02	0.00	3.21	0.05
GOTERM_I	GO:0034220~ion transmembrane transport	14	1.171548	3.1933E-05	106574334	214	46	2694	3.83	0.02	0.00	3.21	0.05
GOTERM_I	GO:0098660~inorganic ion transmembrane transport	14	1.171548	3.1933E-05	106574334	214	46	2694	3.83	0.02	0.00	3.21	0.05
GOTERM_I	GO:0098662~inorganic cation transmembrane transport	14	1.171548	3.1933E-05	106574334	214	46	2694	3.83	0.02	0.00	3.21	0.05
GOTERM_I	GO:0006796~phosphate-containing compound metabolic process	32	2.677824	6.54687E-05	106586630	214	193	2694	2.09	0.05	0.00	2.91	0.10
GOTERM_I	GO:0006793~phosphorus metabolic process	32	2.677824	7.25421E-05	106586630	214	194	2694	2.08	0.05	0.00	2.88	0.11
GOTERM_I	GO:0044281~small molecule metabolic process	42	3.514644	9.39774E-05	106564857	214	291	2694	1.82	0.07	0.00	2.78	0.14
GOTERM_I	GO:1901135~carbohydrate derivative metabolic process	29	2.426778	0.000133871	100306805	214	173	2694	2.11	0.10	0.00	2.63	0.20
GOTERM_I	GO:0055114~oxidation-reduction process	14	1.171548	0.000196296	106564857	214	54	2694	3.26	0.14	0.00	2.48	0.30
GOTERM_I	GO:0015672~monovalent inorganic cation transport	14	1.171548	0.00023934	106574334	214	55	2694	3.20	0.16	0.00	2.40	0.36
GOTERM_I	GO:0051726~regulation of cell cycle	10	0.83682	0.000335697	106573930	214	30	2694	4.20	0.22	0.01	2.26	0.51
GOTERM_I	GO:0055085~transmembrane transport	16	1.338912	0.001009169	106565839	214	79	2694	2.55	0.53	0.02	1.80	1.52
GOTERM_I	GO:0007017~microtubule-based process	12	1.004184	0.001155217	100196567	214	49	2694	3.08	0.58	0.02	1.75	1.74
GOTERM_I	GO:0007049~cell cycle	14	1.171548	0.00204854	100286418	214	68	2694	2.59	0.78	0.03	1.51	3.07
GOTERM_I	GO:0022402~cell cycle process	11	0.920502	0.002960385	100286418	214	47	2694	2.95	0.89	0.04	1.36	4.41
GOTERM_I	GO:0006812~cation transport	16	1.338912	0.003494424	106612257	214	89	2694	2.26	0.93	0.05	1.30	5.19

	(Benjamini value > 0,05)	Count	Percentage	P Value	Genes	List total	Pop hits	Pop Total	Fold Enrich	Bonferroni	Benjamini	log10 benj	FDR
GOTERM_I	GO:0006811~ion transport	17	1.422594	0.005960482	106612257	214	103	2694	2.08	0.99	0.08	1.08	8.69
GOTERM_I	GO:1903047~mitotic cell cycle process	8	0.669456	0.008916888	100286418	214	31	2694	3.25	1.00	0.12	0.93	12.74
GOTERM_I	GO:0008152~metabolic process	136	11.38075	0.012463438	106586630	214	1505	2694	1.14	1.00	0.16	0.80	17.37
GOTERM_I	GO:0000278~mitotic cell cycle	8	0.669456	0.012653485	100286418	214	33	2694	3.05	1.00	0.16	0.80	17.61
GOTERM_I	GO:1901564~organonitrogen compound metabolic process	53	4.435146	0.017913992	106586630	214	503	2694	1.33	1.00	0.21	0.67	24.04
GOTERM_I	GO:1901566~organonitrogen compound biosynthetic process	42	3.514644	0.019494167	100306805	214	381	2694	1.39	1.00	0.23	0.64	25.88
GOTERM_I	GO:0044770~cell cycle phase transition	5	0.41841	0.020214525	100286718	214	14	2694	4.50	1.00	0.23	0.64	26.70
GOTERM_I	GO:0044772~mitotic cell cycle phase transition	5	0.41841	0.020214525	100286718	214	14	2694	4.50	1.00	0.23	0.64	26.70
GOTERM_I	GO:1902578~single-organism localization	26	2.175732	0.020762054	106612257	214	209	2694	1.57	1.00	0.23	0.63	27.32
GOTERM_I	GO:0030163~protein catabolic process	12	1.004184	0.040403436	106590672	214	78	2694	1.94	1.00	0.40	0.40	46.60
GOTERM_I	GO:0045333~cellular respiration	7	0.585774	0.041453966	106564857	214	33	2694	2.67	1.00	0.40	0.39	47.48
GOTERM_I	GO:0009057~macromolecule catabolic process	14	1.171548	0.045216559	100196014	214	99	2694	1.78	1.00	0.43	0.37	50.53
GOTERM_I	GO:0015980~energy derivation by oxidation of organic compounds	7	0.585774	0.047145412	106564857	214	34	2694	2.59	1.00	0.44	0.36	52.03
GOTERM_I	GO:0015991~ATP hydrolysis coupled proton transport	6	0.502092	0.049320291	106600830	214	26	2694	2.91	1.00	0.45	0.35	53.67
GOTERM_I	GO:0015988~energy coupled proton transmembrane transport, against electrochemical gradient	6	0.502092	0.049320291	106600830	214	26	2694	2.91	1.00	0.45	0.35	53.67
GOTERM_I	GO:0090662~ATP hydrolysis coupled transmembrane transport	6	0.502092	0.049320291	106600830	214	26	2694	2.91	1.00	0.45	0.35	53.67
GOTERM_I	GO:0006081~cellular aldehyde metabolic process	4	0.334728	0.050030443	100196702	214	11	2694	4.58	1.00	0.45	0.35	54.19
GOTERM_I	GO:0006091~generation of precursor metabolites and energy	10	0.83682	0.052015174	106564857	214	62	2694	2.03	1.00	0.45	0.34	55.63
GOTERM_I	GO:0042401~cellular biogenic amine biosynthetic process	3	0.251046	0.053043885	100306805	214	5	2694	7.55	1.00	0.46	0.34	56.35
GOTERM_I	GO:0009309~amine biosynthetic process	3	0.251046	0.053043885	100306805	214	5	2694	7.55	1.00	0.46	0.34	56.35
GOTERM_I	GO:0006596~polyamine biosynthetic process	3	0.251046	0.053043885	100306805	214	5	2694	7.55	1.00	0.46	0.34	56.35
GOTERM_I	GO:0006595~polyamine metabolic process	3	0.251046	0.053043885	100306805	214	5	2694	7.55	1.00	0.46	0.34	56.35
GOTERM_I	GO:0051234~establishment of localization	40	3.34728	0.055097008	106586630	214	386	2694	1.30	1.00	0.46	0.33	57.77
GOTERM_I	GO:0044265~cellular macromolecule catabolic process	12	1.004184	0.063692796	106590672	214	84	2694	1.80	1.00	0.51	0.29	63.25
GOTERM_I	GO:0044765~single-organism transport	23	1.924686	0.068292677	106565839	214	201	2694	1.44	1.00	0.53	0.28	65.91
GOTERM_I	GO:0044248~cellular catabolic process	15	1.25523	0.07401949	100196014	214	117	2694	1.61	1.00	0.55	0.26	68.96
GOTERM_I	GO:0006810~transport	39	3.263598	0.075213373	106586630	214	384	2694	1.28	1.00	0.56	0.26	69.56
GOTERM_I	GO:0006006~glucose metabolic process	4	0.334728	0.077197526	100195420	214	13	2694	3.87	1.00	0.56	0.25	70.54
GOTERM_I	GO:0000280~nuclear division	5	0.41841	0.090156244	100286418	214	22	2694	2.86	1.00	0.61	0.21	76.24
GOTERM_I	GO:0007067~mitotic nuclear division	5	0.41841	0.090156244	100286418	214	22	2694	2.86	1.00	0.61	0.21	76.24
GOTERM_I	GO:0006511~ubiquitin-dependent protein catabolic process	8	0.669456	0.095208446	106590672	214	50	2694	2.01	1.00	0.63	0.20	78.17
GOTERM_I	GO:0043632~modification-dependent macromolecule catabolic process	8	0.669456	0.095208446	106590672	214	50	2694	2.01	1.00	0.63	0.20	78.17
GOTERM_I	GO:0019941~modification-dependent protein catabolic process	8	0.669456	0.095208446	106590672	214	50	2694	2.01	1.00	0.63	0.20	78.17
GOTERM_I	GO:1901575~organic substance catabolic process	19	1.589958	0.096046129	100194617	214	165	2694	1.45	1.00	0.63	0.20	78.48
GOTERM_I	GO:0044257~cellular protein catabolic process	10	0.83682	0.096956219	106590672	214	70	2694	1.80	1.00	0.63	0.20	78.80
GOTERM_I	GO:0051603~proteolysis involved in cellular protein catabolic process	10	0.83682	0.096956219	106590672	214	70	2694	1.80	1.00	0.63	0.20	78.80

Ploidy group: Triploid

GO terms for down regulated DEGs

	GO terms for down regulated DEGs for fry when compared to parr (Benjamini value ≤0,05)	Count	Percentage (%)	P Value	Genes	List total	Pop hits	Pop Total	Fold Enrichment	Bonferroni	Benjamini	log10 benjami	FDR
GOTERM_BP_ALL	GO:0009117~nucleotide metabolic process	20	3.154574132	1.97E-07	106586630, 10	106	127	2694	4.00	0.00	0.00	4.64	0.00
GOTERM_BP_ALL	GO:0006753~nucleoside phosphate metabolic process	20	3.154574132	1.97E-07	106586630, 10	106	127	2694	4.00	0.00	0.00	4.64	0.00
GOTERM_BP_ALL	GO:0006754~ATP biosynthetic process	10	1.577287066	1.79E-07	106586630, 10	106	25	2694	10.17	0.00	0.00	4.59	0.00
GOTERM_BP_ALL	GO:0015985~energy coupled proton transport, down electrochemical gradient	10	1.577287066	1.79E-07	106586630, 10	106	25	2694	10.17	0.00	0.00	4.59	0.00
GOTERM_BP_ALL	GO:0015986~ATP synthesis coupled proton transport	10	1.577287066	1.79E-07	106586630, 10	106	25	2694	10.17	0.00	0.00	4.59	0.00
GOTERM_BP_ALL	GO:0009165~nucleotide biosynthetic process	15	2.365930599	1.67E-07	106574334, 10	106	68	2694	5.61	0.00	0.00	4.49	0.00
GOTERM_BP_ALL	GO:1901293~nucleoside phosphate biosynthetic process	15	2.365930599	1.67E-07	106574334, 10	106	68	2694	5.61	0.00	0.00	4.49	0.00
GOTERM_BP_ALL	GO:0009168~purine ribonucleoside monophosphate biosynthetic process	12	1.892744479	1.26E-07	106586630, 10	106	39	2694	7.82	0.00	0.00	4.44	0.00
GOTERM_BP_ALL	GO:0009127~purine nucleoside monophosphate biosynthetic process	12	1.892744479	1.26E-07	106586630, 10	106	39	2694	7.82	0.00	0.00	4.44	0.00
GOTERM_BP_ALL	GO:0009152~purine ribonucleotide biosynthetic process	12	1.892744479	4.88E-07	106586630, 10	106	44	2694	6.93	0.00	0.00	4.33	0.00
GOTERM_BP_ALL	GO:0042451~purine nucleoside biosynthetic process	12	1.892744479	7.95E-07	106586630, 10	106	46	2694	6.63	0.00	0.00	4.29	0.00
GOTERM_BP_ALL	GO:0046129~purine ribonucleoside biosynthetic process	12	1.892744479	7.95E-07	106586630, 10	106	46	2694	6.63	0.00	0.00	4.29	0.00
GOTERM_BP_ALL	GO:0009156~ribonucleoside monophosphate biosynthetic process	12	1.892744479	7.95E-07	106586630, 10	106	46	2694	6.63	0.00	0.00	4.29	0.00
GOTERM_BP_ALL	GO:0006164~purine nucleotide biosynthetic process	12	1.892744479	6.25E-07	106586630, 10	106	45	2694	6.78	0.00	0.00	4.29	0.00
GOTERM_BP_ALL	GO:0055086~nucleobase-containing small molecule metabolic process	20	3.154574132	7.58E-07	106586630, 10	106	138	2694	3.68	0.00	0.00	4.26	0.00
GOTERM_BP_ALL	GO:0009206~purine ribonucleoside triphosphate biosynthetic process	10	1.577287066	1.06E-06	106586630, 10	106	30	2694	8.47	0.00	0.00	4.21	0.00
GOTERM_BP_ALL	GO:0009145~purine nucleoside triphosphate biosynthetic process	10	1.577287066	1.06E-06	106586630, 10	106	30	2694	8.47	0.00	0.00	4.21	0.00
GOTERM_BP_ALL	GO:0009124~nucleoside monophosphate biosynthetic process	13	2.050473186	1.09E-07	106574334, 10	106	47	2694	7.03	0.00	0.00	4.20	0.00
GOTERM_BP_ALL	GO:0090407~organophosphate biosynthetic process	15	2.365930599	1.39E-06	106574334, 10	106	80	2694	4.77	0.00	0.00	4.14	0.00
GOTERM_BP_ALL	GO:0007017~microtubule-based process	12	1.892744479	1.57E-06	100196567, 10	106	49	2694	6.22	0.00	0.00	4.12	0.00
GOTERM_BP_ALL	GO:0006796~phosphate-containing compound metabolic process	23	3.627760252	2.51E-06	100195150, 10	106	193	2694	3.03	0.00	0.00	4.01	0.00
GOTERM_BP_ALL	GO:1901137~carbohydrate derivative biosynthetic process	16	2.523659306	2.21E-06	106574334, 10	106	95	2694	4.28	0.00	0.00	4.01	0.00
GOTERM_BP_ALL	GO:0006793~phosphorus metabolic process	23	3.627760252	2.75E-06	100195150, 10	106	194	2694	3.01	0.00	0.00	4.00	0.00
GOTERM_BP_ALL	GO:0072522~purine-containing compound biosynthetic process	12	1.892744479	2.41E-06	106586630, 10	106	51	2694	5.98	0.00	0.00	4.00	0.00
GOTERM_BP_ALL	GO:0009260~ribonucleotide biosynthetic process	12	1.892744479	3.61E-06	106586630, 10	106	53	2694	5.75	0.00	0.00	3.91	0.01
GOTERM_BP_ALL	GO:0046390~ribose phosphate biosynthetic process	12	1.892744479	3.61E-06	106586630, 10	106	53	2694	5.75	0.00	0.00	3.91	0.01
GOTERM_BP_ALL	GO:0009201~ribonucleoside triphosphate biosynthetic process	10	1.577287066	4.46E-06	106586630, 10	106	35	2694	7.26	0.00	0.00	3.84	0.01
GOTERM_BP_ALL	GO:1901659~glycosyl compound biosynthetic process	12	1.892744479	6.39E-06	106586630, 10	106	56	2694	5.45	0.00	0.00	3.71	0.01
GOTERM_BP_ALL	GO:0042455~ribonucleoside biosynthetic process	12	1.892744479	6.39E-06	106586630, 10	106	56	2694	5.45	0.00	0.00	3.71	0.01
GOTERM_BP_ALL	GO:0009163~nucleoside biosynthetic process	12	1.892744479	6.39E-06	106586630, 10	106	56	2694	5.45	0.00	0.00	3.71	0.01
GOTERM_BP_ALL	GO:0019637~organophosphate metabolic process	20	3.154574132	6.81E-06	106586630, 10	106	159	2694	3.20	0.00	0.00	3.70	0.01
GOTERM_BP_ALL	GO:0007049~cell cycle	13	2.050473186	7.67E-06	106584418, 10	106	68	2694	4.86	0.00	0.00	3.69	0.01
GOTERM_BP_ALL	GO:0009142~nucleoside triphosphate biosynthetic process	10	1.577287066	7.36E-06	106586630, 10	106	37	2694	6.87	0.00	0.00	3.69	0.01
GOTERM_BP_ALL	GO:0044281~small molecule metabolic process	28	4.416403785	8.68E-06	106564857, 10	106	291	2694	2.45	0.01	0.00	3.66	0.01
GOTERM_BP_ALL	GO:0019693~ribose phosphate metabolic process	16	2.523659306	9.08E-06	106574334, 10	106	106	2694	3.84	0.01	0.00	3.66	0.01
GOTERM_BP_ALL	GO:0009123~nucleoside monophosphate metabolic process	15	2.365930599	1.01E-05	106574334, 10	106	94	2694	4.06	0.01	0.00	3.63	0.01
GOTERM_BP_ALL	GO:0044711~single-organism biosynthetic process	19	2.996845426	1.28E-05	106574334, 10	106	151	2694	3.20	0.01	0.00	3.54	0.02
GOTERM_BP_ALL	GO:0009167~purine ribonucleoside monophosphate metabolic process	14	2.208201893	1.84E-05	106574334, 10	106	86	2694	4.14	0.01	0.00	3.40	0.03
GOTERM_BP_ALL	GO:0009126~purine nucleoside monophosphate metabolic process	14	2.208201893	1.84E-05	106574334, 10	106	86	2694	4.14	0.01	0.00	3.40	0.03
GOTERM_BP_ALL	GO:0044710~single-organism metabolic process	34	5.362776025	2.07E-05	106564857, 10	106	416	2694	2.08	0.01	0.00	3.37	0.03
GOTERM_BP_ALL	GO:1901135~carbohydrate derivative metabolic process	20	3.154574132	2.37E-05	106574334, 10	106	173	2694	2.94	0.01	0.00	3.32	0.03
GOTERM_BP_ALL	GO:0046034~ATP metabolic process	13	2.050473186	2.51E-05	106574334, 10	106	76	2694	4.35	0.01	0.00	3.31	0.04
GOTERM_BP_ALL	GO:1902600~hydrogen ion transmembrane transport	10	1.577287066	2.74E-05	106586630, 10	106	43	2694	5.91	0.02	0.00	3.29	0.04
GOTERM_BP_ALL	GO:0009150~purine ribonucleotide metabolic process	14	2.208201893	3.87E-05	106574334, 10	106	92	2694	3.87	0.02	0.00	3.15	0.06
GOTERM_BP_ALL	GO:0015992~proton transport	10	1.577287066	4.04E-05	106586630, 10	106	45	2694	5.65	0.02	0.00	3.15	0.06
GOTERM_BP_ALL	GO:0006818~hydrogen transport	10	1.577287066	4.04E-05	106586630, 10	106	45	2694	5.65	0.02	0.00	3.15	0.06
GOTERM_BP_ALL	GO:0009161~ribonucleoside monophosphate metabolic process	14	2.208201893	4.35E-05	106574334, 10	106	93	2694	3.83	0.02	0.00	3.13	0.06
GOTERM_BP_ALL	GO:0006163~purine nucleotide metabolic process	14	2.208201893	4.89E-05	106574334, 10	106	94	2694	3.79	0.03	0.00	3.12	0.07
GOTERM_BP_ALL	GO:0009144~purine nucleoside triphosphate metabolic process	13	2.050473186	4.87E-05	106574334, 10	106	81	2694	4.08	0.03	0.00	3.11	0.07
GOTERM_BP_ALL	GO:0009205~purine ribonucleoside triphosphate metabolic process	13	2.050473186	4.87E-05	106574334, 10	106	81	2694	4.08	0.03	0.00	3.11	0.07
GOTERM_BP_ALL	GO:0098660~inorganic ion transmembrane transport	10	1.577287066	4.86E-05	106586630, 10	106	46	2694	5.53	0.03	0.00	3.09	0.07
GOTERM_BP_ALL	GO:0098655~cation transmembrane transport	10	1.577287066	4.86E-05	106586630, 10	106	46	2694	5.53	0.03	0.00	3.09	0.07
GOTERM_BP_ALL	GO:0034220~ion transmembrane transport	10	1.577287066	4.86E-05	106586630, 10	106	46	2694	5.53	0.03	0.00	3.09	0.07
GOTERM_BP_ALL	GO:0098662~inorganic cation transmembrane transport	10	1.577287066	4.86E-05	106586630, 10	106	46	2694	5.53	0.03	0.00	3.09	0.07
GOTERM_BP_ALL	GO:0046128~purine ribonucleoside metabolic process	14	2.208201893	5.48E-05	106574334, 10	106	95	2694	3.75	0.03	0.00	3.08	0.08
GOTERM_BP_ALL	GO:0042278~purine nucleoside metabolic process	14	2.208201893	5.48E-05	106574334, 10	106	95	2694	3.75	0.03	0.00	3.08	0.08

GOTERM_BP_ALL	GO:0022402~cell cycle process	10	1.577287066	5.82E-05	100195267, 10	106	47	2694	5.41	0.03	0.00	3.06	0.09
GOTERM_BP_ALL	GO:0044763~single-organism cellular process	63	9.936908517	7.27E-05	106586630, 10	106	1092	2694	1.47	0.04	0.00	2.98	0.11
GOTERM_BP_ALL	GO:0009199~ribonucleoside triphosphate metabolic process	13	2.050473186	8.93E-05	106574334, 10	106	86	2694	3.84	0.05	0.00	2.90	0.13
GOTERM_BP_ALL	GO:0009141~nucleoside triphosphate metabolic process	13	2.050473186	1.00E-04	106574334, 10	106	87	2694	3.80	0.06	0.00	2.86	0.15
GOTERM_BP_ALL	GO:0009259~ribonucleotide metabolic process	14	2.208201893	1.05E-04	106574334, 10	106	101	2694	3.52	0.06	0.00	2.85	0.15
GOTERM_BP_ALL	GO:0072521~purine-containing compound metabolic process	14	2.208201893	1.17E-04	106574334, 10	106	102	2694	3.49	0.07	0.00	2.82	0.17
GOTERM_BP_ALL	GO:0044699~single-organism process	71	11.19873817	1.16E-04	106586630, 10	106	1312	2694	1.38	0.06	0.00	2.82	0.17
GOTERM_BP_ALL	GO:0009119~ribonucleoside metabolic process	14	2.208201893	1.58E-04	106574334, 10	106	105	2694	3.39	0.09	0.00	2.70	0.23
GOTERM_BP_ALL	GO:0055085~transmembrane transport	12	1.892744479	1.83E-04	106565839, 10	106	79	2694	3.86	0.10	0.00	2.65	0.27
GOTERM_BP_ALL	GO:0015672~monovalent inorganic cation transport	10	1.577287066	2.09E-04	106586630, 10	106	55	2694	4.62	0.11	0.00	2.61	0.31
GOTERM_BP_ALL	GO:0000278~mitotic cell cycle	8	1.261829653	2.07E-04	100195267, 10	106	33	2694	6.16	0.11	0.00	2.60	0.30
GOTERM_BP_ALL	GO:0010564~regulation of cell cycle process	5	0.788643533	2.36E-04	100195267, 10	106	9	2694	14.12	0.13	0.00	2.56	0.35
GOTERM_BP_ALL	GO:1901657~glycosyl compound metabolic process	14	2.208201893	3.34E-04	106574334, 10	106	113	2694	3.15	0.18	0.00	2.42	0.49
GOTERM_BP_ALL	GO:0009116~nucleoside metabolic process	14	2.208201893	3.34E-04	106574334, 10	106	113	2694	3.15	0.18	0.00	2.42	0.49
GOTERM_BP_ALL	GO:0007346~regulation of mitotic cell cycle	5	0.788643533	3.82E-04	100195267, 10	106	10	2694	12.71	0.20	0.00	2.37	0.56
GOTERM_BP_ALL	GO:0055114~oxidation-reduction process	9	1.41955836	9.54E-04	106564857, 10	106	54	2694	4.24	0.43	0.01	1.98	1.39
GOTERM_BP_ALL	GO:1903047~mitotic cell cycle process	7	1.104100946	0.001010318	100195267, 10	106	31	2694	5.74	0.44	0.01	1.97	1.48
GOTERM_BP_ALL	GO:1901987~regulation of cell cycle phase transition	4	0.630914826	0.001796577	100195267, 10	106	7	2694	14.52	0.65	0.02	1.73	2.61
GOTERM_BP_ALL	GO:1901990~regulation of mitotic cell cycle phase transition	4	0.630914826	0.001796577	100195267, 10	106	7	2694	14.52	0.65	0.02	1.73	2.61
GOTERM_BP_ALL	GO:0007059~chromosome segregation	4	0.630914826	0.004071921	100286727, 10	106	9	2694	11.30	0.91	0.04	1.38	5.82
GOTERM_BP_ALL	GO:0051726~regulation of cell cycle	6	0.94637224	0.005350135	100195267, 10	106	30	2694	5.08	0.96	0.05	1.27	7.58
	**(Benjamini value > 0,05)**	**Count**	**Percentage (%)**	**P Value**	**Genes**	**List total**	**Pop hits**	**Pop Total**	**Fold Enrichment**	**Bonferroni**	**Benjamini**	**log10 benjami**	**FDR**
GOTERM_BP_ALL	GO:0006812~cation transport	10	1.577287066	0.006909296	106586630, 10	106	89	2694	2.86	0.98	0.07	1.17	9.69
GOTERM_BP_ALL	GO:0045333~cellular respiration	6	0.94637224	0.008121348	106564857, 10	106	33	2694	4.62	0.99	0.08	1.11	11.30
GOTERM_BP_ALL	GO:0015980~energy derivation by oxidation of organic compounds	6	0.94637224	0.009232279	106564857, 10	106	34	2694	4.49	1.00	0.09	1.07	12.75
GOTERM_BP_ALL	GO:0051301~cell division	6	0.94637224	0.009232279	106584418, 10	106	34	2694	4.49	1.00	0.09	1.07	12.75
GOTERM_BP_ALL	GO:0000280~nuclear division	5	0.788643533	0.009267896	100196609, 10	106	22	2694	5.78	1.00	0.08	1.07	12.79
GOTERM_BP_ALL	GO:0007067~mitotic nuclear division	5	0.788643533	0.009267896	100196609, 10	106	22	2694	5.78	1.00	0.08	1.07	12.79
GOTERM_BP_ALL	GO:0048285~organelle fission	5	0.788643533	0.012681838	100196609, 10	106	24	2694	5.29	1.00	0.11	0.95	17.11
GOTERM_BP_ALL	GO:0044772~mitotic cell cycle phase transition	4	0.630914826	0.015306152	100195267, 10	106	14	2694	7.26	1.00	0.13	0.88	20.29
GOTERM_BP_ALL	GO:0044770~cell cycle phase transition	4	0.630914826	0.015306152	100195267, 10	106	14	2694	7.26	1.00	0.13	0.88	20.29
GOTERM_BP_ALL	GO:0006811~ion transport	10	1.577287066	0.017333751	106586630, 10	106	103	2694	2.47	1.00	0.15	0.83	22.67
GOTERM_BP_ALL	GO:0051338~regulation of transferase activity	4	0.630914826	0.018599151	100195267, 10	106	15	2694	6.78	1.00	0.15	0.81	24.12
GOTERM_BP_ALL	GO:0006108~malate metabolic process	3	0.47318612	0.027799891	106564857, 10	106	7	2694	10.89	1.00	0.22	0.66	33.93
GOTERM_BP_ALL	GO:0043648~dicarboxylic acid metabolic process	3	0.47318612	0.036135403	106564857, 10	106	8	2694	9.53	1.00	0.27	0.56	41.79
GOTERM_BP_ALL	GO:0044765~single-organism transport	14	2.208201893	0.045705015	106565839, 10	106	201	2694	1.77	1.00	0.33	0.48	49.73
GOTERM_BP_ALL	GO:0000226~microtubule cytoskeleton organization	4	0.630914826	0.045933499	106588646, 10	106	21	2694	4.84	1.00	0.33	0.49	49.91
GOTERM_BP_ALL	GO:1902578~single-organism localization	14	2.208201893	0.059328904	106565839, 10	106	209	2694	1.70	1.00	0.40	0.40	59.31
GOTERM_BP_ALL	GO:0005975~carbohydrate metabolic process	9	1.41955836	0.070590721	106564857, 10	106	113	2694	2.02	1.00	0.45	0.35	65.91
GOTERM_BP_ALL	GO:0010389~regulation of G2/M transition of mitotic cell cycle	2	0.315457413	0.076445821	100195267, 10	106	2	2694	25.42	1.00	0.47	0.33	68.93
GOTERM_BP_ALL	GO:0009070~serine family amino acid biosynthetic process	2	0.315457413	0.076445821	100196629, 10	106	2	2694	25.42	1.00	0.47	0.33	68.93
GOTERM_BP_ALL	GO:1902749~regulation of cell cycle G2/M phase transition	2	0.315457413	0.076445821	100195267, 10	106	2	2694	25.42	1.00	0.47	0.33	68.93
GOTERM_BP_ALL	GO:0015991~ATP hydrolysis coupled proton transport	4	0.630914826	0.078147659	106590033, 10	106	26	2694	3.91	1.00	0.48	0.32	69.77
GOTERM_BP_ALL	GO:0090662~ATP hydrolysis coupled transmembrane transport	4	0.630914826	0.078147659	106590033, 10	106	26	2694	3.91	1.00	0.48	0.32	69.77
GOTERM_BP_ALL	GO:0015988~energy coupled proton transmembrane transport, against electrochemical gradient	4	0.630914826	0.078147659	106590033, 10	106	26	2694	3.91	1.00	0.48	0.32	69.77
GOTERM_BP_ALL	GO:0031399~regulation of protein modification process	4	0.630914826	0.085521764	100195267, 10	106	27	2694	3.77	1.00	0.50	0.30	73.13
GOTERM_BP_ALL	GO:0006099~tricarboxylic acid cycle	3	0.47318612	0.088747798	106564857, 10	106	13	2694	5.87	1.00	0.51	0.29	74.49
GOTERM_BP_ALL	GO:0043549~regulation of kinase activity	3	0.47318612	0.088747798	100195267, 10	106	13	2694	5.87	1.00	0.51	0.29	74.49
GOTERM_BP_ALL	GO:0045859~regulation of protein kinase activity	3	0.47318612	0.088747798	100195267, 10	106	13	2694	5.87	1.00	0.51	0.29	74.49
GOTERM_BP_ALL	GO:0006091~generation of precursor metabolites and energy	6	0.94637224	0.091553504	106564857, 10	106	62	2694	2.46	1.00	0.52	0.28	75.62
GOTERM_BP_ALL	GO:1901566~organonitrogen compound biosynthetic process	21	3.312302839	0.09524353	106586630, 10	106	381	2694	1.40	1.00	0.53	0.28	77.04

Supplementary Table S4

Ploidy group: Diploid

GO terms for down regulated DEGs for parr when compared to smolt

		**Count**	**Percentage (%)**	**P Value**	**Genes**	**List total**	**Pop hits**	**Pop Total**	**Fold Enrichment**	**Bonferroni**	**Benjamini**	**log10 benjami**	**FDR**
GOTERM_BP_ALL	GO:0042221~response to chemical	7	2.63	3.93E-05	106577762, 10	16	130	2694	9.07	0.01	0.01	2.21	0.05
GOTERM_BP_ALL	GO:0070887~cellular response to chemical stimulus	4	1.50	5.18E-04	100195999, 10	16	30	2694	22.45	0.08	0.04	1.40	0.62
GOTERM_BP_ALL	GO:0042330~taxis	6	2.26	6.07E-04	100270810, 10	16	136	2694	7.43	0.09	0.03	1.50	0.73
GOTERM_BP_ALL	GO:0006935~chemotaxis	5	1.88	1.10E-03	100270810, 10	16	88	2694	9.57	0.16	0.04	1.37	1.32
GOTERM_BP_ALL	GO:0006915~apoptotic process	4	1.50	1.13E-03	100195999, 10	16	39	2694	17.27	0.16	0.04	1.45	1.36
GOTERM_BP_ALL	GO:0016477~cell migration	4	1.50	1.13E-03	100195999, 10	16	39	2694	17.27	0.16	0.04	1.45	1.36
GOTERM_BP_ALL	GO:0009605~response to external stimulus	4	1.50	1.13E-03	100194912, 10	16	39	2694	17.27	0.16	0.04	1.45	1.36
GOTERM_BP_ALL	GO:0048870~cell motility	4	1.50	1.31E-03	100195999, 10	16	41	2694	16.43	0.19	0.03	1.47	1.57
GOTERM_BP_ALL	GO:0051674~localization of cell	5	1.88	1.47E-03	100270810, 10	16	95	2694	8.86	0.21	0.03	1.49	1.76
GOTERM_BP_ALL	GO:0006955~immune response	4	1.50	1.51E-03	100195999, 10	16	43	2694	15.66	0.21	0.03	1.53	1.80
GOTERM_BP_ALL	GO:0044763~single-organism cellular process	4	1.50	1.51E-03	100195999, 10	16	43	2694	15.66	0.21	0.03	1.53	1.80
GOTERM_BP_ALL	GO:0040011~locomotion	5	1.88	1.71E-03	106577762, 10	16	99	2694	8.50	0.24	0.03	1.53	2.05
GOTERM_BP_ALL	GO:0006928~movement of cell or subcellular component	13	4.89	2.15E-03	100194852, 10	16	1092	2694	2.00	0.29	0.03	1.48	2.56
GOTERM_BP_ALL	GO:0012501~programmed cell death	4	1.50	2.48E-03	100195999, 10	16	51	2694	13.21	0.32	0.04	1.46	2.95
GOTERM_BP_ALL	GO:0008219~cell death	4	1.50	2.77E-03	100195999, 10	16	53	2694	12.71	0.35	0.04	1.45	3.29
GOTERM_BP_ALL	GO:0050896~response to stimulus	4	1.50	3.24E-03	100194912, 10	16	56	2694	12.03	0.40	0.04	1.41	3.84
	GO:0044699~single-organism process	4	1.50	3.41E-03	100194912, 10	16	57	2694	11.82	0.42	0.04	1.42	4.03
GOTERM_BP_ALL	GO:0048523~negative regulation of cellular process	9	3.38	4.04E-03	100194852, 10	16	537	2694	2.82	0.47	0.04	1.38	4.76
	**(Benjamini value > 0,05)**	**Count**	**Percentage (%)**	**P Value**	**Genes**	**List total**	**Pop hits**	**Pop Total**	**Fold Enrichment**	**Bonferroni**	**Benjamini**	**log10 benjami**	**FDR**
GOTERM_BP_ALL	(Bejamini value > 0,05)	Count	Percentage (%)	P Value	Genes	List total	Pop hits	Pop Total	Fold Enrichment	Bonferroni	Benjamini	log10 benjami	FDR
GOTERM_BP_ALL	GO:0048519~negative regulation of biological process	13	4.89	1.35E-02	100194852, 10	16	1312	2694	1.67	0.88	0.13	0.90	15.07
GOTERM_BP_ALL	GO:0009112~nucleobase metabolic process	4	1.50	1.50E-02	100194912, 10	16	97	2694	6.94	0.91	0.13	0.88	16.68
GOTERM_BP_ALL	GO:0043066~negative regulation of apoptotic process	4	1.50	2.37E-02	100194912, 10	16	115	2694	5.86	0.98	0.19	0.72	25.09
GOTERM_BP_ALL	GO:0060548~negative regulation of cell death	2	0.75	3.84E-02	100195250, 10	16	7	2694	48.11	1.00	0.28	0.56	37.59
GOTERM_BP_ALL	GO:1901652~response to peptide	2	0.75	3.84E-02	100194912, 10	16	7	2694	48.11	1.00	0.28	0.56	37.59
GOTERM_BP_ALL	GO:0043069~negative regulation of programmed cell death	2	0.75	3.84E-02	100194912, 10	16	7	2694	48.11	1.00	0.28	0.56	37.59
GOTERM_BP_ALL	GO:0043434~response to peptide hormone	2	0.75	3.84E-02	100270810, 10	16	7	2694	48.11	1.00	0.28	0.56	37.59
GOTERM_BP_ALL	GO:0010243~response to organonitrogen compound	2	0.75	3.84E-02	100194912, 10	16	7	2694	48.11	1.00	0.28	0.56	37.59
GOTERM_BP_ALL	GO:0051716~cellular response to stimulus	2	0.75	3.84E-02	100270810, 10	16	7	2694	48.11	1.00	0.28	0.56	37.59
GOTERM_BP_ALL	GO:1901698~response to nitrogen compound	2	0.75	5.44E-02	100270810, 10	16	10	2694	33.68	1.00	0.36	0.45	49.03
GOTERM_BP_ALL	GO:0050776~regulation of immune response	6	2.26	5.73E-02	100194852, 10	16	397	2694	2.54	1.00	0.36	0.45	50.90
		2	0.75	7.02E-02	100270810, 10	16	13	2694	25.90	1.00	0.41	0.39	58.38
		2	0.75	9.08E-02	100136548, 10	16	17	2694	19.81	1.00	0.48	0.32	68.24

Ploidy group: Triploid

GO terms for down regulated DEGs for parr when compared to smolt (Benjamini value ≤0,05)

		Count	Percentage	P Value	Genes	List total	Pop hits	Pop Total	Fold Enrich	Bonferroni	Benjamini	log10 benj	FDR
GOTERM_E	GO:0050896~response to stimulus	34	5.09	0.00	106606886, 1	65	537	2694	2.62	0.00	0.00	5.31	0.00
GOTERM_E	GO:0002376~immune system process	17	2.54	0.00	106606886, 1	65	130	2694	5.42	0.00	0.00	5.28	0.00
GOTERM_E	GO:0042330~taxis	10	1.50	0.00	106606886, 1	65	39	2694	10.63	0.00	0.00	4.62	0.00
GOTERM_E	GO:0006935~chemotaxis	10	1.50	0.00	106606886, 1	65	39	2694	10.63	0.00	0.00	4.62	0.00
GOTERM_E	GO:0040011~locomotion	11	1.65	0.00	106606886, 1	65	51	2694	8.94	0.00	0.00	4.74	0.00
GOTERM_E	GO:0009605~response to external stimulus	13	1.95	0.00	106606886, 1	65	95	2694	5.67	0.00	0.00	3.93	0.00
GOTERM_E	GO:0042221~response to chemical	15	2.25	0.00	106606886, 1	65	136	2694	4.57	0.00	0.00	3.84	0.00
GOTERM_E	GO:0016477~cell migration	9	1.35	0.00	106606886, 1	65	41	2694	9.10	0.00	0.00	3.67	0.00
GOTERM_E	GO:0060326~cell chemotaxis	8	1.20	0.00	106606886, 1	65	30	2694	11.05	0.00	0.00	3.65	0.01
GOTERM_E	GO:0051716~cellular response to stimulus	25	3.74	0.00	106606886, 1	65	397	2694	2.61	0.00	0.00	3.64	0.01
GOTERM_E	GO:0051674~localization of cell	9	1.35	0.00	106606886, 1	65	43	2694	8.67	0.00	0.00	3.66	0.01
GOTERM_E	GO:0048870~cell motility	9	1.35	0.00	106606886, 1	65	43	2694	8.67	0.00	0.00	3.66	0.01
GOTERM_E	GO:0006928~movement of cell or subcellular component	9	1.35	0.00	106606886, 1	65	53	2694	7.04	0.01	0.00	3.00	0.04
GOTERM_E	GO:0070887~cellular response to chemical stimulus	11	1.65	0.00	106606886, 1	65	88	2694	5.18	0.01	0.00	2.96	0.04
GOTERM_E	GO:0006955~immune response	11	1.65	0.00	100136933, 1	65	99	2694	4.61	0.04	0.00	2.55	0.12
GOTERM_E	GO:0050900~leukocyte migration	4	0.60	0.00	106606886, 1	65	8	2694	20.72	0.25	0.02	1.69	0.93
GOTERM_E	GO:0044763~single-organism cellular process	40	5.99	0.00	106606886, 1	65	1092	2694	1.52	0.26	0.02	1.70	0.96

	(Benjamini value > 0,05)	Count	Percentag	P Value	Genes	List total	Pop hits	Pop Total	Fold Enrich	Bonferroni	Benjamini	log10 benj	FDR
GOTERM_B	GO:0006952~defense response	8	1.20	0.00	100136933, 1	65	83	2694	3.99	0.74	0.08	1.09	4.25
GOTERM_B	GO:0030595~leukocyte chemotaxis	3	0.45	0.00	106606886, 1	65	4	2694	31.08	0.76	0.08	1.09	4.48
GOTERM_B	GO:0007165~signal transduction	16	2.40	0.00	100194852, 1	65	302	2694	2.20	0.81	0.09	1.06	5.13
GOTERM_B	GO:0023052~signaling	16	2.40	0.00	100194852, 1	65	306	2694	2.17	0.85	0.09	1.03	5.80
GOTERM_B	GO:0044700~single organism signaling	16	2.40	0.00	100194852, 1	65	306	2694	2.17	0.85	0.09	1.03	5.80
GOTERM_B	GO:0044699~single-organism process	43	6.44	0.00	100194852, 1	65	1312	2694	1.36	0.85	0.09	1.04	5.91
GOTERM_B	GO:0007154~cell communication	16	2.40	0.00	100194852, 1	65	310	2694	2.14	0.88	0.10	1.02	6.55
GOTERM_B	GO:0007586~digestion	3	0.45	0.01	100137024, 1	65	5	2694	24.87	0.91	0.10	0.99	7.25
GOTERM_B	GO:0006954~inflammatory response	5	0.75	0.01	100195359, 1	65	35	2694	5.92	0.98	0.16	0.81	11.67
GOTERM_B	GO:0043434~response to peptide hormone	3	0.45	0.01	100270810, 1	65	7	2694	17.76	0.99	0.18	0.74	14.25
GOTERM_B	GO:1901652~response to peptide	3	0.45	0.01	100270810, 1	65	7	2694	17.76	0.99	0.18	0.74	14.25
GOTERM_B	GO:0010243~response to organonitrogen compound	3	0.45	0.02	100270810, 1	65	10	2694	12.43	1.00	0.33	0.49	27.13
GOTERM_B	GO:0050794~regulation of cellular process	26	3.89	0.03	100194852, 1	65	749	2694	1.44	1.00	0.43	0.37	36.87
GOTERM_B	GO:1901698~response to nitrogen compound	3	0.45	0.04	100270810, 1	65	13	2694	9.56	1.00	0.46	0.34	41.03
GOTERM_B	GO:0002250~adaptive immune response	2	0.30	0.05	100195196, 1	65	2	2694	41.45	1.00	0.53	0.27	49.38
GOTERM_B	GO:0001845~phagolysosome assembly	2	0.30	0.05	106606886, 1	65	2	2694	41.45	1.00	0.53	0.27	49.38
GOTERM_B	GO:0016050~vesicle organization	2	0.30	0.05	106606886, 1	65	2	2694	41.45	1.00	0.53	0.27	49.38
GOTERM_B	GO:0050789~regulation of biological process	26	3.89	0.05	100194852, 1	65	783	2694	1.38	1.00	0.56	0.25	53.63
GOTERM_B	GO:0048583~regulation of response to stimulus	5	0.75	0.06	100136548, 1	65	62	2694	3.34	1.00	0.59	0.23	56.99
GOTERM_B	GO:0045087~innate immune response	4	0.60	0.06	100136933, 1	65	38	2694	4.36	1.00	0.59	0.23	58.23
GOTERM_B	GO:0050776~regulation of immune response	3	0.45	0.06	100136548, 1	65	17	2694	7.31	1.00	0.58	0.24	58.41
GOTERM_B	GO:0006915~apoptotic process	4	0.60	0.06	100136548, 1	65	39	2694	4.25	1.00	0.59	0.23	60.63
GOTERM_B	GO:0065007~biological regulation	27	4.04	0.07	100194852, 1	65	839	2694	1.33	1.00	0.59	0.23	62.15
GOTERM_B	GO:0080171~lytic vacuole organization	2	0.30	0.07	106606886, 1	65	3	2694	27.63	1.00	0.60	0.22	63.99
GOTERM_B	GO:0090382~phagosome maturation	2	0.30	0.07	106606886, 1	65	3	2694	27.63	1.00	0.60	0.22	63.99
GOTERM_B	GO:0007040~lysosome organization	2	0.30	0.07	106606886, 1	65	3	2694	27.63	1.00	0.60	0.22	63.99
GOTERM_B	GO:0007033~vacuole organization	2	0.30	0.07	106606886, 1	65	3	2694	27.63	1.00	0.60	0.22	63.99
GOTERM_B	GO:0009725~response to hormone	3	0.45	0.08	100270810, 1	65	20	2694	6.22	1.00	0.64	0.19	69.40
GOTERM_B	GO:1901700~response to oxygen-containing compound	3	0.45	0.09	100270810, 1	65	21	2694	5.92	1.00	0.67	0.18	72.59

Supplementary Table S5. Down-regulated KEGG pathways in diploid fry when compared to parr. KEGG ortholog (KO no.) numbers are indicated as well.

KO no.	Name	KEGG pathway(s)	Function
00010	myo-inositol 2-dehydrogenase	Glycolysis/Gluconeogenesis (1)	Carbohydrate metabolism
00660	chalcone synthase	C5-branched dibasic acid metabolism (1)	Biosynthesis of secondary metabolites, Environmental adaptation
00710	polypeptide N-acetylgalactosaminytransferase	Carbon synthesis in photosynthetic organisms (1)	Glycan biosynthesis and protein metabolism
00310	pyrimidodiazepine synthase	Lysine degradation (1)	Amino acid metabolism
03022	DNA directed RNA polymerase III subunit	Basal transcription factors (1)	Genetic information processes, Immune system processes
03010	DNA directed RNA polymerase II subunit	Ribosome (1)	Genetic information processes
04066	prisomonal protein N’ (replication factor Y) (superfamily II helicase)	HIF-1 signalling pathway (1)	Genetic information processes
03450	solute carrier family 7 (L-type amino acid transfer)	Non-homologous end-joining (1)	Cellular processes
04530	amyloid beta A4 precursor protein-binding family B member 2	Tight junction (1)	Cellular processes
04921	potassium channel subfamily K member 12	Oxytocin signalling pathway (1)	Cellular processes
04614	vomeronasal 1 receptor	Renin-angiotensin system (1)	Cellular processes
04142/ 04217	adrenergic receptor beta-2/ Somatostatin receptor1	Lysosome (1)/ Necroptosis (1)	Environmental information processing, and organismal systems
04140/ 04137	adrenergic receptor alpha-2C / adrenergic receptor alpha-1D	Autophagy-animal (1)/ Mitophagy-animal (1)	Environmental Information and cellular processes
04270	purinoceptor 1	Vascular smooth muscle contraction (1)	Environmental information processing, organismal system processes and cellular processes
05010	channel 1, skeletal muscle	Alzheimer’s disease (1)	Human diseases

Supplementary Table S6. Down-regulated KEGG pathways in triploid fry when compared to parr. KEGG ortholog numbers (KO no.) are indicated as well.

KO no.	Name	KEGG pathway(s)	Function(s)
04120	ent-copalyl diphosphate synthase	Ubiquitin mediated proteolysis (1)	Metabolism of terpenoids and polyketides
05100	macrophage-stimulating 1 receptor	Bacterial invasion of epithelial cells	Protein metabolism
03022	DNA-directed RNA polymerase III subunit	Basal transcription factors (1)	Genetic information processes and Organismal systems
04921	potassium channel subfamily K member 12	Oxytocin signalling pathway (1)	Cellular processes
04530	amyloid beta A4 precursor protein-binding family B member 2	Tight junction (1)	Cellular processes
04141	adrenergic receptor beta-1	Protein processing in endoplasmic reticulum (1)	Environmental information processing, cellular processes and organismal systems
04142	adrenergic receptor beta-2	Lysosome (1)	Environmental information processing, and organismal systems
04140/ 04137	adrenergic receptor alpha-2C / adrenergic receptor alpha-1D	Autophagy-animal (1)/ Mitophagy-animal (1)	Environmental Information and cellular processing
04270	purinoceptor 1	Vascular smooth muscle contraction (1)	Environmental information processing, organismal system processes and cellular processes
05131	interferon receptor 2	Shigellosis (1)	Environmental information processing, organismal system processes, cellular processes and human diseases.
04725	ubiquitin-protein ligase XIAP	Cholinergic synapse (1)	Human diseases

Supplementary Table S7. Down-regulated KEGG pathways in diploid parr when compared to smolt. KEGG ortholog numbers (KO no.) are indicated as well.

KO no.	Name	KEGG pathway(s)	Function(s)
00600	glycine hydroxymethyltransferase	Sphingolipid metabolism (1)	Carbohydrate, energy, cofactors & vitamins and amino acid metabolism
00270	phenylalanine dehydrogenase	Cysteine and methionine metabolism (1)	Amino acid metabolism
00330	NADH-quinone oxidoreductase subunit A	Argenine and proline metabolism (1)	Energy metabolism
00410	ubiquinol-cytochrome c reductase cytochrome b/c1 subunit	Beta-Alanine metabolism (1)	Metabolism of terpenoids and polyketides
00480	salicylate hydroxylase	Gluthione metabolism (1)	Xenobiotic degradation & metabolism and oxidoreductase
04120	ent-copalyl diphosphate synthase	Ubiquitin mediated proteolysis (1)	Metabolism of terpenoids and polyketides
04015	protein NrfD	Rap1 signalling pathway (1)	Energy metabolism
03010	DNA directed RNA polymerase II subunit	Ribosome (2)	Genetic information processing
03460	solute carrier organic anion transporter family, member 1A	Faconi anemia pathway (1)	Organismal systems
04390/<br>04391/<br>04144/<br>04360	tumor necrosis factor receptor superfamily member 6/<br>monocyte differentiation antigen CD14/<br>dopamine receptor D1/<br>neurotrophic tyrosine kinase receptor type 2	Hippo signalling pathway (1)/<br>Hippo signalling pathway -fly (1)/<br>Endocytosis (1)/<br>Axon guidance (1)	Environmental information processing, organismal system processes, cellular processes and human diseases.
04071	n/d	Sphingolipid signalling path-way (1)	n/d
04140/<br>04137	adrenergic receptor alpha-2C/<br>adrenergic receptor alpha-1D	Autophagy-animal (1)/<br>Mitophagy-animal (1)	Environmental Information and cellular processing
04530	amyloid beta A4 precursor protein-binding family B member 2	Tight junction (1)	Cellular processes

n/d - not determined

Supplementary Table S8. Down-regulated KEGG pathways in triploid parr when compared to smolt. KEGG ortholog numbers (KO no.) are indicated as well.

KO no.	Name	KEGG pathway(s)	Function(s)
03010	DNA directed RNA polymerase II subunit	Ribosome (2)	Genetic information processes
03018	DNA-directed RNA polymerase III subunit	RNA degradation (1)	Genetic information processes
04080	molecular chaperone IbpA	Neuroactive ligand-receptor interaction (1)	Genetic information processes
02010	iron(III) transport system ATP-binding protein	ABC transporters (1)	Environmental Information processes
04330	n/d	Notch signalling pathway (1)	n/d
04020	phosphotransacetylase	Calcium signalling pathway (1)	Carbohydrate metabolism
04024	ethanolamine utilization protein EutJ	cAMP signalling pathway (1)	Amino acid metabolism
04022	alcohol dehydrogenase	cGMP – PKG signalling pathway (1)	Carbohydrate metabolism
04140/ 04137	adrenergic receptor alpha-2C / adrenergic receptor alpha-1D	Autophagy-animal (1)/ Mitophagy-animal (1)	Environmental Information and cellular processing

n/d - not determined
